# Quantify single nucleotide polymorphism (SNP) ratio in pooled DNA based on normalized fluorescence real-time PCR

**DOI:** 10.1186/1471-2164-7-143

**Published:** 2006-06-09

**Authors:** Airong Yu, Haifeng Geng, Xuerui Zhou

**Affiliations:** 1 Department of Biology, Huaiyin Teachers College, 71 Jiao tong Road, Huai'an, Jiangsu Province, 223001, P.R. China; 2 Center of Marine Biotechnology, University of Maryland Biotechnology Institute, MD, 21202, USA

## Abstract

**Background:**

Conventional real-time PCR to quantify the allele ratio in pooled DNA mainly depends on PCR amplification efficiency determination and Ct value, which is defined as the PCR cycle number at which the fluorescence emission exceeds the fixed threshold. Because of the nature of exponential calculation, slight errors are multiplied and the variations of the results seem too large. We have developed a new PCR data point analysis strategy for allele ratio quantification based on normalized fluorescence ratio.

**Results:**

In our method, initial reaction background fluorescence was determined based upon fitting of raw fluorescence data to four-parametric sigmoid function. After that, each fluorescence data point was first subtracted by respective background fluorescence and then each subtracted fluorescence data point was divided by the specific background fluorescence to get normalized fluorescence. By relating the normalized fluorescence ratio to the premixed known allele ratio of two alleles in standard samples, standard linear regression equation was generated, from which unknown specimens allele ratios were extrapolated using the measured normalized fluorescence ratio. In this article, we have compared the results of the proposed method with those of baseline subtracted fluorescence ratio method and conventional Ct method.

**Conclusion:**

Results demonstrated that the proposed method could improve the reliability, precision, and repeatability for quantifying allele ratios. At the same time, it has the potential of fully automatic allelic ratio quantification.

## Background

Single nucleotide polymorphisms (SNPs), the most common source of polymorphism in the human genome, have a wide applications in human genetics, pharmacogenomics, analysis of clinical samples, and identification of human susceptibility genes involved in complex diseases [1-4]. Pooling equal amounts of DNA from all the individual samples and typing one SNP marker at a time can save valuable template DNA and has been successfully utilized in microsatellite markers [5] and SNPs [6,7]. Thus, a quantifying SNPs method in pooled and mixed DNA samples with high precision and efficiency is required in human genetics [8]. Another demand of quantifying SNPs in pooled DNA samples lies in the study of population dynamics of pathogens, from which quantifying SNP frequencies may not only help monitor the pathogen dynamics associated with treatment but could also improve therapeutic decision making [9]. Several genotyping methods were found to be suitable for measuring SNP allele frequencies of wild-type/mutant allele ratios in DNA pools, including SSCP or dHPLC, TAQMAN™ (Applied Biosystems) [10], oligo-ligation assay, Invader assay™ (Third Wave Technologies Inc.), allele-specific amplification with real-time PCR [11] and Pyrosequencing™ (Pyrosequencing). The real-time PCR platform has a promising future for high throughput, sensitive and accurate estimation of SNP allele frequencies in DNA pools [12]. It utilizes PCR primers together with LNA or MGB modified dual labeled specific probes spanning the SNP of interest [13]. In conventional real-time PCR, the threshold cycle (Ct) or crossing point (CP) from pools were used to calculate allele ratios from the value of 2^-ΔCt ^[11,14] or similar to E^-ΔCt ^taking PCR amplification efficiency into account. Those methods ideally assume the two-fluorophore channels have the comparable real-time PCR kinetics. This is, however, a simplified approach, since it has been demonstrated that binding efficiencies [15] differ between the probes, and also one fluorphore channel preferably amplifies of over the other [13] fluorophore. Apart from that, even small standard deviations of ΔCt will amplify exponentially too large variation and hence it cannot give satisfied precision. To circumvent Ct value, Oliver et al. (2000) have initiated allele ratio quantification based on the fluorescence signal ratio given by hydrolyzed allele specific probes. But their method took only the end point fluorescence into consideration where PCR reaction usually processed into plateau phase and reliable quantification result therefore is not arguably reached.

In this article, we compare the normalized fluorescence data point of the two fluorescence channels during the early PCR exponential phase, and furthermore describe a rational real time data point analysis strategy for quantifying allele ratios in DNA pools. It shows that by using the novel fluorescence normalization method and reliance on the more selected data points fluorescence ratio analysis, significant improvement in precision and repeatability was demonstrated in the novel analysis method compared to conventional method although the proposed strategy does not claim to completely replace the conventional method.

## Results

### Principle of simulation

In the optimized Taqman real-time PCR, the measured incremental signal ΔRn is directly proportional to the amount of produced amplicon as described [16], at any cycle as followed:

[amplicon] _synthesized _= ΔR_n_/Δ∅     1

Here Δ∅ represents parameter of the difference between the specific fluorescence of the free fluorophore and the specific fluorescence of the probe-bound fluorophore, ΔRn is the fluorescence increment.

The basic equation for PCR amplification [17] during exponential phase is:

N_n _= N_0_·E^n ^    2

Where N_0 _is the initial number of DNA, n is number of cycles. As for SNP allele ratio quantification, the amount of product for the amplicon allele A and amplicon allele B will be:

[amplicon]_An _= A_0_·E_A_^n ^    3

[amplicon]_Bn _= B_0_·E_B_^n ^    4

So, the initial ratios allele A and B can be concluded as:

A_0_/B_0 _= [amplicon]_An_/[amplicon]_Bn _× (E_B_^n^/E_A_^n^)     5

If let Ri_A/B _to represent A_0_/B_0_, the initial ratio of allele A and allele B, equation 5 can be transformed by inserting equation 1:

ΔR_nA_/ΔR_nB _= Ri_A/B _× (Δ∅_A_/Δ∅_B_) (E_A_^n^/E_B_^n^) = Ri_A/B _× (K1 × K2)     6

Whereby K1, equal to Δ∅_A_/Δ∅_B_, represents probe fluorescence and binding efficiency difference between two different probes. K2, equal to E_A_^n^/E_B_^n^, represents different allele amplification efficiency.

When analysis of the florescence value ΔR_nA _and ΔR_nB _in the selected segment of PCR reaction for each one-reaction tube, K1 representing difference of probe binding efficiencies is the constant parameters. Initial allele ratio Ri_A/B _being constant for one reaction tube, K2 would relatively be constant in only a few chosen cycles. So, ΔR_nA _is predicted to have a linear relationship with ΔR_nB _within the selected fluorescence comparison segment in the reaction tube.

From another point of view, if we defined ΔR_nA_/ΔR_nB _to be K_f_., Eq.6 could also converted as:

K_f_/Ri_A/B _= K1 × K2     7

Assuming that PCR reaction efficiency difference K2 remain constant for all samples in the only a few selected fluorescence comparison segment, and further considering that the K1 is the constant parameter, different allele ratio Ri_A/B _is expected to have a linear relationship with the corresponding value of K_f _for all samples. Here, this relationship is the clue to quantitative analysis with the suggested method.

### Normalized fluorescence data from background

Real-time PCR can be modeled by the four parametric sigmoid function [13,18]:

f(Rx)=y0+a1+e−(x−x0b)     8
 MathType@MTEF@5@5@+=feaafiart1ev1aaatCvAUfKttLearuWrP9MDH5MBPbIqV92AaeXatLxBI9gBaebbnrfifHhDYfgasaacH8akY=wiFfYdH8Gipec8Eeeu0xXdbba9frFj0=OqFfea0dXdd9vqai=hGuQ8kuc9pgc9s8qqaq=dirpe0xb9q8qiLsFr0=vr0=vr0dc8meaabaqaciaacaGaaeqabaqabeGadaaakeaacqWGMbGzcqGGOaakcqWGsbGudaWgaaWcbaGaemiEaGhabeaakiabcMcaPiabg2da9iabdMha5naaBaaaleaacqaIWaamaeqaaOGaey4kaSYaaSaaaeaacqWGHbqyaeaacqaIXaqmcqGHRaWkcqWGLbqzdaahaaWcbeqaaiabgkHiTiabcIcaOmaalaaabaGaemiEaGNaeyOeI0IaemiEaG3aaSbaaWqaaiabicdaWaqabaaaleaacqWGIbGyaaGaeiykaKcaaaaakiaaxMaacaWLjaGaeGioaGdaaa@4710@

Where x is cycle number, f (Rx) is fluorescence of cycle x, y_0 _is the background fluorescence, a is the difference between the maximal fluorescence and background fluorescence, x_0 _is the first derivative maximum of the function or the inflexion point of the curve, b describes the slope of the curve.

**Figure 1:**
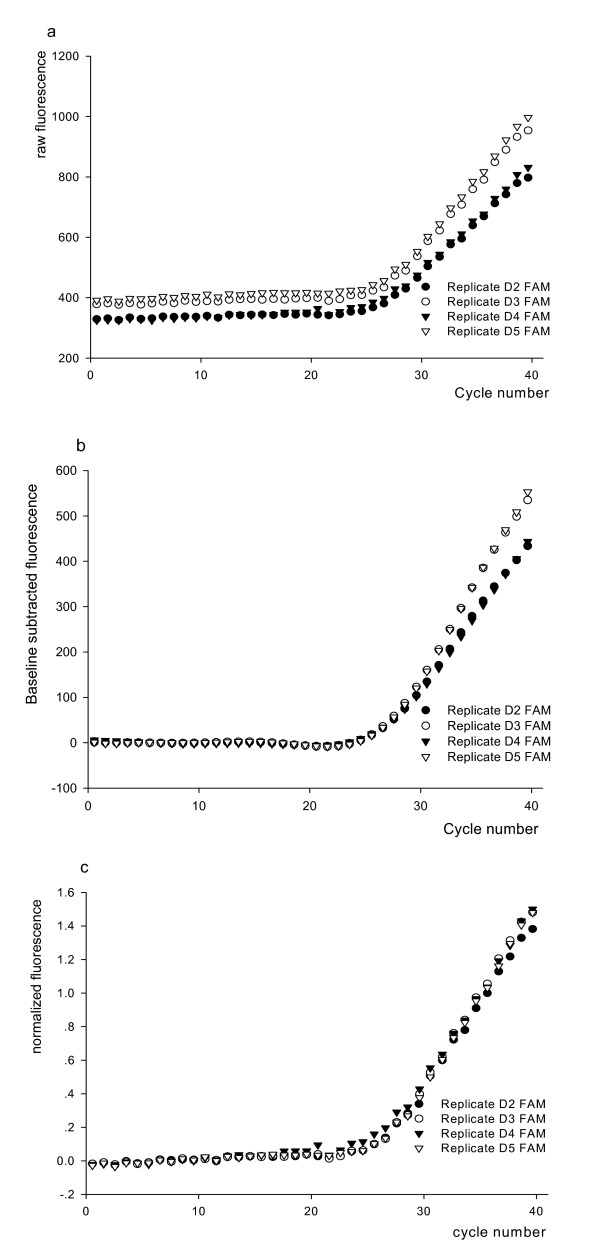
**Effect of fluorescence normalization**. (a) Signal FAM curves of four replicates. (b) Same FAM data as shown in (a) after baseline (cycle 4~20) subtraction. (c) Amplification curve (FAM) of four replicates after fluorescence normalization.

Of particular notice, samples having initial higher fluorescence in baseline cycle, as shown in replicate D3 and replicate D5 from the same sample in figure 1a, are inclined to have higher fluorescence reading in the ensuing cycles. This fluorescence vertical shift was not even eliminated after baseline subtraction as shown in figure 1b. We normalized the fluorescence increment (raw fluorescence-y0) to reaction fluorescence background (y0) for each sample reaction, which we express as: normalized fluorescence=raw fluorescence−y0y0
 MathType@MTEF@5@5@+=feaafiart1ev1aaatCvAUfKttLearuWrP9MDH5MBPbIqV92AaeXatLxBI9gBaebbnrfifHhDYfgasaacH8akY=wiFfYdH8Gipec8Eeeu0xXdbba9frFj0=OqFfea0dXdd9vqai=hGuQ8kuc9pgc9s8qqaq=dirpe0xb9q8qiLsFr0=vr0=vr0dc8meaabaqaciaacaGaaeqabaqabeGadaaakeaacqqGUbGBcqqGVbWBcqqGYbGCcqqGTbqBcqqGHbqycqqGSbaBcqqGPbqAcqqG6bGEcqqGLbqzcqqGKbazcqqGGaaicqqGMbGzcqqGSbaBcqqG1bqDcqqGVbWBcqqGYbGCcqqGLbqzcqqGZbWCcqqGJbWycqqGLbqzcqqGUbGBcqqGJbWycqqGLbqzcqGH9aqpdaWcaaqaaiabbkhaYjabbggaHjabbEha3jabbccaGiabbAgaMjabbYgaSjabbwha1jabb+gaVjabbkhaYjabbwgaLjabbohaZjabbogaJjabbwgaLjabb6gaUjabbogaJjabbwgaLjabgkHiTiabbMha5naaBaaaleaacqaIWaamaeqaaaGcbaGaeeyEaK3aaSbaaSqaaiabicdaWaqabaaaaaaa@67E6@, all fluorescence increasement are displayed as a percent of initial fluorescence. Based on visualization of amplification curve (Fig 1c), homogeneity of intra-runs of four replicates was significantly improved. These results suggested that the proposed method minimized the influence of the initial vertical background shift of reaction. It also suggested that each PCR cycle instrumental fluorescence reading f (Rx) depends on initial fluorescence reading for respective wells, which implied that each PCR cycle fluorescence increasement depends on initial fluorescence reading. We slightly modified equation 8 fluorescence increasement as:

f(Rx)=y0+a′1+e−(x−x0b)y0     9
 MathType@MTEF@5@5@+=feaafiart1ev1aaatCvAUfKttLearuWrP9MDH5MBPbIqV92AaeXatLxBI9gBaebbnrfifHhDYfgasaacH8akY=wiFfYdH8Gipec8Eeeu0xXdbba9frFj0=OqFfea0dXdd9vqai=hGuQ8kuc9pgc9s8qqaq=dirpe0xb9q8qiLsFr0=vr0=vr0dc8meaabaqaciaacaGaaeqabaqabeGadaaakeaacqWGMbGzcqGGOaakcqWGsbGudaWgaaWcbaGaemiEaGhabeaakiabcMcaPiabg2da9iabdMha5naaBaaaleaacqaIWaamaeqaaOGaey4kaSYaaSaaaeaacuWGHbqygaqbaaqaaiabigdaXiabgUcaRiabdwgaLnaaCaaaleqabaGaeyOeI0IaeiikaGYaaSaaaeaacqWG4baEcqGHsislcqWG4baEdaWgaaadbaGaeGimaadabeaaaSqaaiabdkgaIbaacqGGPaqkaaaaaOGaemyEaK3aaSbaaSqaaiabicdaWaqabaGccaWLjaGaaCzcaiabiMda5aaa@49BD@

where a' = a/y_0 _.so, normalized fluorescence can be expressed as:

normalized fluorescence=a′1+e−(x−x0b)     10
 MathType@MTEF@5@5@+=feaafiart1ev1aaatCvAUfKttLearuWrP9MDH5MBPbIqV92AaeXatLxBI9gBaebbnrfifHhDYfgasaacH8akY=wiFfYdH8Gipec8Eeeu0xXdbba9frFj0=OqFfea0dXdd9vqai=hGuQ8kuc9pgc9s8qqaq=dirpe0xb9q8qiLsFr0=vr0=vr0dc8meaabaqaciaacaGaaeqabaqabeGadaaakeaacqqGUbGBcqqGVbWBcqqGYbGCcqqGTbqBcqqGHbqycqqGSbaBcqqGPbqAcqqG6bGEcqqGLbqzcqqGKbazcqqGGaaicqqGMbGzcqqGSbaBcqqG1bqDcqqGVbWBcqqGYbGCcqqGLbqzcqqGZbWCcqqGJbWycqqGLbqzcqqGUbGBcqqGJbWycqqGLbqzcqGH9aqpdaWcaaqaaiqbdggaHzaafaaabaGaeGymaeJaey4kaSIaemyzau2aaWbaaSqabeaacqGHsislcqGGOaakdaWcaaqaaiabdIha4jabgkHiTiabdIha4naaBaaameaacqaIWaamaeqaaaWcbaGaemOyaigaaiabcMcaPaaaaaGccaWLjaGaaCzcaiabigdaXiabicdaWaaa@5D5F@

### Determination of PCR exponential phase for allele ratio quantification

Data points from as many as exponential cycles were selected to data analysis. The start of the exponential is determined by 80% of the second derivative maximum (SDM) of the sigmoidal equation 8 after fitting it to the raw fluorescent data using SigmaPlot (version 8.0, SPSS) [17,18] or is visually chosen from logarithmic amplification graph (Log fluorescence versus Cycle numbers graph). Considering X_0 _is still in the heart of Log linear phase amplification, so, raw fluorescence data points from the determined start cycle of the exponential phase to X_0 _cycle were chosen for further analysis. Considering each well reaction gives two data points of two different fluorescence FAM vs. VIC, overlaps of cycles for all sample replicates and for both dual probes in real-time Taqman PCR assays were included.

### Ratio of two fluorescence during selected exponential phase

**Figure 2:**
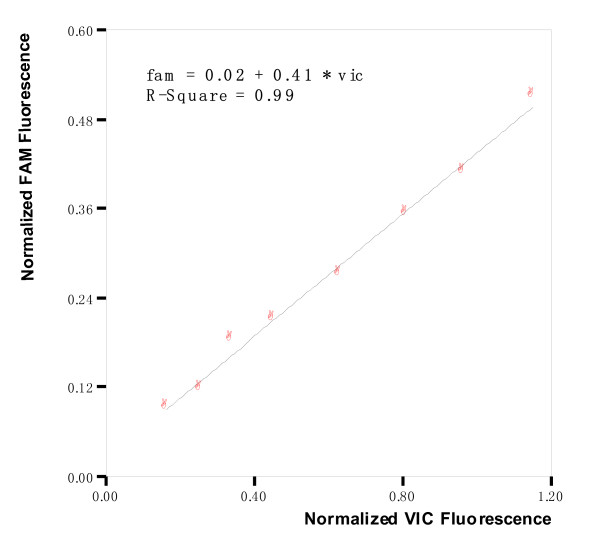
An example of linear regression of two fluorescence for determination of Kf.

Once the beginning and the end of cycles from the exponential phase were selected, normalized fluorescence FAM of those cycles was plotted against normalized fluorescence VIC data points. Normalized fluorescence ratio (K_f_) derived was determined by the slope of the linear regression of the fitting curve R_FAM _= K_f _× R_VIC _+ R_0 _(r^2 ^> 0.99). An average fluorescence ratio (K_f_) value of replicates for the same sample was determined and the standard deviation (S.D.) was also calculated for each sample (Summarized in Table 2. column 4 and column 5). Fig 2 demonstrates FAM vs. VIC linear regression in replicate 1 and Table 2 demonstrates the results of four replicates of sample YMDD: YIDD (50%: 50%). A spreadsheet containing the ratios of normalized fluorescence for all samples is provided as Supplementary Material 1, 2, 3 [see Additional file 1, 2, 3]. It should been noted that K_f_, equals to R_iA/B _× (K1K2) in equation 6, represents the combine effect of allele ratio, the fluorophore probe binding chemistry and allele amplification efficiency. In another data procession workflow, baseline-subtracted fluorescence ratios, which were determined in a way similar to the above mentioned linear regression method, were also provided as Supplementary Material 4, 5,6 [see Additional file 4, 5, 6].

### Standard curve generation

**Figure 3:**
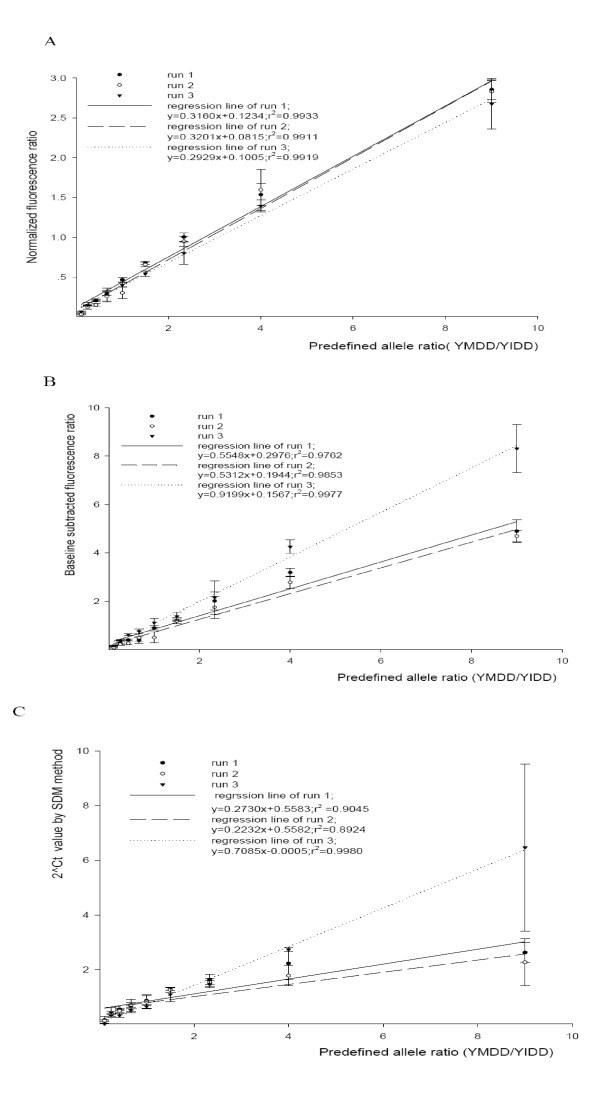
**Standard curves of three methods form three different runs**. (A) Normalized fluorescence ratio method standard curve: Normalized Fluorescence ratio was plotted against predefined ratio of allele YMDD/YIDD. (B) Baseline subtracted fluorescence ratio method standard curve: Baseline subtracted fluorescence ratio was plotted against predefined ratio of allele YMDD/YIDD (C): The value of Ct based method (2^-ΔCt^) was plotted against predefined allele YMDM/YIDD. Ct values were determined by the second derivative maximum. Solid line, long dash line and dotted line respectively represent linear regression line of run 1, run 2 and run 3. Error bars indicate 1 SD for replicates.

To relate normalized fluorescence ratio to allele ratio in the sample, a linear pre-mixed DNA standards with YMDD/YIDD ratio from 9:1 to 1:9 was prepared by mixing the homogenous allele into the proper ratios as methods mentioned above. Totally three runs were performed in three different times. The average K_f _of each group was plotted against the pre-mixed ratio in the sample. The Fig. 3A shows an excellent linear regression (r^2 ^= 0.9933, r^2 ^= 0.9911, r^2 ^= 0.9919) for all three runs. Thus the standard curve allows extrapolation of YMDD/YIDD in unknown specimens using the measured FAM/VIC ratio.

Linear regression analysis was also applied to baseline subtracted fluorescence ratio vs. predefined allele ratio. Excellent linear regression as shown in Fig. 3B was reached.

Traditionally, comparative ΔCt method for each allele frequency measure, which is based on the equation allele ratio = 2^-ΔCt ^shown in Supplementary Material 7, 8, 9 [see Additional file 7, 8, 9], the averaged 2^-ΔCt ^measurements of replicates were used to analyze linear regression to the mixed allele ratio. Ct value was determined by the second derivative maximum method [19]. Run 3 but run 1 or run 2 has good lineation in ΔCt method (Fig 3C). These results suggest run 1 and run 2 cannot conclude reliable result from the standard curve in ΔCt method. In contrast, good linearship in all standard curves in the two fluorescence ratio methods suggests reliable results can be obtained from the standard curve by these two methods.

### Intra-and inter-run variation in allele ratio quantification of the proposed method

It is assumed that the lower coefficient of variation (CV), the more precise quantification will yield. To compare precision and reproducibility of the proposed K_f _method with the other two data analysis, intra-run and inter-run variations of all three analysis methods were investigated shown in Supplementary Material 7, 8, 9 [see Additional file 7, 8, 9]. The within run and run-to-run precision were determined shown in Table 4. All but two CV values (ratio2: 8 in run 1 and ratio 4:6 in run 2) among total 27 values by K_f _method were significantly lower than or proximate to those of SDM Ct method. The results indicate the proposed K_f _holds more precise allele quantification capacity compared to traditional SDM method for each run. Over-time run inter CV values were also compared between K_f _method and SDM method. All the samples from allele ratio 9:1 to 1:9 have more precise result in K_f _method among over time runs. This results show that the repeatability of proposed K_f _method is higher than the SDM method, which is also confirmed by the result that three stand curves almost converge to one linear curve while three standard curves of SDM method diverge from each other markedly (Fig. 3). Both intra run and inter run CV values by baseline subtracted fluorescence did not show significant lower values than those of SDM data point analysis method. From this we conclude that baseline subtracted fluorescence ratio method did not have a significant advantage of more precision over the method of SDM data points analysis method. However, according to table 4 row 3 and row 11, allele ratio 9:1 and 1:9 two samples in three different runs, SDM method in allele ratio 9:1 sample resulted intra CV 37.98% in run 2, 47.29% run 3. In contrast, baseline subtracted fluorescence ratio method brought intra CV in run 2 and run 3 respectively 4.91% and 11.95%, Normalized fluorescence ratio (K_f_) respectively 4.90% and 11.80%. The fluorescence ratio methods, both baseline subtracted fluorescence ratio and K_f _method, have much lower CV in this allele ratio sample. Allele ratio 1:9 has 113.3% CV in run 3 when SDM method was applied, while only 5.98% and 10.24% respective in fluorescence ratio and K_f _method. In SDM method, three values (37.98%, 47.29% 113.3%) coming from two allele ratio samples (9:1 and 1:9) in three runs diverge significantly from average (18.54% run 2, 21.34% run 3) of these samples. Precision decreased markedly in these two samples by the SDM method. The result suggest that precise allele ratio quantification rang in the two fluorescence ratio method is wider than SDM method. Detailed intra and inter CV values were provided in Supplement Material 10, 11, 12 [see Additional file 10, 11, 12].

## Discussion

Because of its conceptual simplicity and practical easy use, real time PCR is widely used to detect and quantify DNA and c DNA in diverse applications such as diagnosis and genetics. The quantification of single nucleotide polymorphism (SNP) allele frequencies in pooled DNA samples using real time PCR is a promising approach for large-scale diagnostics and genotyping. Currently real-time PCR to determine the allele ratio in pooled DNA mainly depends on Ct value. But inconsistency in PCR conditions, position effect, different florescence background etc all can lead to Ct value variability. And the small standard deviation of Ct values is amplified in the exponential way. For example, a Ct difference of one represents two-fold difference [20]. The CV values for the variation of quantification seem to be too large between replicates and over-time run.

To avoid using Ct values in quantification allele frequency, a novel rational data processing method, which is based on the normalized fluorescence ratio K_f _of real-time PCR exponential phase, was presented. K_f _method extends the work of Dwight H. Oliver, and James R. Eshleman [21]. The logic in proposed method derives from parallelism between fluorescence and produced amplicon [22].

Correction of background vertical shift of the amplification curve is the prerequisite for the correct quantification. By background correction, precision was reported improved [23]. However this data analysis did not ideal fully resolve the non-PCR related fluorescence fluctuations occurring well to well (Fig 1B) and overtime run. For this reason, Passive reference dye (such as ROX) was applied to in the experiment setup to deal with problem. In the data procession step after PCR, We probe to investigate the possibility of decreasing difference within replicates due to non-PCR related fluorescence fluctuations by data point analysis. Matthew P (2004) and Liu.W.etc (2002) pointed that background fluorescence can be obtained by the developed four-parametric sigmoid function fit-point method [18]. Utilizing the determined background fluorescence, we process the raw fluorescence data to obtain normalized fluorescence by first subtracting each data point with individual background fluorescence and then dividing the each subtracted fluorescence by the background fluorescence. Thus all fluorescence curves are displayed as fluorescence increment relative to individual initial background fluorescence, similar to PCR curve when passive reference dye was added in the reaction, if initial background were considered to be comparable to passive reference dye. This is not a too much aggressive postulation, if we get down to the fact that in the early PCR reactions, there is no PCR related fluorescence but non-PCR fluorescence difference due to instrument, reaction mixes or tubes. The applicability of the proposed method is attested by its ability to improve the homogeneity of intra-run replicates amplification curves. Thus this analysis step can remove well-to-well variations better than baseline-subtracted method to some degree.

Dwight H. Oliver early novel work in quantifying allele ratio by end point fluorescence ratio instead of the Ct value. However, amplification data points could be easily influenced by cycle-to-cycle signal noise and furthermore the end point fluorescence cannot serve as idea factor for real time PCR quantification. Thus the method in which as many as possible fluorescence data points from early exponential phase used in Kf method could potentially gives more reliable measurement for quantification of allele ratio than the method where only one data point, such as end fluorescence, used to be analyzed for interpretation.Equ.6 revealed that the fluorescence ratio between the FAM and VIC for each cycle is not only proportional to the initial allele ratio but also depend on the probe binding efficiency (k1) and allele amplification efficiency (K2). In Stahlberg, A. etc. [15]early work aiming at quantifying allele ratio, the efficiency ratio X_er _is determined from an intrinsic calibration curve by diluting the test sample. And relative sensitivity K_RS _reflecting the difference in the probes' fluorescence and binding efficiencies are further determined from the measurements on negative samples assuming a 60:40 expression ratio. However even slight PCR amplification efficiency inaccuracy, which is practically more than often confronted due to the fact that amplification efficiencies are estimates by all current method, may distort the results significantly because of exponential nature expressed Equ.6 [24]. Rather than separately calculate the K1 (K_RS_) and K2 (X_er_), the proposed method here determine combined effects of these two factors from the slope of the standard curve. The slopes of the standard curves in overtime runs (0.3160, 0.3201, and 0.2929) show that these combined effects are relatively constant among different runs in the data point analysis K_f _method suggested here.

Major improvement in precision of allele ratio determination in K_f _method was manifest from the fact that CV was always significantly reduced in the proposed k_f _method when compared intra and inter CV value with that of baseline subtracted fluorescence ratio method and SDM Ct method. Our results also suggest that precise allele ratio quantification range in the both baseline subtracted fluorescence ratio method and normalization fluorescence ratio method (k_f _method) was wider than that in SDM method. In addition, two out of the three standard curves (r^2 ^= 0.7966, r^2 ^= 0.8181, r^2 ^= 0.9961) in the SDM Ct method did not have good linear relationship. So, the allele ratio quantification cannot be reliably obtained in these two standard curves while K_f _has exceptionally good linear relationship (r^2 ^> 0.99) with allele ratio in all three runs. In SDM method, the larger difference between two the allele ratios, the larger difference of calculated result of SDM method deviate from linear orbit. This can be explained that slight Ct value determination error was multiplied (E^-ΔCt^) to such an extension that deviation of extreme ratio sample (9:1 or 1:9) was larger in the SDM method assuming the accurate amplification efficiency values were obtained in this method.

Non-PCR related fluorescence might have an influence on the fluorescence reading of each data point. So non-PCR related fluorescence has an effect on the determination of fluorescence ratio of each reaction. This hypothesis was confirmed from result of the initial fluorescence background analysis. Run 3 average FAM fluorescence background (371) was higher than the other two runs FAM fluorescence (187 and 163). But the VIC fluorescence backgrounds (101, 92,134) did not vary greatly in all three runs. So, in the baseline subtracted fluorescence ratio method, the non-PCR related fluorescence ratio of run 3 (2.76) was larger than that of other two runs (1.85, 1.77), which partly explains that the slope of the standard curve for run 3 (0.9199) is bigger than those of other two runs (0.5548, 0.5312). By normalizing each data points of initial fluorescence background, as proposed in fluorescence normalization method (k_f_) method, initial fluorescence background effect was decreased largely. In a parallel experiment, the method was also used to analyze YMDD/YVDD allele mixed samples with above proposed workflow (data not shown). Results both from YMDD/YIDD and from YMDD/YVDD showed that the homogeneity of standard curves in K_f _method was better than in baseline subtracted fluorescence ratio method.

Although our analysis method is tested to quantify single nucleotide polymorphism (SNP) ratio in pooled DNA, we also anticipate that this analysis method could be used in determining the relative expression of two genes in one sample. Anders Stahlberg (2003) etc. reported the real-time method application in the latter area, the method, however, is only to tell weather or not the expression of two genes is ~60:40. The resulted shown here suggested that the proposed method here hold the potential of wider use in the two gene relative expression determination.

## Conclusion

In conclusion, provided raw fluorescent data for independent fluorophore channels can be obtained, the proposed K_f _method to quantify the allele is more precise and can give repeatable results between different PCR runs. It also has an advantage of wider precise quantification range. Additional benefit is that the k_f _method K_f _method, without using any manually set parameters in the analysis process, is adaptable to fully automatic allele ratio quantification

## Methods

### Real-time PCR

Using the primer and probe design software Primer Express™, we designed PCR primer and MGB-probes for amplification of hepatitis B virus (HBV) fragment to detect rtM204I mutation in reverse transcriptase (rt) region of the HBV polymerase gene. In brief, PCR amplification reactions contained 400 nmol forward primer: 5'- GTAGGGCTTTCCCCCACTG -3'and reverse primer: 5'- AGM GGT AAA AAG GGA YTC AMG ATG -3', 200 nmol wild type HBV specific probe: 5'FAM – ATCATCCATATAACTGAAA-MGB-3', 200 nmol mutant HBV specific probe: 5'VIC – CACATCATCAATATAAC -MGB -3', 200 μM each dATP, dCTP and dGTP, 400 μM dUTP, 2 U Amplitaq Gold DNA polymerase, 0.2 U AmpErase Uracil N-glycosidase (UNG), Self-mixed Taqman buffer in a total volume of 50 μL. After a decontamination step at 37°C for 5 min and inactivation of UNG enzyme at 95°C for 10 min, a two step protocol was followed for 40 cycles: 95°C for 20 s and 60°C for 40 s. The real-time PCR was performed in Biorad iCycler. Raw fluorescence readings of each cycle from reactions were exported to an Excel workbook for further analysis.

### Different allele ratio DNA pools preparation

The concentration of the DNAs used to construct pools was measured using the Roche Diagnostics HBV Monitor assay. All samples were measured in duplicates. Range pools were constructed by mixing appropriate volumes of homozygote DNA. The concentrations ranged from 10%–90% to 90%-10% with 10% increments.

### Data analysis

Fluorescence data from real-time PCR were exported to a MS Excel spreadsheet. Raw fluorescence data and normalized fluorescence data .vs cycles were fitted with the four parametric sigmoid function (equation 11) using nonlinear regression function of SigmaPlot. FAM fluorescence vs. VIC fluorescence was fit with linear regression function of SigmaPlot. The slopes of the regression curves were used as normalized fluorescence ratio for quantification. Standard curves were constructed from samples containing known amounts of the given targets.

## Authors' contributions

All authors contribute to the main body of the project.

HG verify statistical methods and mathematical calculation

AY conceived of the study and took out the project

## Supplementary Material

Additional file 1 Contained the raw and analytical datas used during the procession. contain the ratios of normalized fluorescence for all samples. Click here for file

Additional file 2 Contained the raw and analytical datas used during the procession. contain the ratios of normalized fluorescence for all samples. Click here for file

Additional file 3 Contained the raw and analytical datas used during the procession. contain the ratios of normalized fluorescence for all samples. Click here for file

Additional file 4 Contained the raw and analytical datas used during the procession. provide baseline-subtracted fluorescence ratios. Click here for file

Additional file 5 Contained the raw and analytical datas used during the procession. provide baseline-subtracted fluorescence ratios. Click here for file

Additional file 6 Contained the raw and analytical datas used during the procession. provide baseline-subtracted fluorescence ratios. Click here for file

Additional file 7 Contained the raw and analytical datas used during the procession. provide comparative ΔCt method for each allele frequency measurement. Click here for file

Additional file 8 Contained the raw and analytical datas used during the procession. provide comparative ΔCt method for each allele frequency measurement. Click here for file

Additional file 9 Contained the raw and analytical datas used during the procession. provide comparative ΔCt method for each allele frequency measurement. Click here for file

Additional file 10 Contained the raw and analytical datas used during the procession. provide detailed intra and inter CV values of three compared methods. Click here for file

Additional file 11 Contained the raw and analytical datas used during the procession. provide detailed intra and inter CV values of three compared methods. Click here for file

Additional file 12 Contained the raw and analytical datas used during the procession. provide detailed intra and inter CV values of three compared methods. Click here for file

## Figures and Tables

**Table 1:** An example of four-parameter sigmoidal equation modeling and result of fluorescence normalization

Cycle Numberx^a^	D2 sample FAM (Raw Rx)^b^	D2 Normalized Rx . (Raw Rx -/Raw Y_0_-1)^c^
23.58	353.48	0.056045583
24.58	355.38	0.061721963
**25.58**^f^	**367.68**	**0.098469051**
**26.58**	**380.74**	**0.13748669**
**27.58**	**409.2**	**0.222512879**
**28.56**	**429.79**	**0.284026907**
**29.61**	**465.57**	**0.390922095**
**30.56**	**504.15**	**0.506182473**
**31.63**	**535.18**	**0.598886713**
**32.63**	**576.31**	**0.72176539**
**33.63**	**595.56**	**0.779276076**
34.63	639.21	0.909683425
35.63	669.3	0.99957935
Parameter	Sigmoid function fitted for raw fluorescence^d^	Sigmoid function fitted for normalized fluorescence^e^
a	525.5884	1.5702
b	3.2674	3.2674
X0	33.4481	33.4481
Y0	334.7204	0
R	0.998805	0.99880514

^a ^amplification cycle number

^b ^Raw FAM fluorescence data

^c^Normalized fluorescence = Raw Rx/Raw y_o_-1

^d ^4 parameter sigmoid function fitted for raw fluorescence

^e ^4 parameter sigmoid function fitted for normalized fluorescence

^f ^bold text cycles are selected PCR exponential phase for allele ratio quantification

**Table 2:** Fluorescence ratio K_f _determination by linear regression

Num.	R_0_		K_f_		
			
	Value.	SD	Value	SD	R^2^
Rep.1	0.0234	0.01	0.4111	0.0147	0.996175
Rep.2	0.0171	0.0119	0.4132	0.0157	0.995673
Rep.3	0.0132	0.0081	0.3595	0.0095	0.997902
Rep.4	-0.0161	0.0146	0.426	0.0184	0.994446
K_f _Av.			0.40245^a^	0.014575^b^	
K_f _CV	7.30%^c^				

^a^Average K_f _value of four replicates

^b^Standard Deviation of K_f _value for four replicates

^c^K_f _coefficient of variation

**Table 3:** Summary of the fluorescence ratio based method and the Ct base method for quantification of predefined standards

Group No. In predefined standards	Predefined allele ratio YMDD:	K_f _method			Baseline subtracted fluorescence ratio			SDM		
				
	YIDD	Mean (n = 4)^a^	S.D (n = 4)^b^	Intra C.V^c^	Mean (n = 4)^d^	S.D (n = 4)^e^	Intra C.V^c^	Mean (n = 4)^f^	S.D (n = 4)^g^	Intra C.V^c^
1	90%: 10%	2.68	0.32	11.80%	8.31	0.99	11.95%	6.46	3.06	47.29%
2	80%: 20%	1.40	0.08	5.45%	4.25	0.27	6.45%	2.73	0.09	3.30%
3	70%: 30%	0.80	0.14	17.22%	2.15	0.69	31.87%	1.46	0.14	9.32%
4	60%: 40%	0.54	0.03	6.11%	1.38	0.17	12.22%	1.09	0.27	24.43%
5	50%: 50%	0.40	0.03	7.30%	1.12	0.17	15.59%	0.65	0.07	10.38%
6	40%: 60%	0.30	0.02	7.77%	0.76	0.10	13.09%	0.51	0.04	8.56%
7	30%: 70%	0.22	0.00	1.91%	0.62	0.03	4.89%	0.32	0.08	25.12%
8	20%: 80%	0.15	0.01	3.67%	0.39	0.03	6.57%	0.43	0.18	42.29%
9	10%: 90%	0.07	0.01	10.24%	0.17	0.01	5.98%	0.01	0.02	113.30%
Av.	Average CV			7.94%			12.07%			31.56%

The spreadsheet used for this analysis is available as Supplementary Material.1 [see Additional file 1]

a. Arithmetic mean K_f _of 4 replicates

b. Combined Standard deviation K_f _for 4 replicates

c. (SD/mean) × 100%

d. Fluorescence ratio arithmetic mean of 4 replicates

e. Combined Standard deviation fluorescence ratio for 4 replicates

f. Arithmetic mean 2^-ΔCt ^of 4 replicates

g. Standard deviation of 2^-ΔCt ^for 4 replicates

**Table 4:** intra-run and inter-run variations of three methods for three different runs

**allele ratio**	K_f _method				Baseline subtracted fluorescence ratio method				SDM method (2^-ΔCt ^method)			
			
	Run 1	Run 2	Run 3	Combined	Run 1	Run 2	Run 3	Combined	Run 1	Run 2	Run 3	Combined
	intra C.V.	intra C.V.	intra C.V.	inter C.V.	intra C.V.	intra C.V.	intra C.V.	inter C.V.	intra C.V.	intra C.V.	intra C.V.	inter C.V.
90%:10%	4.38%	4.90%	11.80%	7.69%	9.39%	4.91%	11.95%	30.98%	13.81%	37.98%	47.29%	69.23%
80%:20%	9.18%	16.15%	5.45%	12.54%	5.30%	9.11%	6.45%	21.53%	26.72%	20.39%	3.30%	23.96%
70%:30%	5.40%	6.66%	17.22%	13.64%	18.48%	26.65%	31.87%	26.26%	11.90%	10.05%	9.32%	10.36%
60%:40%	2.99%	4.44%	6.11%	10.91%	4.41%	10.11%	12.22%	11.55%	3.52%	7.41%	24.43%	15.06%
50%:50%	6.06%	24.75%	7.30%	23.71%	10.59%	45.08%	15.59%	41.63%	22.12%	31.72%	10.38%	27.37%
40%:60%	28.91%	27.05%	7.77%	20.61%	33.55%	34.65%	13.09%	34.48%	38.09%	13.30%	8.56%	24.53%
30%:70%	4.70%	15.97%	1.91%	20.19%	7.38%	22.71%	4.89%	41.05%	7.61%	11.17%	25.12%	26.06%
20%:80%	29.02%	18.71%	3.67%	17.82%	36.42%	10.93%	6.57%	30.18%	13.72%	13.23%	42.29%	28.82%
10%:90%	61.69%	22.86%	10.24%	44.25%	81.13%	21.84%	5.98%	44.52%	98.39%	21.62%	113.30%	103.45%

Further detailed K_f _values were provided as supplementary material 2, 3 for run1 [see Additional file 2, 3], supplementary material 4, 5 for run 2 [see Additional file 4, 5], supplementary material 6,7 for run 3 [see Additional file 6, 7].
