# Supplementary material for: Quantify single nucleotide polymorphism (SNP) ratio in pooled DNA based on normalized fluorescence real-time PCR
Source: BMC Genomics. 2006 Jun 9;7:143. doi: 10.1186/1471-2164-7-143 (PMC1552069; doi:10.1186/1471-2164-7-143)
Supplement: Additional file 1 — Contained the raw and analytical datas used during the procession. contain the ratios of normalized fluorescence for all samples. [file 1471-2164-7-143-S1.pdf]

| Well/ | Raw FAM fluorescence data | | | Well/ | | | |
|---|---|---|---|---|---|---|---|
| Cycle | B5 | B6 | B7 | Cycle | B5 | B6 | B7 |
| 0.56 | 175.35 | 180.60 | 173.85 | 0.56 | -0.0663 | -0.03 | -0.06 |
| 1.56 | 186.13 | 186.22 | 180.08 | 1.56 | -0.0089 | 0.00 | -0.03 |
| 2.56 | 187.56 | 190.18 | 185.71 | 2.56 | -0.0013 | 0.02 | 0.00 |
| 3.56 | 184.88 | 186.85 | 181.40 | 3.56 | -0.0155 | 0.00 | -0.02 |
| 4.56 | 191.94 | 190.14 | 186.08 | 4.56 | 0.0221 | 0.02 | 0.00 |
| 5.56 | 186.34 | 187.58 | 185.36 | 5.56 | -0.0078 | 0.01 | 0.00 |
| 6.56 | 188.66 | 191.61 | 187.29 | 6.56 | 0.0046 | 0.03 | 0.01 |
| 7.56 | 187.28 | 186.22 | 186.24 | 7.56 | -0.0028 | 0.00 | 0.00 |
| 8.56 | 192.91 | 186.47 | 190.18 | 8.56 | 0.0272 | 0.00 | 0.03 |
| 9.56 | 189.91 | 189.45 | 187.32 | 9.56 | 0.0112 | 0.02 | 0.01 |
| 10.56 | 190.95 | 191.08 | 189.73 | 10.56 | 0.0168 | 0.02 | 0.02 |
| 11.56 | 189.71 | 189.16 | 187.50 | 11.56 | 0.0102 | 0.01 | 0.01 |
| 12.56 | 191.17 | 192.45 | 193.81 | 12.56 | 0.0180 | 0.03 | 0.04 |
| 13.56 | 190.86 | 188.22 | 190.48 | 13.56 | 0.0163 | 0.01 | 0.03 |
| 14.56 | 191.46 | 194.32 | 190.30 | 14.56 | 0.0195 | 0.04 | 0.03 |
| 15.56 | 192.13 | 188.08 | 187.35 | 15.56 | 0.0231 | 0.01 | 0.01 |
| 16.58 | 192.58 | 191.17 | 189.92 | 16.58 | 0.0255 | 0.03 | 0.02 |
| 17.58 | 192.24 | 185.61 | 187.46 | 17.58 | 0.0237 | 0.00 | 0.01 |
| 18.58 | 190.43 | 188.37 | 190.08 | 18.58 | 0.0140 | 0.01 | 0.02 |
| 19.58 | 192.38 | 189.18 | 190.19 | 19.58 | 0.0244 | 0.01 | 0.03 |
| 20.58 | 190.19 | 184.75 | 187.74 | 20.58 | 0.0127 | -0.01 | 0.01 |
| 21.58 | 192.96 | 183.79 | 190.94 | 21.58 | 0.0275 | -0.01 | 0.03 |
| 22.58 | 191.31 | 184.67 | 191.38 | 22.58 | 0.0187 | -0.01 | 0.03 |
| 23.58 | 192.78 | 188.14 | 194.26 | 23.58 | 0.0265 | 0.01 | 0.05 |
| 24.58 | 194.63 | 188.54 | 194.48 | 24.58 | 0.0364 | 0.01 | 0.05 |
| 25.58 | 198.16 | 194.05 | 201.15 | 25.58 | 0.0552 | 0.04 | 0.08 |
| 26.58 | 206.89 | 200.06 | 210.05 | 26.58 | 0.1017 | 0.07 | 0.13 |
| 27.58 | 225.50 | 216.55 | 226.63 | 27.58 | 0.2008 | 0.16 | 0.22 |
| 28.56 | 240.02 | 233.56 | 244.14 | 28.56 | 0.2781 | 0.25 | 0.32 |
| 29.61 | 272.06 | 271.06 | 280.02 | 29.61 | 0.4487 | 0.45 | 0.51 |
| 30.56 | 295.19 | 299.67 | 304.69 | 30.56 | 0.5719 | 0.61 | 0.64 |
| 31.63 | 327.37 | 332.06 | 325.81 | 31.63 | 0.7432 | 0.78 | 0.76 |
| 32.63 | 359.68 | 367.07 | 362.49 | 32.63 | 0.9153 | 0.97 | 0.95 |
| 33.63 | 384.63 | 398.38 | 382.73 | 33.63 | 1.0481 | 1.14 | 1.06 |
| 34.63 | 404.42 | 420.96 | 404.12 | 34.63 | 1.1535 | 1.26 | 1.18 |
| 35.63 | 431.85 | 455.22 | 431.12 | 35.63 | 1.2996 | 1.44 | 1.32 |
| 36.63 | 451.32 | 473.21 | 451.02 | 36.63 | 1.4032 | 1.54 | 1.43 |
| 37.65 | 469.37 | 488.35 | 467.86 | 37.65 | 1.4993 | 1.62 | 1.52 |
| 38.65 | 493.16 | 509.69 | 489.31 | 38.65 | 1.6260 | 1.73 | 1.64 |
| 39.65 | 498.01 | 521.14 | 496.33 | 39.65 | 1.6518 | 1.80 | 1.68 |

| Parameter | | | |
|---|---|---|---|
| a | 323.048500 | 338.957900 | 325.604200 |
| b | 2.526600 | 2.281900 | 2.665600 |
| x0 | 32.518000 | 32.439300 | 32.404000 |
| y0 | 187.797500 | 186.422300 | 185.512400 |
| R | 0.998757 | 0.998715 | 0.998665 |

| | | | |
|---|---|---|---|
| **$CP_{(SDM)}$** | 29.19 | 29.43 | 28.89 |

| Well/ Cycle | Raw VIC fluorescence data B5 | B6 | B7 |
|---|---|---|---|
| 0.56 | 92.95 | 97.65 | 97.6 |
| 1.56 | 97.24 | 101.12 | 101.99 |
| 2.56 | 97.71 | 103.17 | 101.79 |
| 3.56 | 98.71 | 101.57 | 101.62 |
| 4.56 | 97.55 | 101.38 | 102.18 |
| 5.56 | 98.49 | 102.53 | 103.39 |
| 6.56 | 98.8 | 100.15 | 102.05 |
| 7.56 | 99.6 | 99.82 | 103.59 |
| 8.56 | 98.61 | 99.66 | 104.1 |
| 9.56 | 99.42 | 99.93 | 103.62 |
| 10.56 | 100.68 | 100.81 | 104.25 |
| 11.56 | 99.49 | 99.17 | 103.68 |
| 12.56 | 99.89 | 100.26 | 104.5 |
| 13.56 | 99.73 | 100.76 | 104.06 |
| 14.56 | 99.5 | 98.82 | 105.05 |
| 15.56 | 99.2 | 99.06 | 104.49 |
| 16.58 | 99.33 | 98.58 | 103.5 |
| 17.58 | 99.96 | 99.13 | 104.89 |
| 18.58 | 101.17 | 99.84 | 103.82 |
| 19.58 | 99.64 | 99.2 | 104.21 |
| 20.58 | 100.03 | 97.65 | 105.53 |
| 21.58 | 100.4 | 97.26 | 105.96 |
| 22.58 | 101.21 | 100.39 | 106.41 |
| 23.58 | 101.19 | 98.24 | 106.67 |
| 24.58 | 103.7 | 99.71 | 109.34 |
| 25.58 | 103.5 | 100.68 | 111.74 |
| 26.58 | 105.61 | 103.68 | 114.93 |
| 27.58 | 106.8 | 105.84 | 115.82 |
| 28.56 | 111.1 | 110.75 | 118.24 |
| 29.61 | 114.98 | 114.16 | 123.38 |
| 30.56 | 120.16 | 122.61 | 129.99 |
| 31.63 | 126.22 | 127.79 | 135.88 |
| 32.63 | 131.25 | 136.1 | 142.91 |
| 33.63 | 139.87 | 147.99 | 151.32 |
| 34.63 | 146.33 | 156.39 | 157.15 |
| 35.63 | 150.24 | 166.56 | 162.4 |
| 36.63 | 159.25 | 170.52 | 173.37 |
| 37.65 | 161.62 | 180.73 | 175.1 |
| 38.65 | 171.22 | 189.99 | 186.44 |
| 39.65 | 178.67 | 200.37 | 192.47 |
| Parameter | | | |
| a | 98.34 | 112.28 | 115.39 |
| b | 3.454 | 2.7339 | 3.7881 |
| x0 | 35.039 | 34.636 | 35.123 |
| y0 | 98.537 | 99.389 | 102.75 |
| R | 0.9979 | 0.9977 | 0.9985 |
| $CP_{(SDM)}$ | 30.49 | 31.04 | 30.13 |

| Well/ Cycle | Fluorescence Data Normalized To Y0 B5 | B6 | B7 |
|---|---|---|---|
| 0.56 | -0.06 | -0.02 | -0.05 |
| 1.56 | -0.01 | 0.02 | -0.01 |
| 2.56 | -0.01 | 0.04 | -0.01 |
| 3.56 | 0.00 | 0.02 | -0.01 |
| 4.56 | -0.01 | 0.02 | -0.01 |
| 5.56 | 0.00 | 0.03 | 0.01 |
| 6.56 | 0.00 | 0.01 | -0.01 |
| 7.56 | 0.01 | 0.00 | 0.01 |
| 8.56 | 0.00 | 0.00 | 0.01 |
| 9.56 | 0.01 | 0.01 | 0.01 |
| 10.56 | 0.02 | 0.01 | 0.01 |
| 11.56 | 0.01 | 0.00 | 0.01 |
| 12.56 | 0.01 | 0.01 | 0.02 |
| 13.56 | 0.01 | 0.01 | 0.01 |
| 14.56 | 0.01 | -0.01 | 0.02 |
| 15.56 | 0.01 | 0.00 | 0.02 |
| 16.58 | 0.01 | -0.01 | 0.01 |
| 17.58 | 0.01 | 0.00 | 0.02 |
| 18.58 | 0.03 | 0.00 | 0.01 |
| 19.58 | 0.01 | 0.00 | 0.01 |
| 20.58 | 0.02 | -0.02 | 0.03 |
| 21.58 | 0.02 | -0.02 | 0.03 |
| 22.58 | 0.03 | 0.01 | 0.04 |
| 23.58 | 0.03 | -0.01 | 0.04 |
| 24.58 | 0.05 | 0.00 | 0.06 |
| 25.58 | 0.05 | 0.01 | 0.09 |
| 26.58 | 0.07 | 0.04 | 0.12 |
| 27.58 | 0.08 | 0.06 | 0.13 |
| 28.56 | 0.13 | 0.11 | 0.15 |
| 29.61 | 0.17 | 0.15 | 0.20 |
| 30.56 | 0.22 | 0.23 | 0.27 |
| 31.63 | 0.28 | 0.29 | 0.32 |
| 32.63 | 0.33 | 0.37 | 0.39 |
| 33.63 | 0.42 | 0.49 | 0.47 |
| 34.63 | 0.49 | 0.57 | 0.53 |
| 35.63 | 0.52 | 0.68 | 0.58 |
| 36.63 | 0.62 | 0.72 | 0.69 |
| 37.65 | 0.64 | 0.82 | 0.70 |
| 38.65 | 0.74 | 0.91 | 0.81 |
| 39.65 | 0.81 | 1.02 | 0.87 |

| B5 | | | B6 | |
|---|---|---|---|---|
| Well/ Cycle | FAM Normalized Fluorescence | VIC Normalized Fluorescence | Well/ Cycle | FAM Normalized Fluorescence |
| 0.56 | -0.0662815 | -0.056695685 | 0.56 | -0.031231779 |
| 1.56 | -0.008879245 | -0.013158562 | 1.56 | -0.001085171 |
| 2.56 | -0.00126466 | -0.008388761 | 2.56 | 0.020156923 |
| 3.56 | -0.015535351 | 0.001759752 | 3.56 | 0.002294253 |
| 4.56 | 0.022058334 | -0.010012523 | 4.56 | 0.019942357 |
| 5.56 | -0.007761019 | -0.000472921 | 5.56 | 0.006210094 |
| 6.56 | 0.004592713 | 0.002673118 | 6.56 | 0.027827679 |
| 7.56 | -0.002755628 | 0.010791929 | 7.56 | -0.001085171 |
| 8.56 | 0.027223472 | 0.000744901 | 8.56 | 0.000255871 |
| 9.56 | 0.011248819 | 0.008965197 | 9.56 | 0.016241083 |
| 10.56 | 0.016786698 | 0.021752324 | 10.56 | 0.024984672 |
| 11.56 | 0.010183842 | 0.009675593 | 11.56 | 0.014685475 |
| 12.56 | 0.017958173 | 0.013734998 | 12.56 | 0.032333578 |
| 13.56 | 0.016307459 | 0.012111236 | 13.56 | 0.00964316 |
| 14.56 | 0.01950239 | 0.009777078 | 14.56 | 0.042364567 |
| 15.56 | 0.023070062 | 0.006732524 | 15.56 | 0.008892177 |
| 16.58 | 0.02546626 | 0.00805183 | 16.58 | 0.025467447 |
| 17.58 | 0.023655799 | 0.014445394 | 17.58 | -0.004357311 |
| 18.58 | 0.014017758 | 0.026725095 | 18.58 | 0.010447784 |
| 19.58 | 0.024401283 | 0.01119787 | 19.58 | 0.014792758 |
| 20.58 | 0.012739786 | 0.01515579 | 20.58 | -0.008970493 |
| 21.58 | 0.027489716 | 0.01891074 | 21.58 | -0.014120092 |
| 22.58 | 0.018703657 | 0.027131036 | 22.58 | -0.009399627 |
| 23.58 | 0.026531237 | 0.026928065 | 23.58 | 0.009214026 |
| 24.58 | 0.036382273 | 0.052400834 | 24.58 | 0.011359692 |
| 25.58 | 0.055179116 | 0.050371131 | 25.58 | 0.040916242 |
| 26.58 | 0.101665358 | 0.071784494 | 26.58 | 0.073154875 |
| 27.58 | 0.200761458 | 0.083861225 | 27.58 | 0.161609958 |
| 28.56 | 0.278078782 | 0.127499833 | 28.56 | 0.252854406 |
| 29.61 | 0.448688082 | 0.166876064 | 29.61 | 0.454010599 |
| 30.56 | 0.57185266 | 0.219445363 | 30.56 | 0.607479363 |
| 31.63 | 0.743207444 | 0.280945354 | 31.63 | 0.781224671 |
| 32.63 | 0.915254463 | 0.331992376 | 32.63 | 0.969024092 |
| 33.63 | 1.048110332 | 0.419472561 | 33.63 | 1.136976102 |
| 34.63 | 1.153489796 | 0.485031958 | 34.63 | 1.258098951 |
| 35.63 | 1.299551378 | 0.524712645 | 35.63 | 1.441875248 |
| 36.63 | 1.40322688 | 0.61615075 | 36.63 | 1.538376578 |
| 37.65 | 1.499341046 | 0.640202727 | 37.65 | 1.619590038 |
| 38.65 | 1.626020048 | 0.737628455 | 38.65 | 1.734061322 |
| 39.65 | 1.651845738 | 0.813234879 | 39.65 | 1.795481013 |

| Experiment derived parameter | linear regression value |
|---|---|
| y0 | -0.0824 |
| a | 2.9914 |
| R | 0.9965093 |

| Experiment derived parameter | linear regression value |
|---|---|
| y0 | -0.0227 |
| a | 2.7451 |
| R | 0.99416363 |

| | B7 | | |
|---|---|---|---|
| VIC Normalized Fluorescence | Well/ Cycle | **FAM** Normalized Fluorescence | VIC Normalized Fluorescence |
| -0.017498883 | 0.56 | -0.062865879 | -0.050080977 |
| 0.017414367 | 1.56 | -0.029283218 | -0.007354086 |
| 0.03804035 | 2.56 | 0.001065158 | -0.009300642 |
| 0.021942022 | 3.56 | -0.02216779 | -0.010955214 |
| 0.020030345 | 4.56 | 0.003059634 | -0.005504859 |
| 0.031601019 | 5.56 | -0.000821508 | 0.006271801 |
| 0.007654755 | 6.56 | 0.009582109 | -0.00677012 |
| 0.004334475 | 7.56 | 0.00392211 | 0.008218357 |
| 0.002724642 | 8.56 | 0.025160582 | 0.013182073 |
| 0.005441235 | 9.56 | 0.009743823 | 0.00851034 |
| 0.014295316 | 10.56 | 0.022734868 | 0.01464199 |
| -0.002205471 | 11.56 | 0.010714109 | 0.009094307 |
| 0.008761515 | 12.56 | 0.044728007 | 0.017075184 |
| 0.013792243 | 13.56 | 0.026777725 | 0.012792762 |
| -0.00572698 | 14.56 | 0.025807439 | 0.022428211 |
| -0.003312231 | 15.56 | 0.009905537 | 0.016977856 |
| -0.00814173 | 16.58 | 0.023759059 | 0.007342407 |
| -0.002607929 | 17.58 | 0.01049849 | 0.020870967 |
| 0.004535704 | 18.58 | 0.024621535 | 0.010456895 |
| -0.001903627 | 19.58 | 0.025214487 | 0.014252678 |
| -0.017498883 | 20.58 | 0.012007823 | 0.027099944 |
| -0.021422851 | 21.58 | 0.029257343 | 0.031285038 |
| 0.010069505 | 22.58 | 0.031629153 | 0.035664788 |
| -0.011562625 | 23.58 | 0.047153721 | 0.03819531 |
| 0.003227715 | 24.58 | 0.048339626 | 0.064181824 |
| 0.012987327 | 25.58 | 0.084294096 | 0.087540488 |
| 0.043171693 | 26.58 | 0.132269325 | 0.118588047 |
| 0.064904436 | 27.58 | 0.221643405 | 0.127250218 |
| 0.114306182 | 28.56 | 0.316030627 | 0.150803538 |
| 0.148615745 | 29.61 | 0.509440878 | 0.200830011 |
| 0.233635043 | 30.56 | 0.642423903 | 0.265163666 |
| 0.285753382 | 31.63 | 0.75627074 | 0.322489722 |
| 0.369364076 | 32.63 | 0.953993372 | 0.390911144 |
| 0.48899478 | 33.63 | 1.063096591 | 0.472763797 |
| 0.573511005 | 34.63 | 1.178398856 | 0.529505886 |
| 0.675836006 | 35.63 | 1.323941688 | 0.580602965 |
| 0.71567937 | 36.63 | 1.431212145 | 0.687371527 |
| 0.818406829 | 37.65 | 1.521987749 | 0.704209231 |
| 0.911575906 | 38.65 | 1.637613443 | 0.814578921 |
| 1.016013812 | 39.65 | 1.675454579 | 0.873267566 |

| **Experiment derived** parameter | linear regression value |
|---|---|
| y0 | -0.1329 |
| a | 2.8316 |
| **R** | **0.98832278** |

| | **value** |
|---|---|
| y0 | -0.079333333 |
| a | 2.856033333 |
| **R** | |

| Well/ Cycle | Raw fluorescence data B8 | B9 | Well/ Cycle | B8 | B9 |
|---|---|---|---|---|---|
| 0.56 | 192.52 | 226.77 | 0.56 | -0.0811 | -0.0283 |
| 1.56 | 201.40 | 236.94 | 1.56 | -0.0387 | 0.0153 |
| 2.56 | 202.97 | 236.37 | 2.56 | -0.0312 | 0.0129 |
| 3.56 | 202.55 | 238.99 | 3.56 | -0.0332 | 0.0241 |
| 4.56 | 206.76 | 241.88 | 4.56 | -0.0131 | 0.0365 |
| 5.56 | 210.21 | 239.59 | 5.56 | 0.0033 | 0.0267 |
| 6.56 | 209.36 | 238.83 | 6.56 | -0.0007 | 0.0234 |
| 7.56 | 208.01 | 238.12 | 7.56 | -0.0072 | 0.0204 |
| 8.56 | 214.45 | 234.18 | 8.56 | 0.0236 | 0.0035 |
| 9.56 | 214.87 | 237.53 | 9.56 | 0.0256 | 0.0178 |
| 10.56 | 214.02 | 234.69 | 10.56 | 0.0215 | 0.0057 |
| 11.56 | 212.60 | 238.05 | 11.56 | 0.0147 | 0.0201 |
| 12.56 | 213.25 | 239.62 | 12.56 | 0.0178 | 0.0268 |
| 13.56 | 213.10 | 232.51 | 13.56 | 0.0171 | -0.0037 |
| 14.56 | 218.23 | 233.14 | 14.56 | 0.0416 | -0.0010 |
| 15.56 | 214.07 | 233.78 | 15.56 | 0.0218 | 0.0018 |
| 16.58 | 217.73 | 231.92 | 16.58 | 0.0392 | -0.0062 |
| 17.58 | 215.12 | 234.39 | 17.58 | 0.0268 | 0.0044 |
| 18.58 | 214.26 | 236.59 | 18.58 | 0.0227 | 0.0138 |
| 19.58 | 219.16 | 232.69 | 19.58 | 0.0461 | -0.0029 |
| 20.58 | 215.24 | 229.64 | 20.58 | 0.0273 | -0.0160 |
| 21.58 | 217.57 | 238.35 | 21.58 | 0.0385 | 0.0214 |
| 22.58 | 217.44 | 231.52 | 22.58 | 0.0378 | -0.0079 |
| 23.58 | 219.93 | 234.26 | 23.58 | 0.0497 | 0.0038 |
| 24.58 | 219.93 | 235.98 | 24.58 | 0.0497 | 0.0112 |
| 25.58 | 231.93 | 240.88 | 25.58 | 0.1070 | 0.0322 |
| 26.58 | 238.78 | 252.50 | 26.58 | 0.1397 | 0.0820 |
| 27.58 | 258.25 | 276.13 | 27.58 | 0.2326 | 0.1833 |
| 28.56 | 275.96 | 289.03 | 28.56 | 0.3172 | 0.2385 |
| 29.61 | 310.76 | 329.50 | 29.61 | 0.4833 | 0.4120 |
| 30.56 | 340.33 | 363.99 | 30.56 | 0.6244 | 0.5597 |
| 31.63 | 373.77 | 391.88 | 31.63 | 0.7840 | 0.6793 |
| 32.63 | 414.14 | 433.32 | 32.63 | 0.9767 | 0.8568 |
| 33.63 | 440.15 | 463.43 | 33.63 | 1.1008 | 0.9859 |
| 34.63 | 464.60 | 487.82 | 34.63 | 1.2175 | 1.0904 |
| 35.63 | 492.46 | 525.44 | 35.63 | 1.3505 | 1.2516 |
| 36.63 | 517.37 | 545.21 | 36.63 | 1.4694 | 1.3363 |
| 37.65 | 535.88 | 564.28 | 37.65 | 1.5578 | 1.4180 |
| 38.65 | 552.88 | 593.83 | 38.65 | 1.6389 | 1.5446 |
| 39.65 | 570.73 | 602.25 | 39.65 | 1.7241 | 1.5807 |

| Parameter | | |
|---|---|---|
| a | 374.289400 | 379.342000 |
| b | 2.636600 | 2.430800 |
| x0 | 32.386600 | 32.543000 |
| y0 | 209.511300 | 233.364500 |
| R | 0.998774 | 0.998419 |

| | | |
|---|---|---|
| **CP$_{(SDM)}$** | 28.91 | 29.34 |

| Raw fluorescence data | | Well/ Cycle | B8 | B9 |
|---|---|---|---|---|
| 94.49 | 97.15 | 0.56 | -0.0742 | 0.00 |
| 98.04 | 98.65 | 1.56 | -0.0394 | 0.01 |
| 99.36 | 99.17 | 2.56 | -0.0265 | 0.02 |
| 98.49 | 100.63 | 3.56 | -0.0350 | 0.03 |
| 101.41 | 99.11 | 4.56 | -0.0064 | 0.02 |
| 101.47 | 97.04 | 5.56 | -0.0058 | -0.01 |
| 101.83 | 97.13 | 6.56 | -0.0023 | 0.00 |
| 102.55 | 100.17 | 7.56 | 0.0048 | 0.03 |
| 99.98 | 97.43 | 8.56 | -0.0204 | 0.00 |
| 102.47 | 98.47 | 9.56 | 0.0040 | 0.01 |
| 105.60 | 96.12 | 10.56 | 0.0347 | -0.02 |
| 103.56 | 98.44 | 11.56 | 0.0147 | 0.01 |
| 101.04 | 98.74 | 12.56 | -0.0100 | 0.01 |
| 102.55 | 99.53 | 13.56 | 0.0048 | 0.02 |
| 104.04 | 97.26 | 14.56 | 0.0194 | 0.00 |
| 106.71 | 97.93 | 15.56 | 0.0455 | 0.00 |
| 104.87 | 96.99 | 16.58 | 0.0275 | -0.01 |
| 104.69 | 98.08 | 17.58 | 0.0257 | 0.00 |
| 106.40 | 97.76 | 18.58 | 0.0425 | 0.00 |
| 105.53 | 97.12 | 19.58 | 0.0340 | 0.00 |
| 105.34 | 97.26 | 20.58 | 0.0321 | 0.00 |
| 106.11 | 97.37 | 21.58 | 0.0397 | 0.00 |
| 106.62 | 98.08 | 22.58 | 0.0447 | 0.00 |
| 108.08 | 100.68 | 23.58 | 0.0590 | 0.03 |
| 109.50 | 100.51 | 24.58 | 0.0729 | 0.03 |
| 109.49 | 99.27 | 25.58 | 0.0728 | 0.02 |
| 114.31 | 105.19 | 26.58 | 0.1200 | 0.08 |
| 116.38 | 109.71 | 27.58 | 0.1403 | 0.12 |
| 121.90 | 112.55 | 28.56 | 0.1944 | 0.15 |
| 128.70 | 121.43 | 29.61 | 0.2610 | 0.24 |
| 141.73 | 130.28 | 30.56 | 0.3887 | 0.33 |
| 154.37 | 144.65 | 31.63 | 0.5125 | 0.48 |
| 162.03 | 156.08 | 32.63 | 0.5876 | 0.60 |
| 182.94 | 168.04 | 33.63 | 0.7924 | 0.72 |
| 198.60 | 180.90 | 34.63 | 0.9459 | 0.85 |
| 204.65 | 192.52 | 35.63 | 1.0052 | 0.97 |
| 217.39 | 200.53 | 36.63 | 1.1300 | 1.05 |
| 226.12 | 208.22 | 37.65 | 1.2155 | 1.13 |
| 241.28 | 216.67 | 38.65 | 1.3641 | 1.22 |
| 252.76 | 229.71 | 39.65 | 1.4765 | 1.35 |
| | | | | |
| 171.446600 | 137.567800 | | | |
| 3.004700 | 2.556800 | | | |
| 34.271600 | 33.557400 | | | |
| 102.062000 | 97.606300 | | | |
| 0.997992 | 0.998855 | | | |
| | | | | |
| 30.31 | 30.19 | | | |

| B8 | | | B9 | |
|---|---|---|---|---|
| Well/ Cycle | FAM Normalized Fluorescence | VIC Normalized Fluorescence | Well/ Cycle | FAM Normalized Fluorescence |
| 0.56 | -0.081099683 | -0.074190198 | 0.56 | -0.028258368 |
| 1.56 | -0.038715334 | -0.039407419 | 1.56 | 0.015321525 |
| 2.56 | -0.031221705 | -0.026474104 | 2.56 | 0.012878994 |
| 3.56 | -0.03322637 | -0.034998334 | 3.56 | 0.024106066 |
| 4.56 | -0.013131989 | -0.006388274 | 4.56 | 0.036490126 |
| 5.56 | 0.003334904 | -0.005800396 | 5.56 | 0.026677151 |
| 6.56 | -0.000722157 | -0.002273128 | 6.56 | 0.023420443 |
| 7.56 | -0.007165723 | 0.004781407 | 7.56 | 0.020377992 |
| 8.56 | 0.023572476 | -0.020399365 | 8.56 | 0.003494533 |
| 9.56 | 0.025577141 | 0.00399757 | 9.56 | 0.017849759 |
| 10.56 | 0.02152008 | 0.034665204 | 10.56 | 0.005679956 |
| 11.56 | 0.014742403 | 0.014677353 | 11.56 | 0.020078032 |
| 12.56 | 0.017844861 | -0.010013521 | 12.56 | 0.026805705 |
| 13.56 | 0.017128909 | 0.004781407 | 13.56 | -0.003661654 |
| 14.56 | 0.041614462 | 0.019380377 | 14.56 | -0.000962014 |
| 15.56 | 0.021758731 | 0.045540946 | 15.56 | 0.001780476 |
| 16.58 | 0.039227956 | 0.027512688 | 16.58 | -0.006189887 |
| 17.58 | 0.026770394 | 0.025749054 | 17.58 | 0.004394413 |
| 18.58 | 0.022665603 | 0.042503576 | 18.58 | 0.013821725 |
| 19.58 | 0.046053363 | 0.033979346 | 19.58 | -0.002890328 |
| 20.58 | 0.027343155 | 0.032117732 | 20.58 | -0.015960011 |
| 21.58 | 0.038464274 | 0.039662166 | 21.58 | 0.021363575 |
| 22.58 | 0.037843782 | 0.044659129 | 22.58 | -0.007903944 |
| 23.58 | 0.049728583 | 0.058964159 | 23.58 | 0.003837345 |
| 24.58 | 0.049728583 | 0.072877271 | 24.58 | 0.011207789 |
| 25.58 | 0.10700473 | 0.072779291 | 25.58 | 0.032204984 |
| 26.58 | 0.139699863 | 0.120005487 | 26.58 | 0.081998333 |
| 27.58 | 0.232630412 | 0.140287276 | 27.58 | 0.183256236 |
| 28.56 | 0.317160459 | 0.194372048 | 28.56 | 0.238534567 |
| 29.61 | 0.483261285 | 0.260998217 | 29.61 | 0.41195426 |
| 30.56 | 0.624399257 | 0.388665713 | 30.56 | 0.559748805 |
| 31.63 | 0.784008786 | 0.512512003 | 31.63 | 0.679261413 |
| 32.63 | 0.97669529 | 0.587564422 | 32.63 | 0.856837694 |
| 33.63 | 1.100841339 | 0.792439889 | 33.63 | 0.985863317 |
| 34.63 | 1.217541488 | 0.945876036 | 34.63 | 1.090377928 |
| 35.63 | 1.350517609 | 1.00515373 | 35.63 | 1.251584967 |
| 36.63 | 1.469413344 | 1.129979816 | 36.63 | 1.336302222 |
| 37.65 | 1.557761801 | 1.215516059 | 37.65 | 1.418019879 |
| 38.65 | 1.638903009 | 1.364053223 | 38.65 | 1.544645822 |
| 39.65 | 1.724101278 | 1.476533872 | 39.65 | 1.580726717 |

| Experiment derived parameter | linear regression value |
|---|---|
| y0 | -0.0072 |
| a | 1.6368 |
| R | 0.99155602 |

| Experiment derived parameter | linear regression value |
|---|---|
| y0 | 0.0165 |
| a | 1.4373 |
| R | 0.99011384 |

| VIC Normalized Fluorescence |
|---|
| -0.004674903 |
| 0.010692957 |
| 0.016020482 |
| 0.030978533 |
| 0.015405768 |
| -0.00580188 |
| -0.004879808 |
| 0.026265723 |
| -0.001806236 |
| 0.008848814 |
| -0.015227501 |
| 0.008541457 |
| 0.011615029 |
| 0.019708769 |
| -0.003547927 |
| 0.003316384 |
| -0.006314142 |
| 0.00485317 |
| 0.001574693 |
| -0.00498226 |
| -0.003547927 |
| -0.00242095 |
| 0.00485317 |
| 0.031490795 |
| 0.029749104 |
| 0.017045006 |
| 0.077696829 |
| 0.124005315 |
| 0.153101798 |
| 0.244079532 |
| 0.334749909 |
| 0.481974012 |
| 0.599077109 |
| 0.721610183 |
| 0.853363973 |
| 0.972413666 |
| 1.054478041 |
| 1.133263939 |
| 1.21983622 |
| 1.353434153 |

| Well/ Cycle | FAM Raw fluorescence data C3 | C4 | C5 | Well/ Cycle | FAM normalizednced data C3 | C4 | C5 |
|---|---|---|---|---|---|---|---|
| 0.56 | 188.66 | 229.61 | 180.33 | 0.56 | -0.0082 | -0.01 | 0.00 |
| 1.56 | 194.03 | 240.28 | 184.15 | 1.56 | 0.0201 | 0.04 | 0.02 |
| 2.56 | 194.61 | 242.62 | 182.68 | 2.56 | 0.0231 | 0.05 | 0.02 |
| 3.56 | 194.92 | 232.87 | 184.34 | 3.56 | 0.0247 | 0.01 | 0.02 |
| 4.56 | 194.47 | 239.80 | 185.23 | 4.56 | 0.0224 | 0.04 | 0.03 |
| 5.56 | 192.55 | 235.07 | 180.96 | 5.56 | 0.0123 | 0.02 | 0.01 |
| 6.56 | 195.38 | 240.03 | 182.43 | 6.56 | 0.0272 | 0.04 | 0.01 |
| 7.56 | 191.59 | 233.81 | 181.32 | 7.56 | 0.0072 | 0.01 | 0.01 |
| 8.56 | 195.21 | 236.83 | 182.33 | 8.56 | 0.0263 | 0.02 | 0.01 |
| 9.56 | 189.48 | 230.97 | 182.73 | 9.56 | -0.0039 | 0.00 | 0.02 |
| 10.56 | 194.24 | 234.30 | 182.62 | 10.56 | 0.0212 | 0.01 | 0.02 |
| 11.56 | 193.40 | 230.60 | 180.34 | 11.56 | 0.0168 | 0.00 | 0.00 |
| 12.56 | 193.76 | 234.01 | 180.16 | 12.56 | 0.0186 | 0.01 | 0.00 |
| 13.56 | 190.86 | 229.66 | 180.60 | 13.56 | 0.0034 | -0.01 | 0.00 |
| 14.56 | 192.29 | 237.00 | 179.83 | 14.56 | 0.0109 | 0.02 | 0.00 |
| 15.56 | 189.47 | 229.44 | 177.97 | 15.56 | -0.0039 | -0.01 | -0.01 |
| 16.58 | 190.06 | 232.84 | 182.33 | 16.58 | -0.0008 | 0.01 | 0.01 |
| 17.58 | 191.52 | 228.29 | 180.10 | 17.58 | 0.0069 | -0.01 | 0.00 |
| 18.58 | 191.81 | 233.85 | 178.83 | 18.58 | 0.0084 | 0.01 | -0.01 |
| 19.58 | 190.43 | 233.44 | 182.08 | 19.58 | 0.0011 | 0.01 | 0.01 |
| 20.58 | 189.18 | 229.71 | 178.59 | 20.58 | -0.0054 | -0.01 | -0.01 |
| 21.58 | 187.15 | 229.53 | 182.02 | 21.58 | -0.0161 | -0.01 | 0.01 |
| 22.58 | 186.54 | 225.00 | 178.05 | 22.58 | -0.0193 | -0.03 | -0.01 |
| 23.58 | 189.86 | 229.16 | 181.16 | 23.58 | -0.0019 | -0.01 | 0.01 |
| 24.58 | 190.06 | 234.47 | 182.19 | 24.58 | -0.0008 | 0.01 | 0.01 |
| 25.58 | 196.03 | 236.36 | 188.92 | 25.58 | 0.0306 | 0.02 | 0.05 |
| 26.58 | 200.89 | 245.59 | 192.98 | 26.58 | 0.0561 | 0.06 | 0.07 |
| 27.58 | 217.73 | 266.11 | 207.88 | 27.58 | 0.1447 | 0.15 | 0.16 |
| 28.56 | 229.23 | 283.05 | 219.41 | 28.56 | 0.2051 | 0.22 | 0.22 |
| 29.61 | 261.89 | 323.35 | 248.24 | 29.61 | 0.3768 | 0.40 | 0.38 |
| 30.56 | 282.80 | 351.97 | 266.54 | 30.56 | 0.4868 | 0.52 | 0.48 |
| 31.63 | 314.66 | 390.50 | 295.02 | 31.63 | 0.6543 | 0.69 | 0.64 |
| 32.63 | 347.40 | 432.37 | 322.47 | 32.63 | 0.8264 | 0.87 | 0.79 |
| 33.63 | 374.15 | 468.59 | 345.64 | 33.63 | 0.9670 | 1.03 | 0.92 |
| 34.63 | 394.25 | 498.12 | 362.63 | 34.63 | 1.0727 | 1.15 | 1.02 |
| 35.63 | 423.61 | 532.15 | 394.75 | 35.63 | 1.2270 | 1.30 | 1.19 |
| 36.63 | 444.72 | 558.06 | 415.45 | 36.63 | 1.3380 | 1.41 | 1.31 |
| 37.65 | 461.87 | 575.74 | 425.91 | 37.65 | 1.4282 | 1.49 | 1.37 |
| 38.65 | 478.71 | 606.85 | 445.78 | 38.65 | 1.5167 | 1.62 | 1.48 |
| 39.65 | 492.47 | 622.65 | 458.37 | 39.65 | 1.5891 | 1.69 | 1.55 |

| Parameter | | | |
|---|---|---|---|
| a | 308.202100 | 397.860300 | 289.089600 |
| b | 2.330400 | 2.336600 | 2.488000 |
| x0 | 32.735700 | 32.746400 | 32.882900 |
| y0 | 190.212600 | 231.369400 | 179.871100 |
| R | 0.998614 | 0.998401 | 0.998797 |
| **$CP_{(SDM)}$** | 29.67 | 29.67 | 29.61 |

| Well/ Cycle | Raw fluorescence data C3 | C4 | C5 | Well/ Cycle | C3 | C4 | C5 |
|---|---|---|---|---|---|---|---|
| 0.56 | 97.80 | 97.81 | 94.94 | 0.56 | 0.0070 | 0.02 | 0.02 |
| 1.56 | 97.86 | 98.25 | 95.20 | 1.56 | 0.0077 | 0.02 | 0.03 |
| 2.56 | 98.65 | 98.02 | 93.04 | 2.56 | 0.0158 | 0.02 | 0.00 |
| 3.56 | 99.56 | 99.33 | 95.78 | 3.56 | 0.0252 | 0.03 | 0.03 |
| 4.56 | 99.46 | 97.49 | 93.29 | 4.56 | 0.0241 | 0.01 | 0.01 |
| 5.56 | 100.66 | 98.93 | 95.57 | 5.56 | 0.0365 | 0.03 | 0.03 |
| 6.56 | 98.01 | 96.96 | 93.70 | 6.56 | 0.0092 | 0.01 | 0.01 |
| 7.56 | 98.11 | 98.12 | 94.49 | 7.56 | 0.0102 | 0.02 | 0.02 |
| 8.56 | 99.46 | 96.79 | 93.50 | 8.56 | 0.0241 | 0.00 | 0.01 |
| 9.56 | 98.76 | 98.24 | 93.28 | 9.56 | 0.0169 | 0.02 | 0.01 |
| 10.56 | 98.38 | 97.41 | 93.46 | 10.56 | 0.0130 | 0.01 | 0.01 |
| 11.56 | 98.54 | 98.20 | 93.00 | 11.56 | 0.0147 | 0.02 | 0.00 |
| 12.56 | 98.48 | 97.11 | 93.98 | 12.56 | 0.0140 | 0.01 | 0.01 |
| 13.56 | 98.12 | 97.68 | 92.93 | 13.56 | 0.0103 | 0.01 | 0.00 |
| 14.56 | 96.51 | 96.73 | 93.80 | 14.56 | -0.0062 | 0.00 | 0.01 |
| 15.56 | 97.79 | 96.83 | 92.85 | 15.56 | 0.0069 | 0.00 | 0.00 |
| 16.58 | 98.59 | 96.05 | 92.20 | 16.58 | 0.0152 | 0.00 | -0.01 |
| 17.58 | 97.45 | 96.39 | 92.91 | 17.58 | 0.0034 | 0.00 | 0.00 |
| 18.58 | 96.64 | 95.52 | 91.89 | 18.58 | -0.0049 | -0.01 | -0.01 |
| 19.58 | 96.50 | 96.28 | 92.89 | 19.58 | -0.0063 | 0.00 | 0.00 |
| 20.58 | 96.22 | 95.52 | 93.22 | 20.58 | -0.0092 | -0.01 | 0.01 |
| 21.58 | 97.43 | 94.83 | 91.93 | 21.58 | 0.0032 | -0.02 | -0.01 |
| 22.58 | 96.97 | 96.22 | 93.51 | 22.58 | -0.0015 | 0.00 | 0.01 |
| 23.58 | 97.84 | 96.57 | 94.88 | 23.58 | 0.0075 | 0.00 | 0.02 |
| 24.58 | 97.21 | 98.57 | 95.00 | 24.58 | 0.0010 | 0.02 | 0.02 |
| 25.58 | 101.60 | 101.76 | 95.80 | 25.58 | 0.0462 | 0.06 | 0.03 |
| 26.58 | 105.03 | 103.70 | 101.89 | 26.58 | 0.0815 | 0.08 | 0.10 |
| 27.58 | 110.58 | 108.72 | 106.41 | 27.58 | 0.1386 | 0.13 | 0.15 |
| 28.56 | 119.65 | 118.35 | 115.00 | 28.56 | 0.2320 | 0.23 | 0.24 |
| 29.61 | 132.31 | 129.57 | 125.40 | 29.61 | 0.3624 | 0.34 | 0.35 |
| 30.56 | 146.60 | 144.83 | 139.91 | 30.56 | 0.5095 | 0.50 | 0.51 |
| 31.63 | 163.09 | 159.19 | 155.15 | 31.63 | 0.6793 | 0.65 | 0.67 |
| 32.63 | 181.18 | 175.01 | 167.44 | 32.63 | 0.8656 | 0.82 | 0.81 |
| 33.63 | 201.85 | 198.01 | 185.93 | 33.63 | 1.0785 | 1.06 | 1.00 |
| 34.63 | 219.89 | 215.19 | 200.07 | 34.63 | 1.2642 | 1.23 | 1.16 |
| 35.63 | 233.01 | 231.57 | 213.24 | 35.63 | 1.3993 | 1.40 | 1.30 |
| 36.63 | 251.24 | 244.09 | 226.05 | 36.63 | 1.5870 | 1.53 | 1.44 |
| 37.65 | 263.14 | 260.25 | 237.57 | 37.65 | 1.7096 | 1.70 | 1.56 |
| 38.65 | 279.57 | 275.89 | 249.47 | 38.65 | 1.8787 | 1.86 | 1.69 |
| 39.65 | 295.21 | 287.77 | 261.87 | 39.65 | 2.0398 | 1.99 | 1.82 |

| Parameter | | | |
|---|---|---|---|
| a | 210.248100 | 205.600400 | 178.946800 |
| b | 2.568800 | 2.553100 | 2.610700 |
| x0 | 33.811100 | 33.862000 | 33.539300 |
| y0 | 97.115600 | 96.355100 | 92.735700 |
| R | 0.998859 | 0.999099 | 0.998971 |

| | | | |
|---|---|---|---|
| $CP_{(SDM)}$ | 30.43 | 30.50 | 30.10 |

| Well/ Cycle | FAM Normalized Fluorescence | VIC Normalized Fluorescence | Well/ Cycle | FAM Normalized Fluorescence |
|---|---|---|---|---|
| 0.56 | -0.008162446 | 0.007047271 | 0.56 | -0.00760429 |
| 1.56 | 0.020069123 | 0.007665092 | 1.56 | 0.038512439 |
| 2.56 | 0.023118342 | 0.015799727 | 2.56 | 0.048626136 |
| 3.56 | 0.024748098 | 0.025170004 | 3.56 | 0.006485732 |
| 4.56 | 0.022382324 | 0.024140303 | 4.56 | 0.036437835 |
| 5.56 | 0.012288355 | 0.036496711 | 5.56 | 0.015994336 |
| 6.56 | 0.027166444 | 0.009209643 | 6.56 | 0.037431916 |
| 7.56 | 0.007241371 | 0.010239344 | 7.56 | 0.010548499 |
| 8.56 | 0.026272707 | 0.024140303 | 8.56 | 0.02360122 |
| 9.56 | -0.00385148 | 0.016932398 | 9.56 | -0.001726244 |
| 10.56 | 0.02117315 | 0.013019535 | 10.56 | 0.012666325 |
| 11.56 | 0.016757039 | 0.014667057 | 11.56 | -0.003325418 |
| 12.56 | 0.018649658 | 0.014049236 | 12.56 | 0.011412918 |
| 13.56 | 0.00340356 | 0.010342314 | 13.56 | -0.007388185 |
| 14.56 | 0.010921464 | -0.006235867 | 14.56 | 0.024335975 |
| 15.56 | -0.003904053 | 0.006944301 | 15.56 | -0.008339046 |
| 16.58 | -0.00080226 | 0.015181907 | 16.58 | 0.00635607 |
| 17.58 | 0.006873362 | 0.003443319 | 17.58 | -0.013309452 |
| 18.58 | 0.008397972 | -0.004897256 | 18.58 | 0.010721383 |
| 19.58 | 0.001142932 | -0.006338837 | 19.58 | 0.008949325 |
| 20.58 | -0.005428662 | -0.009221999 | 20.58 | -0.007172081 |
| 21.58 | -0.016100931 | 0.003237379 | 21.58 | -0.007950057 |
| 22.58 | -0.019307869 | -0.001499244 | 22.58 | -0.027529137 |
| 23.58 | -0.001853715 | 0.007459152 | 23.58 | -0.009549232 |
| 24.58 | -0.00080226 | 0.000972037 | 24.58 | 0.013401081 |
| 25.58 | 0.030583673 | 0.046175898 | 25.58 | 0.021569836 |
| 26.58 | 0.056134031 | 0.081494631 | 26.58 | 0.061462752 |
| 27.58 | 0.144666547 | 0.138643019 | 27.58 | 0.150152094 |
| 28.56 | 0.205125213 | 0.232036872 | 28.56 | 0.223368345 |
| 29.61 | 0.376827823 | 0.362396978 | 29.61 | 0.397548682 |
| 30.56 | 0.486757449 | 0.509541207 | 30.56 | 0.521246976 |
| 31.63 | 0.65425424 | 0.67933885 | 31.63 | 0.687777208 |
| 32.63 | 0.826377432 | 0.865611704 | 32.63 | 0.868743231 |
| 33.63 | 0.967009546 | 1.078450836 | 33.63 | 1.025289429 |
| 34.63 | 1.072680779 | 1.26420884 | 34.63 | 1.152920827 |
| 35.63 | 1.227034382 | 1.39930557 | 35.63 | 1.300001642 |
| 36.63 | 1.338015463 | 1.587020005 | 36.63 | 1.411987065 |
| 37.65 | 1.428177734 | 1.709554387 | 37.65 | 1.488401664 |
| 38.65 | 1.516710249 | 1.87873421 | 38.65 | 1.622861969 |
| 39.65 | 1.589050357 | 2.039779397 | 39.65 | 1.691151034 |

| Experiment derived parameter | linear regression value |
|---|---|
| y0 | -0.0039 |
| a | 0.9681 |
| R | 0.9981421 |

| Experiment derived parameter | linear regression value |
|---|---|
| y0 | -0.0032 |
| a | 1.0669 |
| R | 0.9979948 |

| VIC Normalized Fluorescence | Well/ Cycle | **FAM** Normalized Fluorescence | VIC Normalized Fluorescence |
|---|---|---|---|
| 0.015099356 | 0.56 | 0.002551271 | 0.023769702 |
| 0.019665799 | 1.56 | 0.023788702 | 0.026573369 |
| 0.017278795 | 2.56 | 0.015616183 | 0.003281368 |
| 0.030874339 | 3.56 | 0.024845014 | 0.032827703 |
| 0.011778308 | 4.56 | 0.029793002 | 0.005977202 |
| 0.026723028 | 5.56 | 0.00605378 | 0.030563203 |
| 0.00627782 | 6.56 | 0.014226299 | 0.010398369 |
| 0.018316623 | 7.56 | 0.008055213 | 0.018917202 |
| 0.004513513 | 8.56 | 0.013670345 | 0.008241702 |
| 0.019562016 | 9.56 | 0.01589416 | 0.005869369 |
| 0.010948045 | 10.56 | 0.015282611 | 0.007810369 |
| 0.019146885 | 11.56 | 0.002606867 | 0.002850035 |
| 0.007834562 | 12.56 | 0.00160615 | 0.013417702 |
| 0.01375018 | 13.56 | 0.004052346 | 0.002095202 |
| 0.003890816 | 14.56 | -0.000228497 | 0.011476702 |
| 0.004928644 | 15.56 | -0.010569235 | 0.001232535 |
| -0.003166413 | 16.58 | 0.013670345 | -0.005776632 |
| 0.000362202 | 17.58 | 0.001272578 | 0.001879535 |
| -0.0086669 | 18.58 | -0.005788034 | -0.009119465 |
| -0.000779409 | 19.58 | 0.012280461 | 0.001663868 |
| -0.0086669 | 20.58 | -0.007122323 | 0.005222369 |
| -0.015827912 | 21.58 | 0.011946889 | -0.008688132 |
| -0.001402105 | 22.58 | -0.010124472 | 0.008349535 |
| 0.002230292 | 23.58 | 0.007165687 | 0.023122702 |
| 0.022986848 | 24.58 | 0.01289201 | 0.024416703 |
| 0.056093554 | 25.58 | 0.050307693 | 0.033043369 |
| 0.076227413 | 26.58 | 0.072879412 | 0.098713872 |
| 0.128326368 | 27.58 | 0.15571651 | 0.14745454 |
| 0.228269183 | 28.56 | 0.21981797 | 0.240083377 |
| 0.344713461 | 29.61 | 0.380099416 | 0.352230047 |
| 0.503085981 | 30.56 | 0.481838939 | 0.508696219 |
| 0.652118051 | 31.63 | 0.640174547 | 0.673034225 |
| 0.816302406 | 32.63 | 0.792783832 | 0.805561397 |
| 1.055002797 | 33.63 | 0.9215983 | 1.004945237 |
| 1.23330161 | 34.63 | 1.01605483 | 1.157421576 |
| 1.403297802 | 35.63 | 1.194627152 | 1.29943808 |
| 1.53323384 | 36.63 | 1.309709564 | 1.437572585 |
| 1.70094681 | 37.65 | 1.367862319 | 1.56179659 |
| 1.863263076 | 38.65 | 1.478330315 | 1.690118261 |
| 1.986557017 | 39.65 | 1.548324884 | 1.823831599 |

| **Experiment derived parameter** | linear regression **value** |
|---|---|
| y0 | -0.0032 |
| a | 0.9788 |
| **R** | **0.99673657** |

| | **value** |
|---|---|
| y0 | -0.003433333 |
| a | 1.0046 |
| **R** | |

| Well/ Cycle | Raw fluorescence data C6 | C7 | C8 | Well/ Cycle | C6 | C7 | C8 |
|---|---|---|---|---|---|---|---|
| 0.56 | 195.24 | 178.61 | 176.25 | 0.56 | -0.0243 | 0.00 | -0.05 |
| 1.56 | 202.70 | 177.93 | 183.59 | 1.56 | 0.0130 | -0.01 | -0.01 |
| 2.56 | 203.48 | 180.25 | 185.00 | 2.56 | 0.0169 | 0.01 | 0.00 |
| 3.56 | 202.04 | 180.36 | 186.45 | 3.56 | 0.0097 | 0.01 | 0.00 |
| 4.56 | 204.65 | 181.86 | 188.53 | 4.56 | 0.0227 | 0.02 | 0.02 |
| 5.56 | 200.69 | 179.03 | 184.46 | 5.56 | 0.0029 | 0.00 | -0.01 |
| 6.56 | 206.03 | 180.79 | 188.17 | 6.56 | 0.0296 | 0.01 | 0.01 |
| 7.56 | 199.66 | 180.13 | 185.71 | 7.56 | -0.0022 | 0.01 | 0.00 |
| 8.56 | 202.69 | 181.49 | 188.65 | 8.56 | 0.0129 | 0.01 | 0.02 |
| 9.56 | 198.90 | 179.77 | 186.93 | 9.56 | -0.0060 | 0.00 | 0.01 |
| 10.56 | 201.52 | 180.11 | 186.83 | 10.56 | 0.0071 | 0.01 | 0.01 |
| 11.56 | 200.89 | 180.44 | 188.09 | 11.56 | 0.0039 | 0.01 | 0.01 |
| 12.56 | 201.73 | 181.69 | 187.99 | 12.56 | 0.0081 | 0.02 | 0.01 |
| 13.56 | 200.43 | 180.55 | 190.80 | 13.56 | 0.0016 | 0.01 | 0.03 |
| 14.56 | 207.71 | 183.60 | 191.38 | 14.56 | 0.0380 | 0.03 | 0.03 |
| 15.56 | 202.05 | 177.13 | 191.42 | 15.56 | 0.0097 | -0.01 | 0.03 |
| 16.58 | 202.10 | 179.24 | 184.26 | 16.58 | 0.0100 | 0.00 | -0.01 |
| 17.58 | 199.42 | 179.56 | 187.11 | 17.58 | -0.0034 | 0.00 | 0.01 |
| 18.58 | 202.81 | 182.31 | 188.77 | 18.58 | 0.0135 | 0.02 | 0.02 |
| 19.58 | 202.92 | 179.86 | 187.45 | 19.58 | 0.0141 | 0.01 | 0.01 |
| 20.58 | 202.00 | 178.21 | 190.75 | 20.58 | 0.0095 | 0.00 | 0.03 |
| 21.58 | 201.56 | 180.43 | 190.96 | 21.58 | 0.0073 | 0.01 | 0.03 |
| 22.58 | 197.18 | 178.34 | 183.75 | 22.58 | -0.0146 | 0.00 | -0.01 |
| 23.58 | 204.39 | 183.65 | 191.23 | 23.58 | 0.0214 | 0.03 | 0.03 |
| 24.58 | 203.00 | 181.06 | 192.46 | 24.58 | 0.0145 | 0.01 | 0.04 |
| 25.58 | 204.54 | 185.96 | 198.61 | 25.58 | 0.0222 | 0.04 | 0.07 |
| 26.58 | 209.11 | 190.23 | 201.09 | 26.58 | 0.0450 | 0.06 | 0.08 |
| 27.58 | 223.59 | 199.88 | 215.12 | 27.58 | 0.1174 | 0.12 | 0.16 |
| 28.56 | 239.93 | 214.97 | 229.33 | 28.56 | 0.1990 | 0.20 | 0.23 |
| 29.61 | 264.32 | 241.66 | 254.63 | 29.61 | 0.3209 | 0.35 | 0.37 |
| 30.56 | 286.65 | 253.69 | 271.57 | 30.56 | 0.4325 | 0.42 | 0.46 |
| 31.63 | 316.82 | 279.23 | 294.40 | 31.63 | 0.5833 | 0.56 | 0.59 |
| 32.63 | 343.88 | 305.68 | 325.80 | 32.63 | 0.7185 | 0.71 | 0.75 |
| 33.63 | 366.08 | 329.35 | 348.29 | 33.63 | 0.8295 | 0.84 | 0.88 |
| 34.63 | 387.38 | 344.72 | 363.82 | 34.63 | 0.9359 | 0.93 | 0.96 |
| 35.63 | 412.66 | 370.16 | 390.65 | 35.63 | 1.0622 | 1.07 | 1.10 |
| 36.63 | 432.11 | 388.83 | 405.40 | 36.63 | 1.1594 | 1.17 | 1.18 |
| 37.65 | 451.58 | 402.22 | 422.03 | 37.65 | 1.2567 | 1.25 | 1.27 |
| 38.65 | 473.27 | 417.73 | 443.54 | 38.65 | 1.3651 | 1.34 | 1.39 |
| 39.65 | 483.59 | 428.36 | 455.61 | 39.65 | 1.4167 | 1.39 | 1.45 |
| Parameter | | | | | | | |
| a | 294.535000 | 259.619100 | 283.897400 | | | | |
| b | 2.474300 | 2.466800 | 2.665300 | | | | |
| x0 | 32.993100 | 32.912200 | 32.968100 | | | | |
| y0 | 200.102700 | 178.895000 | 185.708400 | | | | |
| R | 0.998399 | 0.998981 | 0.998692 | | | | |

| | | | |
|---|---|---|---|
| **$CP_{(SDM)}$** | 29.73 | 29.66 | 29.46 |

| Well/ Cycle | Raw fluorescence data | | | Well/ Cycle | | | |
|---|---|---|---|---|---|---|---|
| | C6 | C7 | C8 | | C6 | C7 | C8 |
| 0.56 | 100.85 | 98.29 | 94.51 | 0.56 | 0.0005 | -0.02 | -0.03 |
| 1.56 | 102.18 | 103.51 | 97.75 | 1.56 | 0.0137 | 0.03 | 0.00 |
| 2.56 | 102.18 | 102.65 | 95.08 | 2.56 | 0.0137 | 0.03 | -0.03 |
| 3.56 | 102.45 | 101.03 | 97.44 | 3.56 | 0.0164 | 0.01 | 0.00 |
| 4.56 | 102.51 | 99.97 | 97.40 | 4.56 | 0.0170 | 0.00 | 0.00 |
| 5.56 | 101.12 | 103.49 | 98.65 | 5.56 | 0.0032 | 0.03 | 0.01 |
| 6.56 | 101.46 | 101.30 | 97.22 | 6.56 | 0.0066 | 0.01 | -0.01 |
| 7.56 | 102.54 | 102.06 | 98.62 | 7.56 | 0.0173 | 0.02 | 0.01 |
| 8.56 | 102.83 | 101.73 | 98.73 | 8.56 | 0.0202 | 0.02 | 0.01 |
| 9.56 | 103.13 | 101.54 | 97.79 | 9.56 | 0.0231 | 0.01 | 0.00 |
| 10.56 | 101.41 | 100.30 | 98.90 | 10.56 | 0.0061 | 0.00 | 0.01 |
| 11.56 | 101.88 | 100.23 | 98.98 | 11.56 | 0.0107 | 0.00 | 0.01 |
| 12.56 | 103.25 | 101.83 | 99.99 | 12.56 | 0.0243 | 0.02 | 0.02 |
| 13.56 | 101.74 | 102.49 | 99.64 | 13.56 | 0.0093 | 0.02 | 0.02 |
| 14.56 | 100.83 | 102.56 | 100.98 | 14.56 | 0.0003 | 0.02 | 0.03 |
| 15.56 | 101.63 | 102.13 | 99.35 | 15.56 | 0.0083 | 0.02 | 0.02 |
| 16.58 | 101.23 | 99.40 | 98.72 | 16.58 | 0.0043 | -0.01 | 0.01 |
| 17.58 | 102.23 | 100.48 | 100.41 | 17.58 | 0.0142 | 0.00 | 0.03 |
| 18.58 | 101.38 | 98.98 | 99.75 | 18.58 | 0.0058 | -0.01 | 0.02 |
| 19.58 | 99.74 | 100.42 | 99.54 | 19.58 | -0.0105 | 0.00 | 0.02 |
| 20.58 | 100.53 | 99.09 | 101.44 | 20.58 | -0.0027 | -0.01 | 0.04 |
| 21.58 | 101.14 | 101.27 | 102.17 | 21.58 | 0.0034 | 0.01 | 0.04 |
| 22.58 | 102.27 | 101.98 | 102.38 | 22.58 | 0.0146 | 0.02 | 0.05 |
| 23.58 | 101.74 | 101.53 | 102.10 | 23.58 | 0.0093 | 0.01 | 0.04 |
| 24.58 | 102.90 | 103.85 | 104.88 | 24.58 | 0.0209 | 0.04 | 0.07 |
| 25.58 | 108.12 | 106.67 | 106.48 | 25.58 | 0.0726 | 0.07 | 0.09 |
| 26.58 | 112.04 | 110.38 | 113.59 | 26.58 | 0.1115 | 0.10 | 0.16 |
| 27.58 | 116.94 | 118.82 | 121.09 | 27.58 | 0.1601 | 0.19 | 0.24 |
| 28.56 | 131.44 | 133.65 | 134.13 | 28.56 | 0.3040 | 0.34 | 0.37 |
| 29.61 | 146.07 | 144.85 | 146.44 | 29.61 | 0.4491 | 0.45 | 0.50 |
| 30.56 | 165.24 | 165.78 | 167.38 | 30.56 | 0.6393 | 0.66 | 0.71 |
| 31.63 | 186.02 | 185.11 | 189.20 | 31.63 | 0.8455 | 0.85 | 0.93 |
| 32.63 | 207.00 | 202.82 | 208.92 | 32.63 | 1.0536 | 1.03 | 1.14 |
| 33.63 | 236.00 | 228.77 | 234.19 | 33.63 | 1.3413 | 1.29 | 1.39 |
| 34.63 | 256.30 | 246.20 | 254.95 | 34.63 | 1.5427 | 1.46 | 1.61 |
| 35.63 | 276.90 | 267.95 | 271.25 | 35.63 | 1.7471 | 1.68 | 1.77 |
| 36.63 | 296.02 | 284.87 | 290.27 | 36.63 | 1.9368 | 1.85 | 1.97 |
| 37.65 | 309.13 | 294.64 | 302.66 | 37.65 | 2.0668 | 1.94 | 2.09 |
| 38.65 | 326.22 | 313.81 | 315.67 | 38.65 | 2.2364 | 2.14 | 2.23 |
| 39.65 | 339.92 | 329.07 | 338.43 | 39.65 | 2.3723 | 2.29 | 2.46 |
| Parameter | | | | | | | |
| a | 251.023400 | 240.949100 | 252.469500 | | | | |
| b | 2.452600 | 2.579900 | 2.685300 | | | | |
| x0 | 33.390800 | 33.401600 | 33.332200 | | | | |
| y0 | 100.797900 | 100.085400 | 97.814400 | | | | |
| R | 0.999417 | 0.999046 | 0.999257 | | | | |

| | | | |
|---|---|---|---|
| $CP_{(SDM)}$ | 30.16 | 30.00 | 29.80 |

| C6 | | | C7 | |
|---|---|---|---|---|
| Well/ Cycle | **FAM** Normalized Fluorescence | VIC Normalized Fluorescence | Well/ Cycle | **FAM** Normalized Fluorescence |
| 0.56 | -0.024301021 | 0.000516876 | 0.56 | -0.001593113 |
| 1.56 | 0.012979835 | 0.013711595 | 1.56 | -0.005394226 |
| 2.56 | 0.016877833 | 0.013711595 | 2.56 | 0.007574275 |
| 3.56 | 0.009681529 | 0.016390222 | 3.56 | 0.008189161 |
| 4.56 | 0.022724831 | 0.016985473 | 4.56 | 0.016573968 |
| 5.56 | 0.002934993 | 0.003195503 | 5.56 | 0.000754633 |
| 6.56 | 0.029621289 | 0.006568589 | 6.56 | 0.010592806 |
| 7.56 | -0.002212364 | 0.017283098 | 7.56 | 0.006903491 |
| 8.56 | 0.012929861 | 0.020160142 | 8.56 | 0.014505716 |
| 9.56 | -0.006010414 | 0.023136395 | 9.56 | 0.004891137 |
| 10.56 | 0.007082863 | 0.006072547 | 10.56 | 0.006791693 |
| 11.56 | 0.00393448 | 0.010735343 | 11.56 | 0.008636351 |
| 12.56 | 0.008132324 | 0.024326896 | 12.56 | 0.01562369 |
| 13.56 | 0.00163566 | 0.009346425 | 13.56 | 0.009251237 |
| 14.56 | 0.038016978 | 0.000318459 | 14.56 | 0.026300344 |
| 15.56 | 0.009731503 | 0.008255132 | 15.56 | -0.009866123 |
| 16.58 | 0.009981375 | 0.004286796 | 16.58 | 0.001928506 |
| 17.58 | -0.003411748 | 0.014207637 | 17.58 | 0.003717264 |
| 18.58 | 0.013529553 | 0.005774922 | 18.58 | 0.01908941 |
| 19.58 | 0.01407927 | -0.010495258 | 19.58 | 0.005394226 |
| 20.58 | 0.009481631 | -0.002657793 | 20.58 | -0.003829062 |
| 21.58 | 0.00728276 | 0.00339392 | 21.58 | 0.008580452 |
| 22.58 | -0.014606 | 0.014604471 | 22.58 | -0.003102378 |
| 23.58 | 0.021425498 | 0.009346425 | 23.58 | 0.026579837 |
| 24.58 | 0.014479065 | 0.020854601 | 24.58 | 0.012102071 |
| 25.58 | 0.022175113 | 0.072641394 | 25.58 | 0.03949244 |
| 26.58 | 0.045013386 | 0.111531093 | 26.58 | 0.06336119 |
| 27.58 | 0.117376227 | 0.160143217 | 27.58 | 0.117303446 |
| 28.56 | 0.199034296 | 0.303995421 | 28.56 | 0.201654602 |
| 29.61 | 0.320921707 | 0.449137333 | 29.61 | 0.350848263 |
| 30.56 | 0.432514404 | 0.639319867 | 30.56 | 0.418094413 |
| 31.63 | 0.583286982 | 0.845474955 | 31.63 | 0.560859722 |
| 32.63 | 0.718517541 | 1.053614212 | 32.63 | 0.708711814 |
| 33.63 | 0.829460572 | 1.341318619 | 33.63 | 0.841024064 |
| 34.63 | 0.935905912 | 1.542711703 | 34.63 | 0.926940384 |
| 35.63 | 1.062241039 | 1.74708104 | 35.63 | 1.069146706 |
| 36.63 | 1.159441127 | 1.936767532 | 36.63 | 1.173509601 |
| 37.65 | 1.256741163 | 2.066829765 | 37.65 | 1.248357975 |
| 38.65 | 1.365135503 | 2.236376948 | 38.65 | 1.335056877 |
| 39.65 | 1.41670902 | 2.372292478 | 39.65 | 1.394477207 |

| **Experiment derived parameter** | linear regression **value** |
|---|---|
| y0 | -0.0101 |
| a | 0.6981 |
| **R** | **0.99843518** |

| **Experiment derived parameter** | linear regression **value** |
|---|---|
| y0 | -0.0069 |
| a | 0.6848 |
| **R** | **0.99393004** |

| | C8 | | |
|---|---|---|---|
| VIC Normalized Fluorescence | Well/ Cycle | FAM Normalized Fluorescence | VIC Normalized Fluorescence |
| -0.01793868 | 0.56 | -0.05093146 | -0.033782347 |
| 0.034216779 | 1.56 | -0.011407131 | -0.00065839 |
| 0.025624117 | 2.56 | -0.003814582 | -0.027954984 |
| 0.00943794 | 3.56 | 0.003993357 | -0.003827657 |
| -0.001153015 | 4.56 | 0.015193712 | -0.004236595 |
| 0.03401695 | 5.56 | -0.006722367 | 0.008542709 |
| 0.012135636 | 6.56 | 0.013255189 | -0.006076815 |
| 0.019729151 | 7.56 | 8.61566E-06 | 0.008236006 |
| 0.016431967 | 8.56 | 0.015839887 | 0.009360585 |
| 0.014533588 | 9.56 | 0.006578055 | -0.000249452 |
| 0.002144169 | 10.56 | 0.006039576 | 0.01109857 |
| 0.001444766 | 11.56 | 0.012824406 | 0.011916446 |
| 0.017431114 | 12.56 | 0.012285928 | 0.022242124 |
| 0.024025482 | 13.56 | 0.027417177 | 0.018663919 |
| 0.024724885 | 14.56 | 0.030540353 | 0.032363333 |
| 0.020428554 | 15.56 | 0.030755744 | 0.01569912 |
| -0.006848152 | 16.58 | -0.007799324 | 0.009258351 |
| 0.003942633 | 17.58 | 0.007547316 | 0.02653597 |
| -0.011044568 | 18.58 | 0.016486061 | 0.019788497 |
| 0.003343145 | 19.58 | 0.009378143 | 0.017641574 |
| -0.009945507 | 20.58 | 0.027147937 | 0.037066117 |
| 0.011835892 | 21.58 | 0.028278742 | 0.044529231 |
| 0.018929834 | 22.58 | -0.010545565 | 0.046676154 |
| 0.014433674 | 23.58 | 0.029732635 | 0.04381359 |
| 0.037613878 | 24.58 | 0.036355921 | 0.072234763 |
| 0.065789815 | 25.58 | 0.069472356 | 0.088592273 |
| 0.102858159 | 26.58 | 0.082826625 | 0.161280957 |
| 0.187186143 | 27.58 | 0.158375173 | 0.237956783 |
| 0.335359603 | 28.56 | 0.234892983 | 0.371270488 |
| 0.447264037 | 29.61 | 0.37112807 | 0.497121078 |
| 0.656385447 | 30.56 | 0.462346345 | 0.711199987 |
| 0.849520509 | 31.63 | 0.58528101 | 0.934275526 |
| 1.026469395 | 32.63 | 0.754363292 | 1.135881833 |
| 1.285747971 | 33.63 | 0.87546713 | 1.394228253 |
| 1.459899246 | 34.63 | 0.959092857 | 1.606466941 |
| 1.67721366 | 35.63 | 1.103566667 | 1.773109072 |
| 1.846269286 | 36.63 | 1.182992261 | 1.967558969 |
| 1.943885921 | 37.65 | 1.272541253 | 2.094227435 |
| 2.135422349 | 38.65 | 1.388368001 | 2.227234436 |
| 2.28789214 | 39.65 | 1.453362368 | 2.459920012 |

| Experiment derived parameter | linear regression value |
|---|---|
| y0 | -0.0022 |
| a | 0.6581 |
| **R** | **0.99496935** |

| average | value |
|---|---|
| y0 | -0.0064 |
| a | 0.680333333 |

| Raw fluorescence data | | | Well/ | | | |
|---|---|---|---|---|---|---|
| C9 | D2 | D3 | Cycle | C9 | D2 | D3 |
| 165.88 | 143.77 | 170.44 | 0.56 | -0.0748 | -0.01 | -0.03 |
| 172.57 | 146.07 | 174.16 | 1.56 | -0.0375 | 0.00 | -0.01 |
| 177.39 | 148.38 | 178.68 | 2.56 | -0.0106 | 0.02 | 0.01 |
| 177.97 | 146.70 | 177.71 | 3.56 | -0.0074 | 0.01 | 0.01 |
| 178.37 | 150.87 | 181.30 | 4.56 | -0.0051 | 0.04 | 0.03 |
| 177.80 | 145.62 | 177.18 | 5.56 | -0.0083 | 0.00 | 0.00 |
| 179.64 | 148.94 | 182.16 | 6.56 | 0.0019 | 0.02 | 0.03 |
| 178.39 | 146.77 | 177.45 | 7.56 | -0.0050 | 0.01 | 0.00 |
| 178.07 | 150.21 | 178.30 | 8.56 | -0.0068 | 0.03 | 0.01 |
| 182.65 | 146.21 | 177.07 | 9.56 | 0.0187 | 0.00 | 0.00 |
| 181.35 | 148.13 | 179.84 | 10.56 | 0.0115 | 0.02 | 0.02 |
| 181.06 | 148.15 | 178.59 | 11.56 | 0.0099 | 0.02 | 0.01 |
| 183.00 | 149.09 | 178.88 | 12.56 | 0.0207 | 0.02 | 0.01 |
| 183.04 | 145.52 | 177.41 | 13.56 | 0.0209 | 0.00 | 0.00 |
| 182.32 | 145.71 | 178.92 | 14.56 | 0.0169 | 0.00 | 0.01 |
| 183.56 | 142.95 | 176.01 | 15.56 | 0.0238 | -0.02 | 0.00 |
| 183.45 | 146.45 | 178.39 | 16.58 | 0.0232 | 0.01 | 0.01 |
| 183.85 | 145.38 | 176.52 | 17.58 | 0.0254 | 0.00 | 0.00 |
| 185.07 | 145.48 | 179.51 | 18.58 | 0.0322 | 0.00 | 0.02 |
| 182.56 | 146.86 | 178.33 | 19.58 | 0.0182 | 0.01 | 0.01 |
| 183.07 | 143.06 | 176.74 | 20.58 | 0.0211 | -0.02 | 0.00 |
| 185.20 | 144.52 | 178.88 | 21.58 | 0.0330 | -0.01 | 0.01 |
| 183.26 | 143.79 | 173.64 | 22.58 | 0.0221 | -0.01 | -0.02 |
| 187.78 | 143.55 | 176.89 | 23.58 | 0.0473 | -0.01 | 0.00 |
| 188.21 | 144.49 | 179.09 | 24.58 | 0.0497 | -0.01 | 0.01 |
| 192.76 | 148.60 | 181.28 | 25.58 | 0.0751 | 0.02 | 0.03 |
| 194.83 | 150.33 | 184.74 | 26.58 | 0.0867 | 0.03 | 0.05 |
| 200.61 | 158.32 | 195.55 | 27.58 | 0.1189 | 0.09 | 0.11 |
| 214.68 | 162.72 | 202.39 | 28.56 | 0.1974 | 0.12 | 0.15 |
| 229.46 | 180.41 | 223.52 | 29.61 | 0.2798 | 0.24 | 0.27 |
| 243.90 | 187.13 | 237.39 | 30.56 | 0.3604 | 0.28 | 0.34 |
| 262.27 | 200.86 | 257.96 | 31.63 | 0.4628 | 0.38 | 0.46 |
| 285.14 | 216.32 | 281.61 | 32.63 | 0.5904 | 0.49 | 0.59 |
| 300.02 | 234.66 | 303.53 | 33.63 | 0.6734 | 0.61 | 0.72 |
| 313.42 | 242.82 | 318.65 | 34.63 | 0.7481 | 0.67 | 0.80 |
| 334.28 | 259.74 | 343.00 | 35.63 | 0.8645 | 0.78 | 0.94 |
| 347.08 | 272.83 | 357.42 | 36.63 | 0.9358 | 0.87 | 1.02 |
| 359.01 | 283.84 | 371.84 | 37.65 | 1.0024 | 0.95 | 1.11 |
| 374.48 | 291.41 | 389.10 | 38.65 | 1.0887 | 1.00 | 1.20 |
| 386.55 | 298.58 | 400.64 | 39.65 | 1.1560 | 1.05 | 1.27 |
| | | | | | | |
| 224.713800 | 160.865100 | 235.319900 | | | | |
| 2.887800 | 2.405800 | 2.483800 | | | | |
| 33.286800 | 33.294300 | 33.357000 | | | | |
| 179.291400 | 145.646500 | 176.576100 | | | | |
| 0.998348 | 0.998185 | 0.998733 | | | | |
| | | | | | | |
| 29.48 | 30.13 | 30.09 | | | | |

| Well/ Cycle | Raw fluorescence data C9 | D2 | D3 | Well/ Cycle | C9 | D2 | D3 |
|---|---|---|---|---|---|---|---|
| 0.56 | 87.90 | 82.39 | 86.32 | 0.56 | -0.0379 | 0.00 | 0.01 |
| 1.56 | 88.06 | 84.00 | 87.36 | 1.56 | -0.0361 | 0.02 | 0.02 |
| 2.56 | 88.36 | 84.87 | 87.28 | 2.56 | -0.0328 | 0.03 | 0.02 |
| 3.56 | 90.37 | 84.05 | 88.47 | 3.56 | -0.0108 | 0.02 | 0.03 |
| 4.56 | 89.88 | 83.35 | 87.36 | 4.56 | -0.0162 | 0.01 | 0.02 |
| 5.56 | 91.06 | 83.24 | 88.58 | 5.56 | -0.0033 | 0.01 | 0.03 |
| 6.56 | 90.95 | 84.63 | 87.31 | 6.56 | -0.0045 | 0.03 | 0.02 |
| 7.56 | 90.47 | 85.48 | 87.12 | 7.56 | -0.0097 | 0.04 | 0.02 |
| 8.56 | 92.68 | 83.81 | 87.46 | 8.56 | 0.0145 | 0.02 | 0.02 |
| 9.56 | 91.77 | 84.29 | 85.79 | 9.56 | 0.0045 | 0.02 | 0.00 |
| 10.56 | 92.05 | 82.84 | 86.07 | 10.56 | 0.0076 | 0.01 | 0.00 |
| 11.56 | 92.26 | 82.66 | 87.37 | 11.56 | 0.0099 | 0.00 | 0.02 |
| 12.56 | 94.31 | 84.48 | 86.98 | 12.56 | 0.0323 | 0.03 | 0.01 |
| 13.56 | 92.09 | 82.66 | 86.59 | 13.56 | 0.0080 | 0.00 | 0.01 |
| 14.56 | 93.25 | 82.73 | 86.87 | 14.56 | 0.0207 | 0.00 | 0.01 |
| 15.56 | 94.42 | 82.40 | 86.15 | 15.56 | 0.0335 | 0.00 | 0.00 |
| 16.58 | 94.45 | 82.35 | 85.63 | 16.58 | 0.0338 | 0.00 | 0.00 |
| 17.58 | 95.40 | 82.50 | 85.48 | 17.58 | 0.0442 | 0.00 | 0.00 |
| 18.58 | 94.01 | 81.57 | 85.47 | 18.58 | 0.0290 | -0.01 | 0.00 |
| 19.58 | 94.74 | 82.00 | 85.94 | 19.58 | 0.0370 | 0.00 | 0.00 |
| 20.58 | 94.92 | 82.12 | 85.82 | 20.58 | 0.0390 | 0.00 | 0.00 |
| 21.58 | 95.55 | 83.19 | 86.36 | 21.58 | 0.0459 | 0.01 | 0.01 |
| 22.58 | 94.68 | 83.18 | 87.46 | 22.58 | 0.0364 | 0.01 | 0.02 |
| 23.58 | 97.96 | 85.09 | 86.52 | 23.58 | 0.0723 | 0.03 | 0.01 |
| 24.58 | 98.93 | 85.63 | 88.94 | 24.58 | 0.0829 | 0.04 | 0.04 |
| 25.58 | 102.63 | 87.00 | 90.82 | 25.58 | 0.1234 | 0.06 | 0.06 |
| 26.58 | 106.71 | 92.79 | 95.78 | 26.58 | 0.1680 | 0.13 | 0.12 |
| 27.58 | 113.63 | 99.79 | 102.11 | 27.58 | 0.2438 | 0.21 | 0.19 |
| 28.56 | 127.87 | 112.55 | 114.33 | 28.56 | 0.3996 | 0.37 | 0.33 |
| 29.61 | 140.12 | 123.07 | 128.45 | 29.61 | 0.5337 | 0.49 | 0.50 |
| 30.56 | 162.27 | 143.48 | 147.66 | 30.56 | 0.7762 | 0.74 | 0.72 |
| 31.63 | 181.45 | 158.62 | 169.57 | 31.63 | 0.9861 | 0.93 | 0.98 |
| 32.63 | 199.79 | 173.53 | 188.01 | 32.63 | 1.1869 | 1.11 | 1.19 |
| 33.63 | 222.37 | 193.40 | 214.51 | 33.63 | 1.4340 | 1.35 | 1.50 |
| 34.63 | 240.32 | 207.41 | 231.58 | 34.63 | 1.6305 | 1.52 | 1.70 |
| 35.63 | 256.38 | 219.09 | 245.19 | 35.63 | 1.8063 | 1.66 | 1.86 |
| 36.63 | 273.64 | 231.45 | 265.34 | 36.63 | 1.9952 | 1.81 | 2.09 |
| 37.65 | 285.20 | 245.77 | 280.17 | 37.65 | 2.1218 | 1.98 | 2.27 |
| 38.65 | 297.00 | 255.35 | 290.94 | 38.65 | 2.2509 | 2.10 | 2.39 |
| 39.65 | 311.22 | 263.40 | 303.33 | 39.65 | 2.4066 | 2.20 | 2.54 |

| Parameter | C9 | D2 | D3 |
|---|---|---|---|
| a | 231.752900 | 187.575500 | 225.555800 |
| b | 2.670100 | 2.505800 | 2.410400 |
| x0 | 33.015700 | 32.791800 | 33.119500 |
| y0 | 91.358900 | 82.358100 | 85.790100 |
| R | 0.999367 | 0.998961 | 0.999236 |
| **$CP_{(SDM)}$** | 29.50 | 29.49 | 29.95 |

| C9 | | | D2 | |
|---|---|---|---|---|
| Well/ Cycle | FAM Normalized Fluorescence | VIC Normalized Fluorescence | Well/ Cycle | FAM Normalized Fluorescence |
| 0.56 | -0.074802249 | -0.03786057 | 0.56 | -0.012883935 |
| 1.56 | -0.037488692 | -0.036109235 | 1.56 | 0.002907725 |
| 2.56 | -0.010605082 | -0.032825483 | 2.56 | 0.018768045 |
| 3.56 | -0.007370125 | -0.010824342 | 3.56 | 0.007233267 |
| 4.56 | -0.00513912 | -0.016187804 | 4.56 | 0.035864233 |
| 5.56 | -0.008318302 | -0.003271712 | 5.56 | -0.000181947 |
| 6.56 | 0.001944321 | -0.004475754 | 6.56 | 0.02261297 |
| 7.56 | -0.00502757 | -0.009729758 | 7.56 | 0.007713883 |
| 8.56 | -0.006812374 | 0.014460551 | 8.56 | 0.031332713 |
| 9.56 | 0.018732633 | 0.004499835 | 9.56 | 0.003868957 |
| 10.56 | 0.011481867 | 0.007564671 | 10.56 | 0.01705156 |
| 11.56 | 0.009864388 | 0.009863297 | 11.56 | 0.017188879 |
| 12.56 | 0.020684762 | 0.032302272 | 12.56 | 0.023642861 |
| 13.56 | 0.020907863 | 0.008002504 | 13.56 | -0.000868541 |
| 14.56 | 0.016892054 | 0.02069968 | 14.56 | 0.000435987 |
| 15.56 | 0.023808169 | 0.033506314 | 15.56 | -0.018514005 |
| 16.58 | 0.023194643 | 0.033834689 | 16.58 | 0.005516782 |
| 17.58 | 0.025425648 | 0.044233238 | 17.58 | -0.001829773 |
| 18.58 | 0.032230213 | 0.029018519 | 18.58 | -0.001143179 |
| 19.58 | 0.018230657 | 0.037008983 | 19.58 | 0.008331817 |
| 20.58 | 0.021075188 | 0.038979235 | 20.58 | -0.017758751 |
| 21.58 | 0.03295529 | 0.045875115 | 21.58 | -0.00773448 |
| 22.58 | 0.022134916 | 0.036352233 | 22.58 | -0.012746616 |
| 23.58 | 0.047345271 | 0.072254592 | 23.58 | -0.014394441 |
| 24.58 | 0.049743602 | 0.082872057 | 24.58 | -0.007940459 |
| 25.58 | 0.075121283 | 0.123371669 | 25.58 | 0.020278551 |
| 26.58 | 0.086666734 | 0.168030701 | 26.58 | 0.032156626 |
| 27.58 | 0.118904755 | 0.243775921 | 27.58 | 0.087015479 |
| 28.56 | 0.197380354 | 0.399644698 | 28.56 | 0.117225611 |
| 29.61 | 0.279815987 | 0.533731251 | 29.61 | 0.238684074 |
| 30.56 | 0.360355265 | 0.776181631 | 30.56 | 0.284823185 |
| 31.63 | 0.462814167 | 0.986122863 | 31.63 | 0.379092529 |
| 32.63 | 0.590371875 | 1.186869588 | 32.63 | 0.485239947 |
| 33.63 | 0.673365259 | 1.434026679 | 33.63 | 0.611161271 |
| 34.63 | 0.748103925 | 1.630504527 | 34.63 | 0.667187334 |
| 35.63 | 0.864450833 | 1.806294734 | 35.63 | 0.783359023 |
| 36.63 | 0.935842991 | 1.995219951 | 36.63 | 0.873234166 |
| 37.65 | 1.002382713 | 2.121753874 | 37.65 | 0.948828156 |
| 38.65 | 1.08866683 | 2.250914799 | 38.65 | 1.000803315 |
| 39.65 | 1.155987404 | 2.406564659 | 39.65 | 1.050032098 |

| Experiment derived parameter | linear regression value |
|---|---|
| y0 | 0.009 |
| a | 0.4753 |
| R | 0.99753434 |

| Experiment derived parameter | linear regression value |
|---|---|
| y0 | -0.014 |
| a | 0.4357 |
| R | 0.99141641 |

| | D3 | | |
|---|---|---|---|
| VIC Normalized Fluorescence | Well/ Cycle | FAM Normalized Fluorescence | VIC Normalized Fluorescence |
| 0.000387333 | 0.56 | -0.034750456 | 0.006176703 |
| 0.019936108 | 1.56 | -0.013683052 | 0.018299314 |
| 0.030499732 | 2.56 | 0.011914976 | 0.017366806 |
| 0.020543213 | 3.56 | 0.006421594 | 0.03123787 |
| 0.012043746 | 4.56 | 0.026752771 | 0.018299314 |
| 0.010708115 | 5.56 | 0.003420055 | 0.032520069 |
| 0.027585629 | 6.56 | 0.031623192 | 0.017716496 |
| 0.037906411 | 7.56 | 0.004949141 | 0.015501789 |
| 0.01762911 | 8.56 | 0.009762929 | 0.01946495 |
| 0.023457316 | 9.56 | 0.002797094 | -1.16564E-06 |
| 0.005851276 | 10.56 | 0.018484382 | 0.003262614 |
| 0.003665699 | 11.56 | 0.011405281 | 0.018415878 |
| 0.025764315 | 12.56 | 0.013047632 | 0.013869899 |
| 0.003665699 | 13.56 | 0.00472261 | 0.00932392 |
| 0.004515646 | 14.56 | 0.013274163 | 0.0125877 |
| 0.000508754 | 15.56 | -0.003205983 | 0.004195123 |
| -9.8351E-05 | 16.58 | 0.010272625 | -0.001866183 |
| 0.001722963 | 17.58 | -0.00031771 | -0.003614636 |
| -0.009569186 | 18.58 | 0.016615499 | -0.0037312 |
| -0.004348085 | 19.58 | 0.009932828 | 0.001747288 |
| -0.002891033 | 20.58 | 0.000928212 | 0.000348525 |
| 0.01010101 | 21.58 | 0.013047632 | 0.006642958 |
| 0.009979589 | 22.58 | -0.016627958 | 0.01946495 |
| 0.033170994 | 23.58 | 0.001777704 | 0.008507975 |
| 0.039727726 | 24.58 | 0.014236921 | 0.036716358 |
| 0.056362398 | 25.58 | 0.026639506 | 0.058630308 |
| 0.126665137 | 26.58 | 0.046234456 | 0.116445837 |
| 0.211659812 | 27.58 | 0.10745452 | 0.190230574 |
| 0.366592964 | 28.56 | 0.146191359 | 0.332671252 |
| 0.49432782 | 29.61 | 0.265856478 | 0.497259008 |
| 0.74214801 | 30.56 | 0.34440618 | 0.721177618 |
| 0.925979351 | 31.63 | 0.460899861 | 0.976568392 |
| 1.10701801 | 32.63 | 0.594836447 | 1.191511608 |
| 1.348281468 | 33.63 | 0.718975558 | 1.500405058 |
| 1.518392241 | 34.63 | 0.80460436 | 1.699379066 |
| 1.660211928 | 35.63 | 0.942505243 | 1.858022079 |
| 1.810288241 | 36.63 | 1.024169749 | 2.092897665 |
| 1.984163063 | 37.65 | 1.105834255 | 2.265761434 |
| 2.100484348 | 38.65 | 1.203582478 | 2.391300395 |
| 2.198228225 | 39.65 | 1.268936736 | 2.535722653 |

| Experiment derived parameter | linear regression value |
|---|---|
| y0 | -0.0014 |
| a | 0.4906 |
| **R** | **0.99728241** |
| **average** | |
| | value |
| y0 | -0.002133333 |
| **a** | 0.4672 |

| Well/ Cycle | Raw fluorescence data D4 | D5 | D6 | Well/ Cycle | D4 | D5 | D6 |
|---|---|---|---|---|---|---|---|
| 0.56 | 130.12 | 159.40 | 167.25 | 0.56 | -0.0472 | -0.04 | -0.03 |
| 1.56 | 132.71 | 163.22 | 173.07 | 1.56 | -0.0283 | -0.02 | 0.00 |
| 2.56 | 134.38 | 165.57 | 176.28 | 2.56 | -0.0160 | 0.00 | 0.02 |
| 3.56 | 136.57 | 164.06 | 172.85 | 3.56 | 0.0000 | -0.01 | 0.00 |
| 4.56 | 135.62 | 167.44 | 175.29 | 4.56 | -0.0069 | 0.01 | 0.02 |
| 5.56 | 134.38 | 164.99 | 173.56 | 5.56 | -0.0160 | -0.01 | 0.01 |
| 6.56 | 136.71 | 167.28 | 176.17 | 6.56 | 0.0010 | 0.01 | 0.02 |
| 7.56 | 137.55 | 167.57 | 174.01 | 7.56 | 0.0072 | 0.01 | 0.01 |
| 8.56 | 138.53 | 169.08 | 175.32 | 8.56 | 0.0144 | 0.02 | 0.02 |
| 9.56 | 137.69 | 166.72 | 174.92 | 9.56 | 0.0082 | 0.00 | 0.01 |
| 10.56 | 138.12 | 168.77 | 175.87 | 10.56 | 0.0114 | 0.02 | 0.02 |
| 11.56 | 137.34 | 166.81 | 171.73 | 11.56 | 0.0056 | 0.01 | 0.00 |
| 12.56 | 139.64 | 168.62 | 174.21 | 12.56 | 0.0225 | 0.02 | 0.01 |
| 13.56 | 136.04 | 165.84 | 174.19 | 13.56 | -0.0039 | 0.00 | 0.01 |
| 14.56 | 138.42 | 167.07 | 172.87 | 14.56 | 0.0136 | 0.01 | 0.00 |
| 15.56 | 136.57 | 166.75 | 172.86 | 15.56 | 0.0000 | 0.00 | 0.00 |
| 16.58 | 137.73 | 167.64 | 172.23 | 16.58 | 0.0085 | 0.01 | 0.00 |
| 17.58 | 137.93 | 164.82 | 172.49 | 17.58 | 0.0100 | -0.01 | 0.00 |
| 18.58 | 139.10 | 170.63 | 172.85 | 18.58 | 0.0185 | 0.03 | 0.00 |
| 19.58 | 138.81 | 168.06 | 174.83 | 19.58 | 0.0164 | 0.01 | 0.01 |
| 20.58 | 137.21 | 169.46 | 172.51 | 20.58 | 0.0047 | 0.02 | 0.00 |
| 21.58 | 138.98 | 167.97 | 175.13 | 21.58 | 0.0177 | 0.01 | 0.02 |
| 22.58 | 137.81 | 165.87 | 172.15 | 22.58 | 0.0091 | 0.00 | 0.00 |
| 23.58 | 140.14 | 170.61 | 173.76 | 23.58 | 0.0261 | 0.03 | 0.01 |
| 24.58 | 138.66 | 166.61 | 173.09 | 24.58 | 0.0153 | 0.00 | 0.00 |
| 25.58 | 141.77 | 172.42 | 177.98 | 25.58 | 0.0381 | 0.04 | 0.03 |
| 26.58 | 142.61 | 171.95 | 180.28 | 26.58 | 0.0442 | 0.04 | 0.05 |
| 27.58 | 145.95 | 181.45 | 190.71 | 27.58 | 0.0687 | 0.09 | 0.11 |
| 28.56 | 152.19 | 184.39 | 196.95 | 28.56 | 0.1144 | 0.11 | 0.14 |
| 29.61 | 161.59 | 198.60 | 223.82 | 29.61 | 0.1832 | 0.20 | 0.30 |
| 30.56 | 169.44 | 207.22 | 234.97 | 30.56 | 0.2407 | 0.25 | 0.36 |
| 31.63 | 178.72 | 221.06 | 252.30 | 31.63 | 0.3086 | 0.33 | 0.46 |
| 32.63 | 192.41 | 236.54 | 276.53 | 32.63 | 0.4089 | 0.43 | 0.60 |
| 33.63 | 210.33 | 247.54 | 291.29 | 33.63 | 0.5401 | 0.49 | 0.69 |
| 34.63 | 212.39 | 259.56 | 303.72 | 34.63 | 0.5552 | 0.56 | 0.76 |
| 35.63 | 244.92 | 274.48 | 325.54 | 35.63 | 0.7934 | 0.65 | 0.89 |
| 36.63 | 249.58 | 285.93 | 345.78 | 36.63 | 0.8275 | 0.72 | 1.01 |
| 37.65 | 257.20 | 293.70 | 355.61 | 37.65 | 0.8833 | 0.77 | 1.06 |
| 38.65 | 265.09 | 304.15 | 372.38 | 38.65 | 0.9411 | 0.83 | 1.16 |
| 39.65 | 275.23 | 315.73 | 379.81 | 39.65 | 1.0153 | 0.90 | 1.20 |
| Parameter | | | | | | | |
| a | 151.089800 | 159.660600 | 220.085300 | | | | |
| b | 2.521500 | 2.688400 | 2.563100 | | | | |
| x0 | 33.951000 | 33.527700 | 33.231600 | | | | |
| y0 | 136.568900 | 165.957200 | 172.377900 | | | | |
| R | 0.997774 | 0.998378 | 0.998223 | | | | |
| **$CP_{(SDM)}$** | 30.63 | 29.99 | 29.86 | | | | |

| Well/ | Raw fluorescence data | | | Well/ | | | |
|---|---|---|---|---|---|---|---|
| Cycle | D4 | D5 | D6 | Cycle | D4 | D5 | D6 |
| 0.56 | 89.42 | 132.83 | 115.58 | 0.56 | 0.0124 | 0.03 | 0.01 |
| 1.56 | 88.87 | 132.34 | 118.35 | 1.56 | 0.0062 | 0.02 | 0.03 |
| 2.56 | 89.12 | 132.52 | 118.78 | 2.56 | 0.0090 | 0.02 | 0.03 |
| 3.56 | 90.99 | 135.29 | 117.24 | 3.56 | 0.0302 | 0.04 | 0.02 |
| 4.56 | 89.36 | 131.99 | 114.71 | 4.56 | 0.0117 | 0.02 | 0.00 |
| 5.56 | 90.93 | 131.76 | 116.21 | 5.56 | 0.0295 | 0.02 | 0.01 |
| 6.56 | 90.68 | 132.86 | 118.00 | 6.56 | 0.0267 | 0.03 | 0.03 |
| 7.56 | 90.82 | 133.35 | 115.95 | 7.56 | 0.0282 | 0.03 | 0.01 |
| 8.56 | 90.03 | 132.73 | 118.47 | 8.56 | 0.0193 | 0.02 | 0.03 |
| 9.56 | 89.42 | 131.25 | 116.46 | 9.56 | 0.0124 | 0.01 | 0.01 |
| 10.56 | 91.13 | 130.20 | 116.19 | 10.56 | 0.0317 | 0.01 | 0.01 |
| 11.56 | 89.04 | 129.94 | 117.48 | 11.56 | 0.0081 | 0.00 | 0.02 |
| 12.56 | 90.07 | 130.76 | 117.68 | 12.56 | 0.0197 | 0.01 | 0.02 |
| 13.56 | 90.89 | 132.54 | 115.93 | 13.56 | 0.0290 | 0.02 | 0.01 |
| 14.56 | 89.82 | 130.31 | 116.66 | 14.56 | 0.0169 | 0.01 | 0.02 |
| 15.56 | 88.60 | 129.04 | 117.10 | 15.56 | 0.0031 | 0.00 | 0.02 |
| 16.58 | 89.00 | 130.12 | 115.37 | 16.58 | 0.0076 | 0.00 | 0.00 |
| 17.58 | 89.23 | 130.75 | 115.06 | 17.58 | 0.0102 | 0.01 | 0.00 |
| 18.58 | 88.77 | 130.25 | 115.85 | 18.58 | 0.0050 | 0.01 | 0.01 |
| 19.58 | 88.16 | 129.95 | 114.26 | 19.58 | -0.0019 | 0.00 | -0.01 |
| 20.58 | 88.80 | 131.91 | 114.31 | 20.58 | 0.0054 | 0.02 | -0.01 |
| 21.58 | 89.68 | 133.02 | 115.10 | 21.58 | 0.0153 | 0.03 | 0.00 |
| 22.58 | 90.83 | 129.16 | 115.98 | 22.58 | 0.0284 | 0.00 | 0.01 |
| 23.58 | 90.85 | 135.40 | 117.62 | 23.58 | 0.0286 | 0.05 | 0.02 |
| 24.58 | 94.67 | 136.50 | 120.88 | 24.58 | 0.0718 | 0.05 | 0.05 |
| 25.58 | 94.91 | 144.73 | 125.14 | 25.58 | 0.0745 | 0.12 | 0.09 |
| 26.58 | 101.98 | 158.13 | 134.30 | 26.58 | 0.1546 | 0.22 | 0.17 |
| 27.58 | 117.08 | 170.72 | 145.35 | 27.58 | 0.3255 | 0.32 | 0.27 |
| 28.56 | 135.57 | 200.09 | 170.21 | 28.56 | 0.5349 | 0.54 | 0.48 |
| 29.61 | 153.28 | 238.12 | 196.61 | 29.61 | 0.7354 | 0.84 | 0.71 |
| 30.56 | 178.38 | 288.54 | 232.29 | 30.56 | 1.0196 | 1.23 | 1.02 |
| 31.63 | 200.98 | 337.17 | 267.96 | 31.63 | 1.2754 | 1.60 | 1.33 |
| 32.63 | 222.84 | 382.22 | 301.34 | 32.63 | 1.5229 | 1.95 | 1.62 |
| 33.63 | 250.95 | 442.35 | 343.07 | 33.63 | 1.8412 | 2.42 | 1.99 |
| 34.63 | 267.97 | 483.61 | 374.59 | 34.63 | 2.0339 | 2.73 | 2.26 |
| 35.63 | 284.32 | 520.64 | 399.15 | 35.63 | 2.2190 | 3.02 | 2.47 |
| 36.63 | 301.25 | 553.62 | 423.02 | 36.63 | 2.4107 | 3.27 | 2.68 |
| 37.65 | 313.05 | 581.64 | 446.41 | 37.65 | 2.5443 | 3.49 | 2.89 |
| 38.65 | 328.24 | 615.28 | 459.04 | 38.65 | 2.7162 | 3.75 | 3.00 |
| 39.65 | 346.35 | 634.07 | 480.16 | 39.65 | 2.9213 | 3.90 | 3.18 |
| Parameter | | | | | | | |
| a | 259.448000 | 517.739900 | 370.682400 | | | | |
| b | 2.489800 | 2.348700 | 2.318700 | | | | |
| x0 | 32.464100 | 32.744000 | 32.599900 | | | | |
| y0 | 88.325700 | 129.519200 | 114.901000 | | | | |
| R | 0.998782 | 0.999383 | 0.999373 | | | | |
| **$CP_{(SDM)}$** | 29.19 | 29.65 | 29.55 | | | | |

| Well/ Cycle | FAM Normalized Fluorescence | VIC Normalized Fluorescence | Well/ Cycle | FAM Normalized Fluorescence |
|---|---|---|---|---|
| 0.56 | -0.047220853 | 0.012389373 | 0.56 | -0.039511392 |
| 1.56 | -0.028256067 | 0.006162419 | 1.56 | -0.016493409 |
| 2.56 | -0.016027807 | 0.008992853 | 2.56 | -0.002333132 |
| 3.56 | 8.05454E-06 | 0.030164493 | 3.56 | -0.011431863 |
| 4.56 | -0.006948141 | 0.011710069 | 4.56 | 0.008934834 |
| 5.56 | -0.016027807 | 0.029485189 | 5.56 | -0.005828009 |
| 6.56 | 0.001033178 | 0.026654756 | 6.56 | 0.00797073 |
| 7.56 | 0.00718392 | 0.028239799 | 7.56 | 0.009718168 |
| 8.56 | 0.014359785 | 0.01929563 | 8.56 | 0.0188169 |
| 9.56 | 0.008209043 | 0.012389373 | 9.56 | 0.004596366 |
| 10.56 | 0.011357637 | 0.031749536 | 10.56 | 0.016948948 |
| 11.56 | 0.005646234 | 0.008087114 | 11.56 | 0.005138674 |
| 12.56 | 0.02248755 | 0.019748499 | 12.56 | 0.016045101 |
| 13.56 | -0.00387277 | 0.02903232 | 13.56 | -0.000706206 |
| 14.56 | 0.01355433 | 0.016918066 | 14.56 | 0.006705343 |
| 15.56 | 8.05454E-06 | 0.003105551 | 15.56 | 0.004777135 |
| 16.58 | 0.008501936 | 0.007634245 | 16.58 | 0.010139964 |
| 17.58 | 0.009966398 | 0.010238243 | 17.58 | -0.006852369 |
| 18.58 | 0.018533502 | 0.005030246 | 18.58 | 0.028156657 |
| 19.58 | 0.016410032 | -0.001876011 | 19.58 | 0.012670737 |
| 20.58 | 0.004694334 | 0.005369898 | 20.58 | 0.021106647 |
| 21.58 | 0.017654825 | 0.015333023 | 21.58 | 0.012128428 |
| 22.58 | 0.009087721 | 0.028353016 | 22.58 | -0.000525437 |
| 23.58 | 0.026148706 | 0.028579451 | 23.58 | 0.028036144 |
| 24.58 | 0.015311685 | 0.071828471 | 24.58 | 0.003933544 |
| 25.58 | 0.038084073 | 0.074545687 | 25.58 | 0.038942571 |
| 26.58 | 0.044234815 | 0.15459034 | 26.58 | 0.036110515 |
| 27.58 | 0.068691335 | 0.32554851 | 27.58 | 0.09335419 |
| 28.56 | 0.114382557 | 0.534887354 | 28.56 | 0.111069601 |
| 29.61 | 0.183212283 | 0.735395247 | 29.61 | 0.196694087 |
| 30.56 | 0.240692427 | 1.019570748 | 30.56 | 0.24863519 |
| 31.63 | 0.308643476 | 1.275441916 | 31.63 | 0.332030186 |
| 32.63 | 0.408885918 | 1.522935001 | 32.63 | 0.425307248 |
| 33.63 | 0.540101736 | 1.841188918 | 33.63 | 0.491589398 |
| 34.63 | 0.555185697 | 2.033884815 | 34.63 | 0.564017711 |
| 35.63 | 0.793380484 | 2.218995151 | 35.63 | 0.653920408 |
| 36.63 | 0.827502455 | 2.410672092 | 36.63 | 0.722914101 |
| 37.65 | 0.883298467 | 2.544268542 | 37.65 | 0.769733401 |
| 38.65 | 0.941071503 | 2.716245668 | 38.65 | 0.832701444 |
| 39.65 | 1.01531974 | 2.921282254 | 39.65 | 0.90247847 |

| Experiment derived parameter | linear regression value |
|---|---|
| y0 | -0.0138 |
| a | 0.2631 |
| R | 0.99372558 |

| Experiment derived parameter | linear regression value |
|---|---|
| y0 | 0.0052 |
| a | 0.2098 |
| R | 0.9938927 |

| VIC Normalized Fluorescence | Well/ Cycle | FAM Normalized Fluorescence | VIC Normalized Fluorescence |
|---|---|---|---|
| 0.025562233 | 0.56 | -0.029748013 | 0.005909435 |
| 0.02177901 | 1.56 | 0.004015016 | 0.030017145 |
| 0.023168766 | 2.56 | 0.022636893 | 0.033759497 |
| 0.044555556 | 3.56 | 0.00273875 | 0.020356655 |
| 0.019076708 | 4.56 | 0.016893697 | -0.001662301 |
| 0.01730091 | 5.56 | 0.006857608 | 0.011392416 |
| 0.025793859 | 6.56 | 0.02199876 | 0.026971045 |
| 0.029577082 | 7.56 | 0.009468151 | 0.009129599 |
| 0.024790147 | 8.56 | 0.017067733 | 0.031061523 |
| 0.01336327 | 9.56 | 0.01474725 | 0.013568202 |
| 0.005256364 | 10.56 | 0.020258397 | 0.011218353 |
| 0.003248939 | 11.56 | -0.003758602 | 0.02244541 |
| 0.009580047 | 12.56 | 0.010628393 | 0.024186038 |
| 0.023323183 | 13.56 | 0.010512368 | 0.008955536 |
| 0.006105658 | 14.56 | 0.002854774 | 0.015308831 |
| -0.003699838 | 15.56 | 0.002796762 | 0.019138215 |
| 0.004638694 | 16.58 | -0.000857999 | 0.004081775 |
| 0.009502838 | 17.58 | 0.000650315 | 0.0013838 |
| 0.005642407 | 18.58 | 0.00273875 | 0.008259284 |
| 0.003326148 | 19.58 | 0.014225141 | -0.005578716 |
| 0.018459039 | 20.58 | 0.00076634 | -0.005143558 |
| 0.027029197 | 21.58 | 0.015965504 | 0.001731926 |
| -0.002773334 | 22.58 | -0.001322095 | 0.009390693 |
| 0.045404851 | 23.58 | 0.008017849 | 0.02366385 |
| 0.0538978 | 24.58 | 0.00413104 | 0.052036101 |
| 0.117440503 | 25.58 | 0.032498946 | 0.089111496 |
| 0.220900067 | 26.58 | 0.045841723 | 0.168832299 |
| 0.318105733 | 27.58 | 0.10634832 | 0.265002045 |
| 0.544867479 | 28.56 | 0.142547856 | 0.481362216 |
| 0.838491899 | 29.61 | 0.298426306 | 0.71112523 |
| 1.227777812 | 30.56 | 0.363109772 | 1.021653423 |
| 1.60324338 | 31.63 | 0.463644702 | 1.332094586 |
| 1.951068259 | 32.63 | 0.604207964 | 1.622605547 |
| 2.415323751 | 33.63 | 0.68983379 | 1.985787765 |
| 2.733886559 | 34.63 | 0.761942801 | 2.260110878 |
| 3.019790116 | 35.63 | 0.888525153 | 2.473860106 |
| 3.274424178 | 36.63 | 1.005941597 | 2.681604164 |
| 3.490762759 | 37.65 | 1.062967469 | 2.885170712 |
| 3.750492591 | 38.65 | 1.160253722 | 2.995091427 |
| 3.895567607 | 39.65 | 1.203356695 | 3.178901837 |

| Experiment derived parameter | linear regression value |
|---|---|
| y0 | -0.0063 |
| a | 0.3692 |
| **R** | **0.99274487** |

| **average** | value |
|---|---|
| y0 | -0.004966667 |
| **a** | 0.2807 |

| Well/ Cycle | Raw fluorescence data D7 | D8 | D9 | Well/ Cycle | D7 | D8 | D9 |
|---|---|---|---|---|---|---|---|
| 0.56 | 225.89 | 226.07 | 183.59 | 0.56 | -0.0453 | -0.03 | -0.04 |
| 1.56 | 234.51 | 230.34 | 186.41 | 1.56 | -0.0089 | -0.01 | -0.03 |
| 2.56 | 238.37 | 239.26 | 190.43 | 2.56 | 0.0074 | 0.02 | 0.00 |
| 3.56 | 236.82 | 234.46 | 188.54 | 3.56 | 0.0009 | 0.00 | -0.01 |
| 4.56 | 243.07 | 236.06 | 193.36 | 4.56 | 0.0273 | 0.01 | 0.01 |
| 5.56 | 239.87 | 235.25 | 191.98 | 5.56 | 0.0138 | 0.01 | 0.00 |
| 6.56 | 239.65 | 241.00 | 193.13 | 6.56 | 0.0128 | 0.03 | 0.01 |
| 7.56 | 237.17 | 236.74 | 193.63 | 7.56 | 0.0024 | 0.01 | 0.01 |
| 8.56 | 241.81 | 237.42 | 196.53 | 8.56 | 0.0220 | 0.02 | 0.03 |
| 9.56 | 240.76 | 236.41 | 190.68 | 9.56 | 0.0175 | 0.01 | 0.00 |
| 10.56 | 237.51 | 237.90 | 192.16 | 10.56 | 0.0038 | 0.02 | 0.00 |
| 11.56 | 236.44 | 233.28 | 192.30 | 11.56 | -0.0007 | 0.00 | 0.01 |
| 12.56 | 234.93 | 237.02 | 194.01 | 12.56 | -0.0071 | 0.01 | 0.01 |
| 13.56 | 235.44 | 234.39 | 190.47 | 13.56 | -0.0049 | 0.00 | 0.00 |
| 14.56 | 240.59 | 242.30 | 196.10 | 14.56 | 0.0168 | 0.04 | 0.03 |
| 15.56 | 236.41 | 235.80 | 192.10 | 15.56 | -0.0008 | 0.01 | 0.00 |
| 16.58 | 239.27 | 235.17 | 191.76 | 16.58 | 0.0112 | 0.01 | 0.00 |
| 17.58 | 238.13 | 235.80 | 194.26 | 17.58 | 0.0064 | 0.01 | 0.02 |
| 18.58 | 237.68 | 236.22 | 197.29 | 18.58 | 0.0045 | 0.01 | 0.03 |
| 19.58 | 238.06 | 235.05 | 192.13 | 19.58 | 0.0061 | 0.01 | 0.00 |
| 20.58 | 238.61 | 230.35 | 191.43 | 20.58 | 0.0085 | -0.01 | 0.00 |
| 21.58 | 239.83 | 231.07 | 193.21 | 21.58 | 0.0136 | -0.01 | 0.01 |
| 22.58 | 237.02 | 228.92 | 194.62 | 22.58 | 0.0017 | -0.02 | 0.02 |
| 23.58 | 239.52 | 234.22 | 193.93 | 23.58 | 0.0123 | 0.00 | 0.01 |
| 24.58 | 239.84 | 230.05 | 194.38 | 24.58 | 0.0137 | -0.02 | 0.02 |
| 25.58 | 246.26 | 238.06 | 199.83 | 25.58 | 0.0408 | 0.02 | 0.04 |
| 26.58 | 248.10 | 237.21 | 198.32 | 26.58 | 0.0486 | 0.02 | 0.04 |
| 27.58 | 252.71 | 253.67 | 208.14 | 27.58 | 0.0680 | 0.09 | 0.09 |
| 28.56 | 261.36 | 255.46 | 212.81 | 28.56 | 0.1046 | 0.09 | 0.11 |
| 29.61 | 277.45 | 275.13 | 230.39 | 29.61 | 0.1726 | 0.18 | 0.20 |
| 30.56 | 288.78 | 292.80 | 237.77 | 30.56 | 0.2205 | 0.25 | 0.24 |
| 31.63 | 300.33 | 310.05 | 251.25 | 31.63 | 0.2693 | 0.33 | 0.31 |
| 32.63 | 315.91 | 333.44 | 266.61 | 32.63 | 0.3351 | 0.43 | 0.39 |
| 33.63 | 324.10 | 345.74 | 283.38 | 33.63 | 0.3698 | 0.48 | 0.48 |
| 34.63 | 336.53 | 363.86 | 296.61 | 34.63 | 0.4223 | 0.56 | 0.55 |
| 35.63 | 348.71 | 389.24 | 314.95 | 35.63 | 0.4738 | 0.67 | 0.65 |
| 36.63 | 366.70 | 406.29 | 329.43 | 36.63 | 0.5498 | 0.74 | 0.72 |
| 37.65 | 374.11 | 418.76 | 345.42 | 37.65 | 0.5811 | 0.79 | 0.81 |
| 38.65 | 391.67 | 443.42 | 355.96 | 38.65 | 0.6553 | 0.90 | 0.86 |
| 39.65 | 399.58 | 458.60 | 366.15 | 39.65 | 0.6888 | 0.96 | 0.91 |

| Parameter | D7 | D8 | D9 |
|---|---|---|---|
| a | 182.901700 | 243.151300 | 197.443200 |
| b | 3.044000 | 2.659200 | 2.833300 |
| x0 | 33.835400 | 34.016500 | 34.088600 |
| y0 | 236.610200 | 233.667700 | 191.307000 |
| R | 0.997165 | 0.996890 | 0.998542 |
| $CP_{(SDM)}$ | 29.83 | 30.51 | 30.36 |

| Well/ Cycle | Raw fluorescence data D7 | D8 | D9 | Well/ Cycle | D7 | D8 | D9 |
|---|---|---|---|---|---|---|---|
| 0.56 | 111.95 | 111.23 | 100.10 | 0.56 | -0.0068 | 0.01 | -0.01 |
| 1.56 | 112.95 | 113.70 | 104.72 | 1.56 | 0.0021 | 0.03 | 0.03 |
| 2.56 | 114.32 | 112.04 | 101.52 | 2.56 | 0.0142 | 0.02 | 0.00 |
| 3.56 | 116.45 | 112.96 | 104.12 | 3.56 | 0.0331 | 0.02 | 0.03 |
| 4.56 | 112.93 | 112.70 | 103.34 | 4.56 | 0.0019 | 0.02 | 0.02 |
| 5.56 | 114.42 | 115.36 | 102.30 | 5.56 | 0.0151 | 0.05 | 0.01 |
| 6.56 | 111.88 | 115.07 | 102.97 | 6.56 | -0.0074 | 0.04 | 0.02 |
| 7.56 | 114.25 | 114.87 | 102.98 | 7.56 | 0.0136 | 0.04 | 0.02 |
| 8.56 | 114.63 | 113.40 | 103.48 | 8.56 | 0.0170 | 0.03 | 0.02 |
| 9.56 | 115.12 | 114.07 | 104.03 | 9.56 | 0.0213 | 0.03 | 0.03 |
| 10.56 | 114.93 | 111.77 | 103.67 | 10.56 | 0.0196 | 0.01 | 0.02 |
| 11.56 | 115.98 | 112.58 | 104.10 | 11.56 | 0.0289 | 0.02 | 0.03 |
| 12.56 | 115.53 | 112.70 | 103.18 | 12.56 | 0.0249 | 0.02 | 0.02 |
| 13.56 | 114.56 | 112.84 | 102.94 | 13.56 | 0.0163 | 0.02 | 0.02 |
| 14.56 | 112.69 | 111.62 | 103.21 | 14.56 | -0.0003 | 0.01 | 0.02 |
| 15.56 | 113.90 | 112.97 | 102.21 | 15.56 | 0.0105 | 0.02 | 0.01 |
| 16.58 | 116.05 | 111.21 | 101.86 | 16.58 | 0.0296 | 0.01 | 0.01 |
| 17.58 | 114.89 | 112.50 | 102.79 | 17.58 | 0.0193 | 0.02 | 0.02 |
| 18.58 | 116.11 | 110.02 | 102.78 | 18.58 | 0.0301 | 0.00 | 0.02 |
| 19.58 | 114.12 | 107.88 | 101.21 | 19.58 | 0.0124 | -0.02 | 0.00 |
| 20.58 | 115.83 | 111.37 | 101.37 | 20.58 | 0.0276 | 0.01 | 0.00 |
| 21.58 | 114.51 | 109.26 | 102.65 | 21.58 | 0.0159 | -0.01 | 0.01 |
| 22.58 | 118.46 | 111.24 | 102.68 | 22.58 | 0.0509 | 0.01 | 0.01 |
| 23.58 | 119.67 | 112.65 | 104.29 | 23.58 | 0.0617 | 0.02 | 0.03 |
| 24.58 | 122.65 | 116.51 | 106.31 | 24.58 | 0.0881 | 0.06 | 0.05 |
| 25.58 | 128.85 | 120.53 | 111.72 | 25.58 | 0.1431 | 0.09 | 0.10 |
| 26.58 | 140.67 | 134.71 | 121.02 | 26.58 | 0.2480 | 0.22 | 0.20 |
| 27.58 | 152.94 | 147.78 | 133.88 | 27.58 | 0.3568 | 0.34 | 0.32 |
| 28.56 | 177.11 | 176.93 | 159.40 | 28.56 | 0.5713 | 0.60 | 0.57 |
| 29.61 | 203.92 | 211.27 | 184.75 | 29.61 | 0.8091 | 0.91 | 0.83 |
| 30.56 | 233.49 | 260.67 | 222.26 | 30.56 | 1.0714 | 1.36 | 1.20 |
| 31.63 | 263.50 | 305.79 | 260.15 | 31.63 | 1.3377 | 1.77 | 1.57 |
| 32.63 | 288.75 | 345.64 | 294.68 | 32.63 | 1.5617 | 2.13 | 1.91 |
| 33.63 | 322.76 | 400.63 | 332.94 | 33.63 | 1.8634 | 2.63 | 2.29 |
| 34.63 | 350.26 | 431.09 | 365.84 | 34.63 | 2.1074 | 2.91 | 2.61 |
| 35.63 | 367.06 | 466.07 | 388.81 | 35.63 | 2.2564 | 3.22 | 2.84 |
| 36.63 | 390.09 | 499.44 | 412.98 | 36.63 | 2.4607 | 3.53 | 3.08 |
| 37.65 | 412.17 | 529.33 | 431.33 | 37.65 | 2.6566 | 3.80 | 3.26 |
| 38.65 | 433.18 | 542.28 | 454.01 | 38.65 | 2.8430 | 3.91 | 3.49 |
| 39.65 | 448.73 | 564.97 | 466.62 | 39.65 | 2.9810 | 4.12 | 3.61 |

| Parameter | D7 | D8 | D9 |
|---|---|---|---|
| a | 349.345500 | 461.967300 | 371.601300 |
| b | 2.680500 | 2.283700 | 2.308900 |
| x0 | 32.586700 | 32.540200 | 32.481900 |
| y0 | 112.718700 | 110.348500 | 101.223400 |
| R | 0.999158 | 0.999234 | 0.999359 |
| **$CP_{(SDM)}$** | 29.06 | 29.53 | 29.44 |

| D7 | | | D8 | |
|---|---|---|---|---|
| Well/ Cycle | FAM Normalized Fluorescence | VIC Normalized Fluorescence | Well/ Cycle | FAM Normalized Fluorescence |
| 0.56 | -0.04530743 | -0.006819632 | 0.56 | -0.032514977 |
| 1.56 | -0.008876202 | 0.002052011 | 1.56 | -0.014241164 |
| 2.56 | 0.007437549 | 0.014206161 | 2.56 | 0.023932704 |
| 3.56 | 0.00088669 | 0.033102759 | 3.56 | 0.003390713 |
| 4.56 | 0.027301443 | 0.001874578 | 4.56 | 0.010238043 |
| 5.56 | 0.01377709 | 0.015093325 | 5.56 | 0.006771582 |
| 6.56 | 0.012847291 | -0.007440646 | 6.56 | 0.031379176 |
| 7.56 | 0.002365917 | 0.013585146 | 7.56 | 0.013148159 |
| 8.56 | 0.021976229 | 0.01695637 | 8.56 | 0.016058274 |
| 9.56 | 0.017538551 | 0.021303475 | 9.56 | 0.011735897 |
| 10.56 | 0.003802879 | 0.019617863 | 10.56 | 0.018112473 |
| 11.56 | -0.000719327 | 0.028933087 | 11.56 | -0.001659194 |
| 12.56 | -0.007101131 | 0.024940848 | 12.56 | 0.014346442 |
| 13.56 | -0.004945687 | 0.016335355 | 13.56 | 0.003091142 |
| 14.56 | 0.016820069 | -0.000254616 | 14.56 | 0.036942633 |
| 15.56 | -0.000846117 | 0.010480071 | 15.56 | 0.009125352 |
| 16.58 | 0.011241274 | 0.029554102 | 16.58 | 0.006429216 |
| 17.58 | 0.006423223 | 0.019262997 | 17.58 | 0.009125352 |
| 18.58 | 0.00452136 | 0.030086401 | 18.58 | 0.010922776 |
| 19.58 | 0.006127377 | 0.012431833 | 19.58 | 0.005915666 |
| 20.58 | 0.008451876 | 0.027602341 | 20.58 | -0.014198368 |
| 21.58 | 0.013608035 | 0.015891773 | 21.58 | -0.011117069 |
| 22.58 | 0.001731963 | 0.050934761 | 22.58 | -0.02031817 |
| 23.58 | 0.012297864 | 0.061669448 | 23.58 | 0.002363613 |
| 24.58 | 0.013650299 | 0.088106942 | 24.58 | -0.015482243 |
| 25.58 | 0.040783533 | 0.143111125 | 25.58 | 0.018797206 |
| 26.58 | 0.048560037 | 0.247973939 | 26.58 | 0.015159562 |
| 27.58 | 0.068043559 | 0.356828991 | 27.58 | 0.085601476 |
| 28.56 | 0.104601577 | 0.571256588 | 28.56 | 0.093261927 |
| 29.61 | 0.172603717 | 0.809105321 | 29.61 | 0.177441298 |
| 30.56 | 0.220488381 | 1.071439788 | 30.56 | 0.253061506 |
| 31.63 | 0.269302845 | 1.337677777 | 31.63 | 0.326884289 |
| 32.63 | 0.335149541 | 1.561686748 | 32.63 | 0.426983704 |
| 33.63 | 0.369763434 | 1.863411306 | 33.63 | 0.479622558 |
| 34.63 | 0.422297095 | 2.107381473 | 34.63 | 0.557168577 |
| 35.63 | 0.473774165 | 2.256425065 | 35.63 | 0.66578436 |
| 36.63 | 0.54980639 | 2.46073899 | 36.63 | 0.738751227 |
| 37.65 | 0.581123722 | 2.656624855 | 37.65 | 0.79211761 |
| 38.65 | 0.655338612 | 2.843018062 | 38.65 | 0.897652093 |
| 39.65 | 0.688769123 | 2.980972101 | 39.65 | 0.962616142 |

| Experiment derived parameter | linear regression value |
|---|---|
| y0 | -0.0093 |
| a | 0.2155 |
| R | 0.99768911 |

| Experiment derived parameter | linear regression value |
|---|---|
| y0 | -0.0126 |
| a | 0.1997 |
| R | 0.99265544 |

| | D9 | | |
|---|---|---|---|
| VIC Normalized Fluorescence | Well/ Cycle | FAM Normalized Fluorescence | VIC Normalized Fluorescence |
| 0.007988328 | 0.56 | -0.040338304 | -0.011098224 |
| 0.030371958 | 1.56 | -0.0255976 | 0.034543396 |
| 0.015328709 | 2.56 | -0.004584255 | 0.002930153 |
| 0.023665931 | 3.56 | -0.014463663 | 0.028615913 |
| 0.02130976 | 4.56 | 0.010731442 | 0.020910185 |
| 0.045415207 | 5.56 | 0.003517906 | 0.010635881 |
| 0.04278717 | 6.56 | 0.009529186 | 0.017254904 |
| 0.04097473 | 7.56 | 0.012142786 | 0.017353695 |
| 0.027653298 | 8.56 | 0.027301667 | 0.022293264 |
| 0.033724971 | 9.56 | -0.003277455 | 0.02772679 |
| 0.012881915 | 10.56 | 0.004458802 | 0.024170301 |
| 0.020222296 | 11.56 | 0.00519061 | 0.02841833 |
| 0.02130976 | 12.56 | 0.014129122 | 0.019329523 |
| 0.022578467 | 13.56 | -0.004375167 | 0.016958529 |
| 0.011522585 | 14.56 | 0.025053971 | 0.019625897 |
| 0.023756553 | 15.56 | 0.00414517 | 0.009746758 |
| 0.007807084 | 16.58 | 0.002367922 | 0.00628906 |
| 0.01949732 | 17.58 | 0.015435922 | 0.015476659 |
| -0.002976932 | 18.58 | 0.031274339 | 0.015377867 |
| -0.022370037 | 19.58 | 0.004301986 | -0.00013238 |
| 0.009257036 | 20.58 | 0.000642946 | 0.001448282 |
| -0.009864203 | 21.58 | 0.009947362 | 0.014093579 |
| 0.00807895 | 22.58 | 0.017317714 | 0.014389953 |
| 0.02085665 | 23.58 | 0.013710946 | 0.030295366 |
| 0.055836735 | 24.58 | 0.016063186 | 0.050251226 |
| 0.092266773 | 25.58 | 0.044551428 | 0.103697366 |
| 0.220768746 | 26.58 | 0.036658355 | 0.195573356 |
| 0.339211679 | 27.58 | 0.087989462 | 0.322619078 |
| 0.603374763 | 28.56 | 0.112400487 | 0.574734696 |
| 0.914570656 | 29.61 | 0.204294668 | 0.82517086 |
| 1.362243257 | 30.56 | 0.242871406 | 1.195737349 |
| 1.771129648 | 31.63 | 0.313334065 | 1.570057912 |
| 2.132258255 | 32.63 | 0.393623861 | 1.911184568 |
| 2.630588544 | 33.63 | 0.481284009 | 2.289160412 |
| 2.906623108 | 34.63 | 0.550439869 | 2.614184072 |
| 3.223618808 | 35.63 | 0.646306722 | 2.841107886 |
| 3.526024368 | 36.63 | 0.721996581 | 3.079886667 |
| 3.796893478 | 37.65 | 0.805579514 | 3.26116886 |
| 3.914248948 | 38.65 | 0.860674204 | 3.485227724 |
| 4.119870229 | 39.65 | 0.913939375 | 3.609803662 |

| **Experiment derived parameter** | linear regression **value** |
|---|---|
| y0 | 0.0121 |
| a | 0.1981 |
| **R** | **0.99242474** |

| | value |
|---|---|
| y0 | -0.003266667 |
| **a** | 0.204433333 |

| Well/ Cycle | Raw fluorescence data E2 | E3 | E4 | Well/ Cycle | E2 | E3 | E4 |
|---|---|---|---|---|---|---|---|
| 0.56 | 165.26 | 174.18 | 167.18 | 0.56 | -0.0483 | -0.04 | -0.04 |
| 1.56 | 172.18 | 178.01 | 169.74 | 1.56 | -0.0085 | -0.02 | -0.02 |
| 2.56 | 175.10 | 179.49 | 171.92 | 2.56 | 0.0083 | -0.01 | -0.01 |
| 3.56 | 172.52 | 181.59 | 172.00 | 3.56 | -0.0065 | 0.00 | -0.01 |
| 4.56 | 176.30 | 182.00 | 173.00 | 4.56 | 0.0152 | 0.00 | 0.00 |
| 5.56 | 174.99 | 179.64 | 170.59 | 5.56 | 0.0077 | -0.01 | -0.02 |
| 6.56 | 176.74 | 183.15 | 173.00 | 6.56 | 0.0178 | 0.01 | 0.00 |
| 7.56 | 175.02 | 183.56 | 174.99 | 7.56 | 0.0079 | 0.01 | 0.01 |
| 8.56 | 175.77 | 182.35 | 175.35 | 8.56 | 0.0122 | 0.00 | 0.01 |
| 9.56 | 173.53 | 182.22 | 176.71 | 9.56 | -0.0007 | 0.00 | 0.02 |
| 10.56 | 176.95 | 185.04 | 177.27 | 10.56 | 0.0190 | 0.02 | 0.02 |
| 11.56 | 174.70 | 182.55 | 174.44 | 11.56 | 0.0060 | 0.01 | 0.00 |
| 12.56 | 176.69 | 185.48 | 174.97 | 12.56 | 0.0175 | 0.02 | 0.01 |
| 13.56 | 176.94 | 184.48 | 174.18 | 13.56 | 0.0189 | 0.02 | 0.00 |
| 14.56 | 176.09 | 184.18 | 175.68 | 14.56 | 0.0140 | 0.01 | 0.01 |
| 15.56 | 173.37 | 180.65 | 174.39 | 15.56 | -0.0016 | 0.00 | 0.00 |
| 16.58 | 175.81 | 184.24 | 176.09 | 16.58 | 0.0124 | 0.01 | 0.01 |
| 17.58 | 174.20 | 182.32 | 175.83 | 17.58 | 0.0031 | 0.00 | 0.01 |
| 18.58 | 175.97 | 185.38 | 177.39 | 18.58 | 0.0133 | 0.02 | 0.02 |
| 19.58 | 173.70 | 183.37 | 176.01 | 19.58 | 0.0003 | 0.01 | 0.01 |
| 20.58 | 173.58 | 179.50 | 173.94 | 20.58 | -0.0004 | -0.01 | 0.00 |
| 21.58 | 173.73 | 183.74 | 177.93 | 21.58 | 0.0004 | 0.01 | 0.02 |
| 22.58 | 170.09 | 181.22 | 175.36 | 22.58 | -0.0205 | 0.00 | 0.01 |
| 23.58 | 176.10 | 182.82 | 178.35 | 23.58 | 0.0141 | 0.01 | 0.03 |
| 24.58 | 175.10 | 183.76 | 174.47 | 24.58 | 0.0083 | 0.01 | 0.00 |
| 25.58 | 177.30 | 186.99 | 177.80 | 25.58 | 0.0210 | 0.03 | 0.02 |
| 26.58 | 178.29 | 185.08 | 177.76 | 26.58 | 0.0267 | 0.02 | 0.02 |
| 27.58 | 186.40 | 192.26 | 183.39 | 27.58 | 0.0734 | 0.06 | 0.06 |
| 28.56 | 188.48 | 192.41 | 183.91 | 28.56 | 0.0854 | 0.06 | 0.06 |
| 29.61 | 202.29 | 203.31 | 194.22 | 29.61 | 0.1649 | 0.12 | 0.12 |
| 30.56 | 210.27 | 212.04 | 197.17 | 30.56 | 0.2109 | 0.17 | 0.14 |
| 31.63 | 221.79 | 218.62 | 202.67 | 31.63 | 0.2772 | 0.20 | 0.17 |
| 32.63 | 235.80 | 237.33 | 214.64 | 32.63 | 0.3579 | 0.31 | 0.24 |
| 33.63 | 245.01 | 244.29 | 221.36 | 33.63 | 0.4109 | 0.35 | 0.27 |
| 34.63 | 253.40 | 254.55 | 228.34 | 34.63 | 0.4592 | 0.40 | 0.31 |
| 35.63 | 270.23 | 266.31 | 241.63 | 35.63 | 0.5561 | 0.47 | 0.39 |
| 36.63 | 280.91 | 274.86 | 249.16 | 36.63 | 0.6176 | 0.51 | 0.43 |
| 37.65 | 286.76 | 287.37 | 254.77 | 37.65 | 0.6513 | 0.58 | 0.47 |
| 38.65 | 301.78 | 298.16 | 263.88 | 38.65 | 0.7378 | 0.64 | 0.52 |
| 39.65 | 308.24 | 304.75 | 275.46 | 39.65 | 0.7750 | 0.68 | 0.59 |
| a | 146.345300 | 136.996900 | 120.808000 | | | | |
| b | 2.697000 | 2.699200 | 3.094900 | | | | |
| x0 | 33.795000 | 34.190900 | 35.049400 | | | | |
| y0 | 173.653200 | 181.556400 | 173.703500 | | | | |
| R | 0.997336 | 0.997354 | 0.996683 | | | | |
| **$CP_{(SDM)}$** | 30.24 | 30.64 | 30.97 | | | | |

| Well/ Cycle | Raw fluorescence data E2 | E3 | E4 | Well/ Cycle | E2 | E3 | E4 |
|---|---|---|---|---|---|---|---|
| 0.56 | 84.95 | 90.20 | 105.44 | 0.56 | -0.0066 | -0.03 | 0.00 |
| 1.56 | 85.60 | 92.42 | 106.52 | 1.56 | 0.0010 | 0.00 | 0.01 |
| 2.56 | 86.36 | 92.63 | 105.15 | 2.56 | 0.0099 | 0.00 | 0.00 |
| 3.56 | 86.88 | 91.43 | 105.57 | 3.56 | 0.0159 | -0.01 | 0.00 |
| 4.56 | 86.59 | 93.04 | 105.32 | 4.56 | 0.0126 | 0.00 | 0.00 |
| 5.56 | 87.19 | 94.87 | 107.99 | 5.56 | 0.0196 | 0.02 | 0.02 |
| 6.56 | 87.21 | 93.49 | 105.87 | 6.56 | 0.0198 | 0.01 | 0.00 |
| 7.56 | 86.23 | 93.83 | 105.77 | 7.56 | 0.0083 | 0.01 | 0.00 |
| 8.56 | 85.89 | 93.66 | 106.24 | 8.56 | 0.0044 | 0.01 | 0.01 |
| 9.56 | 86.04 | 94.49 | 105.75 | 9.56 | 0.0061 | 0.02 | 0.00 |
| 10.56 | 87.05 | 92.63 | 106.48 | 10.56 | 0.0179 | 0.00 | 0.01 |
| 11.56 | 87.40 | 93.97 | 106.91 | 11.56 | 0.0220 | 0.01 | 0.01 |
| 12.56 | 87.36 | 95.55 | 106.72 | 12.56 | 0.0216 | 0.03 | 0.01 |
| 13.56 | 87.30 | 94.70 | 107.58 | 13.56 | 0.0209 | 0.02 | 0.02 |
| 14.56 | 87.41 | 93.49 | 108.60 | 14.56 | 0.0221 | 0.01 | 0.03 |
| 15.56 | 87.68 | 94.10 | 107.33 | 15.56 | 0.0253 | 0.02 | 0.02 |
| 16.58 | 85.77 | 92.76 | 107.75 | 16.58 | 0.0030 | 0.00 | 0.02 |
| 17.58 | 87.71 | 92.94 | 108.40 | 17.58 | 0.0256 | 0.00 | 0.03 |
| 18.58 | 86.05 | 93.66 | 108.01 | 18.58 | 0.0062 | 0.01 | 0.02 |
| 19.58 | 87.28 | 94.59 | 109.45 | 19.58 | 0.0206 | 0.02 | 0.04 |
| 20.58 | 86.59 | 95.42 | 108.35 | 20.58 | 0.0126 | 0.03 | 0.03 |
| 21.58 | 86.90 | 96.57 | 109.35 | 21.58 | 0.0162 | 0.04 | 0.04 |
| 22.58 | 88.18 | 95.80 | 109.57 | 22.58 | 0.0311 | 0.03 | 0.04 |
| 23.58 | 89.88 | 97.23 | 112.09 | 23.58 | 0.0510 | 0.05 | 0.06 |
| 24.58 | 92.56 | 99.26 | 115.64 | 24.58 | 0.0824 | 0.07 | 0.10 |
| 25.58 | 97.58 | 101.97 | 119.63 | 25.58 | 0.1411 | 0.10 | 0.13 |
| 26.58 | 106.98 | 111.92 | 131.35 | 26.58 | 0.2510 | 0.21 | 0.24 |
| 27.58 | 119.15 | 120.98 | 143.49 | 27.58 | 0.3933 | 0.31 | 0.36 |
| 28.56 | 141.93 | 142.42 | 171.93 | 28.56 | 0.6597 | 0.54 | 0.63 |
| 29.61 | 166.64 | 166.32 | 201.71 | 29.61 | 0.9486 | 0.80 | 0.91 |
| 30.56 | 196.83 | 196.89 | 243.97 | 30.56 | 1.3017 | 1.13 | 1.31 |
| 31.63 | 220.23 | 226.45 | 278.01 | 31.63 | 1.5753 | 1.45 | 1.63 |
| 32.63 | 244.53 | 254.02 | 315.69 | 32.63 | 1.8594 | 1.74 | 1.99 |
| 33.63 | 275.81 | 286.85 | 357.37 | 33.63 | 2.2252 | 2.10 | 2.39 |
| 34.63 | 297.90 | 313.34 | 384.89 | 34.63 | 2.4835 | 2.38 | 2.65 |
| 35.63 | 312.03 | 328.82 | 410.99 | 35.63 | 2.6488 | 2.55 | 2.89 |
| 36.63 | 327.39 | 347.98 | 434.69 | 36.63 | 2.8284 | 2.76 | 3.12 |
| 37.65 | 339.04 | 359.37 | 448.76 | 37.65 | 2.9646 | 2.88 | 3.25 |
| 38.65 | 349.66 | 374.70 | 473.53 | 38.65 | 3.0888 | 3.05 | 3.49 |
| 39.65 | 362.58 | 386.27 | 488.78 | 39.65 | 3.2399 | 3.17 | 3.63 |

| Parameter | E2 | E3 | E4 |
|---|---|---|---|
| a | 277.385900 | 296.198200 | 387.529200 |
| b | 2.330700 | 2.277900 | 2.358600 |
| x0 | 31.825800 | 32.189500 | 32.256500 |
| y0 | 85.516700 | 92.608200 | 105.520200 |
| R | 0.999374 | 0.999502 | 0.999389 |
| $CP_{(SDM)}$ | 28.76 | 29.19 | 29.15 |

| E2 | | | E3 | |
|---|---|---|---|---|
| Well/ Cycle | FAM Normalized Fluorescence | VIC Normalized Fluorescence | Well/ Cycle | FAM Normalized Fluorescence |
| 0.56 | -0.048333115 | -0.006626776 | 0.56 | -0.040628697 |
| 1.56 | -0.008483575 | 0.000974079 | 1.56 | -0.019533324 |
| 2.56 | 0.008331548 | 0.009861232 | 2.56 | -0.011381587 |
| 3.56 | -0.00652565 | 0.015941915 | 3.56 | 0.000185066 |
| 4.56 | 0.015241873 | 0.012550765 | 4.56 | 0.002443318 |
| 5.56 | 0.007698102 | 0.019566938 | 5.56 | -0.010555398 |
| 6.56 | 0.017775659 | 0.019800811 | 6.56 | 0.008777438 |
| 7.56 | 0.00787086 | 0.008341061 | 7.56 | 0.011035689 |
| 8.56 | 0.012189813 | 0.004365229 | 8.56 | 0.004371094 |
| 9.56 | -0.00070946 | 0.006119273 | 9.56 | 0.003655063 |
| 10.56 | 0.018984965 | 0.017929831 | 10.56 | 0.019187426 |
| 11.56 | 0.006028107 | 0.022022599 | 11.56 | 0.00547268 |
| 12.56 | 0.017487728 | 0.021554854 | 12.56 | 0.021610915 |
| 13.56 | 0.018927379 | 0.020853237 | 13.56 | 0.016102985 |
| 14.56 | 0.014032566 | 0.022139535 | 14.56 | 0.014450606 |
| 15.56 | -0.001630837 | 0.025296813 | 15.56 | -0.004992388 |
| 16.58 | 0.012420157 | 0.002961995 | 16.58 | 0.014781082 |
| 17.58 | 0.003148805 | 0.025647622 | 17.58 | 0.004205856 |
| 18.58 | 0.013341534 | 0.006236209 | 18.58 | 0.021060122 |
| 19.58 | 0.000269503 | 0.020619364 | 19.58 | 0.009989182 |
| 20.58 | -0.00042153 | 0.012550765 | 20.58 | -0.011326508 |
| 21.58 | 0.000442261 | 0.016175788 | 21.58 | 0.012027117 |
| 22.58 | -0.020519058 | 0.031143625 | 22.58 | -0.001852868 |
| 23.58 | 0.014090152 | 0.051022783 | 23.58 | 0.006959821 |
| 24.58 | 0.008331548 | 0.082361691 | 24.58 | 0.012137275 |
| 25.58 | 0.021000477 | 0.141063675 | 25.58 | 0.02992789 |
| 26.58 | 0.026701495 | 0.250983726 | 26.58 | 0.019407743 |
| 27.58 | 0.073403773 | 0.393295111 | 27.58 | 0.058954683 |
| 28.56 | 0.085381669 | 0.659675829 | 28.56 | 0.059780873 |
| 29.61 | 0.164907989 | 0.948625239 | 29.61 | 0.119817313 |
| 30.56 | 0.210861648 | 1.3016557 | 30.56 | 0.167901545 |
| 31.63 | 0.277200766 | 1.575286465 | 31.63 | 0.204143726 |
| 32.63 | 0.357878807 | 1.859441489 | 32.63 | 0.307197102 |
| 33.63 | 0.410915549 | 2.225217998 | 33.63 | 0.345532297 |
| 34.63 | 0.459230236 | 2.483530118 | 34.63 | 0.402043662 |
| 35.63 | 0.55614754 | 2.648761002 | 35.63 | 0.466816923 |
| 36.63 | 0.61764943 | 2.828375043 | 36.63 | 0.513909727 |
| 37.65 | 0.651337263 | 2.964605744 | 37.65 | 0.582813936 |
| 38.65 | 0.737831494 | 3.088792014 | 38.65 | 0.642244504 |
| 39.65 | 0.775032075 | 3.239873615 | 39.65 | 0.678541764 |

| Experiment derived parameter | linear regression value |
|---|---|
| y0 | -0.0147 |
| a | 0.1807 |
| **R** | **0.99006553** |

| Experiment derived parameter | linear regression value |
|---|---|
| y0 | -0.0026 |
| a | 0.1461 |
| **R** | **0.9859991** |

| VIC Normalized Fluorescence | Well/ Cycle | E4 FAM Normalized Fluorescence | VIC Normalized Fluorescence |
|---|---|---|---|
| -0.026004177 | 0.56 | -0.037555375 | -0.000760044 |
| -0.002032217 | 1.56 | -0.022817617 | 0.009474963 |
| 0.0002354 | 2.56 | -0.010267496 | -0.003508333 |
| -0.012722416 | 3.56 | -0.009806941 | 0.000471948 |
| 0.004662654 | 4.56 | -0.004050005 | -0.001897267 |
| 0.024423323 | 5.56 | -0.017924221 | 0.023405945 |
| 0.009521835 | 6.56 | -0.004050005 | 0.003315005 |
| 0.013193216 | 7.56 | 0.007406299 | 0.002367319 |
| 0.011357526 | 8.56 | 0.009478796 | 0.006821443 |
| 0.020320015 | 9.56 | 0.017308229 | 0.002177782 |
| 0.0002354 | 10.56 | 0.020532114 | 0.009095889 |
| 0.014704961 | 11.56 | 0.004239984 | 0.013170938 |
| 0.031766086 | 12.56 | 0.00729116 | 0.011370335 |
| 0.022587633 | 13.56 | 0.00274318 | 0.019520433 |
| 0.009521835 | 14.56 | 0.011378585 | 0.029186829 |
| 0.016108725 | 15.56 | 0.003952137 | 0.017151218 |
| 0.001639164 | 16.58 | 0.013738929 | 0.021131499 |
| 0.003582836 | 17.58 | 0.012242125 | 0.027291457 |
| 0.011357526 | 18.58 | 0.021222946 | 0.023595482 |
| 0.021399833 | 19.58 | 0.013278374 | 0.037242158 |
| 0.030362322 | 20.58 | 0.001361515 | 0.026817614 |
| 0.042780229 | 21.58 | 0.024331692 | 0.036294473 |
| 0.03446563 | 22.58 | 0.009536365 | 0.038379381 |
| 0.049907028 | 23.58 | 0.026749605 | 0.062261065 |
| 0.071827333 | 24.58 | 0.004412692 | 0.095903912 |
| 0.1010904 | 25.58 | 0.02358329 | 0.133716577 |
| 0.20853229 | 26.58 | 0.023353012 | 0.244785359 |
| 0.306363799 | 27.58 | 0.055764564 | 0.35983442 |
| 0.537876775 | 28.56 | 0.058758171 | 0.629356275 |
| 0.795953274 | 29.61 | 0.118112185 | 0.91157712 |
| 1.126053632 | 30.56 | 0.135095148 | 1.312069158 |
| 1.445247829 | 31.63 | 0.166758298 | 1.63466142 |
| 1.742953648 | 32.63 | 0.235668826 | 1.991749447 |
| 2.097457892 | 33.63 | 0.274355439 | 2.386744908 |
| 2.383501677 | 34.63 | 0.314538855 | 2.647548052 |
| 2.550657501 | 35.63 | 0.39104854 | 2.894894058 |
| 2.757550627 | 36.63 | 0.434398271 | 3.119495604 |
| 2.880541896 | 37.65 | 0.466694684 | 3.252835002 |
| 3.046077993 | 38.65 | 0.519140374 | 3.487576786 |
| 3.171012934 | 39.65 | 0.585805698 | 3.632098878 |

| Experiment derived parameter | linear regression value |
|---|---|
| y0 | 0.0092 |
| a | 0.0987 |
| **R** | **0.97456685** |

| **AVERAGE** | value |
|---|---|
| y0 | -0.0027 |
| **a** | 0.141833333 |

| Well/ Cycle | Raw fluorescence data E5 | E6 | E7 | Well/ Cycle | E5 | E6 | E7 |
|---|---|---|---|---|---|---|---|
| 0.56 | 185.08 | 193.07 | 211.09 | 0.56 | -0.0363 | -0.04 | -0.03 |
| 1.56 | 190.20 | 196.16 | 213.70 | 1.56 | -0.0096 | -0.02 | -0.02 |
| 2.56 | 191.13 | 199.41 | 219.49 | 2.56 | -0.0048 | -0.01 | 0.01 |
| 3.56 | 190.14 | 198.09 | 216.04 | 3.56 | -0.0099 | -0.01 | 0.00 |
| 4.56 | 194.32 | 200.10 | 219.81 | 4.56 | 0.0118 | 0.00 | 0.01 |
| 5.56 | 193.15 | 195.97 | 219.84 | 5.56 | 0.0057 | -0.02 | 0.01 |
| 6.56 | 192.13 | 201.74 | 219.24 | 6.56 | 0.0004 | 0.00 | 0.01 |
| 7.56 | 191.04 | 200.88 | 217.43 | 7.56 | -0.0052 | 0.00 | 0.00 |
| 8.56 | 192.92 | 203.18 | 217.20 | 8.56 | 0.0046 | 0.01 | 0.00 |
| 9.56 | 192.75 | 200.93 | 215.34 | 9.56 | 0.0037 | 0.00 | -0.01 |
| 10.56 | 192.12 | 204.64 | 220.22 | 10.56 | 0.0004 | 0.02 | 0.01 |
| 11.56 | 193.93 | 202.72 | 216.40 | 11.56 | 0.0098 | 0.01 | 0.00 |
| 12.56 | 195.51 | 203.88 | 215.05 | 12.56 | 0.0180 | 0.01 | -0.01 |
| 13.56 | 190.86 | 199.74 | 219.65 | 13.56 | -0.0062 | -0.01 | 0.01 |
| 14.56 | 193.82 | 201.16 | 219.15 | 14.56 | 0.0092 | 0.00 | 0.01 |
| 15.56 | 192.52 | 201.21 | 218.99 | 15.56 | 0.0025 | 0.00 | 0.01 |
| 16.58 | 194.31 | 206.65 | 218.19 | 16.58 | 0.0118 | 0.03 | 0.01 |
| 17.58 | 191.90 | 203.92 | 215.28 | 17.58 | -0.0008 | 0.01 | -0.01 |
| 18.58 | 192.90 | 205.62 | 218.20 | 18.58 | 0.0044 | 0.02 | 0.01 |
| 19.58 | 194.29 | 205.03 | 219.21 | 19.58 | 0.0117 | 0.02 | 0.01 |
| 20.58 | 193.33 | 199.23 | 213.79 | 20.58 | 0.0067 | -0.01 | -0.02 |
| 21.58 | 195.08 | 205.93 | 220.14 | 21.58 | 0.0158 | 0.02 | 0.01 |
| 22.58 | 191.41 | 200.02 | 214.46 | 22.58 | -0.0033 | 0.00 | -0.01 |
| 23.58 | 193.68 | 200.48 | 218.31 | 23.58 | 0.0085 | 0.00 | 0.01 |
| 24.58 | 194.37 | 202.04 | 216.78 | 24.58 | 0.0121 | 0.01 | 0.00 |
| 25.58 | 193.72 | 204.60 | 224.53 | 25.58 | 0.0087 | 0.02 | 0.03 |
| 26.58 | 193.15 | 202.50 | 218.40 | 26.58 | 0.0057 | 0.01 | 0.01 |
| 27.58 | 196.96 | 205.68 | 225.72 | 27.58 | 0.0256 | 0.02 | 0.04 |
| 28.56 | 196.41 | 206.18 | 222.70 | 28.56 | 0.0227 | 0.03 | 0.03 |
| 29.61 | 197.63 | 214.07 | 232.14 | 29.61 | 0.0291 | 0.07 | 0.07 |
| 30.56 | 197.37 | 214.73 | 233.73 | 30.56 | 0.0277 | 0.07 | 0.08 |
| 31.63 | 200.16 | 216.06 | 239.54 | 31.63 | 0.0423 | 0.08 | 0.10 |
| 32.63 | 205.21 | 226.00 | 248.35 | 32.63 | 0.0685 | 0.12 | 0.14 |
| 33.63 | 205.44 | 229.69 | 255.68 | 33.63 | 0.0697 | 0.14 | 0.18 |
| 34.63 | 208.58 | 235.80 | 258.39 | 34.63 | 0.0861 | 0.17 | 0.19 |
| 35.63 | 214.48 | 243.72 | 267.89 | 35.63 | 0.1168 | 0.21 | 0.23 |
| 36.63 | 217.09 | 254.31 | 275.44 | 36.63 | 0.1304 | 0.27 | 0.27 |
| 37.65 | 221.27 | 258.58 | 281.49 | 37.65 | 0.1522 | 0.29 | 0.30 |
| 38.65 | 226.15 | 267.06 | 289.00 | 38.65 | 0.1776 | 0.33 | 0.33 |
| 39.65 | 234.73 | 270.26 | 292.79 | 39.65 | 0.2223 | 0.34 | 0.35 |

| Parameter | E5 | E6 | E7 |
|---|---|---|---|
| a | 134.083800 | 88.231000 | 88.465500 |
| b | 4.369000 | 2.909300 | 2.854600 |
| x0 | 43.108700 | 35.687900 | 34.670500 |
| y0 | 192.045900 | 200.981800 | 217.047300 |
| R | 0.986443 | 0.990521 | 0.994262 |
| $CP_{(SDM)}$ | 37.35 | 31.86 | 30.91 |

| Well/ Cycle | Raw fluorescence data E5 | E6 | E7 | Well/ Cycle | E5 | E6 | E7 |
|---|---|---|---|---|---|---|---|
| 0.56 | 118.55 | 112.65 | 103.62 | 0.56 | 0.0055 | -0.02 | 0.01 |
| 1.56 | 116.56 | 115.68 | 101.83 | 1.56 | -0.0114 | 0.00 | -0.01 |
| 2.56 | 114.14 | 116.30 | 102.35 | 2.56 | -0.0319 | 0.01 | 0.00 |
| 3.56 | 115.77 | 116.34 | 102.39 | 3.56 | -0.0181 | 0.01 | 0.00 |
| 4.56 | 116.41 | 115.87 | 102.48 | 4.56 | -0.0127 | 0.00 | 0.00 |
| 5.56 | 117.67 | 114.82 | 103.20 | 5.56 | -0.0020 | -0.01 | 0.01 |
| 6.56 | 120.17 | 115.27 | 103.84 | 6.56 | 0.0192 | 0.00 | 0.01 |
| 7.56 | 117.97 | 117.56 | 104.38 | 7.56 | 0.0005 | 0.02 | 0.02 |
| 8.56 | 118.14 | 117.17 | 104.43 | 8.56 | 0.0020 | 0.01 | 0.02 |
| 9.56 | 117.39 | 117.28 | 104.09 | 9.56 | -0.0044 | 0.02 | 0.02 |
| 10.56 | 120.86 | 118.13 | 103.55 | 10.56 | 0.0251 | 0.02 | 0.01 |
| 11.56 | 119.92 | 117.84 | 104.01 | 11.56 | 0.0171 | 0.02 | 0.02 |
| 12.56 | 119.78 | 118.94 | 104.01 | 12.56 | 0.0159 | 0.03 | 0.02 |
| 13.56 | 120.52 | 117.05 | 103.18 | 13.56 | 0.0222 | 0.01 | 0.01 |
| 14.56 | 119.90 | 119.36 | 103.99 | 14.56 | 0.0169 | 0.03 | 0.02 |
| 15.56 | 119.68 | 119.10 | 104.92 | 15.56 | 0.0150 | 0.03 | 0.02 |
| 16.58 | 120.47 | 118.07 | 104.71 | 16.58 | 0.0217 | 0.02 | 0.02 |
| 17.58 | 121.71 | 119.14 | 104.91 | 17.58 | 0.0323 | 0.03 | 0.02 |
| 18.58 | 120.67 | 117.15 | 104.49 | 18.58 | 0.0234 | 0.01 | 0.02 |
| 19.58 | 120.93 | 116.21 | 104.18 | 19.58 | 0.0256 | 0.01 | 0.02 |
| 20.58 | 122.92 | 120.21 | 105.30 | 20.58 | 0.0425 | 0.04 | 0.03 |
| 21.58 | 124.38 | 118.55 | 105.83 | 21.58 | 0.0549 | 0.03 | 0.03 |
| 22.58 | 123.62 | 120.24 | 106.91 | 22.58 | 0.0485 | 0.04 | 0.04 |
| 23.58 | 126.80 | 120.15 | 108.37 | 23.58 | 0.0754 | 0.04 | 0.06 |
| 24.58 | 133.06 | 124.12 | 110.75 | 24.58 | 0.1285 | 0.07 | 0.08 |
| 25.58 | 136.82 | 132.64 | 116.55 | 25.58 | 0.1604 | 0.15 | 0.14 |
| 26.58 | 150.13 | 146.71 | 128.00 | 26.58 | 0.2733 | 0.27 | 0.25 |
| 27.58 | 167.40 | 165.49 | 143.20 | 27.58 | 0.4198 | 0.43 | 0.40 |
| 28.56 | 199.20 | 198.96 | 172.06 | 28.56 | 0.6895 | 0.72 | 0.68 |
| 29.61 | 238.57 | 240.27 | 204.21 | 29.61 | 1.0234 | 1.08 | 0.99 |
| 30.56 | 293.66 | 288.51 | 246.78 | 30.56 | 1.4906 | 1.50 | 1.41 |
| 31.63 | 340.11 | 335.18 | 288.48 | 31.63 | 1.8846 | 1.90 | 1.82 |
| 32.63 | 383.91 | 380.86 | 325.77 | 32.63 | 2.2561 | 2.30 | 2.18 |
| 33.63 | 440.57 | 440.99 | 372.60 | 33.63 | 2.7366 | 2.82 | 2.64 |
| 34.63 | 478.53 | 472.62 | 405.43 | 34.63 | 3.0586 | 3.09 | 2.96 |
| 35.63 | 518.12 | 500.80 | 423.17 | 35.63 | 3.3943 | 3.34 | 3.13 |
| 36.63 | 536.71 | 527.61 | 452.10 | 36.63 | 3.5520 | 3.57 | 3.41 |
| 37.65 | 558.69 | 552.13 | 469.96 | 37.65 | 3.7384 | 3.78 | 3.59 |
| 38.65 | 582.22 | 565.58 | 486.82 | 38.65 | 3.9380 | 3.90 | 3.75 |
| 39.65 | 595.76 | 584.24 | 504.11 | 39.65 | 4.0528 | 4.06 | 3.92 |
| Parameter | | | | | | | |
| a | 485.580000 | 471.534000 | 403.567700 | | | | |
| b | 2.264800 | 2.222500 | 2.264800 | | | | |
| x0 | 32.119000 | 31.990900 | 32.114000 | | | | |
| y0 | 117.906200 | 115.520700 | 102.440900 | | | | |
| R | 0.999647 | 0.999564 | 0.999487 | | | | |
| **$CP_{(SDM)}$** | 29.14 | 29.06 | 29.13 | | | | |

| E5 | | | E6 | |
|---|---|---|---|---|
| Well/ Cycle | FAM Normalized Fluorescence | HEX Normalized Fluorescence | Well/ Cycle | FAM Normalized Fluorescence |
| 0.56 | -0.036272058 | 0.005460273 | 0.56 | -0.039365754 |
| 1.56 | -0.009611765 | -0.011417551 | 1.56 | -0.023991227 |
| 2.56 | -0.004769172 | -0.031942341 | 2.56 | -0.007820609 |
| 3.56 | -0.00992419 | -0.018117792 | 3.56 | -0.014388368 |
| 4.56 | 0.01184144 | -0.012689748 | 4.56 | -0.004387462 |
| 5.56 | 0.005749146 | -0.002003287 | 5.56 | -0.024936586 |
| 6.56 | 0.000437916 | 0.019200008 | 6.56 | 0.003772481 |
| 7.56 | -0.00523781 | 0.000541108 | 7.56 | -0.000506514 |
| 8.56 | 0.004551516 | 0.001982932 | 8.56 | 0.010937309 |
| 9.56 | 0.003666311 | -0.004378056 | 9.56 | -0.000257735 |
| 10.56 | 0.000385845 | 0.025052118 | 10.56 | 0.018201648 |
| 11.56 | 0.009810675 | 0.017079679 | 11.56 | 0.008648544 |
| 12.56 | 0.018037875 | 0.015892294 | 12.56 | 0.014420211 |
| 13.56 | -0.006175086 | 0.02216847 | 13.56 | -0.006178669 |
| 14.56 | 0.009237896 | 0.016910052 | 14.56 | 0.000886647 |
| 15.56 | 0.002468681 | 0.015044162 | 15.56 | 0.001135426 |
| 16.58 | 0.011789369 | 0.021744404 | 16.58 | 0.028202554 |
| 17.58 | -0.000759714 | 0.032261238 | 17.58 | 0.014619234 |
| 18.58 | 0.004447374 | 0.023440667 | 18.58 | 0.023077712 |
| 19.58 | 0.011685227 | 0.02564581 | 19.58 | 0.020142122 |
| 20.58 | 0.006686422 | 0.042523633 | 20.58 | -0.008716212 |
| 21.58 | 0.015798827 | 0.054906358 | 21.58 | 0.02462014 |
| 22.58 | -0.003311188 | 0.048460556 | 22.58 | -0.004785508 |
| 23.58 | 0.008508903 | 0.075431148 | 23.58 | -0.002496743 |
| 24.58 | 0.012101794 | 0.1285242 | 24.58 | 0.005265153 |
| 25.58 | 0.008717187 | 0.160413956 | 25.58 | 0.018002625 |
| 26.58 | 0.005749146 | 0.273300301 | 26.58 | 0.007553918 |
| 27.58 | 0.025588154 | 0.419772667 | 27.58 | 0.023376246 |
| 28.56 | 0.022724255 | 0.689478586 | 28.56 | 0.025864033 |
| 29.61 | 0.029076903 | 1.023388083 | 29.61 | 0.065121319 |
| 30.56 | 0.02772306 | 1.490623903 | 30.56 | 0.068405199 |
| 31.63 | 0.042250837 | 1.884581133 | 31.63 | 0.075022713 |
| 32.63 | 0.068546634 | 2.25606287 | 32.63 | 0.124479928 |
| 33.63 | 0.069744264 | 2.73661436 | 33.63 | 0.142839799 |
| 34.63 | 0.086094522 | 3.058565198 | 34.63 | 0.173240562 |
| 35.63 | 0.116816344 | 3.394340586 | 35.63 | 0.212647115 |
| 36.63 | 0.130406845 | 3.552008291 | 36.63 | 0.265338454 |
| 37.65 | 0.152172475 | 3.738427665 | 37.65 | 0.286584158 |
| 38.65 | 0.177583067 | 3.937993083 | 38.65 | 0.328777034 |
| 39.65 | 0.222259887 | 4.052830131 | 39.65 | 0.344698873 |

| Experiment derived parameter | linear regression value |
|---|---|
| y0 | 0.0179 |
| a | 0.0105 |
| R | 0.82758174 |

| Experiment derived parameter | linear regression value |
|---|---|
| y0 | 0.0078 |
| a | 0.0388 |
| R | 0.91826165 |

| HEX Normalized Fluorescence | E7 Well/ Cycle | E7 FAM Normalized Fluorescence | E7 HEX Normalized Fluorescence |
|---|---|---|---|
| -0.024850092 | 0.56 | -0.027447013 | 0.011510051 |
| 0.001378974 | 1.56 | -0.015421984 | -0.005963438 |
| 0.006745977 | 2.56 | 0.011254229 | -0.000887341 |
| 0.007092235 | 3.56 | -0.004640924 | -0.000496872 |
| 0.003023701 | 4.56 | 0.012728562 | 0.000381683 |
| -0.00606558 | 5.56 | 0.012866781 | 0.007410126 |
| -0.002170174 | 6.56 | 0.010102406 | 0.013657631 |
| 0.017653113 | 7.56 | 0.00176321 | 0.018928963 |
| 0.014277095 | 8.56 | 0.000703533 | 0.019417049 |
| 0.015229305 | 9.56 | -0.007866027 | 0.016098062 |
| 0.022587294 | 10.56 | 0.014617551 | 0.01082673 |
| 0.020076921 | 11.56 | -0.002982299 | 0.015317124 |
| 0.029599024 | 12.56 | -0.009202142 | 0.015317124 |
| 0.01323832 | 13.56 | 0.011991395 | 0.007214892 |
| 0.033234736 | 14.56 | 0.00968775 | 0.01512189 |
| 0.030984057 | 15.56 | 0.008950584 | 0.024200295 |
| 0.022067906 | 16.58 | 0.005264751 | 0.022150333 |
| 0.031330316 | 17.58 | -0.008142465 | 0.024102678 |
| 0.014103966 | 18.58 | 0.005310824 | 0.020002753 |
| 0.005966896 | 19.58 | 0.009964188 | 0.016976618 |
| 0.040592725 | 20.58 | -0.015007328 | 0.027909751 |
| 0.026223006 | 21.58 | 0.014248968 | 0.033083466 |
| 0.040852419 | 22.58 | -0.011920443 | 0.04362613 |
| 0.040073338 | 23.58 | 0.005817626 | 0.05787825 |
| 0.074439473 | 24.58 | -0.001231529 | 0.081111158 |
| 0.148192488 | 25.58 | 0.034474974 | 0.137729169 |
| 0.269988842 | 26.58 | 0.006232282 | 0.249500932 |
| 0.432557109 | 27.58 | 0.03995765 | 0.397879167 |
| 0.722288733 | 28.56 | 0.026043632 | 0.679602581 |
| 1.079886981 | 29.61 | 0.069536456 | 0.993442072 |
| 1.497474479 | 30.56 | 0.076862048 | 1.40899875 |
| 1.901471338 | 31.63 | 0.103630407 | 1.816062725 |
| 2.296898305 | 32.63 | 0.144220638 | 2.180077489 |
| 2.817411079 | 33.63 | 0.177992078 | 2.637219119 |
| 3.091214821 | 34.63 | 0.190477836 | 2.957696584 |
| 3.335153786 | 35.63 | 0.234247097 | 3.130869604 |
| 3.567233405 | 36.63 | 0.269032142 | 3.413276338 |
| 3.779489736 | 37.65 | 0.29690625 | 3.587620765 |
| 3.895919086 | 38.65 | 0.331507003 | 3.752203466 |
| 4.057448578 | 39.65 | 0.348968635 | 3.920983709 |

| **Experiment derived parameter** | linear regression **value** |
|---|---|
| y0 | 0.0099 |
| a | 0.0503 |
| **R** | **0.92575888** |

| **AVERAGE** | value |
|---|---|
| y0 | 0.011866667 |
| **a** | 0.0332 |
