# Supplementary material for: Quantify single nucleotide polymorphism (SNP) ratio in pooled DNA based on normalized fluorescence real-time PCR
Source: BMC Genomics. 2006 Jun 9;7:143. doi: 10.1186/1471-2164-7-143 (PMC1552069; doi:10.1186/1471-2164-7-143)
Supplement: Additional file 2 — Contained the raw and analytical datas used during the procession. contain the ratios of normalized fluorescence for all samples. [file 1471-2164-7-143-S2.pdf]

Well/ Cycle	Raw fluorescence data A7	A8	A9	B2
0.56	175.18	154.04	165.20	146.22
1.56	180.55	162.86	167.08	148.78
2.56	187.15	160.67	173.58	152.21
3.56	185.63	161.94	175.26	149.66
4.56	191.35	164.88	177.39	151.00
5.56	185.42	166.32	177.89	152.11
6.56	192.56	169.05	178.63	151.42
7.56	191.28	167.48	179.90	150.17
8.56	195.24	169.30	179.28	152.45
9.56	191.05	169.20	181.29	151.80
10.56	193.92	172.19	184.34	152.97
11.56	193.81	169.17	182.93	151.38
12.56	192.21	168.79	185.63	152.70
13.56	190.37	165.97	180.94	152.59
14.56	193.72	172.65	185.34	153.10
15.56	190.91	174.68	185.00	150.94
16.58	191.51	172.14	188.18	151.10
17.58	194.22	173.82	184.55	149.10
18.58	193.02	174.01	188.26	154.42
19.58	190.24	175.12	185.16	149.77
20.58	193.94	174.47	183.27	153.07
21.58	191.95	172.51	182.94	149.16
22.58	192.87	173.43	191.49	150.94
23.58	188.83	172.75	188.28	149.58
24.58	194.28	177.32	193.29	153.13
25.58	191.89	174.96	187.19	152.07
26.58	204.70	183.03	200.53	155.68
27.58	209.56	190.39	203.39	161.05
28.56	227.95	208.22	226.79	174.12
29.61	251.54	225.14	243.86	184.59
30.56	282.57	253.18	278.01	204.41
31.63	310.58	271.81	302.05	215.32
32.63	354.06	308.38	341.26	243.90
33.63	379.22	331.44	363.20	256.30
34.63	412.38	359.02	393.22	274.46
35.63	452.41	384.14	426.39	296.48
36.63	475.35	404.27	443.21	318.11
37.65	507.67	429.80	469.64	333.19
38.65	525.19	444.60	489.01	345.32
39.65	545.32	461.71	506.71	359.48
Parameter				
a	378.609800	315.128600	348.626200	225.954100
b	2.443600	2.607400	2.614400	2.519300
x0	33.557600	33.447500	33.314200	33.909800
y0	188.679700	167.557400	179.587500	150.126500
R	0.998780	0.998642	0.998388	0.998671
$CP_{(SDM)}$	30.34	30.01	29.87	30.59

Well/ Cycle	A7	A8	A9	B2
0.56	-0.0715	-0.08	-0.08	-0.03
1.56	-0.0431	-0.03	-0.07	-0.01
2.56	-0.0081	-0.04	-0.03	0.01
3.56	-0.0162	-0.03	-0.02	0.00
4.56	0.0142	-0.02	-0.01	0.01
5.56	-0.0173	-0.01	-0.01	0.01
6.56	0.0206	0.01	-0.01	0.01
7.56	0.0138	0.00	0.00	0.00
8.56	0.0348	0.01	0.00	0.02
9.56	0.0126	0.01	0.01	0.01
10.56	0.0278	0.03	0.03	0.02
11.56	0.0272	0.01	0.02	0.01
12.56	0.0187	0.01	0.03	0.02
13.56	0.0090	-0.01	0.01	0.02
14.56	0.0267	0.03	0.03	0.02
15.56	0.0118	0.04	0.03	0.01
16.58	0.0150	0.03	0.05	0.01
17.58	0.0294	0.04	0.03	-0.01
18.58	0.0230	0.04	0.05	0.03
19.58	0.0083	0.05	0.03	0.00
20.58	0.0279	0.04	0.02	0.02
21.58	0.0173	0.03	0.02	-0.01
22.58	0.0222	0.04	0.07	0.01
23.58	0.0008	0.03	0.05	0.00
24.58	0.0297	0.06	0.08	0.02
25.58	0.0170	0.04	0.04	0.01
26.58	0.0849	0.09	0.12	0.04
27.58	0.1107	0.14	0.13	0.07
28.56	0.2081	0.24	0.26	0.16
29.61	0.3332	0.34	0.36	0.23
30.56	0.4976	0.51	0.55	0.36
31.63	0.6461	0.62	0.68	0.43
32.63	0.8765	0.84	0.90	0.62
33.63	1.0099	0.98	1.02	0.71
34.63	1.1856	1.14	1.19	0.83
35.63	1.3978	1.29	1.37	0.97
36.63	1.5193	1.41	1.47	1.12
37.65	1.6906	1.57	1.62	1.22
38.65	1.7835	1.65	1.72	1.30
39.65	1.8902	1.76	1.82	1.39

Well/ Cycle	A7	A8	A9	B2	Well/ Cycle	A7	A8	A9	B2
						**Fluorescence Data Normalized To Y0**			
0.56	93.56	92.33	83.65	82.17	0.56	-0.06	-0.07	-0.08	-0.01
1.56	97.81	95.56	87.69	82.8	1.56	-0.01	-0.04	-0.04	0.00
2.56	96.9	95.93	86.4	82.3	2.56	-0.02	-0.03	-0.05	-0.01
3.56	99.47	100.12	88.03	83.11	3.56	0.00	0.01	-0.03	0.00
4.56	98.39	97.62	89.17	83.18	4.56	-0.01	-0.02	-0.02	0.00
5.56	99.66	98.29	90.79	83.79	5.56	0.01	-0.01	0.00	0.01
6.56	99.55	98.16	90.76	83.63	6.56	0.01	-0.01	0.00	0.01
7.56	99.09	99.46	91.24	83.88	7.56	0.00	0.00	0.00	0.01
8.56	100.34	98.92	91.12	83.24	8.56	0.01	0.00	0.00	0.00
9.56	99.04	101.53	91.56	84.14	9.56	0.00	0.02	0.01	0.01
10.56	102.19	100.09	91.94	83.92	10.56	0.03	0.01	0.01	0.01
11.56	99.66	100.66	92.24	83.22	11.56	0.01	0.01	0.01	0.00
12.56	99.44	101.25	93.81	84.33	12.56	0.00	0.02	0.03	0.01
13.56	100.23	101.05	94.73	83.17	13.56	0.01	0.02	0.04	0.00
14.56	98.8	98.76	94.41	83.42	14.56	0.00	-0.01	0.04	0.00
15.56	100.21	103.44	92.41	83.39	15.56	0.01	0.04	0.02	0.00
16.58	99.99	101.75	91.54	83.44	16.58	0.01	0.02	0.01	0.00
17.58	100.41	102.2	92.68	82.09	17.58	0.01	0.03	0.02	-0.01
18.58	99.18	103.18	94.87	83.41	18.58	0.00	0.04	0.04	0.00
19.58	100.25	102.53	91.66	82.99	19.58	0.01	0.03	0.01	0.00
20.58	98.93	103.52	95.23	82.83	20.58	0.00	0.04	0.05	0.00
21.58	101.02	102.89	95.03	83.48	21.58	0.02	0.04	0.04	0.00
22.58	101.58	102.68	96.94	82.27	22.58	0.03	0.03	0.07	-0.01
23.58	101.98	104.43	93.09	83.38	23.58	0.03	0.05	0.02	0.00
24.58	101.51	104.12	94.98	83.85	24.58	0.02	0.05	0.04	0.01
25.58	103.74	104.39	97.96	84.92	25.58	0.05	0.05	0.08	0.02
26.58	103.6	107.69	97.12	85.58	26.58	0.05	0.08	0.07	0.03
27.58	106.34	107.69	101.24	86.6	27.58	0.07	0.08	0.11	0.04
28.56	110.15	112.14	103.52	87.74	28.56	0.11	0.13	0.14	0.06
29.61	114.39	115.41	109.11	90.18	29.61	0.15	0.16	0.20	0.09
30.56	118.9	119.71	110.63	93.08	30.56	0.20	0.21	0.22	0.12
31.63	125.08	125.19	117.09	98.46	31.63	0.26	0.26	0.29	0.18
32.63	132.51	131.5	125.73	101.45	32.63	0.34	0.32	0.38	0.22
33.63	141.76	140.46	135.3	107.2	33.63	0.43	0.41	0.49	0.29
34.63	152.08	147.5	145.4	112.76	34.63	0.54	0.48	0.60	0.36
35.63	157.53	151.62	151.12	116.65	35.63	0.59	0.53	0.66	0.40
36.63	167.8	160.21	157.2	119.89	36.63	0.69	0.61	0.73	0.44
37.65	179.86	167.06	170.69	123.53	37.65	0.82	0.68	0.88	0.49
38.65	188.67	175.28	177.08	126.44	38.65	0.90	0.76	0.95	0.52
39.65	193.48	182.66	186.44	131.93	39.65	0.95	0.84	1.05	0.59
Parameter									
a	120.27	117.12	130.7	51.865					
b	3.0633	3.7963	3.5324	2.533					
x0	35.561	36.303	36.204	34.098					
y0	99.043	99.339	90.955	83.098					
R	0.9987	0.9964	0.9964	0.9986					
**$CP_{(SDM)}$**	31.53	31.30	31.55	30.76					

A8			A9		
Well/ Cycle	**FAM** Normalized Fluorescence	VIC Normalized Fluorescence	Well/ Cycle	**FAM** Normalized Fluorescence	VIC Normalized Fluorescence
0.56	-0.08067325	-0.070554506	0.56	-0.08011415	-0.080314441
1.56	-0.028034572	-0.038039517	1.56	-0.069645716	-0.035896872
2.56	-0.04110472	-0.03431489	2.56	-0.03345166	-0.05007971
3.56	-0.033525228	0.007863997	3.56	-0.024096889	-0.03215876
4.56	-0.015979002	-0.017302403	4.56	-0.012236375	-0.019625089
5.56	-0.007384932	-0.010557808	5.56	-0.009452217	-0.001814084
6.56	0.008907992	-0.011866461	6.56	-0.005331663	-0.002143917
7.56	-0.000461931	0.001220067	7.56	0.001740099	0.003133418
8.56	0.010400018	-0.004215875	8.56	-0.001712257	0.001814084
9.56	0.009803208	0.022057846	9.56	0.009480058	0.006651641
10.56	0.027647839	0.007562	10.56	0.026463423	0.010829531
11.56	0.009624165	0.013299939	11.56	0.018612097	0.014127865
12.56	0.007356285	0.01923921	12.56	0.033646551	0.031389148
13.56	-0.009473768	0.017225898	13.56	0.007531148	0.04150404
14.56	0.030393167	-0.005826525	14.56	0.032031739	0.037985817
15.56	0.042508418	0.041284976	15.56	0.030138512	0.015996922
16.58	0.027349434	0.02427249	16.58	0.047845758	0.006431752
17.58	0.037375849	0.028802442	17.58	0.02763277	0.018965422
18.58	0.038509788	0.038667671	18.58	0.048291223	0.043043263
19.58	0.045134384	0.032124407	19.58	0.031029442	0.007751086
20.58	0.041255116	0.042090301	20.58	0.020505325	0.047001264
21.58	0.029557632	0.035748368	21.58	0.01866778	0.044802375
22.58	0.035048288	0.033634391	22.58	0.066276885	0.06580177
23.58	0.030989977	0.051250871	23.58	0.048402589	0.023473146
24.58	0.058264213	0.048130237	24.58	0.076299854	0.044252652
25.58	0.044179487	0.050848208	25.58	0.042333125	0.077016107
26.58	0.092342087	0.084067857	26.58	0.116614464	0.067780771
27.58	0.136267333	0.084067857	27.58	0.132539848	0.113077896
28.56	0.242678628	0.128864049	28.56	0.262838449	0.138145237
29.61	0.343658949	0.161781701	29.61	0.357889608	0.1996042
30.56	0.511004587	0.205067909	30.56	0.548047609	0.216315761
31.63	0.622190366	0.260232658	31.63	0.681909932	0.287339893
32.63	0.840443931	0.323752653	32.63	0.900243614	0.382331922
33.63	0.978068411	0.413949031	33.63	1.022412473	0.487548788
34.63	1.142668721	0.484817614	34.63	1.189573328	0.598592711
35.63	1.292587495	0.526291842	35.63	1.374274379	0.661480952
36.63	1.41272543	0.612763593	36.63	1.467933459	0.728327195
37.65	1.565091127	0.68171953	37.65	1.615104058	0.876642296
38.65	1.653419067	0.764466654	38.65	1.722962344	0.946896817
39.65	1.755533328	0.838757867	39.65	1.821521542	1.049804849

**periment derived** linear regress	
**parameter**	**value**
y0	-0.1386
a	3.0123
**R**	**0.9970893**

**Experiment derived** linear regression	
**parameter**	**value**
y0	-0.1084
a	2.6762
**R**	**0.98346659**

B2

Well/ Cycle	**FAM** Normalized Fluorescence	VIC Normalized Fluorescence
0.56	-0.026021389	-0.011167537
1.56	-0.008969103	-0.003586127
2.56	0.013878296	-0.009603119
3.56	-0.003107379	0.000144408
4.56	0.005818426	0.000986787
5.56	0.013212191	0.008327517
6.56	0.008616067	0.006402079
7.56	0.000289756	0.009410575
8.56	0.015476948	0.001708826
9.56	0.011147266	0.012539411
10.56	0.018940693	0.009891935
11.56	0.008349625	0.001468146
12.56	0.01714221	0.014825868
13.56	0.016409495	0.000866447
14.56	0.01980663	0.003874943
15.56	0.005418764	0.003513923
16.58	0.006484531	0.004115623
17.58	-0.006837567	-0.012130256
18.58	0.028599215	0.003754603
19.58	-0.002374664	-0.00129967
20.58	0.019606798	-0.003225108
21.58	-0.006437904	0.004596982
22.58	0.005418764	-0.009964139
23.58	-0.003640263	0.003393583
24.58	0.020006461	0.009049556
25.58	0.012945749	0.021925919
26.58	0.036992137	0.029868348
27.58	0.072761971	0.042143012
28.56	0.159821884	0.055861754
29.61	0.229563068	0.085224674
30.56	0.361585063	0.120123228
31.63	0.43425711	0.184866062
32.63	0.624629895	0.220847674
33.63	0.707226905	0.290043082
34.63	0.828191558	0.356952033
35.63	0.974867861	0.40376423
36.63	1.118946355	0.442754338
37.65	1.219394977	0.48655804
38.65	1.300193503	0.521576933
39.65	1.39451396	0.587643505

**periment derived**	linear regress
**parameter**	**value**
y0	-0.0242
a	2.8275
**R**	**0.98357906**

AVERAGE		**value**
	y0	-0.088225
	a	2.82955
	**R**	

Well/	Raw fluorescence data				Well/				
Cycle	**B4**	**B5**	**B6**	**B8**	Cycle	B4	B5	B6	B8
0.56	175.59	159.74	168.86	164.05	0.56	-0.0205	-0.05	-0.04	-0.04
1.56	179.13	161.81	172.04	167.75	1.56	-0.0007	-0.04	-0.02	-0.02
2.56	183.65	166.73	175.03	170.58	2.56	0.0245	-0.01	0.00	-0.01
3.56	179.46	164.12	173.61	168.52	3.56	0.0011	-0.02	-0.01	-0.02
4.56	185.10	168.13	177.61	172.24	4.56	0.0326	0.00	0.01	0.00
5.56	182.77	166.71	175.22	167.97	5.56	0.0196	-0.01	0.00	-0.02
6.56	181.98	170.30	178.95	172.66	6.56	0.0151	0.01	0.02	0.01
7.56	182.89	168.17	177.04	178.44	7.56	0.0202	0.00	0.01	0.04
8.56	183.26	170.83	178.11	173.34	8.56	0.0223	0.02	0.02	0.01
9.56	181.05	168.01	176.98	174.41	9.56	0.0100	0.00	0.01	0.02
10.56	184.90	169.60	178.12	173.01	10.56	0.0314	0.01	0.02	0.01
11.56	179.71	170.73	177.75	173.32	11.56	0.0025	0.02	0.02	0.01
12.56	182.45	170.06	178.68	174.42	12.56	0.0178	0.01	0.02	0.02
13.56	182.55	171.05	176.92	171.51	13.56	0.0183	0.02	0.01	0.00
14.56	183.93	171.79	177.74	176.53	14.56	0.0260	0.02	0.01	0.03
15.56	180.18	168.41	177.42	171.97	15.56	0.0051	0.00	0.01	0.00
16.58	182.42	172.24	178.23	175.68	16.58	0.0176	0.03	0.02	0.02
17.58	177.29	169.16	175.39	172.46	17.58	-0.0110	0.01	0.00	0.00
18.58	181.30	171.97	176.81	175.24	18.58	0.0114	0.02	0.01	0.02
19.58	176.51	171.60	174.98	176.20	19.58	-0.0154	0.02	0.00	0.03
20.58	179.55	172.23	174.92	174.35	20.58	0.0016	0.03	0.00	0.02
21.58	177.81	169.88	177.67	176.76	21.58	-0.0081	0.01	0.01	0.03
22.58	176.65	170.34	178.79	178.86	22.58	-0.0146	0.01	0.02	0.04
23.58	179.05	172.41	179.81	175.31	23.58	-0.0012	0.03	0.03	0.02
24.58	180.85	173.60	180.63	178.75	24.58	0.0088	0.03	0.03	0.04
25.58	179.41	173.29	180.78	179.23	25.58	0.0008	0.03	0.03	0.04
26.58	188.44	179.30	185.21	179.93	26.58	0.0512	0.07	0.06	0.05
27.58	191.14	183.22	190.31	187.67	27.58	0.0662	0.09	0.09	0.09
28.56	210.00	194.93	206.41	206.32	28.56	0.1715	0.16	0.18	0.20
29.61	227.52	208.93	224.23	220.17	29.61	0.2692	0.24	0.28	0.28
30.56	254.58	229.98	254.32	245.62	30.56	0.4201	0.37	0.45	0.43
31.63	274.68	246.65	271.49	267.31	31.63	0.5323	0.47	0.55	0.56
32.63	306.63	275.34	306.85	296.26	32.63	0.7105	0.64	0.75	0.73
33.63	326.50	293.20	323.69	315.19	33.63	0.8213	0.75	0.85	0.84
34.63	349.95	313.98	347.36	341.08	34.63	0.9521	0.87	0.98	0.99
35.63	379.65	333.14	376.41	365.01	35.63	1.1178	0.98	1.15	1.13
36.63	397.53	351.94	397.26	382.36	36.63	1.2176	1.10	1.27	1.23
37.65	423.00	370.93	419.50	408.17	37.65	1.3596	1.21	1.40	1.38
38.65	441.05	389.38	435.44	423.92	38.65	1.4603	1.32	1.49	1.47
39.65	461.47	400.13	451.98	438.36	39.65	1.5742	1.38	1.58	1.55
Parameter									
a	296.948200	251.330500	293.646300	285.991000					
b	2.479400	2.593700	2.506000	2.574600					
x0	33.692300	33.724100	33.535900	33.594200					
y0	179.264200	167.853500	175.121100	171.736700					
R	0.998081	0.998774	0.998724	0.998700					
**CP(SDM)**	30.43	30.31	30.24	30.20					

Well/ Cycle	B4	B5	B6	B8
0.56	86.9	87.98	94.48	87.47
1.56	88.33	90.19	97.31	89.05
2.56	88.63	90.34	95.59	88.9
3.56	88.29	90.53	96.29	89.03
4.56	88.31	89.94	96.79	89.39
5.56	87.48	92.6	97.16	90
6.56	88.35	89.88	97.18	91.09
7.56	88.34	91.69	97.04	90.99
8.56	88.58	92.66	98.27	90.69
9.56	89.01	90.98	97.24	91.94
10.56	88.37	92.12	97.27	90.62
11.56	89.08	91.2	97.66	92.04
12.56	89.33	90.94	97.37	91.8
13.56	87.25	91.21	97.52	92.36
14.56	88.3	91.9	99.23	92.28
15.56	87.84	91.11	98.21	90.47
16.58	86.89	91.06	96.91	89.01
17.58	87.48	91.96	97.83	91.69
18.58	87.72	91.82	97.55	91.59
19.58	87.88	91.49	97.82	91.93
20.58	86.53	91.14	97.2	92.16
21.58	87.67	91.67	98.99	92.62
22.58	86.78	93.55	98.35	90.94
23.58	88.8	94.15	99.21	93.67
24.58	87.95	93.51	99.67	93.64
25.58	89.3	94.44	100.97	93.86
26.58	91.45	97.99	101.02	97.68
27.58	92.45	99.08	105.29	99.29
28.56	98.13	101.14	108.13	101.77
29.61	103.18	109.13	116.16	107.08
30.56	110.8	112.82	121.11	111.22
31.63	123.83	122.87	133.88	119.91
32.63	134.38	129.28	144.85	128.74
33.63	147.62	139.72	155.82	142.14
34.63	160.25	150.45	169.15	150.51
35.63	169.43	157.2	179.76	161.65
36.63	183.97	165.27	192.72	166.55
37.65	191.38	175.69	204.77	180.31
38.65	202.69	182.09	216.14	187.43
39.65	214.58	188.06	226.18	198.34
a	137.35	111.32	149.57	127.16
b	2.4762	2.8197	2.7386	2.9086
x0	34.43	34.408	34.9	35.061
y0	87.487	90.907	96.913	90.469
R	0.9989	0.9993	0.9994	0.9988
**$CP_{(SDM)}$**	31.17	30.69	31.29	31.23

Well/ Cycle	**luorescence Data Normalized To Y** B4	B5	B6	B8
0.56	-0.01	-0.03	-0.03	-0.03
1.56	0.01	-0.01	0.00	-0.02
2.56	0.01	-0.01	-0.01	-0.02
3.56	0.01	0.00	-0.01	-0.02
4.56	0.01	-0.01	0.00	-0.01
5.56	0.00	0.02	0.00	-0.01
6.56	0.01	-0.01	0.00	0.01
7.56	0.01	0.01	0.00	0.01
8.56	0.01	0.02	0.01	0.00
9.56	0.02	0.00	0.00	0.02
10.56	0.01	0.01	0.00	0.00
11.56	0.02	0.00	0.01	0.02
12.56	0.02	0.00	0.00	0.01
13.56	0.00	0.00	0.01	0.02
14.56	0.01	0.01	0.02	0.02
15.56	0.00	0.00	0.01	0.00
16.58	-0.01	0.00	0.00	-0.02
17.58	0.00	0.01	0.01	0.01
18.58	0.00	0.01	0.01	0.01
19.58	0.00	0.01	0.01	0.02
20.58	-0.01	0.00	0.00	0.02
21.58	0.00	0.01	0.02	0.02
22.58	-0.01	0.03	0.01	0.01
23.58	0.02	0.04	0.02	0.04
24.58	0.01	0.03	0.03	0.04
25.58	0.02	0.04	0.04	0.04
26.58	0.05	0.08	0.04	0.08
27.58	0.06	0.09	0.09	0.10
28.56	0.12	0.11	0.12	0.12
29.61	0.18	0.20	0.20	0.18
30.56	0.27	0.24	0.25	0.23
31.63	0.42	0.35	0.38	0.33
32.63	0.54	0.42	0.49	0.42
33.63	0.69	0.54	0.61	0.57
34.63	0.83	0.65	0.75	0.66
35.63	0.94	0.73	0.85	0.79
36.63	1.10	0.82	0.99	0.84
37.65	1.19	0.93	1.11	0.99
38.65	1.32	1.00	1.23	1.07
39.65	1.45	1.07	1.33	1.19

B4			B5		
Well/ Cycle	**FAM** Normalized Fluorescence	VIC Normalized Fluorescence	Well/ Cycle	**FAM** Normalized Fluorescence	VIC Normalized Fluorescence
0.56	-0.020496005	-0.006711839	0.56	-0.048336794	-0.032193482
1.56	-0.000748616	0.009633409	1.56	-0.036004611	-0.007882816
2.56	0.024465565	0.013062482	2.56	-0.006693337	-0.006232771
3.56	0.001092243	0.009176199	3.56	-0.02224261	-0.004142714
4.56	0.032554185	0.009404804	4.56	0.00164727	-0.010632891
5.56	0.01955661	-8.22978E-05	5.56	-0.006812488	0.01862791
6.56	0.015149706	0.009862014	6.56	0.01457521	-0.011292909
7.56	0.020226013	0.009747712	7.56	0.001885573	0.008617636
8.56	0.022290005	0.01249097	8.56	0.017732725	0.019287928
9.56	0.009961833	0.017405975	9.56	0.000932361	0.000807422
10.56	0.031438514	0.010090619	10.56	0.010404907	0.013347766
11.56	0.002486832	0.018206092	11.56	0.017136968	0.003227488
12.56	0.017771535	0.021063653	12.56	0.013145392	0.00036741
13.56	0.018329371	-0.002711254	13.56	0.019043392	0.003337491
14.56	0.026027506	0.009290502	14.56	0.023451998	0.010927699
15.56	0.005108661	0.00403259	15.56	0.003315391	0.002237461
16.58	0.017604184	-0.006826141	16.58	0.026132908	0.001687446
17.58	-0.011012796	-8.22978E-05	17.58	0.007783573	0.011587718
18.58	0.011356423	0.002660961	18.58	0.024524362	0.010047675
19.58	-0.015363915	0.0044898	19.58	0.022320059	0.006417576
20.58	0.001594295	-0.010941029	20.58	0.026073332	0.00256747
21.58	-0.008112049	0.002089449	21.58	0.012073028	0.00839763
22.58	-0.014582945	-0.008083468	22.58	0.014813513	0.029078197
23.58	-0.001194884	0.015005624	23.58	0.027145696	0.035678378
24.58	0.008846161	0.005289917	24.58	0.034235211	0.028638185
25.58	0.000813325	0.020720745	25.58	0.032388362	0.038868465
26.58	0.051185903	0.045295769	26.58	0.068193395	0.077919535
27.58	0.066247472	0.056726012	27.58	0.091547093	0.089909864
28.56	0.171455316	0.121649796	28.56	0.161310309	0.112570484
29.61	0.269188159	0.179372525	29.61	0.244716375	0.200462893
30.56	0.420138544	0.266470981	30.56	0.370123352	0.241054005
31.63	0.532263553	0.415407054	31.63	0.469436145	0.351607034
32.63	0.710492112	0.535996123	32.63	0.640359004	0.422118966
33.63	0.821334098	0.687332547	33.63	0.746761313	0.536962113
34.63	0.952146608	0.831696522	34.63	0.870559744	0.654995347
35.63	1.11782386	0.936626158	35.63	0.984706902	0.729247381
36.63	1.217564913	1.102821899	36.63	1.096709333	0.818019814
37.65	1.359645707	1.187520003	37.65	1.209843703	0.932642954
38.65	1.460335081	1.316796057	38.65	1.319760982	1.003044883
39.65	1.574245164	1.452701652	39.65	1.383804925	1.068716683

**Experiment derived parameter**	linear regression **value**	**periment derived parameter**	linear regress **value**
y0	0.011	y0	-0.0426
a	1.3227	a	1.5671
**R**	**0.99276711**	**R**	**0.99092575**

B6			B8		
Well/ Cycle	**FAM** Normalized Fluorescence	VIC Normalized Fluorescence	Well/ Cycle	**FAM** Normalized Fluorescence	VIC Normalized Fluorescence
0.56	-0.035752973	-0.025105997	0.56	-0.044758633	-0.033149477
1.56	-0.017594111	0.004095422	1.56	-0.023214025	-0.015684931
2.56	-0.000520211	-0.013652437	2.56	-0.006735311	-0.017342957
3.56	-0.008628886	-0.006429471	3.56	-0.018730417	-0.015906001
4.56	0.014212451	-0.00127021	4.56	0.002930649	-0.011926737
5.56	0.000564752	0.002547643	5.56	-0.021932994	-0.005184096
6.56	0.021864298	0.002754014	6.56	0.005376253	0.00686423
7.56	0.01095756	0.001309421	7.56	0.039032426	0.005758879
8.56	0.017067618	0.014001203	8.56	0.009335803	0.002442826
9.56	0.01061494	0.003373125	9.56	0.015566271	0.016259713
10.56	0.017124721	0.003682681	10.56	0.007414257	0.00166908
11.56	0.015011897	0.007706904	11.56	0.009219346	0.017365064
12.56	0.020322508	0.004714533	12.56	0.0156245	0.014712222
13.56	0.01027232	0.006262311	13.56	-0.001320044	0.020902187
14.56	0.014954794	0.023906985	14.56	0.027910749	0.020017907
15.56	0.013127487	0.013382092	15.56	0.001358475	1.10535E-05
16.58	0.017752858	-3.19874E-05	16.58	0.022961312	-0.016127071
17.58	0.001535509	0.009461053	17.58	0.00421168	0.013496336
18.58	0.009644183	0.006571867	18.58	0.020399251	0.012390985
19.58	-0.000805728	0.009357868	19.58	0.025989203	0.016149178
20.58	-0.001148348	0.002960384	20.58	0.015216899	0.018691485
21.58	0.014555071	0.021430539	21.58	0.029250009	0.0237761
22.58	0.020950645	0.014826685	22.58	0.04147803	0.005206203
23.58	0.026775186	0.023700614	23.58	0.020806851	0.035382286
24.58	0.03145766	0.028447135	24.58	0.040837515	0.03505068
25.58	0.03231421	0.041861214	25.58	0.043632491	0.037482453
26.58	0.05761099	0.04237714	26.58	0.047708498	0.079706861
27.58	0.086733695	0.086437231	27.58	0.09277749	0.097503012
28.56	0.178670075	0.115741835	28.56	0.201373964	0.124915717
29.61	0.280428229	0.19859957	29.61	0.282020675	0.183609855
30.56	0.452252184	0.249676256	30.56	0.430212645	0.229371387
31.63	0.550298622	0.381443788	31.63	0.556510635	0.325426389
32.63	0.752216038	0.49463798	32.63	0.725082641	0.423028883
33.63	0.848378065	0.607832171	33.63	0.835309517	0.571145917
34.63	0.983541675	0.745378076	34.63	0.98606355	0.663663796
35.63	1.149426882	0.854857599	35.63	1.125404762	0.786799898
36.63	1.26848735	0.98858565	36.63	1.226431508	0.840962098
37.65	1.395485181	1.112923846	37.65	1.376719711	0.993058396
38.65	1.486507908	1.230245447	38.65	1.46842987	1.071759387
39.65	1.580956835	1.333843412	39.65	1.552512072	1.192353182

**periment derived**	linear regress		**periment derived**	linear regress	
**parameter**	**value**		**parameter**	**value**	
y0	-0.0106		y0	-0.073	0.0281
a	1.5504		a	1.9448	0.117
**R**	**0.98942526**		**R**	**0.99106621**	

AVERAGE	**value**
y0	-0.0288
a	1.59625
**R**	

Well/	Raw fluorescence data				Well/				
Cycle	**B9**	**C3**	**C4**	**C5**	Cycle	B9	C3	C4	C5
0.56	192.55	165.46	190.28	157.44	0.56	-0.0149	-0.02	-0.04	-0.03
1.56	191.71	169.21	195.87	156.54	1.56	-0.0192	0.01	-0.01	-0.04
2.56	198.93	169.48	199.10	161.99	2.56	0.0177	0.01	0.00	-0.01
3.56	195.46	170.30	195.89	161.64	3.56	0.0000	0.01	-0.01	-0.01
4.56	202.43	169.98	199.70	163.49	4.56	0.0357	0.01	0.01	0.00
5.56	198.00	171.51	199.20	164.17	5.56	0.0130	0.02	0.00	0.01
6.56	197.12	174.03	203.97	165.38	6.56	0.0085	0.03	0.03	0.02
7.56	197.52	171.30	200.96	162.62	7.56	0.0105	0.02	0.01	0.00
8.56	199.19	171.12	204.95	163.88	8.56	0.0191	0.02	0.03	0.01
9.56	203.40	169.96	200.54	166.14	9.56	0.0406	0.01	0.01	0.02
10.56	198.46	171.50	203.23	166.64	10.56	0.0153	0.02	0.02	0.02
11.56	193.77	170.99	201.25	162.21	11.56	-0.0087	0.02	0.01	0.00
12.56	199.77	171.93	203.99	165.93	12.56	0.0220	0.02	0.03	0.02
13.56	195.64	167.45	198.47	163.96	13.56	0.0009	-0.01	0.00	0.01
14.56	198.76	170.86	203.94	164.89	14.56	0.0169	0.01	0.03	0.01
15.56	198.92	167.50	198.94	164.73	15.56	0.0177	-0.01	0.00	0.01
16.58	198.47	170.44	200.85	166.17	16.58	0.0154	0.01	0.01	0.02
17.58	195.72	167.46	199.37	165.73	17.58	0.0013	-0.01	0.00	0.02
18.58	198.91	169.55	201.09	163.79	18.58	0.0176	0.01	0.01	0.01
19.58	189.55	168.33	198.29	163.92	19.58	-0.0302	0.00	0.00	0.01
20.58	196.81	169.42	198.29	163.11	20.58	0.0069	0.01	0.00	0.00
21.58	193.18	165.84	195.70	165.16	21.58	-0.0117	-0.01	-0.01	0.01
22.58	195.44	164.93	200.41	167.88	22.58	-0.0001	-0.02	0.01	0.03
23.58	193.78	165.07	199.57	163.20	23.58	-0.0086	-0.02	0.01	0.00
24.58	197.74	169.96	200.63	165.99	24.58	0.0117	0.01	0.01	0.02
25.58	198.16	168.60	198.11	167.22	25.58	0.0138	0.00	0.00	0.03
26.58	205.27	171.89	205.91	170.65	26.58	0.0502	0.02	0.04	0.05
27.58	213.23	177.48	210.43	174.06	27.58	0.0909	0.05	0.06	0.07
28.56	226.26	191.98	225.18	186.98	28.56	0.1576	0.14	0.14	0.15
29.61	241.65	198.78	239.70	195.07	29.61	0.2363	0.18	0.21	0.20
30.56	271.48	219.84	264.15	215.66	30.56	0.3889	0.31	0.33	0.32
31.63	289.45	231.06	285.79	229.79	31.63	0.4809	0.37	0.44	0.41
32.63	314.21	259.21	321.57	258.26	32.63	0.6075	0.54	0.62	0.59
33.63	338.58	271.92	341.15	272.45	33.63	0.7322	0.62	0.72	0.67
34.63	356.74	289.07	364.95	293.40	34.63	0.8251	0.72	0.84	0.80
35.63	388.73	311.35	394.72	315.96	35.63	0.9888	0.85	0.99	0.94
36.63	407.35	328.25	417.63	333.07	36.63	1.0840	0.95	1.11	1.05
37.65	428.90	346.35	436.17	354.70	37.65	1.1943	1.06	1.20	1.18
38.65	445.80	361.03	455.74	364.35	38.65	1.2808	1.14	1.30	1.24
39.65	464.40	370.92	476.69	378.99	39.65	1.3759	1.20	1.40	1.33
a	286.197600	217.443800	291.769100	233.357200					
b	2.542000	2.439100	2.391000	2.491100					
x0	33.698200	33.876400	33.780400	33.921400					
y0	195.461000	168.364300	198.392100	162.869400					
R	0.998224	0.998106	0.998393	0.998889					
**$CP_{(SDM)}$**	30.35	30.66	30.63	30.64					

Well/ Cycle	B9	C3	C4	C5
0.56	86.83	86.98	91.82	90.94
1.56	89.09	88.48	92.02	93.66
2.56	86.54	87.57	92.08	91.69
3.56	90.38	88.41	94.1	94.17
4.56	88.51	86.73	92.93	92.19
5.56	89.96	87.59	92.76	92.53
6.56	89.41	86.83	91.75	93.77
7.56	89.6	87.52	93.23	93.71
8.56	90.98	86.67	91.95	93.76
9.56	89.86	87.64	92.69	93.04
10.56	91.4	87.8	92.58	93.92
11.56	91.27	87.14	93.15	94.2
12.56	89.02	87.2	92.24	93.82
13.56	90.54	86.45	92.75	93.45
14.56	88.95	87.63	91.67	94.09
15.56	92.25	86.83	92.93	93.08
16.58	90.32	86.1	92.73	94.43
17.58	91.42	85.17	90.25	93.6
18.58	88.48	86.24	91.28	94.07
19.58	88.79	86.23	92.41	93.55
20.58	90.2	84.5	90.92	94.84
21.58	91.11	85.59	90.72	94.54
22.58	89.63	86.65	91.5	94.12
23.58	91.21	86.42	92.06	94.28
24.58	91.77	87.25	92.03	95.23
25.58	93.15	88.38	93.51	97.09
26.58	93.98	90.54	95.67	97.62
27.58	99.76	94.4	100.13	101.73
28.56	101.47	98.09	103.68	104.97
29.61	110.45	106.21	112.06	113.1
30.56	117.9	114.38	118.14	122.84
31.63	132.33	126.56	130.7	136.3
32.63	139.99	136.72	145.69	146.84
33.63	158.24	153.92	158.55	164.42
34.63	174.48	167.3	174.67	179.6
35.63	186.26	177.3	185.85	193.39
36.63	194.59	192.29	197.83	204.45
37.65	208.18	203.96	215.65	217.56
38.65	219.32	215.93	226.75	231.43
39.65	234.61	226.9	240.16	241.77
Parameter				
a	159.76	155.57	167.73	164.37
b	2.634	2.5538	2.616	2.5429
x0	34.524	34.527	34.839	34.463
y0	89.269	86.306	91.574	93.026
R	0.9988	0.9991	0.999	0.9994
$CP_{(SDM)}$	31.05	31.16	31.39	31.11

Well/ Cycle	luorescence Data Normalized To Y B9	C3	C4	C5
0.56	-0.03	0.01	0.00	-0.02
1.56	0.00	0.03	0.00	0.01
2.56	-0.03	0.01	0.01	-0.01
3.56	0.01	0.02	0.03	0.01
4.56	-0.01	0.00	0.01	-0.01
5.56	0.01	0.01	0.01	-0.01
6.56	0.00	0.01	0.00	0.01
7.56	0.00	0.01	0.02	0.01
8.56	0.02	0.00	0.00	0.01
9.56	0.01	0.02	0.01	0.00
10.56	0.02	0.02	0.01	0.01
11.56	0.02	0.01	0.02	0.01
12.56	0.00	0.01	0.01	0.01
13.56	0.01	0.00	0.01	0.00
14.56	0.00	0.02	0.00	0.01
15.56	0.03	0.01	0.01	0.00
16.58	0.01	0.00	0.01	0.02
17.58	0.02	-0.01	-0.01	0.01
18.58	-0.01	0.00	0.00	0.01
19.58	-0.01	0.00	0.01	0.01
20.58	0.01	-0.02	-0.01	0.02
21.58	0.02	-0.01	-0.01	0.02
22.58	0.00	0.00	0.00	0.01
23.58	0.02	0.00	0.01	0.01
24.58	0.03	0.01	0.00	0.02
25.58	0.04	0.02	0.02	0.04
26.58	0.05	0.05	0.04	0.05
27.58	0.12	0.09	0.09	0.09
28.56	0.14	0.14	0.13	0.13
29.61	0.24	0.23	0.22	0.22
30.56	0.32	0.33	0.29	0.32
31.63	0.48	0.47	0.43	0.47
32.63	0.57	0.58	0.59	0.58
33.63	0.77	0.78	0.73	0.77
34.63	0.95	0.94	0.91	0.93
35.63	1.09	1.05	1.03	1.08
36.63	1.18	1.23	1.16	1.20
37.65	1.33	1.36	1.35	1.34
38.65	1.46	1.50	1.48	1.49
39.65	1.63	1.63	1.62	1.60

B9			C3			
Well/ Cycle	FAM Normalized Fluorescence	VIC Normalized Fluorescence	Well/ Cycle	FAM Normalized Fluorescence	VIC Normalized Fluorescence	Well/ Cycle
0.56	-0.014892997	-0.027325183	0.56	-0.017250094	0.007812925	0.56
1.56	-0.019190529	-0.002008529	1.56	0.005023036	0.025193006	1.56
2.56	0.017747786	-0.030573781	2.56	0.006626702	0.01464909	2.56
3.56	-5.11611E-06	0.012442127	3.56	0.011497093	0.024381935	3.56
4.56	0.035654171	-0.008505724	4.56	0.009596452	0.004916245	4.56
5.56	0.012989804	0.007737262	5.56	0.01868389	0.014880825	5.56
6.56	0.008487627	0.00157613	6.56	0.033651433	0.006074917	6.56
7.56	0.010534071	0.003704521	7.56	0.017436594	0.014069754	7.56
8.56	0.019077975	0.019163363	8.56	0.016367484	0.004221042	8.56
9.56	0.040616798	0.006617056	9.56	0.009477662	0.015460161	9.56
10.56	0.015343214	0.023868228	10.56	0.018624495	0.017314036	10.56
11.56	-0.008651342	0.02241196	11.56	0.015595349	0.009666801	11.56
12.56	0.022045319	-0.002792673	12.56	0.02117848	0.010362004	12.56
13.56	0.000915784	0.014234457	13.56	-0.005430486	0.001671964	13.56
14.56	0.016878047	-0.003576818	14.56	0.014823214	0.015344294	14.56
15.56	0.017696625	0.033389978	15.56	-0.005133511	0.006074917	15.56
16.58	0.015394375	0.011770004	16.58	0.012328623	-0.002383388	16.58
17.58	0.001325073	0.024092269	17.58	-0.005371091	-0.013159038	17.58
18.58	0.017645464	-0.008841785	18.58	0.007042467	-0.000761248	18.58
19.58	-0.030241327	-0.005369147	19.58	-0.000203725	-0.000877115	19.58
20.58	0.006901633	0.010425757	20.58	0.006270332	-0.020922141	20.58
21.58	-0.011669847	0.020619631	21.58	-0.014993083	-0.008292616	21.58
22.58	-0.000107438	0.004040583	22.58	-0.02039803	0.003989308	22.58
23.58	-0.008600181	0.021739837	23.58	-0.0195665	0.001324362	23.58
24.58	0.011659615	0.02801299	24.58	0.009477662	0.01094134	24.58
25.58	0.013808381	0.043471832	25.58	0.00139994	0.024034334	25.58
26.58	0.050183924	0.052769541	26.58	0.0209409	0.049061649	26.58
27.58	0.090908161	0.117517444	27.58	0.054142713	0.09378639	27.58
28.56	0.157571076	0.136672966	28.56	0.140265484	0.136541387	28.56
29.61	0.23630801	0.237267459	29.61	0.180654094	0.230625555	29.61
30.56	0.388921575	0.320722802	30.56	0.305739994	0.32528906	30.56
31.63	0.480858074	0.482368519	31.63	0.3723812	0.466415312	31.63
32.63	0.607532961	0.568176294	32.63	0.539578165	0.58413639	32.63
33.63	0.732212564	0.772613877	33.63	0.615069228	0.783427978	33.63
34.63	0.825121124	0.954535322	34.63	0.716931677	0.938458294	34.63
35.63	0.988785487	1.086495581	35.63	0.849263769	1.054325496	35.63
36.63	1.084047457	1.179808736	36.63	0.949641343	1.228010433	36.63
37.65	1.19429963	1.332044723	37.65	1.057146319	1.363227458	37.65
38.65	1.280761891	1.456835665	38.65	1.1443382	1.501920499	38.65
39.65	1.375921539	1.628115153	39.65	1.203079869	1.62902682	39.65

Experiment derived	linear regression	eriment derived	linear regress	eriment der
parameter	value	parameter	value	parameter
y0	0.0196	y0	0.0135	y0
a	0.9704	a	0.9003	a
R	0.98788979	R	0.99036027	R

C4			C5	
**FAM** Normalized Fluorescence	VIC Normalized Fluorescence	Well/ Cycle	**FAM** Normalized Fluorescence	VIC Normalized Fluorescence
-0.040889229	0.002687447	0.56	-0.033335912	-0.022423838
-0.012712704	0.004871475	1.56	-0.038861812	0.006815299
0.003568186	0.005526684	2.56	-0.005399418	-0.014361576
-0.012611893	0.027585371	3.56	-0.007548379	0.012297637
0.0065925	0.014808805	4.56	0.003810415	-0.008986735
0.004072239	0.012952381	5.56	0.007985539	-0.005331843
0.028115535	0.001923037	6.56	0.015414805	0.007997764
0.01294356	0.018084847	7.56	-0.001531288	0.007352783
0.033055248	0.004107065	8.56	0.006204972	0.007890267
0.01082654	0.012187971	9.56	0.02008112	0.000150496
0.024385548	0.010986755	10.56	0.023151065	0.009610216
0.014405312	0.017211236	11.56	-0.004048643	0.012620128
0.028216345	0.007273907	12.56	0.018791744	0.008535248
0.000392657	0.012843179	13.56	0.006696163	0.004557866
0.027964319	0.001049426	14.56	0.012406259	0.011437663
0.002761703	0.014808805	15.56	0.011423877	0.000580483
0.012389102	0.012624776	16.58	0.020265317	0.015092555
0.004929128	-0.014457176	17.58	0.017563766	0.006170318
0.013598828	-0.00320943	18.58	0.005652382	0.011222669
-0.000514637	0.009130331	19.58	0.006450567	0.005632834
-0.000514637	-0.007140681	20.58	0.001477257	0.019499925
-0.013569593	-0.009324709	21.58	0.014064029	0.01627502
0.010171272	-0.000806999	22.58	0.030764527	0.011760153
0.005937232	0.005308281	23.58	0.002029847	0.013480102
0.011280187	0.004980677	24.58	0.019160137	0.023692301
-0.001421932	0.021142487	25.58	0.0267122	0.043686711
0.03789415	0.044729994	26.58	0.047772019	0.049384043
0.060677315	0.093433828	27.58	0.068709039	0.09356524
0.135025034	0.132200332	28.56	0.148036402	0.128394212
0.208213432	0.223711123	29.61	0.197708102	0.215789134
0.331454226	0.290105587	30.56	0.324128412	0.320491046
0.44053115	0.427262572	31.63	0.41088504	0.465181777
0.620881073	0.590955502	32.63	0.585687674	0.578483435
0.719574519	0.731388529	33.63	0.672812695	0.76746286
0.839538974	0.907421219	34.63	0.801443365	0.930643046
0.989595352	1.029508408	35.63	0.939959256	1.078881173
1.10507374	1.16033171	36.63	1.045012753	1.197772666
1.198525042	1.354928642	37.65	1.177818547	1.338701008
1.297168083	1.47614222	38.65	1.237068473	1.48779911
1.402767046	1.622581325	39.65	1.326956445	1.598950831

**rived** linear regress		**periment derived**	linear regress	
**value**		**parameter**	**value**	
-0.0074		y0	0.0135	0.0244
1.0317		a	0.9003	0.0563
**0.99483364**		**R**	**0.99036027**	
		**AVERAGE**	value	
		y0	0.0098	
		**a**	0.950675	

Well/ Cycle	Raw fluorescence data C6	C7	C8	C9	Well/ Cycle	C6	C7	C8	C9
0.56	176.02	152.86	164.43	153.13	0.56	-0.0579	-0.06	-0.05	-0.05
1.56	178.87	156.37	168.45	157.65	1.56	-0.0426	-0.04	-0.02	-0.02
2.56	184.88	159.54	166.80	157.03	2.56	-0.0104	-0.02	-0.03	-0.02
3.56	181.76	158.66	170.83	159.88	3.56	-0.0271	-0.02	-0.01	0.00
4.56	186.96	161.63	172.80	160.22	4.56	0.0007	0.00	0.00	0.00
5.56	182.07	160.07	169.81	160.65	5.56	-0.0255	-0.01	-0.02	0.00
6.56	188.84	162.26	176.44	162.20	6.56	0.0108	0.00	0.02	0.01
7.56	186.13	162.48	173.13	159.01	7.56	-0.0037	0.00	0.00	-0.01
8.56	190.69	162.54	172.18	162.97	8.56	0.0207	0.00	0.00	0.02
9.56	187.65	162.72	173.21	161.28	9.56	0.0044	0.00	0.00	0.01
10.56	187.84	166.17	176.23	160.44	10.56	0.0054	0.02	0.02	0.00
11.56	189.96	162.28	175.07	161.13	11.56	0.0168	0.00	0.01	0.00
12.56	190.02	164.59	173.37	163.79	12.56	0.0171	0.01	0.00	0.02
13.56	188.05	163.41	173.55	159.38	13.56	0.0065	0.01	0.00	-0.01
14.56	190.79	166.17	181.04	164.06	14.56	0.0212	0.02	0.05	0.02
15.56	189.83	165.20	175.71	161.65	15.56	0.0161	0.02	0.02	0.01
16.58	190.10	164.99	175.90	161.76	16.58	0.0175	0.02	0.02	0.01
17.58	190.55	166.28	171.79	161.55	17.58	0.0199	0.02	-0.01	0.01
18.58	191.70	165.61	178.91	163.27	18.58	0.0261	0.02	0.04	0.02
19.58	188.39	166.84	175.80	162.07	19.58	0.0084	0.03	0.02	0.01
20.58	190.92	166.28	178.13	164.14	20.58	0.0219	0.02	0.03	0.02
21.58	192.44	166.96	174.07	163.74	21.58	0.0300	0.03	0.01	0.02
22.58	194.04	168.28	175.12	166.15	22.58	0.0386	0.04	0.01	0.04
23.58	192.74	168.68	177.19	165.89	23.58	0.0316	0.04	0.03	0.03
24.58	198.18	169.39	176.56	166.00	24.58	0.0608	0.04	0.02	0.03
25.58	192.75	167.49	178.39	164.88	25.58	0.0317	0.03	0.03	0.03
26.58	199.04	173.12	183.74	169.26	26.58	0.0654	0.07	0.06	0.06
27.58	203.44	175.39	184.60	172.37	27.58	0.0889	0.08	0.07	0.07
28.56	214.77	185.02	196.95	182.21	28.56	0.1496	0.14	0.14	0.14
29.61	224.88	193.80	208.17	193.38	29.61	0.2037	0.19	0.21	0.21
30.56	245.94	210.47	229.44	208.46	30.56	0.3164	0.30	0.33	0.30
31.63	259.83	220.29	243.32	218.17	31.63	0.3907	0.36	0.41	0.36
32.63	289.96	243.89	268.90	242.50	32.63	0.5520	0.50	0.56	0.51
33.63	306.76	254.71	282.63	248.89	33.63	0.6419	0.57	0.64	0.55
34.63	321.05	270.56	304.70	267.59	34.63	0.7184	0.67	0.76	0.67
35.63	348.60	290.13	326.13	289.79	35.63	0.8659	0.79	0.89	0.81
36.63	366.62	303.10	341.56	302.96	36.63	0.9623	0.87	0.98	0.89
37.65	383.65	319.45	362.16	314.65	37.65	1.0535	0.97	1.10	0.96
38.65	399.62	331.47	375.03	326.24	38.65	1.1390	1.04	1.17	1.03
39.65	416.54	341.76	387.55	339.57	39.65	1.2295	1.10	1.24	1.12
Parameter									
a	255.483300	202.213100	234.644200	197.649600					
b	2.766300	2.799000	2.600800	2.721000					
x0	34.115800	34.117400	33.905200	34.000900					
y0	186.829400	162.373900	172.714600	160.427800					
R	0.998317	0.998370	0.998453	0.998613					
**CP$_{(SDM)}$**	30.47	30.43	30.48	30.42					

Well/ Cycle	C6	C7	C8	C9
0.56	99.47	93.62	88.73	81.11
1.56	103.47	93.83	88.86	82.13
2.56	103.43	93.22	90.3	81.76
3.56	103.68	94.05	89.81	83.14
4.56	102.84	93.76	89.09	83.53
5.56	104.25	94.37	88.59	83.48
6.56	104.81	93.37	90.4	84.55
7.56	104.57	94.86	91.34	83.71
8.56	104.19	95.7	90.79	83.8
9.56	104.92	96.01	91.57	84.11
10.56	104.67	96.28	91.9	83.99
11.56	105.92	97.4	90.98	83.65
12.56	104.88	96.2	91.66	85.22
13.56	106.12	95.22	90.83	83.68
14.56	104.3	95.2	89.32	85.33
15.56	105.12	96.71	91.59	84.38
16.58	105.6	96.29	90.93	86.01
17.58	105.41	96.76	91.57	84.6
18.58	105.07	96.84	91.11	85.02
19.58	105.34	96.3	91.37	85.93
20.58	104.87	96.79	91.67	85.43
21.58	108.12	97.78	92.61	85.74
22.58	107.52	97.45	93.09	86.26
23.58	108.91	100.59	92.22	86.21
24.58	109.16	100.09	94.04	87.66
25.58	111.07	100.93	96.83	87.98
26.58	114.24	103.98	97.2	91.87
27.58	117.96	108.46	101.7	93.65
28.56	124.24	111.94	108.76	96.45
29.61	136.66	120.92	117.52	103.75
30.56	148.29	133.7	125.76	115.32
31.63	166.89	149	142.61	126.16
32.63	184.76	164.03	158.95	137.93
33.63	205.11	183.17	177.34	154.63
34.63	227.33	197.61	196.45	168.66
35.63	244.47	211.79	209.25	179.78
36.63	258.45	227.33	227.01	195.37
37.65	276.3	239.57	238.54	207.77
38.65	293.05	252.54	249.8	212.91
39.65	307.98	263.76	263.4	226.2
Parameter				
a	225.64	185.31	187.83	156.33
b	2.657	2.6254	2.5245	2.5676
x0	34.285	34.1	34.1	34.263
y0	104.1	95.134	90.329	83.856
R	0.9995	0.9995	0.9996	0.9994
$CP_{(SDM)}$	30.79	30.64	30.77	30.88

Well/ Cycle	luorescence Data Normalized To Y C6	C7	C8	C9
0.56	-0.04	-0.02	-0.02	-0.03
1.56	-0.01	-0.01	-0.02	-0.02
2.56	-0.01	-0.02	0.00	-0.02
3.56	0.00	-0.01	-0.01	-0.01
4.56	-0.01	-0.01	-0.01	0.00
5.56	0.00	-0.01	-0.02	0.00
6.56	0.01	-0.02	0.00	0.01
7.56	0.00	0.00	0.01	0.00
8.56	0.00	0.01	0.01	0.00
9.56	0.01	0.01	0.01	0.00
10.56	0.01	0.01	0.02	0.00
11.56	0.02	0.02	0.01	0.00
12.56	0.01	0.01	0.01	0.02
13.56	0.02	0.00	0.01	0.00
14.56	0.00	0.00	-0.01	0.02
15.56	0.01	0.02	0.01	0.01
16.58	0.01	0.01	0.01	0.03
17.58	0.01	0.02	0.01	0.01
18.58	0.01	0.02	0.01	0.01
19.58	0.01	0.01	0.01	0.02
20.58	0.01	0.02	0.01	0.02
21.58	0.04	0.03	0.03	0.02
22.58	0.03	0.02	0.03	0.03
23.58	0.05	0.06	0.02	0.03
24.58	0.05	0.05	0.04	0.05
25.58	0.07	0.06	0.07	0.05
26.58	0.10	0.09	0.08	0.10
27.58	0.13	0.14	0.13	0.12
28.56	0.19	0.18	0.20	0.15
29.61	0.31	0.27	0.30	0.24
30.56	0.42	0.41	0.39	0.38
31.63	0.60	0.57	0.58	0.50
32.63	0.77	0.72	0.76	0.64
33.63	0.97	0.93	0.96	0.84
34.63	1.18	1.08	1.17	1.01
35.63	1.35	1.23	1.32	1.14
36.63	1.48	1.39	1.51	1.33
37.65	1.65	1.52	1.64	1.48
38.65	1.82	1.65	1.77	1.54
39.65	1.96	1.77	1.92	1.70

C6			C7			
Well/ Cycle	FAM Normalized Fluorescence	VIC Normalized Fluorescence	Well/ Cycle	FAM Normalized Fluorescence	VIC Normalized Fluorescence	Well/ Cycle
0.56	-0.057857061	-0.044440666	0.56	-0.058592545	-0.015916463	0.56
1.56	-0.042602503	-0.006014635	1.56	-0.03697577	-0.013709055	1.56
2.56	-0.010434118	-0.006398895	2.56	-0.017452928	-0.02012105	2.56
3.56	-0.027133845	-0.003997268	3.56	-0.022872518	-0.011396532	3.56
4.56	0.000699033	-0.012066734	4.56	-0.004581401	-0.014444858	4.56
5.56	-0.025474577	0.001478442	5.56	-0.014188857	-0.008032863	5.56
6.56	0.01076169	0.006858086	6.56	-0.000701467	-0.01854433	6.56
7.56	-0.003743522	0.004552524	7.56	0.00065343	-0.002882244	7.56
8.56	0.020663771	0.000902051	8.56	0.001022948	0.005947388	8.56
9.56	0.004392242	0.007914802	9.56	0.0021315	0.009205943	9.56
10.56	0.005409213	0.005513175	10.56	0.023378757	0.012044039	10.56
11.56	0.016756463	0.01752131	11.56	-0.000578295	0.023816882	11.56
12.56	0.017077612	0.007530541	12.56	0.013648129	0.011203121	12.56
13.56	0.006533233	0.019442611	13.56	0.006380952	0.000901884	13.56
14.56	0.021199019	0.001958767	14.56	0.023378757	0.000691655	14.56
15.56	0.016060641	0.009836103	15.56	0.017404891	0.01656397	15.56
16.58	0.01750581	0.014447227	16.58	0.01611158	0.012149154	16.58
17.58	0.019914425	0.012621991	17.58	0.024056206	0.017089543	17.58
18.58	0.026069773	0.009355778	18.58	0.019929927	0.01793046	18.58
19.58	0.008353075	0.011949535	19.58	0.027505036	0.012254268	19.58
20.58	0.021894841	0.007434476	20.58	0.024056206	0.017404887	20.58
21.58	0.030030605	0.038655627	21.58	0.028244071	0.027811239	21.58
22.58	0.038594568	0.032891722	22.58	0.036373457	0.024342455	22.58
23.58	0.031636348	0.046244768	23.58	0.038836907	0.057348461	23.58
24.58	0.060753821	0.048646395	24.58	0.043209531	0.052092728	24.58
25.58	0.031689873	0.066994825	25.58	0.031508143	0.06092236	25.58
26.58	0.065356951	0.097447455	26.58	0.066181203	0.092982334	26.58
27.58	0.088907849	0.133183664	27.58	0.080161282	0.140073706	27.58
28.56	0.149551409	0.193512533	28.56	0.139468843	0.176653611	28.56
29.61	0.203664948	0.31282536	29.61	0.193541573	0.271046585	29.61
30.56	0.316388106	0.424549047	30.56	0.296205856	0.405383132	30.56
31.63	0.390734007	0.603230092	31.63	0.356683556	0.566208577	31.63
32.63	0.552004128	0.774898387	32.63	0.502027112	0.724195925	32.63
33.63	0.641925735	0.970390822	33.63	0.568663437	0.925385403	33.63
34.63	0.718412627	1.183847426	34.63	0.666277647	1.077170986	34.63
35.63	0.865873358	1.34850297	35.63	0.786801943	1.226223587	35.63
36.63	0.962324987	1.482801949	36.63	0.866679312	1.389571784	36.63
37.65	1.053477665	1.654278114	37.65	0.967372835	1.518232139	37.65
38.65	1.138956717	1.81518712	38.65	1.041399511	1.654565866	38.65
39.65	1.229520621	1.958612282	39.65	1.104771764	1.772504525	39.65

Experiment derived	linear regression
parameter	value
y0	0.0105
a	0.6651
R	0.99550803

periment derived	linear regress
parameter	value
y0	0.022
a	0.6178
R	0.99158714

periment der
parameter
y0
a
R

C8			C9	
**FAM** Normalized Fluorescence	VIC Normalized Fluorescence	Well/ Cycle	**FAM** Normalized Fluorescence	VIC Normalized Fluorescence
-0.047966993	-0.017701956	0.56	-0.045489622	-0.032746613
-0.024691601	-0.016262773	1.56	-0.017314954	-0.020582904
-0.034244934	-0.000321049	2.56	-0.021179621	-0.02499523
-0.010911643	-0.005745663	3.56	-0.00341462	-0.008538447
0.000494457	-0.013716525	4.56	-0.001295287	-0.003887617
-0.016817339	-0.019251846	5.56	0.001385047	-0.004483877
0.021569688	0.000786016	6.56	0.011046714	0.008276092
0.002405124	0.011192419	7.56	-0.00883762	-0.00174108
-0.00309528	0.005103566	8.56	0.015846381	-0.000667811
0.002868316	0.013738666	9.56	0.005312047	0.003029002
0.020353809	0.017391978	10.56	7.60467E-05	0.001597977
0.013637527	0.007206988	11.56	0.004377047	-0.002456592
0.003794699	0.014735024	12.56	0.020957714	0.01626598
0.004836881	0.005546392	13.56	-0.006531287	-0.002098836
0.048203221	-0.011170278	14.56	0.022640714	0.017577752
0.017343062	0.013960079	15.56	0.00761838	0.006248807
0.018443143	0.006653456	16.58	0.008304047	0.025686892
-0.00535334	0.013738666	17.58	0.006995047	0.008872353
0.035870737	0.008646171	18.58	0.017716381	0.013880939
0.017864153	0.011524538	19.58	0.01023638	0.024732875
0.031354616	0.014845731	20.58	0.023139381	0.018770273
0.007847628	0.025252134	21.58	0.020646048	0.022467086
0.013927022	0.030566042	22.58	0.035668382	0.028668193
0.025912112	0.020934584	23.58	0.034047715	0.028071933
0.022264476	0.041083152	24.58	0.034733382	0.04536348
0.03285999	0.071970242	25.58	0.027752048	0.049179546
0.063835947	0.07606638	26.58	0.055054049	0.095568594
0.068815259	0.125884268	27.58	0.074439717	0.116795459
0.140320506	0.204042998	28.56	0.13577572	0.150186033
0.205283167	0.30102182	29.61	0.205402056	0.237240031
0.328434307	0.392243908	30.56	0.299400727	0.375214654
0.408798098	0.578784222	31.63	0.359926397	0.504483877
0.556903701	0.759678509	32.63	0.511583404	0.644843541
0.636399007	0.963267611	33.63	0.551414406	0.843994467
0.764182067	1.174827575	34.63	0.667977745	1.011305094
0.888259591	1.316531789	35.63	0.806357751	1.143913375
0.977597725	1.513146387	36.63	0.888450755	1.329827323
1.096869633	1.640790887	37.65	0.961318425	1.477699866
1.171385627	1.765446313	38.65	1.033562762	1.538995421
1.243875156	1.916007041	39.65	1.116653099	1.697481397

rived linear regress
**value**
0.0085
0.6875
**0.99025758**

**periment derived**	linear regress	
**parameter**	**value**	
y0	0.0334	0.0256
a	0.6628	0.0533
**R**	**0.98422807**	

	**value**
y0	0.0186
a	0.6583
**R**	

Well/ Cycle	Raw fluorescence data D3	D4	D5	D6	D7	Well/ Cycle	D3	D4	D5	D6	D7
0.56	157.21	122.21	150.05	149.74	214.60	0.56	-0.0222	-0.03	-0.03	-0.01	-0.02
1.56	158.01	122.63	153.66	149.52	216.14	1.56	-0.0173	-0.02	-0.01	-0.01	-0.01
2.56	161.18	124.58	152.26	151.32	219.49	2.56	0.0025	-0.01	-0.02	0.00	0.01
3.56	159.74	122.76	152.98	152.99	220.62	3.56	-0.0065	-0.02	-0.01	0.01	0.01
4.56	162.88	126.15	155.58	152.53	220.69	4.56	0.0130	0.00	0.00	0.01	0.01
5.56	162.29	125.40	154.62	152.69	222.34	5.56	0.0094	0.00	0.00	0.01	0.02
6.56	161.65	126.57	159.82	153.70	220.77	6.56	0.0054	0.01	0.03	0.02	0.01
7.56	160.19	125.42	154.93	150.70	218.33	7.56	-0.0037	0.00	0.00	0.00	0.00
8.56	163.56	127.62	157.75	153.88	215.39	8.56	0.0173	0.02	0.02	0.02	-0.01
9.56	162.54	125.79	154.13	150.05	220.93	9.56	0.0109	0.00	-0.01	0.00	0.01
10.56	163.36	128.88	157.48	154.49	220.05	10.56	0.0160	0.03	0.02	0.02	0.01
11.56	161.32	125.20	154.68	150.64	220.10	11.56	0.0033	0.00	0.00	0.00	0.01
12.56	163.11	126.99	157.93	151.24	219.78	12.56	0.0145	0.01	0.02	0.00	0.01
13.56	162.15	128.03	154.48	149.89	217.95	13.56	0.0085	0.02	0.00	-0.01	0.00
14.56	162.13	126.70	156.78	153.86	220.81	14.56	0.0084	0.01	0.01	0.02	0.01
15.56	160.39	127.61	155.19	150.22	221.49	15.56	-0.0025	0.02	0.00	0.00	0.02
16.58	163.17	126.47	156.06	151.27	221.82	16.58	0.0148	0.01	0.01	0.00	0.02
17.58	160.37	125.94	156.40	149.92	217.47	17.58	-0.0026	0.00	0.01	-0.01	0.00
18.58	163.70	126.20	156.75	152.40	222.30	18.58	0.0181	0.01	0.01	0.01	0.02
19.58	161.61	126.82	156.68	152.32	224.20	19.58	0.0051	0.01	0.01	0.01	0.03
20.58	162.19	126.15	158.21	151.71	221.98	20.58	0.0087	0.00	0.02	0.01	0.02
21.58	159.09	126.65	155.31	149.79	216.14	21.58	-0.0105	0.01	0.00	-0.01	-0.01
22.58	161.59	127.55	159.10	151.47	216.05	22.58	0.0050	0.02	0.03	0.00	-0.01
23.58	162.45	128.74	156.27	150.10	219.16	23.58	0.0104	0.03	0.01	0.00	0.00
24.58	162.49	126.75	159.74	149.80	218.61	24.58	0.0106	0.01	0.03	-0.01	0.00
25.58	163.01	127.12	159.63	151.99	217.62	25.58	0.0138	0.01	0.03	0.01	0.00
26.58	164.64	128.74	165.51	152.94	219.69	26.58	0.0240	0.03	0.07	0.01	0.01
27.58	167.30	129.89	163.43	154.86	223.28	27.58	0.0405	0.03	0.05	0.03	0.02
28.56	173.52	134.58	170.86	161.47	239.46	28.56	0.0792	0.07	0.10	0.07	0.10
29.61	180.85	139.74	175.06	166.95	251.14	29.61	0.1248	0.11	0.13	0.11	0.15
30.56	193.51	147.16	189.02	181.81	272.56	30.56	0.2035	0.17	0.22	0.21	0.25
31.63	207.69	151.22	195.38	185.68	285.28	31.63	0.2917	0.20	0.26	0.23	0.31
32.63	222.90	161.05	213.43	206.07	315.14	32.63	0.3863	0.28	0.38	0.37	0.44
33.63	234.93	168.31	219.57	211.34	332.74	33.63	0.4611	0.34	0.42	0.40	0.53
34.63	246.51	176.49	232.29	221.32	353.80	34.63	0.5332	0.41	0.50	0.47	0.62
35.63	261.36	186.59	244.68	237.00	379.38	35.63	0.6255	0.49	0.58	0.57	0.74
36.63	276.59	200.10	253.18	247.06	393.37	36.63	0.7202	0.59	0.63	0.64	0.80
37.65	289.84	209.67	265.00	259.50	416.96	37.65	0.8027	0.67	0.71	0.72	0.91
38.65	302.25	214.42	274.86	269.64	438.27	38.65	0.8798	0.71	0.77	0.79	1.01
39.65	310.44	229.47	283.99	279.75	453.17	39.65	0.9308	0.83	0.83	0.86	1.08
Parameter											
a	164.3334	127.0203	144.3406	140.8843	252.9196						
b	2.5147	2.9348	2.8051	2.5282	2.4510						
x0	34.2529	35.7050	34.2203	34.3940	34.1912						
y0	160.7854	125.5662	155.1217	150.7943	218.1714						
R	0.9986	0.9979	0.9982	0.9974	0.9979						
**$CP_{(SDM)}$**	30.94	31.84	30.53	31.06	30.96						

Well/						Well/	Fluorescence Data Normalized To Y0				
Cycle	**D3**	**D4**	**D5**	**D6**	**D7**	Cycle	**D3**	**D4**	**D5**	**D6**	**D7**
0.56	78.7	84.3	123.94	117.84	116.78	0.56	0.00	0.02	0.02	0.01	0.01
1.56	79.58	82.46	124.65	122.32	120.21	1.56	0.01	0.00	0.03	0.05	0.03
2.56	78.97	82.95	124.51	120.71	118.4	2.56	0.01	0.00	0.03	0.04	0.02
3.56	78.77	84.59	124.3	120.91	117.21	3.56	0.00	0.02	0.03	0.04	0.01
4.56	79.55	83.66	125.29	122.3	119.23	4.56	0.01	0.01	0.04	0.05	0.03
5.56	79.26	83.53	122.45	119.67	117.59	5.56	0.01	0.01	0.01	0.03	0.01
6.56	78.59	83.45	122.64	118.64	118.37	6.56	0.00	0.01	0.01	0.02	0.02
7.56	79.47	84.01	125.04	120.8	119.58	7.56	0.01	0.02	0.03	0.04	0.03
8.56	80.06	83.26	122.11	118.33	118.54	8.56	0.02	0.01	0.01	0.02	0.02
9.56	79.47	84.37	121.55	117.61	120.44	9.56	0.01	0.02	0.00	0.01	0.04
10.56	80.95	83.62	123.03	116.52	116.53	10.56	0.03	0.01	0.02	0.00	0.00
11.56	80.96	84.22	120.15	117.26	117.22	11.56	0.03	0.02	-0.01	0.01	0.01
12.56	78.25	83.56	121.23	116.85	115.74	12.56	0.00	0.01	0.00	0.01	0.00
13.56	79.01	83.48	122.62	115.53	118.32	13.56	0.01	0.01	0.01	-0.01	0.02
14.56	79.78	83.68	122.71	117.02	114.43	14.56	0.02	0.01	0.01	0.01	-0.02
15.56	79.16	82.46	121.98	115.63	118.12	15.56	0.01	0.00	0.01	0.00	0.02
16.58	78.78	83.34	121.58	115.67	119.65	16.58	0.00	0.01	0.00	0.00	0.03
17.58	78.18	82.93	119.28	116.74	120.57	17.58	0.00	0.00	-0.01	0.01	0.04
18.58	79.2	82.41	122.76	118.22	117.91	18.58	0.01	0.00	0.01	0.02	0.01
19.58	79.73	83.53	121.06	114.23	113.87	19.58	0.01	0.01	0.00	-0.02	-0.02
20.58	79.14	81.98	120.42	112.74	112.33	20.58	0.01	-0.01	0.00	-0.03	-0.03
21.58	78.05	82.92	123.3	114.96	116.17	21.58	-0.01	0.00	0.02	-0.01	0.00
22.58	79.42	83.14	123.39	115.35	116.03	22.58	0.01	0.01	0.02	-0.01	0.00
23.58	80.15	83.13	121.82	114.25	117.69	23.58	0.02	0.01	0.01	-0.02	0.01
24.58	79.41	83.66	123.47	116.9	117.96	24.58	0.01	0.01	0.02	0.01	0.02
25.58	81.81	85.81	130.2	121.26	121.34	25.58	0.04	0.04	0.08	0.04	0.04
26.58	83.93	86.86	138.64	125.42	126.82	26.58	0.07	0.05	0.15	0.08	0.09
27.58	89.24	91.16	146.73	133.61	133.16	27.58	0.14	0.10	0.21	0.15	0.15
28.56	95.16	96.77	161.19	145.25	145.67	28.56	0.21	0.17	0.33	0.25	0.25
29.61	105.72	107.47	184.3	164.23	164.28	29.61	0.35	0.30	0.52	0.41	0.41
30.56	117.88	127.1	213.98	187.09	185.12	30.56	0.50	0.54	0.77	0.61	0.59
31.63	136.51	147.53	255.5	222.33	223.04	31.63	0.74	0.79	1.11	0.91	0.92
32.63	152.86	162.67	297.98	256.24	255.57	32.63	0.95	0.97	1.46	1.21	1.20
33.63	172.82	186.13	345.25	296.13	289.05	33.63	1.20	1.25	1.85	1.55	1.49
34.63	187.75	204.12	385.8	329.64	323.06	34.63	1.39	1.47	2.19	1.84	1.78
35.63	201.92	218.62	421.53	355.8	346.19	35.63	1.57	1.65	2.48	2.06	1.98
36.63	217.52	232.99	459.13	387.06	379.94	36.63	1.77	1.82	2.79	2.33	2.27
37.65	231.68	249.74	489.73	415.08	408.62	37.65	1.95	2.02	3.05	2.57	2.52
38.65	243.59	260.42	522.51	443.12	431.05	38.65	2.10	2.15	3.32	2.81	2.71
39.65	254.29	273.3	553.67	466.28	453.81	39.65	2.24	2.31	3.58	3.01	2.91
Parameter											
a	185.77	196.17	458.96	369.69	358.474						
b	2.3968	2.2437	2.4247	2.3526	2.3884						
x0	33.753	33.545	33.911	33.956	33.9367						
y0	78.564	82.578	120.99	116.15	116.188						
R	0.9993	0.9989	0.9994	0.999	0.999089						
**$CP_{(SDM)}$**	30.60	30.59	30.72	30.86	30.79						

D3			D4			
Well/ Cycle	**FAM** Normalized Fluorescence	VIC Normalized Fluorescence	Well/ Cycle	**FAM** Normalized Fluorescence	VIC Normalized Fluorescence	Well/ Cycle
0.56	-0.022237094	0.001732348	0.56	-0.02672853	0.020859193	0.56
1.56	-0.017261518	0.012933421	1.56	-0.023383681	-0.001422906	1.56
2.56	0.002454203	0.005169041	2.56	-0.007854024	0.004510914	2.56
3.56	-0.006501834	0.002623342	3.56	-0.022348371	0.024371045	3.56
4.56	0.013027302	0.012551566	4.56	0.00464934	0.013108898	4.56
5.56	0.009357815	0.008860304	5.56	-0.001323605	0.011534619	5.56
6.56	0.005377354	0.000332214	6.56	0.00799419	0.010565832	6.56
7.56	-0.003703073	0.011533287	7.56	-0.001164326	0.01734734	7.56
8.56	0.017256542	0.019043097	8.56	0.016356312	0.008264963	8.56
9.56	0.010912682	0.011533287	9.56	0.001782327	0.021706881	9.56
10.56	0.016012648	0.030371456	10.56	0.02639086	0.012624504	10.56
11.56	0.003324929	0.030498741	11.56	-0.00291639	0.019890406	11.56
12.56	0.01445778	-0.003995474	12.56	0.011339039	0.011897914	12.56
13.56	0.008487089	0.00567818	13.56	0.019621522	0.010929127	13.56
14.56	0.0083627	0.01547912	14.56	0.0090295	0.013351094	14.56
15.56	-0.002459179	0.007587454	15.56	0.016276673	-0.001422906	15.56
16.58	0.014830949	0.002750627	16.58	0.007197797	0.00923375	16.58
17.58	-0.002583568	-0.004886468	17.58	0.002976916	0.004268717	17.58
18.58	0.018127268	0.008096594	18.58	0.005047537	-0.002028398	18.58
19.58	0.005128575	0.014842695	19.58	0.009985171	0.011534619	19.58
20.58	0.008735868	0.007332884	20.58	0.00464934	-0.007235627	20.58
21.58	-0.01054449	-0.006541172	21.58	0.008631304	0.004147619	21.58
22.58	0.005004186	0.010896862	22.58	0.015798838	0.006811783	22.58
23.58	0.01035293	0.020188662	23.58	0.02527591	0.006690685	23.58
24.58	0.010601709	0.010769577	24.58	0.009427696	0.013108898	24.58
25.58	0.013835833	0.041317959	25.58	0.012374349	0.039145046	25.58
26.58	0.02397357	0.068302363	26.58	0.02527591	0.051860374	26.58
27.58	0.04051736	0.135890657	27.58	0.034434426	0.103932669	27.58
28.56	0.079202465	0.211243332	28.56	0.071785242	0.17186885	28.56
29.61	0.124791181	0.345656211	29.61	0.112879103	0.301444098	29.61
30.56	0.203529674	0.500434678	30.56	0.171971438	0.539160183	30.56
31.63	0.291721761	0.73756649	31.63	0.20430498	0.786564137	31.63
32.63	0.386319902	0.94567734	32.63	0.282590379	0.969907057	32.63
33.63	0.461140128	1.199738048	33.63	0.340408486	1.254003815	33.63
34.63	0.533161593	1.389774438	34.63	0.405553405	1.471859768	34.63
35.63	0.625520725	1.570137175	35.63	0.485989064	1.647452393	35.63
36.63	0.720243256	1.768701656	36.63	0.593581712	1.82147074	36.63
37.65	0.802651236	1.948937107	37.65	0.66979649	2.024310496	37.65
38.65	0.879834861	2.100533451	38.65	0.707625141	2.153643547	38.65
39.65	0.930772321	2.236728319	39.65	0.827482236	2.309618237	39.65

**eriment derived**	linear regress
**parameter**	**value**
y0	-0.0074
a	0.402
**R**	**0.99812799**

**eriment derived**	linear regress
**parameter**	**value**
y0	0.0236
a	0.2552
**R**	**0.99270657**

**eriment de**
**parameter**
y0
a
**R**

D5			D6		D7	
FAM Normalized Fluorescence	VIC Normalized Fluorescence	Well/ Cycle	FAM Normalized Fluorescence	IC Normalize Fluorescenc	AM Normalize Fluorescence	VIC Normalized Fluorescence
-0.032694974	0.024388954	0.56	-0.006991644	0.01452919	-0.016369698	0.005095191
-0.009422924	0.030257246	1.56	-0.008450585	0.0530992	-0.009311028	0.034616311
-0.018448096	0.029100118	2.56	0.003486206	0.0392381	0.006043872	0.019038111
-0.013806579	0.027364426	3.56	0.014560895	0.04095998	0.011223286	0.008796089
0.002954454	0.035546974	4.56	0.011510382	0.05292702	0.011544135	0.026181706
-0.003234235	0.012073805	5.56	0.01257143	0.03028435	0.019106996	0.012066651
0.030287832	0.013644193	6.56	0.019269296	0.02141669	0.011910819	0.018779908
-0.001235804	0.033480674	7.56	-0.000625355	0.04001295	0.000726951	0.029194065
0.016943471	0.009263637	8.56	0.020462975	0.01874778	-0.012748692	0.020243054
-0.006393045	0.004635124	9.56	-0.004935863	0.01254903	0.012644187	0.036595862
0.015202902	0.016867621	10.56	0.024508221	0.00316481	0.008610661	0.002943505
-0.002847442	-0.006936156	11.56	-0.001023248	0.00953575	0.008839839	0.008882157
0.01810385	0.00199026	12.56	0.002955682	0.0060059	0.007373102	-0.00385582
-0.004136752	0.013478889	13.56	-0.005996911	-0.0053585	-0.001014798	0.018349571
0.010690316	0.014222757	14.56	0.020330344	0.0074695	0.012094161	-0.01513065
0.000440299	0.008189161	15.56	-0.003808499	-0.0044975	0.015210976	0.016628223
0.006048799	0.00488308	16.58	0.003154629	-0.0041532	0.016723549	0.029796537
0.008240627	-0.014126881	17.58	-0.005797965	0.00505887	-0.003214904	0.037714738
0.01049692	0.014636017	18.58	0.01064828	0.01780075	0.018923654	0.014820808
0.010045661	0.000585176	19.58	0.010117756	-0.0165507	0.027632403	-0.019950425
0.019908884	-0.004704552	20.58	0.006072511	-0.0293786	0.017456917	-0.033204806
0.001213886	0.019099225	21.58	-0.006660066	-0.0102658	-0.009311028	-0.000154921
0.025646315	0.019843093	22.58	0.004480939	-0.0069082	-0.009723548	-0.001359865
0.007402575	0.006866729	23.58	-0.004604285	-0.0163785	0.0045313	0.012927325
0.029772108	0.020504309	24.58	-0.006593751	0.00643637	0.002010346	0.015251145
0.029062987	0.076129109	25.58	0.007929345	0.04397326	-0.002527371	0.044341929
0.066968709	0.145887402	26.58	0.014229318	0.07978828	0.006960582	0.091506868
0.053559882	0.212752874	27.58	0.026961894	0.15029909	0.023415535	0.146073605
0.101457759	0.332267673	28.56	0.070796443	0.25051226	0.097577409	0.253743932
0.128533274	0.523276458	29.61	0.107137339	0.41391827	0.151113299	0.413915379
0.218527131	0.768587609	30.56	0.205682178	0.61072866	0.249292987	0.593279857
0.259527197	1.111758735	31.63	0.231346278	0.91412317	0.307595771	0.919647468
0.375887448	1.462864454	32.63	0.36656359	1.2060672	0.44446064	1.199624746
0.415469273	1.853560483	33.63	0.401511861	1.54949532	0.525131158	1.487778428
0.497469406	2.188714365	34.63	0.467694734	1.8379956	0.621660768	1.780493683
0.577342177	2.48402998	35.63	0.571677444	2.06321695	0.738908033	1.979567597
0.632137863	2.794801519	36.63	0.638390841	2.33234612	0.803031928	2.270045099
0.7083361	3.047716656	37.65	0.720887328	2.57358092	0.911157924	2.516886425
0.771899096	3.318649929	38.65	0.788131249	2.8149879	1.008833422	2.709935622
0.83075611	3.576193578	39.65	0.855176224	3.01438111	1.07712835	2.905825042

erived linear regres
**value**
0.0211
0.2243
**0.98996287**

iment derived	linear regre	
**parameter**	**value**	
y0	0.0022	0.0191
a	0.2736	0.022
**R**	**0.98427259**	

eriment derived	linear regress
**parameter**	**value**
y0	-0.0019
a	0.3613
**R**	**0.99255245**

	value	SD2
y0	0.00752	
a	0.30328	0.075070613
**R**		

Well/ Cycle	Raw fluorescence data					Well/ Cycle					
	D8	D9	E2	E3	E4		D8	D9	E2	E3	E4
0.56	229.79	166.54	147.23	151.65	143.84	0.56	-0.0160	-0.06	-0.07	-0.05	-0.03
1.56	234.43	174.87	158.92	155.48	144.53	1.56	0.0039	-0.01	0.00	-0.02	-0.02
2.56	238.66	175.23	158.08	157.91	147.90	2.56	0.0220	-0.01	0.00	-0.01	0.00
3.56	239.02	175.30	157.37	156.41	147.93	3.56	0.0236	-0.01	-0.01	-0.02	0.00
4.56	244.07	179.69	160.11	161.38	147.96	4.56	0.0452	0.02	0.01	0.01	0.00
5.56	235.61	175.75	158.80	159.87	148.03	5.56	0.0090	0.00	0.00	0.00	0.00
6.56	236.15	180.40	161.36	159.18	150.77	6.56	0.0113	0.02	0.02	0.00	0.02
7.56	236.34	178.76	159.38	161.40	149.22	7.56	0.0121	0.01	0.00	0.01	0.01
8.56	236.87	177.07	161.02	161.21	148.00	8.56	0.0144	0.00	0.02	0.01	0.00
9.56	235.72	177.13	160.73	161.61	147.15	9.56	0.0094	0.00	0.01	0.01	0.00
10.56	237.70	180.82	161.45	160.83	147.02	10.56	0.0179	0.02	0.02	0.01	0.00
11.56	235.63	176.26	158.99	161.74	150.19	11.56	0.0090	0.00	0.00	0.02	0.02
12.56	236.49	179.37	161.01	162.17	148.99	12.56	0.0127	0.02	0.01	0.02	0.01
13.56	233.80	177.21	159.39	160.74	146.20	13.56	0.0012	0.00	0.00	0.01	-0.01
14.56	235.92	179.68	162.03	160.91	148.11	14.56	0.0103	0.02	0.02	0.01	0.00
15.56	235.19	176.24	160.42	159.74	147.22	15.56	0.0072	0.00	0.01	0.00	0.00
16.58	237.17	177.93	161.21	163.17	150.59	16.58	0.0156	0.01	0.02	0.02	0.02
17.58	232.87	179.06	160.02	160.83	147.18	17.58	-0.0028	0.01	0.01	0.01	0.00
18.58	235.20	180.17	160.73	162.22	149.58	18.58	0.0072	0.02	0.01	0.02	0.01
19.58	230.79	177.00	157.65	159.21	147.53	19.58	-0.0117	0.00	-0.01	0.00	0.00
20.58	233.26	179.70	158.53	159.67	147.95	20.58	-0.0011	0.02	0.00	0.00	0.00
21.58	229.08	179.26	158.14	158.55	148.83	21.58	-0.0190	0.02	0.00	0.00	0.01
22.58	228.90	178.09	159.38	161.34	147.68	22.58	-0.0198	0.01	0.00	0.01	0.00
23.58	229.51	173.77	159.50	158.24	146.69	23.58	-0.0172	-0.02	0.01	-0.01	-0.01
24.58	229.24	177.29	159.93	160.55	147.51	24.58	-0.0183	0.00	0.01	0.01	0.00
25.58	227.46	177.63	160.11	162.22	150.85	25.58	-0.0259	0.01	0.01	0.02	0.02
26.58	233.10	180.12	161.29	163.04	151.56	26.58	-0.0018	0.02	0.02	0.02	0.03
27.58	234.86	179.48	164.53	162.88	150.37	27.58	0.0057	0.02	0.04	0.02	0.02
28.56	244.67	187.58	169.40	172.19	156.64	28.56	0.0478	0.06	0.07	0.08	0.06
29.61	252.20	195.32	175.03	175.45	160.95	29.61	0.0800	0.11	0.10	0.10	0.09
30.56	278.36	207.51	189.04	188.61	167.57	30.56	0.1920	0.18	0.19	0.18	0.14
31.63	287.09	214.19	191.18	194.93	170.62	31.63	0.2294	0.21	0.21	0.22	0.16
32.63	318.96	226.29	206.59	211.39	182.55	32.63	0.3659	0.28	0.30	0.33	0.24
33.63	323.18	237.01	214.12	221.34	187.88	33.63	0.3840	0.34	0.35	0.39	0.27
34.63	343.89	244.83	222.84	231.47	196.45	34.63	0.4726	0.39	0.40	0.45	0.33
35.63	365.56	259.15	236.77	249.35	205.72	35.63	0.5654	0.47	0.49	0.57	0.39
36.63	380.43	268.89	247.20	259.35	214.15	36.63	0.6291	0.52	0.56	0.63	0.45
37.65	398.66	278.77	255.82	270.32	221.22	37.65	0.7072	0.58	0.61	0.70	0.50
38.65	412.36	292.86	264.57	282.04	226.83	38.65	0.7659	0.66	0.67	0.77	0.54
39.65	428.59	304.25	274.51	290.61	235.06	39.65	0.8354	0.72	0.73	0.82	0.59
a	202.27	143.91	127.79	145.25	97.72						
b	2.24	2.75	2.63	2.52	2.64						
x0	34.06	34.74	34.38	34.45	34.55						
y0	233.52	176.50	158.64	159.28	147.53						
R	1.00	1.00	1.00	1.00	1.00						
**$CP_{(SDM)}$**	31.10	31.12	30.92	31.14	31.07						

Well/ Cycle	D8	D9	E2	E3	E4
0.56	126.48	107.73	77.35	81.84	95.76
1.56	127.66	109.64	80.67	82.58	97.44
2.56	122.9	104.99	81.64	80.87	97.13
3.56	122.96	108.51	81.82	80.26	96.98
4.56	122.54	107.26	80.77	80.42	98.35
5.56	116.28	106.61	82.24	80.13	98.22
6.56	117.12	104.13	81.97	78.89	96.52
7.56	118.81	107.69	81.47	79.48	97.74
8.56	121.07	104.28	82.09	79.11	97.92
9.56	122.44	109.15	82.82	79.38	98.03
10.56	117.4	105.78	81.95	78.49	97.46
11.56	117.63	105.54	81.62	79.48	96.83
12.56	117.56	104.75	81.5	79.38	97.23
13.56	117.45	104	81.58	78.61	98.11
14.56	114.74	102.09	82.54	79.24	99.06
15.56	115.24	104.2	82.91	78.95	99.51
16.58	115.06	99.62	82.11	77.48	97.05
17.58	112.19	102.09	82.4	78.13	97.91
18.58	115	104.23	82.32	79.18	97.82
19.58	111.5	103.89	82.63	78.9	98.82
20.58	114.34	102.37	82.7	79.62	96.95
21.58	112.36	101.49	82.58	79.33	98.31
22.58	107.8	99.97	83.05	78.35	98.43
23.58	116.73	103.35	83.97	80.32	99.59
24.58	115.46	105.01	85.53	79.99	100.47
25.58	120.62	108.74	86.86	81.69	104.95
26.58	121.55	109.46	89.15	83.54	107.69
27.58	135.31	121.35	93.89	87.41	113.51
28.56	152.56	130.17	101.95	92.9	122.57
29.61	178.31	155.91	112.88	103.04	135.1
30.56	210.34	181.25	125.3	113.26	155.97
31.63	259.26	211.59	144.22	130.1	177.57
32.63	300.17	241.04	161.75	145.83	195.42
33.63	353.5	281.14	177.67	164.72	221.34
34.63	396.2	314.24	194.93	180.56	242.78
35.63	431.77	339.9	206.39	193.19	259.78
36.63	472.63	366.88	220.28	208.07	274.96
37.65	505.36	395.4	237.16	221.83	292.1
38.65	540.54	423.56	246.93	232.66	308.39
39.65	565.24	437.01	260.25	242.4	323.22

Parameter	D8	D9	E2	E3	E4
a	468.23	353.25	190.81	172.86	238.75
b	2.2527	2.3392	2.5808	2.3386	2.5107
x0	33.729	33.75	33.684	33.833	33.558
y0	115.75	103.34	81.133	78.911	96.868
R	0.9987	0.9989	0.9992	0.9993	0.9993
**$CP_{(SDM)}$**	30.76	30.67	30.29	30.75	30.25

Well/ Cycle	Fluorescence Data Normalized To Y0 D8	D9	E2	E3	E4
0.56	0.09	0.04	-0.05	0.04	-0.01
1.56	0.10	0.06	-0.01	0.05	0.01
2.56	0.06	0.02	0.01	0.02	0.00
3.56	0.06	0.05	0.01	0.02	0.00
4.56	0.06	0.04	0.00	0.02	0.02
5.56	0.00	0.03	0.01	0.02	0.01
6.56	0.01	0.01	0.01	0.00	0.00
7.56	0.03	0.04	0.00	0.01	0.01
8.56	0.05	0.01	0.01	0.00	0.01
9.56	0.06	0.06	0.02	0.01	0.01
10.56	0.01	0.02	0.01	-0.01	0.01
11.56	0.02	0.02	0.01	0.01	0.00
12.56	0.02	0.01	0.00	0.01	0.00
13.56	0.01	0.01	0.01	0.00	0.01
14.56	-0.01	-0.01	0.02	0.00	0.02
15.56	0.00	0.01	0.02	0.00	0.03
16.58	-0.01	-0.04	0.01	-0.02	0.00
17.58	-0.03	-0.01	0.02	-0.01	0.01
18.58	-0.01	0.01	0.01	0.00	0.01
19.58	-0.04	0.01	0.02	0.00	0.02
20.58	-0.01	-0.01	0.02	0.01	0.00
21.58	-0.03	-0.02	0.02	0.01	0.01
22.58	-0.07	-0.03	0.02	-0.01	0.02
23.58	0.01	0.00	0.03	0.02	0.03
24.58	0.00	0.02	0.05	0.01	0.04
25.58	0.04	0.05	0.07	0.04	0.08
26.58	0.05	0.06	0.10	0.06	0.11
27.58	0.17	0.17	0.16	0.11	0.17
28.56	0.32	0.26	0.26	0.18	0.27
29.61	0.54	0.51	0.39	0.31	0.39
30.56	0.82	0.75	0.54	0.44	0.61
31.63	1.24	1.05	0.78	0.65	0.83
32.63	1.59	1.33	0.99	0.85	1.02
33.63	2.05	1.72	1.19	1.09	1.28
34.63	2.42	2.04	1.40	1.29	1.51
35.63	2.73	2.29	1.54	1.45	1.68
36.63	3.08	2.55	1.72	1.64	1.84
37.65	3.37	2.83	1.92	1.81	2.02
38.65	3.67	3.10	2.04	1.95	2.18
39.65	3.88	3.23	2.21	2.07	2.34

D8			D9			E2	
Well/ Cycle	FAM Normalized Fluorescence	VIC Normalized Fluorescence	Well/ Cycle	FAM Normalized Fluorescence	VIC Normalized Fluorescence	Well/ Cycle	FAM Normalized Fluorescence
0.56	-0.015966615	0.092687512	0.56	-0.056447702	0.042443806	0.56	-0.071915662
1.56	0.003903331	0.102881782	1.56	-0.00925309	0.060925823	1.56	0.001773843
2.56	0.022017528	0.061759134	2.56	-0.007213467	0.015930337	2.56	-0.003521211
3.56	0.023559161	0.062277486	3.56	-0.006816874	0.049991436	3.56	-0.007996793
4.56	0.045184857	0.058649017	4.56	0.018055197	0.037895876	4.56	0.00927517
5.56	0.008956464	0.004567551	5.56	-0.004267345	0.031606184	5.56	0.001017407
6.56	0.011268914	0.011824489	6.56	0.022077787	0.007608592	6.56	0.017154715
7.56	0.012082554	0.026424757	7.56	0.01278617	0.042056749	7.56	0.004673516
8.56	0.014352182	0.045949376	8.56	0.003211273	0.009060059	8.56	0.015011479
9.56	0.009427519	0.057785096	9.56	0.00355121	0.056184363	9.56	0.013183424
10.56	0.017906504	0.014243469	10.56	0.024457347	0.023574732	10.56	0.017722042
11.56	0.00904211	0.016230487	11.56	-0.001377879	0.021252384	11.56	0.002215098
12.56	0.012724902	0.015625742	12.56	0.016242198	0.01360799	12.56	0.014948443
13.56	0.001205472	0.014675429	13.56	0.00400446	0.006350653	13.56	0.004736552
14.56	0.010283982	-0.008736835	14.56	0.017998541	-0.012131364	14.56	0.021378151
15.56	0.007157891	-0.004417229	15.56	-0.001491191	0.008285943	15.56	0.011229297
16.58	0.015636877	-0.005972287	16.58	0.008083706	-0.036032192	16.58	0.01620917
17.58	-0.002777082	-0.030766825	17.58	0.014485856	-0.012131364	17.58	0.008707843
18.58	0.007200714	-0.00649064	18.58	0.020774694	0.008576236	18.58	0.013183424
19.58	-0.011684299	-0.036727881	19.58	0.00281468	0.005286244	19.58	-0.006231775
20.58	-0.001106979	-0.01219252	20.58	0.018111853	-0.009421958	20.58	-0.000684575
21.58	-0.019007059	-0.029298159	21.58	0.01561898	-0.017937233	21.58	-0.003142993
22.58	-0.019777876	-0.068692965	22.58	0.008990205	-0.032645435	22.58	0.004673516
23.58	-0.017165664	0.008455197	23.58	-0.015485272	6.09616E-05	23.58	0.005429952
24.58	-0.018321889	-0.002516602	24.58	0.00445771	0.016123866	24.58	0.008140516
25.58	-0.025944411	0.042061731	25.58	0.00638402	0.05221702	25.58	0.00927517
26.58	-0.001792149	0.050096198	26.58	0.020491413	0.059184063	26.58	0.016713461
27.58	0.005744727	0.168971752	27.58	0.016865417	0.174237036	27.58	0.037137242
28.56	0.047754246	0.317998156	28.56	0.062756936	0.259583313	28.56	0.067835949
29.61	0.080000086	0.540457861	29.61	0.106608832	0.508655099	29.61	0.10332542
30.56	0.192025471	0.817171816	30.56	0.175672736	0.753856307	30.56	0.191639361
31.63	0.22941009	1.239802058	31.63	0.213519075	1.047439757	31.63	0.205129143
32.63	0.365887499	1.593232214	32.63	0.282073073	1.332411168	32.63	0.302268174
33.63	0.383958873	2.053961381	33.63	0.342808516	1.720436756	33.63	0.349734554
34.63	0.472645636	2.422855726	34.63	0.387113661	2.040727204	34.63	0.40470226
35.63	0.565443423	2.730152491	35.63	0.468245335	2.289024875	35.63	0.492511911
36.63	0.629121461	3.083150686	36.63	0.52342847	2.550095458	36.63	0.558258835
37.65	0.707188081	3.365912089	37.65	0.579404793	2.826067772	37.65	0.612596178
38.65	0.76585581	3.669839561	38.65	0.659233374	3.098556564	38.65	0.667752993
39.65	0.835357798	3.883228093	39.65	0.723764782	3.228704798	39.65	0.730411136

periment derived	linear regress
parameter	value
y0	-0.0177
a	0.2122
R	0.9805126

periment derived	linear regress
parameter	value
y0	0.0017
a	0.205
R	0.9936369

periment derived	linear regress
parameter	value
y0	-0.0065
a	0.3021
R	0.98881082

VIC Normalized Fluorescence	Well/ Cycle	E3 FAM Normalized Fluorescence	E3 VIC Normalized Fluorescence	E4 FAM Normalized Fluorescence	E4 VIC Normalized Fluorescence
-0.046628318	0.56	-0.047924582	0.037120394	-0.025024418	-0.011433143
-0.005707905	1.56	-0.02387942	0.046498071	-0.020347464	0.005910135
0.006247758	2.56	-0.008623612	0.024828034	0.002495054	0.002709887
0.008466335	3.56	-0.018040778	0.017097786	0.002698399	0.00116138
-0.004475362	4.56	0.01316143	0.019125392	0.002901745	0.015304411
0.013643014	5.56	0.003681484	0.015450357	0.003376219	0.013962371
0.010315149	6.56	-0.000650412	-0.000263589	0.021948474	-0.003587375
0.004152436	7.56	0.013286993	0.007213208	0.011442271	0.009007149
0.0117942	8.56	0.012094152	0.002524369	0.003172873	0.010865357
0.020791761	9.56	0.014605396	0.005945954	-0.002588593	0.012000929
0.01006864	10.56	0.00970847	-0.005332603	-0.003469758	0.006116603
0.00600125	11.56	0.01542155	0.007213208	0.01801712	-0.000387127
0.004522199	12.56	0.018121137	0.005945954	0.009883286	0.003742225
0.005508233	13.56	0.00914344	-0.003811899	-0.009027878	0.012826799
0.017340641	14.56	0.010210718	0.004171799	0.003918475	0.02263401
0.021901049	15.56	0.00286533	0.000496763	-0.002114119	0.027279531
0.012040708	16.58	0.024399248	-0.018131865	0.020728398	0.001884017
0.015615082	17.58	0.00970847	-0.009894717	-0.002385247	0.010762124
0.014629048	18.58	0.018435043	0.003411447	0.013882421	0.009833019
0.01844993	19.58	-0.000462069	-0.000136863	-1.28786E-05	0.020156399
0.01931271	20.58	0.002425862	0.008987363	0.002833963	0.000851679
0.017833659	21.58	-0.004605622	0.005312327	0.008798775	0.014891475
0.023626609	22.58	0.012910306	-0.007106759	0.001003851	0.016130281
0.034966	23.58	-0.006551836	0.017858139	-0.005706562	0.028105402
0.054193664	24.58	0.007950599	0.013676201	-0.000148442	0.037189976
0.070586481	25.58	0.018435043	0.035219514	0.022490729	0.083438718
0.098811706	26.58	0.023583093	0.058663706	0.027303248	0.111724779
0.157234224	27.58	0.022578596	0.107706423	0.019237195	0.17180685
0.256577155	28.56	0.081027802	0.177278649	0.061736479	0.265336671
0.391294059	29.61	0.101494441	0.30577817	0.0909505	0.394688621
0.544375847	30.56	0.184114372	0.435291494	0.135822151	0.610137559
0.777572902	31.63	0.223792029	0.64869701	0.156495646	0.833122564
0.993637615	32.63	0.327129723	0.84803601	0.237359513	1.017394895
1.189858393	33.63	0.38959692	1.087420226	0.273487293	1.284976901
1.402595242	34.63	0.453194177	1.288153206	0.331576425	1.506310166
1.543844621	35.63	0.565446788	1.448207343	0.394410294	1.681807624
1.715044784	36.63	0.62822789	1.636774687	0.451550478	1.83851653
1.923097971	37.65	0.69709876	1.811148791	0.499472318	2.015459261
2.043517381	38.65	0.770678212	1.948392362	0.537497992	2.18362712
2.207692052	39.65	0.824481616	2.071822868	0.593282538	2.336722843

periment derived parameter	linear regress: value	SD
y0	0.0008	0.0131
a	0.3667	0.0214
**R**	**0.9916244**	

Experiment derived parameter	linear regression value
y0	-0.0051
a	0.2208
**R**	**0.98795002**

AVERAGE	**value**
y0	-0.00536
a	0.26136
**R**	

Well/ Cycle	Raw fluorescence data E5	E6	E7	E8	E9	Well/ Cycle	E5	E6	E7	E8	E9
0.56	165.75	171.98	194.61	166.58	178.83	0.56	-0.0303	-0.02	0.01	-0.05	0.01
1.56	169.23	175.63	192.33	169.18	178.59	1.56	-0.0099	0.00	0.00	-0.04	0.01
2.56	171.12	176.73	192.18	172.85	177.98	2.56	0.0011	0.01	0.00	-0.02	0.00
3.56	172.44	175.18	196.05	170.07	179.90	3.56	0.0088	0.00	0.02	-0.03	0.01
4.56	173.37	177.80	195.32	174.26	181.59	4.56	0.0143	0.01	0.01	-0.01	0.02
5.56	173.26	175.74	194.86	172.85	180.21	5.56	0.0136	0.00	0.01	-0.02	0.02
6.56	172.02	177.80	194.15	176.65	178.41	6.56	0.0064	0.01	0.01	0.00	0.01
7.56	172.47	179.11	196.67	176.70	178.79	7.56	0.0090	0.02	0.02	0.00	0.01
8.56	174.60	179.77	195.38	178.14	177.86	8.56	0.0215	0.02	0.01	0.01	0.00
9.56	171.04	176.31	197.36	179.28	176.29	9.56	0.0007	0.00	0.02	0.02	-0.01
10.56	172.32	179.87	195.44	179.28	178.71	10.56	0.0081	0.02	0.01	0.02	0.01
11.56	174.14	175.25	193.99	177.07	179.11	11.56	0.0188	0.00	0.00	0.01	0.01
12.56	173.80	180.60	197.20	177.54	181.01	12.56	0.0168	0.03	0.02	0.01	0.02
13.56	170.00	174.86	193.36	177.76	177.94	13.56	-0.0054	0.00	0.00	0.01	0.00
14.56	173.12	180.35	197.57	180.44	179.48	14.56	0.0128	0.03	0.02	0.03	0.01
15.56	172.18	175.33	189.57	178.68	180.85	15.56	0.0073	0.00	-0.02	0.02	0.02
16.58	170.82	175.77	194.00	181.10	177.84	16.58	-0.0006	0.00	0.01	0.03	0.00
17.58	169.27	173.54	191.70	180.37	178.66	17.58	-0.0097	-0.01	-0.01	0.03	0.01
18.58	171.46	177.14	195.94	179.96	175.61	18.58	0.0031	0.01	0.02	0.02	-0.01
19.58	169.58	171.79	191.83	178.56	175.49	19.58	-0.0079	-0.02	-0.01	0.02	-0.01
20.58	171.41	174.16	192.19	180.08	176.41	20.58	0.0028	-0.01	0.00	0.02	0.00
21.58	167.54	172.06	188.82	180.47	173.58	21.58	-0.0198	-0.02	-0.02	0.03	-0.02
22.58	173.48	173.49	188.73	183.47	175.99	22.58	0.0149	-0.01	-0.02	0.04	-0.01
23.58	168.03	173.05	190.76	180.83	173.56	23.58	-0.0170	-0.01	-0.01	0.03	-0.02
24.58	170.92	174.51	188.44	182.56	173.06	24.58	0.0000	-0.01	-0.02	0.04	-0.02
25.58	170.27	175.29	188.54	183.67	175.29	25.58	-0.0039	0.00	-0.02	0.04	-0.01
26.58	170.39	175.53	193.78	186.92	178.62	26.58	-0.0031	0.00	0.00	0.06	0.01
27.58	175.06	176.41	194.92	185.73	176.81	27.58	0.0242	0.01	0.01	0.06	0.00
28.56	176.54	182.12	202.22	192.49	185.69	28.56	0.0328	0.04	0.05	0.09	0.05
29.61	182.23	182.65	202.74	193.46	189.68	29.61	0.0661	0.04	0.05	0.10	0.07
30.56	191.06	192.34	215.11	204.06	202.39	30.56	0.1178	0.10	0.11	0.16	0.14
31.63	190.79	196.39	215.44	211.45	207.45	31.63	0.1162	0.12	0.12	0.20	0.17
32.63	202.85	213.14	234.77	223.27	218.48	32.63	0.1868	0.21	0.22	0.27	0.23
33.63	207.66	212.51	236.26	225.03	223.68	33.63	0.2149	0.21	0.22	0.28	0.26
34.63	218.28	223.72	248.34	234.47	234.58	34.63	0.2770	0.27	0.29	0.33	0.32
35.63	229.11	238.31	259.42	244.98	245.04	35.63	0.3404	0.36	0.34	0.39	0.38
36.63	230.73	245.64	271.24	251.41	254.53	36.63	0.3499	0.40	0.41	0.43	0.44
37.65	241.37	255.18	279.67	263.67	265.17	37.65	0.4121	0.45	0.45	0.50	0.50
38.65	246.71	266.95	290.05	271.17	275.23	38.65	0.4434	0.52	0.50	0.54	0.55
39.65	256.47	272.54	300.66	282.58	284.27	39.65	0.5005	0.55	0.56	0.61	0.60
Parameter											
a	92.2695	109.1196	118.7074	152.9854	117.6231						
b	2.4755	2.3676	2.4334	3.9397	2.5096						
x0	34.5634	35.0125	34.9120	36.5032	34.6783						
y0	170.9283	175.5163	193.0256	175.8413	177.2635						
R	0.9949	0.9947	0.9940	0.9947	0.9949						
$CP_{(SDM)}$	31.3032	31.8944	31.7072	31.3146	31.3732						

Well/ Cycle	E5	E6	E7	E8	E9	Well/ Cycle	E5	E6	E7	E8	E9
							Fluorescence Data Normalized To Y0				
0.56	99.49	113.83	118.27	106.79	97.99	0.56	-0.01	0.02	0.01	-0.02	0.01
1.56	100.74	114.94	120.97	108.98	100.59	1.56	0.00	0.03	0.03	0.00	0.04
2.56	100.39	113.78	118.87	107.47	98.44	2.56	0.00	0.02	0.02	-0.01	0.01
3.56	99.26	116.32	121.26	109.45	100.54	3.56	-0.01	0.05	0.04	0.01	0.03
4.56	100.54	117.27	121.91	107.31	101.27	4.56	0.00	0.05	0.04	-0.01	0.04
5.56	100.66	116.39	119.83	109.73	99.48	5.56	0.00	0.05	0.02	0.01	0.02
6.56	100.01	114.62	120.86	108.97	99.44	6.56	-0.01	0.03	0.03	0.00	0.02
7.56	100.96	114.94	119.83	108.9	99.46	7.56	0.00	0.03	0.02	0.00	0.02
8.56	100.85	113.65	119.98	107.22	96.55	8.56	0.00	0.02	0.02	-0.01	-0.01
9.56	101.11	114.97	119.21	110.3	98.93	9.56	0.01	0.03	0.02	0.01	0.02
10.56	101.92	111.59	119.14	111.12	100.08	10.56	0.01	0.00	0.02	0.02	0.03
11.56	101.62	113.05	118.36	110.02	98.39	11.56	0.01	0.02	0.01	0.01	0.01
12.56	100.89	110.39	116.9	110.03	98.53	12.56	0.00	-0.01	0.00	0.01	0.01
13.56	101.67	112.03	119.01	111.2	98.63	13.56	0.01	0.01	0.02	0.02	0.02
14.56	102.14	109.92	117.71	110.28	95.42	14.56	0.02	-0.01	0.01	0.01	-0.02
15.56	103.97	112.33	118.24	111.04	97.11	15.56	0.03	0.01	0.01	0.02	0.00
16.58	102.1	110.09	117.71	111.47	98.25	16.58	0.02	-0.01	0.01	0.02	0.01
17.58	102.46	109.46	117.46	111.23	96.94	17.58	0.02	-0.02	0.00	0.02	0.00
18.58	101.75	110.48	117.61	110	97.43	18.58	0.01	-0.01	0.00	0.01	0.00
19.58	103.15	110.1	116.9	111.21	98.3	19.58	0.03	-0.01	0.00	0.02	0.01
20.58	103.36	109.98	115.15	111.62	96.55	20.58	0.03	-0.01	-0.02	0.03	-0.01
21.58	103.84	111.5	116.69	112.19	96.43	21.58	0.03	0.00	0.00	0.03	-0.01
22.58	102.69	110.73	118.75	113.19	97.11	22.58	0.02	0.00	0.01	0.04	0.00
23.58	104.12	112.8	115.54	112.4	97.34	23.58	0.04	0.01	-0.01	0.03	0.00
24.58	104.51	112.42	118.71	115.19	99.7	24.58	0.04	0.01	0.01	0.06	0.03
25.58	107.01	113.62	123.17	119.74	103.46	25.58	0.06	0.02	0.05	0.10	0.06
26.58	109.07	120.78	127.03	125	105.72	26.58	0.08	0.09	0.09	0.15	0.09
27.58	118.26	129.83	137.02	132.97	114.19	27.58	0.18	0.17	0.17	0.22	0.18
28.56	129.58	144.61	149.65	147.94	125.25	28.56	0.29	0.30	0.28	0.36	0.29
29.61	146.93	167.86	176.95	170.06	146.99	29.61	0.46	0.51	0.51	0.56	0.51
30.56	164.06	195.51	206.94	193.78	166.32	30.56	0.63	0.76	0.77	0.78	0.71
31.63	191.93	233.94	248.03	228.92	192.5	31.63	0.91	1.10	1.12	1.10	0.98
32.63	217.08	269.41	287.34	265.15	218.93	32.63	1.16	1.42	1.45	1.44	1.25
33.63	247.75	315.98	334.4	305.87	249.33	33.63	1.46	1.84	1.86	1.81	1.57
34.63	271.33	348.62	370.69	342.56	278.57	34.63	1.70	2.14	2.17	2.15	1.87
35.63	291.35	380.23	403.71	366.1	297.1	35.63	1.90	2.42	2.45	2.37	2.06
36.63	313.18	407.16	439.39	395.87	315.24	36.63	2.12	2.66	2.75	2.64	2.24
37.65	333.05	440.03	466.29	422.26	341.39	37.65	2.31	2.96	2.98	2.88	2.51
38.65	351.4	461.29	492.88	445.79	359.08	38.65	2.50	3.15	3.21	3.10	2.70
39.65	366.75	483.73	515.38	470.58	374.32	39.65	2.65	3.35	3.40	3.33	2.85
Parameter											
a	280.72	385.91	412.89	381.09	290.0358						
b	2.4326	2.2603	2.2473	2.4474	2.3875						
x0	33.574	33.545	33.549	33.622	33.5004						
y0	100.53	111.18	117.07	108.78	97.1572						
R	0.9994	0.9991	0.9993	0.9995	0.999095						
$CP_{(SDM)}$	30.37	30.57	30.59	30.40	30.36						

E5			E6			
Well/ Cycle	FAM Normalized Fluorescence	VIC Normalized Fluorescence	Well/ Cycle	FAM Normalized Fluorescence	VIC Normalized Fluorescence	Well/ Cycle
0.56	-0.030295159	-0.010352062	0.56	-0.020147986	0.023818647	0.56
1.56	-0.009935745	0.002081951	1.56	0.000647803	0.033802295	1.56
2.56	0.001121523	-0.001399572	2.56	0.006915027	0.023368933	2.56
3.56	0.008844059	-0.01263992	3.56	-0.001916061	0.046214398	3.56
4.56	0.014284937	9.25091E-05	4.56	0.013011327	0.054758962	4.56
5.56	0.013641392	0.001286174	5.56	0.001274525	0.046843998	5.56
6.56	0.006386889	-0.005179512	6.56	0.013011327	0.030924126	6.56
7.56	0.009019571	0.004270337	7.56	0.020475021	0.033802295	7.56
8.56	0.021480937	0.003176144	8.56	0.024235356	0.022199677	8.56
9.56	0.00065349	0.005762419	9.56	0.004522087	0.034072123	9.56
10.56	0.00814201	0.013819659	10.56	0.024805104	0.003671464	10.56
11.56	0.01878975	0.010835496	11.56	-0.001517238	0.01680311	11.56
12.56	0.016800612	0.003574033	12.56	0.028964261	-0.007121669	12.56
13.56	-0.005430932	0.011332857	13.56	-0.003739254	0.007628946	13.56
14.56	0.012822335	0.016008045	14.56	0.027539892	-0.01134898	14.56
15.56	0.007322954	0.03421144	15.56	-0.00106144	0.01032723	15.56
16.58	-0.000633599	0.015610157	16.58	0.00144545	-0.009819953	16.58
17.58	-0.009701729	0.019191153	17.58	-0.011259923	-0.015486348	17.58
18.58	0.003110661	0.012128633	18.58	0.009250993	-0.006312184	18.58
19.58	-0.007888103	0.026054728	19.58	-0.021230507	-0.00973001	19.58
20.58	0.002818141	0.028143642	20.58	-0.007727487	-0.010809323	20.58
21.58	-0.019822932	0.032918303	21.58	-0.019692188	0.002861979	21.58
22.58	0.014928482	0.021479011	22.58	-0.011544797	-0.004063615	22.58
23.58	-0.016956233	0.035703521	23.58	-0.014051686	0.01455454	23.58
24.58	-4.85584E-05	0.039582933	24.58	-0.005733371	0.011136715	24.58
25.58	-0.003851322	0.064450959	25.58	-0.001289339	0.021929848	25.58
26.58	-0.003149274	0.084942212	26.58	7.80554E-05	0.086328878	26.58
27.58	0.024172124	0.176357073	27.58	0.005091835	0.167727092	27.58
28.56	0.032830725	0.288959492	28.56	0.037624426	0.300662519	28.56
29.61	0.066119537	0.461543588	29.61	0.040644088	0.509779478	29.61
30.56	0.117778624	0.631939298	30.56	0.095852636	0.758471261	30.56
31.63	0.116199014	0.909168045	31.63	0.118927416	1.104121358	31.63
32.63	0.186754914	1.159340381	32.63	0.214360148	1.423148393	32.63
33.63	0.214895368	1.464421316	33.63	0.210770738	1.842011912	33.63
34.63	0.277026683	1.698976532	34.63	0.274639449	2.135585141	34.63
35.63	0.340386583	1.898119679	35.63	0.357765632	2.419894263	35.63
36.63	0.349864241	2.115267277	36.63	0.399528135	2.662110166	36.63
37.65	0.412112564	2.312918342	37.65	0.453882061	2.957752078	37.65
38.65	0.443353734	2.495449649	38.65	0.52094136	3.148970425	38.65
39.65	0.500453699	2.648139325	39.65	0.552790254	3.35080202	39.65

periment derived	linear regress
parameter	value
y0	-0.0023
a	0.1521
R	0.98058337

periment derived	linear regress
parameter	value
y0	-0.0119
a	0.1321
R	0.96964032

Experiment derived
parameter
y0
a
R

E7			E8		E9	
FAM Normalized Fluorescence	VIC Normalized Fluorescence	Well/ Cycle	FAM Normalized Fluorescence	VIC Normalized Fluorescence	FAM Normalized Fluorescence	VIC Normalized Fluorescence
0.008208238	0.010220938	0.56	-0.052668514	-0.018273949	0.008837127	0.008571676
-0.003603667	0.033283393	1.56	-0.037882454	0.001858835	0.00748321	0.03533243
-0.004380766	0.015345928	2.56	-0.017011362	-0.012022674	0.004042005	0.013203345
0.015668388	0.035760472	3.56	-0.032821072	0.00617957	0.014873338	0.0348178
0.011886506	0.041312544	4.56	-0.008992768	-0.013493562	0.024407168	0.042331397
0.009503403	0.023545912	5.56	-0.017011362	0.008753624	0.016622147	0.023907647
0.005825134	0.032343812	6.56	0.004599033	0.001766905	0.006467773	0.023495943
0.018880397	0.023545912	7.56	0.004883381	0.001123391	0.008611474	0.023701795
0.012197346	0.02482716	8.56	0.013072583	-0.014320937	0.003365047	-0.006249665
0.022455053	0.018250089	9.56	0.019555702	0.013993664	-0.005491824	0.018246718
0.012508185	0.017652174	10.56	0.019555702	0.021531967	0.008160168	0.030083205
0.004996228	0.010989687	11.56	0.006987551	0.01141961	0.010416696	0.012688715
0.021626147	-0.001481122	12.56	0.009660415	0.01151154	0.021135203	0.014129679
0.001732413	0.016541759	13.56	0.010911544	0.022267411	0.003816352	0.015158938
0.023542991	0.005437614	14.56	0.026152559	0.013809803	0.012503984	-0.017880301
-0.017902289	0.009964689	15.56	0.016143534	0.020796523	0.020232592	-0.000485811
0.005048035	0.005437614	16.58	0.029905944	0.024749535	0.003252221	0.011247751
-0.006867483	0.003302202	17.58	0.025754473	0.022543203	0.007878102	-0.002235552
0.015098515	0.004583449	18.58	0.023422825	0.011235748	-0.009327921	0.002807821
-0.006193997	-0.001481122	19.58	0.0154611	0.022359342	-0.01000488	0.011762381
-0.004328959	-0.016429009	20.58	0.024105259	0.026128493	-0.004814866	-0.006249665
-0.021787784	-0.003274869	21.58	0.026323168	0.031368533	-0.0207798	-0.007484777
-0.022254043	0.01432093	22.58	0.043384006	0.040561585	-0.00718422	-0.000485811
-0.011737303	-0.013097766	23.58	0.028370468	0.033299074	-0.020892626	0.001881487
-0.023756434	0.013979264	24.58	0.038208885	0.05894769	-0.023713286	0.026172018
-0.023238368	0.052075023	25.58	0.044521395	0.100776077	-0.011133144	0.064872187
0.00390829	0.085045792	26.58	0.06300397	0.149131532	0.00765245	0.088133458
0.009814242	0.170376875	27.58	0.056236504	0.222400159	-0.002558338	0.175311763
0.04763306	0.278257913	28.56	0.09468026	0.360020151	0.047536577	0.289147896
0.050327003	0.511444957	29.61	0.100196598	0.563370467	0.070045441	0.512908976
0.114411767	0.767609038	30.56	0.160478227	0.781429667	0.141746609	0.711864895
0.116121385	1.118585434	31.63	0.202504759	1.104473523	0.170291684	0.98132511
0.216263542	1.454357694	32.63	0.269724462	1.437537806	0.232515436	1.253358475
0.223982726	1.85632774	33.63	0.279733487	1.811878894	0.261850296	1.566253453
0.286565098	2.166304216	34.63	0.333418258	2.149171982	0.323340676	1.867209018
0.343966811	2.448349497	35.63	0.393188062	2.365576432	0.382348876	2.057930858
0.405202212	2.753115567	36.63	0.429755126	2.639253598	0.435884996	2.244638586
0.448875175	2.98288595	37.65	0.499477085	2.881858247	0.495908633	2.513790023
0.502650426	3.210008422	38.65	0.542129181	3.098170766	0.552660305	2.695866081
0.557617228	3.402195546	39.65	0.607017237	3.326066532	0.603657831	2.852725274

d linear regression
value
-0.0023
0.1291
0.96857666

periment derived	linear regress	SD
parameter	value	SD
y0	0.0331	0.0133
a	0.1483	0.0127
R	0.98227572	

Experiment derived	linear regression
parameter	value
y0	-0.0167
a	0.1892
R	0.98612443

AVERAGE	value	SD
y0	-2E-05	
a	0.15016	0.023984537

Well/ Cycle	Raw fluorescence data F2	F3	F4	F5	F6	Well/ Cycle	F2	F3	F4	F5	F6
0.56	155.03	155.67	158.16	151.83	164.19	0.56	-0.0337	-0.03	-0.03	-0.02	-0.02
1.56	160.65	158.58	160.86	150.38	165.45	1.56	0.0013	-0.02	-0.01	-0.03	-0.01
2.56	159.81	161.00	165.51	154.79	168.27	2.56	-0.0039	0.00	0.02	0.00	0.00
3.56	162.37	161.58	162.66	154.00	166.82	3.56	0.0121	0.00	0.00	0.00	0.00
4.56	163.90	163.11	163.69	155.72	170.50	4.56	0.0216	0.01	0.01	0.01	0.02
5.56	162.43	163.05	164.09	155.58	170.37	5.56	0.0124	0.01	0.01	0.01	0.02
6.56	162.01	164.46	166.27	155.19	169.50	6.56	0.0098	0.02	0.02	0.01	0.01
7.56	160.84	161.83	165.07	155.65	170.79	7.56	0.0025	0.00	0.02	0.01	0.02
8.56	164.22	161.80	166.81	155.72	170.63	8.56	0.0236	0.00	0.03	0.01	0.02
9.56	161.96	160.89	161.82	155.74	168.77	9.56	0.0095	0.00	0.00	0.01	0.01
10.56	163.33	162.74	163.36	154.90	168.76	10.56	0.0180	0.01	0.00	0.00	0.01
11.56	159.93	160.98	161.87	155.30	168.15	11.56	-0.0031	0.00	0.00	0.01	0.00
12.56	162.45	162.47	166.14	153.66	167.35	12.56	0.0126	0.01	0.02	0.00	0.00
13.56	159.31	159.62	165.02	152.49	165.67	13.56	-0.0070	-0.01	0.01	-0.01	-0.01
14.56	164.06	163.44	163.13	157.29	170.17	14.56	0.0226	0.01	0.00	0.02	0.02
15.56	158.31	161.24	161.33	154.34	167.04	15.56	-0.0132	0.00	-0.01	0.00	0.00
16.58	161.50	161.14	164.87	154.80	169.62	16.58	0.0066	0.00	0.01	0.00	0.01
17.58	159.90	161.72	159.47	154.97	168.24	17.58	-0.0033	0.00	-0.02	0.00	0.00
18.58	161.40	164.53	164.62	156.09	169.91	18.58	0.0060	0.02	0.01	0.01	0.01
19.58	159.43	163.12	160.61	153.79	166.45	19.58	-0.0063	0.01	-0.01	0.00	-0.01
20.58	159.29	160.36	161.12	154.24	166.23	20.58	-0.0071	0.00	-0.01	0.00	-0.01
21.58	159.67	160.30	160.10	154.08	164.25	21.58	-0.0048	-0.01	-0.02	0.00	-0.02
22.58	160.15	161.34	162.53	155.16	167.90	22.58	-0.0018	0.00	0.00	0.01	0.00
23.58	159.51	161.00	159.24	154.06	164.16	23.58	-0.0058	0.00	-0.02	0.00	-0.02
24.58	159.22	160.93	163.57	155.72	167.39	24.58	-0.0076	0.00	0.01	0.01	0.00
25.58	159.06	159.89	161.51	153.33	164.37	25.58	-0.0086	-0.01	-0.01	-0.01	-0.02
26.58	161.57	161.97	165.35	155.06	169.25	26.58	0.0071	0.01	0.02	0.00	0.01
27.58	158.47	162.38	165.40	155.50	169.94	27.58	-0.0122	0.01	0.02	0.01	0.01
28.56	161.65	167.49	168.52	158.58	174.31	28.56	0.0076	0.04	0.04	0.03	0.04
29.61	166.88	165.40	166.80	157.86	175.31	29.61	0.0402	0.03	0.03	0.02	0.05
30.56	173.84	176.53	177.49	165.34	181.23	30.56	0.0836	0.10	0.09	0.07	0.08
31.63	176.81	175.72	177.18	166.74	185.89	31.63	0.1021	0.09	0.09	0.08	0.11
32.63	185.11	183.21	190.63	175.88	194.79	32.63	0.1538	0.14	0.17	0.14	0.16
33.63	188.06	187.93	191.06	177.08	197.50	33.63	0.1722	0.17	0.17	0.15	0.18
34.63	198.49	192.80	199.65	182.57	206.64	34.63	0.2372	0.20	0.23	0.18	0.23
35.63	203.68	202.77	206.83	186.16	212.38	35.63	0.2695	0.26	0.27	0.21	0.27
36.63	209.29	207.96	212.38	195.05	217.42	36.63	0.3045	0.29	0.31	0.26	0.30
37.65	219.89	215.74	219.38	198.53	225.88	37.65	0.3706	0.34	0.35	0.29	0.35
38.65	226.70	220.38	227.68	203.46	232.28	38.65	0.4130	0.37	0.40	0.32	0.39
39.65	229.45	227.04	230.78	207.52	237.94	39.65	0.4302	0.41	0.42	0.34	0.42
Parameter											
a	77.14	76.26	76.61	59.73	78.07						
b	2.31	2.57	2.45	2.48	2.51						
x0	34.88	35.27	34.80	34.85	34.75						
y0	160.44	161.16	162.62	154.32	167.50						
R	0.99	0.99	0.99	0.99	0.99						
**$CP_{(SDM)}$**	31.84	31.89	31.58	31.58	31.44						

Well/ Cycle	F2	F3	F4	F5	F6	Well/ Cycle	F2	F3	F4	F5	F6
							Fluorescence Data Normalized To Y0				
0.56	79.76	81.4	87.15	84.48	90.63	0.56	-0.1343	-0.03	0.01	0.04	0.14
1.56	81.31	82.65	88.44	84.89	93.77	1.56	-0.1174	-0.02	0.02	0.04	0.18
2.56	81.27	82.88	88.48	84.74	92.42	2.56	-0.1179	-0.01	0.02	0.04	0.16
3.56	80.65	82.03	89.77	85.13	93.53	3.56	-0.1246	-0.03	0.04	0.04	0.17
4.56	80.94	82.67	88.87	85.26	93.3	4.56	-0.1214	-0.02	0.03	0.04	0.17
5.56	81.89	82.73	88.12	85.79	93.13	5.56	-0.1111	-0.02	0.02	0.05	0.17
6.56	80.93	82.35	87.65	85.13	93.45	6.56	-0.1216	-0.02	0.01	0.04	0.17
7.56	81.68	83.66	87.1	84.57	92.57	7.56	-0.1134	-0.01	0.01	0.04	0.16
8.56	79.24	81.88	88.15	83.56	94.09	8.56	-0.1399	-0.03	0.02	0.02	0.18
9.56	80.84	82.43	88.49	84.27	93.29	9.56	-0.1225	-0.02	0.02	0.03	0.17
10.56	80.84	81.91	88.06	84.83	92.65	10.56	-0.1225	-0.03	0.02	0.04	0.16
11.56	80.59	83.49	88.58	84.73	93.75	11.56	-0.1252	-0.01	0.02	0.04	0.18
12.56	80.94	82.35	85.93	84.51	93.59	12.56	-0.1214	-0.02	-0.01	0.04	0.17
13.56	81.23	82.33	87.21	85.03	91.77	13.56	-0.1183	-0.02	0.01	0.04	0.15
14.56	79.91	82.51	86.83	84.82	92.09	14.56	-0.1326	-0.02	0.00	0.04	0.16
15.56	80.71	82.45	87.32	84.95	92.03	15.56	-0.1239	-0.02	0.01	0.04	0.16
16.58	78.85	82.59	86.15	84.34	91.8	16.58	-0.1441	-0.02	0.00	0.03	0.15
17.58	80.29	82.14	86.22	84.96	93.4	17.58	-0.1285	-0.02	0.00	0.04	0.17
18.58	79.72	82.71	87.01	84.31	94.31	18.58	-0.1347	-0.02	0.01	0.03	0.18
19.58	78.75	81.39	86.55	85.5	92.64	19.58	-0.1452	-0.03	0.00	0.05	0.16
20.58	79.93	82.68	85.66	84.54	93.13	20.58	-0.1324	-0.02	-0.01	0.04	0.17
21.58	80.98	83.13	86.75	86.07	92.69	21.58	-0.1210	-0.01	0.00	0.05	0.16
22.58	79.89	82.89	86.67	84.85	94.23	22.58	-0.1328	-0.01	0.00	0.04	0.18
23.58	81.18	82.76	87.55	85.8	96.14	23.58	-0.1188	-0.02	0.01	0.05	0.21
24.58	81.37	83.52	88.28	86.9	95.19	24.58	-0.1168	-0.01	0.02	0.06	0.19
25.58	84.6	86.93	91.72	89.33	97.8	25.58	-0.0817	0.03	0.06	0.09	0.23
26.58	86.71	89.53	94.82	92.02	101.22	26.58	-0.0588	0.06	0.10	0.13	0.27
27.58	93.29	96.92	103.08	98.52	108.55	27.58	0.0126	0.15	0.19	0.21	0.36
28.56	101.72	105.64	113.34	106.12	119.97	28.56	0.1041	0.26	0.31	0.30	0.51
29.61	116.88	121.05	132.26	121.82	138.04	29.61	0.2687	0.44	0.53	0.49	0.73
30.56	133.98	138.79	153.91	136.51	155.8	30.56	0.4543	0.65	0.78	0.67	0.96
31.63	156.76	162.45	183.47	162.45	184.54	31.63	0.7015	0.93	1.12	0.99	1.32
32.63	182.68	184.57	207.25	183.02	208.79	32.63	0.9829	1.19	1.40	1.24	1.62
33.63	206.59	208.89	240.92	210.87	239.59	33.63	1.2424	1.48	1.79	1.58	2.01
34.63	227.49	232.07	263.86	233.22	265.69	34.63	1.4693	1.76	2.05	1.86	2.33
35.63	244.54	250.69	283.67	249.02	284.54	35.63	1.6543	1.98	2.28	2.05	2.57
36.63	261.52	263.7	305.07	266.42	304.31	36.63	1.8386	2.13	2.53	2.26	2.82
37.65	278.3	283.43	324.68	282.38	323.85	37.65	2.0208	2.37	2.76	2.46	3.06
38.65	292.23	299.4	337.16	296.97	337.77	38.65	2.1720	2.56	2.90	2.64	3.24
39.65	303.96	307.78	351.12	307.2	351.97	39.65	2.2993	2.66	3.06	2.76	3.42
Parameter											
a	269.52	231.55	272.05	236.64	231.435						
b	2.3056	2.2735	2.2417	2.3365	2.2716						
x0	33.307	33.343	33.174	33.35	33.3227						
y0	92.129	84.134	86.448	81.609	79.6712						
R	0.9994	0.9994	0.9994	0.9995	0.999537						
**$CP_{(SDM)}$**	30.27	30.35	30.22	30.27	30.33						

F3			F4			
Well/ Cycle	FAM Normalized Fluorescence	VIC Normalized Fluorescence	Well/ Cycle	FAM Normalized Fluorescence	VIC Normalized Fluorescence	Well/ Cycle
0.56	-0.034064926	-0.032491181	0.56	-0.027445637	0.008120489	0.56
1.56	-0.016008325	-0.017633859	1.56	-0.01084285	0.023042754	1.56
2.56	-0.000992182	-0.014900111	2.56	0.017750839	0.02350546	2.56
3.56	0.002606728	-0.02500309	3.56	0.000225675	0.038427725	3.56
4.56	0.012100405	-0.017396141	4.56	0.006559331	0.028016842	4.56
5.56	0.011728104	-0.01668299	5.56	0.009019003	0.019341107	5.56
6.56	0.020477178	-0.021199616	6.56	0.022424216	0.013904312	6.56
7.56	0.004157982	-0.005629142	7.56	0.0150452	0.007542106	7.56
8.56	0.003971832	-0.026785969	8.56	0.025744773	0.019688136	8.56
9.56	-0.001674734	-0.020248747	9.56	-0.004939637	0.023621136	9.56
10.56	0.009804548	-0.026429393	10.56	0.004530101	0.018647048	10.56
11.56	-0.001116283	-0.007649738	11.56	-0.004632178	0.024662225	11.56
12.56	0.008129193	-0.021199616	12.56	0.021624823	-0.005992041	12.56
13.56	-0.009555106	-0.021437333	13.56	0.014737741	0.008814547	13.56
14.56	0.01414806	-0.019297879	14.56	0.00311579	0.004418841	14.56
15.56	0.000497022	-0.02001103	15.56	-0.007952735	0.010086989	15.56
16.58	-0.00012348	-0.01834701	16.58	0.013815363	-0.003447159	16.58
17.58	0.00347543	-0.023695646	17.58	-0.01939021	-0.002637424	17.58
18.58	0.020911529	-0.016920707	18.58	0.012278068	0.006501018	18.58
19.58	0.012162455	-0.032610039	19.58	-0.012380145	0.0011799	19.58
20.58	-0.004963393	-0.017277283	20.58	-0.009244063	-0.009115306	20.58
21.58	-0.005335695	-0.011928647	21.58	-0.015516227	0.00349343	21.58
22.58	0.001117524	-0.014781253	22.58	-0.000573719	0.002568018	22.58
23.58	-0.000992182	-0.016326414	23.58	-0.020804522	0.012747548	23.58
24.58	-0.001426534	-0.007293162	24.58	0.005821429	0.02119193	24.58
25.58	-0.007879752	0.033237613	25.58	-0.006845882	0.060984638	25.58
26.58	0.005026685	0.064140843	26.58	0.01676697	0.096844346	26.58
27.58	0.007570742	0.151977331	27.58	0.017074429	0.192393115	27.58
28.56	0.039278381	0.255622011	28.56	0.036259872	0.311077179	28.56
29.61	0.026309895	0.438783078	29.61	0.025683282	0.529937072	29.61
30.56	0.095371739	0.649638194	30.56	0.091418019	0.780376643	30.56
31.63	0.090345675	0.930857588	31.63	0.089511774	1.122316306	31.63
32.63	0.136821256	1.193772761	32.63	0.172218249	1.397394966	32.63
33.63	0.166108939	1.482836821	33.63	0.174862397	1.786877661	33.63
34.63	0.196327374	1.758351004	34.63	0.227683856	2.052239497	34.63
35.63	0.258191399	1.979665675	35.63	0.271834971	2.281394596	35.63
36.63	0.29039544	2.134300684	36.63	0.305962922	2.528942254	36.63
37.65	0.338670476	2.368808657	37.65	0.349007184	2.755783824	37.65
38.65	0.367461757	2.558625805	38.65	0.400045381	2.900148066	38.65
39.65	0.408787173	2.658229292	39.65	0.41910784	3.061632426	39.65

periment derived	linear regress		periment derived	linear regress		periment der
**parameter**	**value**		**parameter**	**value**		**parameter**
y0	-0.0036		y0	-0.0119		y0
a	0.1153		a	0.1162		a
**R**	**0.94014058**		**R**	**0.93256072**		**R**

F5		F6	
FAM Normalized Fluorescence	VIC Normalized Fluorescence	FAM Normalized Fluorescence	VIC Normalized Fluorescence
-0.01612574	0.03518248	-0.019786358	0.137550332
-0.025521891	0.040206448	-0.012264163	0.176962315
0.003055369	0.038368411	0.004571226	0.160017673
-0.002063913	0.043147308	-0.004085268	0.173949934
0.009081866	0.044740273	0.017884317	0.171063069
0.008174652	0.051234671	0.017108218	0.168929299
0.005647411	0.043147308	0.011914321	0.172945807
0.008628259	0.036285303	0.019615616	0.161900411
0.009081866	0.023909186	0.018660416	0.180978823
0.009211468	0.032609228	0.007556224	0.170937553
0.003768181	0.039471233	0.007496524	0.162904538
0.006360223	0.038245875	0.003854826	0.176711283
-0.004267149	0.035550088	-0.00092117	0.174703029
-0.011848871	0.04192195	-0.010950764	0.151859141
0.01925563	0.039348698	0.015914218	0.155875649
0.000139322	0.040941663	-0.002771869	0.155122554
0.00312017	0.033466979	0.012630721	0.152235689
0.004221788	0.041064199	0.004392126	0.172318228
0.011479505	0.033099372	0.014362019	0.183740172
-0.003424735	0.047681132	-0.006294167	0.162779022
-0.000508688	0.035917695	-0.007607566	0.168929299
-0.001545505	0.054665673	-0.019428158	0.163406601
0.005453008	0.039716305	0.002362327	0.182736045
-0.001675107	0.051357207	-0.019965458	0.206709576
0.009081866	0.064836145	-0.000682371	0.194785569
-0.006405583	0.094612346	-0.018711758	0.227545211
0.004804997	0.127574477	0.010421822	0.270471638
0.007656243	0.20722275	0.014541119	0.362474771
0.027614965	0.300349962	0.040630002	0.505813895
0.02294929	0.492731176	0.046599998	0.732621073
0.071420471	0.672736274	0.081942374	0.955537258
0.080492618	0.990594152	0.109762555	1.316269869
0.139720772	1.242650302	0.16289552	1.620645854
0.147496898	1.583912519	0.179074209	2.007234735
0.183072671	1.857780043	0.233639972	2.334831156
0.206336246	2.051386615	0.267907749	2.571428571
0.263944375	2.264598916	0.297996529	2.819573447
0.286495138	2.460166061	0.348502695	3.064831457
0.318442053	2.6389458	0.38671067	3.239549549
0.344751277	2.764299928	0.420500847	3.417782084
rived linear regress		Experiment derived linear regression	
**value**	**SD**	**parameter**	**value**
-0.0178	0.0129	y0	-0.0251
0.117	0.0172	a	0.1105
**0.95932913**		**R**	**0.98704815**

AVERAGE	value	SD2
y0	-0.01322	
a	0.12516	0.023414162

Well/ Cycle	Raw fluorescence data F7	F8	F9	G2	G3	Well/ Cycle	F7	F8	F9	G2	G3
0.56	142.62	159.64	147.69	166.96	148.97	0.56	-0.0362	-0.03	-0.04	-0.02	-0.04
1.56	144.97	164.56	151.07	173.07	153.11	1.56	-0.0204	0.00	-0.01	0.01	-0.02
2.56	145.78	167.03	150.74	172.03	153.87	2.56	-0.0149	0.02	-0.02	0.00	-0.01
3.56	144.44	167.70	152.42	175.53	153.62	3.56	-0.0239	0.02	0.00	0.03	-0.01
4.56	149.75	168.07	154.36	179.79	159.65	4.56	0.0119	0.02	0.01	0.05	0.03
5.56	146.52	167.38	154.01	175.41	156.98	5.56	-0.0099	0.02	0.01	0.02	0.01
6.56	150.29	166.79	156.06	175.90	158.46	6.56	0.0156	0.02	0.02	0.03	0.02
7.56	148.44	168.08	153.75	172.46	156.76	7.56	0.0031	0.02	0.00	0.01	0.01
8.56	149.11	166.68	155.67	171.20	155.14	8.56	0.0076	0.02	0.02	0.00	0.00
9.56	147.78	165.47	154.08	171.17	158.92	9.56	-0.0014	0.01	0.01	0.00	0.02
10.56	151.65	166.26	154.94	173.23	157.71	10.56	0.0248	0.01	0.01	0.01	0.01
11.56	148.90	166.33	153.58	173.78	159.04	11.56	0.0062	0.01	0.00	0.01	0.02
12.56	148.98	164.19	154.62	174.84	159.41	12.56	0.0067	0.00	0.01	0.02	0.02
13.56	148.96	165.07	152.35	170.29	156.17	13.56	0.0066	0.01	0.00	-0.01	0.00
14.56	148.27	165.29	154.55	174.47	156.16	14.56	0.0019	0.01	0.01	0.02	0.00
15.56	147.22	163.95	153.78	170.95	155.66	15.56	-0.0052	0.00	0.00	0.00	0.00
16.58	150.27	162.32	152.99	172.85	156.00	16.58	0.0154	-0.01	0.00	0.01	0.00
17.58	148.48	162.55	152.05	169.82	155.73	17.58	0.0034	-0.01	-0.01	-0.01	0.00
18.58	149.83	164.16	154.55	171.35	157.66	18.58	0.0125	0.00	0.01	0.00	0.01
19.58	147.83	162.36	152.83	168.86	155.82	19.58	-0.0010	-0.01	0.00	-0.01	0.00
20.58	149.02	162.11	153.37	168.87	155.34	20.58	0.0070	-0.01	0.00	-0.01	0.00
21.58	149.88	161.94	151.05	168.40	153.26	21.58	0.0128	-0.01	-0.01	-0.02	-0.01
22.58	150.29	163.66	152.79	170.63	154.08	22.58	0.0156	0.00	0.00	0.00	-0.01
23.58	147.17	162.20	153.56	166.02	154.30	23.58	-0.0055	-0.01	0.00	-0.03	-0.01
24.58	147.83	161.12	153.24	164.54	155.08	24.58	-0.0010	-0.02	0.00	-0.04	0.00
25.58	147.02	159.91	152.72	165.30	150.40	25.58	-0.0065	-0.03	0.00	-0.03	-0.03
26.58	149.98	164.02	152.95	166.50	154.06	26.58	0.0135	0.00	0.00	-0.03	-0.01
27.58	150.18	160.52	151.67	168.62	154.75	27.58	0.0148	-0.02	-0.01	-0.02	-0.01
28.56	153.26	163.45	154.60	172.38	154.93	28.56	0.0357	0.00	0.01	0.01	0.00
29.61	149.47	160.65	155.33	169.59	157.83	29.61	0.0100	-0.02	0.01	-0.01	0.01
30.56	157.00	165.69	156.74	177.95	161.63	30.56	0.0609	0.01	0.02	0.04	0.04
31.63	152.23	163.62	156.80	172.76	159.74	31.63	0.0287	0.00	0.02	0.01	0.03
32.63	159.24	169.49	159.63	179.46	164.54	32.63	0.0761	0.03	0.04	0.05	0.06
33.63	158.91	166.52	157.92	178.98	163.57	33.63	0.0738	0.01	0.03	0.05	0.05
34.63	160.58	170.07	161.02	181.54	167.59	34.63	0.0851	0.04	0.05	0.06	0.08
35.63	164.41	170.55	164.10	182.17	167.64	35.63	0.1110	0.04	0.07	0.06	0.08
36.63	168.69	176.51	169.01	189.46	174.41	36.63	0.1399	0.08	0.10	0.11	0.12
37.65	172.25	176.15	168.53	188.51	180.62	37.65	0.1640	0.07	0.10	0.10	0.16
38.65	176.75	179.03	170.38	191.15	180.99	38.65	0.1944	0.09	0.11	0.12	0.16
39.65	176.84	177.28	173.89	193.40	181.07	39.65	0.1950	0.08	0.14	0.13	0.16
Parameter											
a	44.94	14.60	25.03	23.06	31.03						
b	3.34	1.31	2.48	1.90	2.17						
x0	37.26	35.27	36.16	34.97	35.65						
y0	147.98	164.17	153.12	171.21	155.59						
R	0.97	0.87	0.96	0.88	0.95						
**$CP_{(SDM)}$**	32.86	33.55	32.89	32.47	32.79						

Well/ Cycle	F7	F8	F9	G2	G3
0.56	88.53	89.11	83.82	83.51	82.16
1.56	90.27	90.3	84.51	87.54	84.16
2.56	88.2	90.49	85.22	85.52	85.39
3.56	90.84	90.76	84.24	86.37	84.08
4.56	90.58	91.45	84.09	85.26	83.96
5.56	90.22	90.25	84.8	86.73	84.87
6.56	89.3	90.3	84.11	85.95	85
7.56	89.68	90.26	85.84	86.71	85.88
8.56	91.06	89.64	86.25	85.5	85.91
9.56	91.22	90.36	85.67	86.25	86.31
10.56	90.93	89.77	85.25	85.51	85.07
11.56	91.84	89.57	85.84	86.09	85.43
12.56	90.82	89.57	84.49	86.01	85.07
13.56	91.54	89.08	85.84	86.32	85.79
14.56	90.94	88.83	84.97	85.57	86.27
15.56	91.53	88.08	86.15	86.5	85.47
16.58	90.43	90.74	85.51	85.18	86.35
17.58	90.63	89.77	85.54	86.01	85.6
18.58	90.97	88.8	84.97	85.65	85.48
19.58	90.83	89.1	85.78	86.07	84.59
20.58	90.72	88.28	85.07	85.91	85.08
21.58	90.86	89.15	85.1	86.82	84.71
22.58	93.05	88.91	85.66	85.47	85.6
23.58	92.84	89.87	86.62	86.79	86.75
24.58	95.68	89.91	86.8	87.53	87.54
25.58	99.57	92.87	89.61	90.96	91
26.58	104.66	96.64	92.93	96.74	93.42
27.58	116.83	104.67	100.19	106.9	99.98
28.56	133.17	114.6	110.58	120.71	109.09
29.61	156.34	131.51	127.11	142.42	125.87
30.56	181.53	148.58	142.94	163.75	146.1
31.63	215.53	174.99	162.97	191.23	170.62
32.63	238.74	198.67	182.27	214.2	193.05
33.63	269.47	225.15	204.39	242.08	218.69
34.63	290.84	248.86	221.22	261.6	238.3
35.63	311.7	271.71	240.2	277.73	256.82
36.63	324.99	284.41	252.02	293.78	272.14
37.65	342.08	301.9	267.79	310.78	288.29
38.65	355.26	315.12	278.01	323.5	301.93
39.65	369.93	329.21	291.8	333.32	313.7
Parameter					
a	282.47	246.66	214.65	252.97	235.8485
b	2.324	2.259	2.4356	2.3159	2.3095
x0	32.39	33.206	33.141	32.576	33.1058
y0	89.499	88.781	84.253	84.792	84.3201
R	0.9994	0.9994	0.9993	0.9993	0.9993728
$CP_{(SDM)}$	29.33	30.23	29.93	29.53	30.06

Fluorescence Data Normalized To Y0

Well/ Cycle	F7	F8	F9	G2	G3
0.56	-0.0108	0.00	-0.01	-0.02	-0.03
1.56	0.0086	0.02	0.00	0.03	0.00
2.56	-0.0145	0.02	0.01	0.01	0.01
3.56	0.0150	0.02	0.00	0.02	0.00
4.56	0.0121	0.03	0.00	0.01	0.00
5.56	0.0081	0.02	0.01	0.02	0.01
6.56	-0.0022	0.02	0.00	0.01	0.01
7.56	0.0020	0.02	0.02	0.02	0.02
8.56	0.0174	0.01	0.02	0.01	0.02
9.56	0.0192	0.02	0.02	0.02	0.02
10.56	0.0160	0.01	0.01	0.01	0.01
11.56	0.0262	0.01	0.02	0.02	0.01
12.56	0.0148	0.01	0.00	0.01	0.01
13.56	0.0228	0.00	0.02	0.02	0.02
14.56	0.0161	0.00	0.01	0.01	0.02
15.56	0.0227	-0.01	0.02	0.02	0.01
16.58	0.0104	0.02	0.01	0.00	0.02
17.58	0.0126	0.01	0.02	0.01	0.02
18.58	0.0164	0.00	0.01	0.01	0.01
19.58	0.0149	0.00	0.02	0.02	0.00
20.58	0.0136	-0.01	0.01	0.01	0.01
21.58	0.0152	0.00	0.01	0.02	0.00
22.58	0.0397	0.00	0.02	0.01	0.02
23.58	0.0373	0.01	0.03	0.02	0.03
24.58	0.0691	0.01	0.03	0.03	0.04
25.58	0.1125	0.05	0.06	0.07	0.08
26.58	0.1694	0.09	0.10	0.14	0.11
27.58	0.3054	0.18	0.19	0.26	0.19
28.56	0.4879	0.29	0.31	0.42	0.29
29.61	0.7468	0.48	0.51	0.68	0.49
30.56	1.0283	0.67	0.70	0.93	0.73
31.63	1.4082	0.97	0.93	1.26	1.02
32.63	1.6675	1.24	1.16	1.53	1.29
33.63	2.0109	1.54	1.43	1.85	1.59
34.63	2.2496	1.80	1.63	2.09	1.83
35.63	2.4827	2.06	1.85	2.28	2.05
36.63	2.6312	2.20	1.99	2.46	2.23
37.65	2.8222	2.40	2.18	2.67	2.42
38.65	2.9694	2.55	2.30	2.82	2.58
39.65	3.1333	2.71	2.46	2.93	2.72

F7			F8			
Well/ Cycle	FAM Normalized Fluorescence	VIC Normalized Fluorescence	Well/ Cycle	FAM Normalized Fluorescence	VIC Normalized Fluorescence	Well/ Cycle
0.56	-0.036245859	-0.010830252	0.56	-0.027620002	0.003702356	0.56
1.56	-0.020365743	0.008611241	1.56	0.002348111	0.01710608	1.56
2.56	-0.014892171	-0.014517432	2.56	0.017393079	0.01924617	2.56
3.56	-0.023947216	0.014980005	3.56	0.021474102	0.022287351	3.56
4.56	0.01193509	0.012074955	4.56	0.023727802	0.030059258	4.56
5.56	-0.009891623	0.008052577	5.56	0.019524957	0.016542898	5.56
6.56	0.015584138	-0.002226833	6.56	0.01593122	0.01710608	6.56
7.56	0.00308277	0.00201901	7.56	0.023788713	0.016655534	7.56
8.56	0.007610292	0.017438125	8.56	0.015261201	0.009672082	8.56
9.56	-0.001377178	0.019225849	9.56	0.007890994	0.017781898	9.56
10.56	0.024774333	0.0159856	10.56	0.012702947	0.011136354	10.56
11.56	0.006191218	0.026153277	11.56	0.013129323	0.008883628	11.56
12.56	0.006731818	0.01475654	12.56	9.44117E-05	0.008883628	12.56
13.56	0.006596668	0.022801296	13.56	0.005454562	0.003364447	13.56
14.56	0.001933995	0.016097333	14.56	0.0067946	0.000548539	14.56
15.56	-0.005161376	0.022689563	15.56	-0.001367447	-0.007899186	15.56
16.58	0.015448988	0.010398964	16.58	-0.011295908	0.022062078	16.58
17.58	0.00335307	0.012633618	17.58	-0.009894959	0.011136354	17.58
18.58	0.01247569	0.016432531	18.58	-8.83207E-05	0.00021063	18.58
19.58	-0.001039303	0.014868273	19.58	-0.011052265	0.00358972	19.58
20.58	0.007002118	0.013639213	20.58	-0.012575034	-0.005646459	20.58
21.58	0.012813565	0.015203471	21.58	-0.013610518	0.004152902	21.58
22.58	0.015584138	0.039672936	22.58	-0.003133861	0.00144963	22.58
23.58	-0.005499251	0.037326549	23.58	-0.012026837	0.012262717	23.58
24.58	-0.001039303	0.069058641	24.58	-0.018605204	0.012713263	24.58
25.58	-0.006512875	0.112522668	25.58	-0.02597541	0.046053617	25.58
26.58	0.013489314	0.169394621	26.58	-0.000941072	0.088517514	26.58
27.58	0.014840814	0.305373338	27.58	-0.022259852	0.178964489	27.58
28.56	0.035653903	0.487944598	28.56	-0.004412987	0.290812367	28.56
29.61	0.010042991	0.746829305	29.61	-0.021468011	0.481280405	29.61
30.56	0.060926939	1.02828402	30.56	0.009231032	0.673550624	30.56
31.63	0.028693681	1.408175259	31.63	-0.003377504	0.971023177	31.63
32.63	0.076063731	1.667506897	32.63	0.032377135	1.237746012	32.63
33.63	0.073833757	2.010861537	33.63	0.014286628	1.536007019	33.63
34.63	0.085118777	2.249634355	34.63	0.035909962	1.803067763	34.63
35.63	0.110999988	2.482708803	35.63	0.03883368	2.060441782	35.63
36.63	0.139922073	2.631201585	36.63	0.075136516	2.203489924	36.63
37.65	0.16397876	2.822152799	37.65	0.072943728	2.400490869	37.65
38.65	0.194387494	2.969416521	38.65	0.090486038	2.5493961	38.65
39.65	0.194995668	3.133328417	39.65	0.079826648	2.708100692	39.65

periment derived parameter	linear regress value
y0	-0.0093
a	0.0431
R	0.73998972

eriment derived parameter	linear regress value
y0	-0.0268
a	0.0337
R	0.71003981

periment der parameter
y0
a
R

F9			G2		G3	
FAM Normalized Fluorescence	VIC Normalized Fluorescence	Well/ Cycle	FAM Normalized Fluorescence	VIC Normalized Fluorescence	FAM Normalized Fluorescence	VIC Normalized Fluorescence
-0.035430885	-0.005142825	0.56	-0.024850086	-0.015118189	-0.042556337	-0.025617854
-0.013355974	0.003046765	1.56	0.010836102	0.032409935	-0.015948182	-0.001898717
-0.015511217	0.011473735	2.56	0.004761857	0.008586905	-0.011063593	0.012688552
-0.004539072	-0.000157857	3.56	0.025204027	0.018611448	-0.012670366	-0.002847482
0.008131143	-0.001938203	4.56	0.050085069	0.005520574	0.026084989	-0.004270631
0.00584528	0.006488767	5.56	0.024503153	0.022857136	0.008924658	0.006521577
0.019233909	-0.001700824	6.56	0.027365057	0.013658144	0.018436752	0.008063321
0.00414721	0.018832497	7.56	0.007273324	0.022621265	0.007510698	0.018499741
0.016686804	0.023698775	8.56	-8.58571E-05	0.008351034	-0.002901189	0.018855528
0.006302452	0.016814772	9.56	-0.000261076	0.017196218	0.021393213	0.023599355
0.011919146	0.011829804	10.56	0.011770601	0.008468969	0.013616434	0.00889349
0.003036933	0.018832497	11.56	0.014982942	0.015309245	0.022164464	0.013162935
0.009829213	0.002809386	12.56	0.021174	0.014365759	0.024542488	0.00889349
-0.004996245	0.018832497	13.56	-0.005400821	0.018021769	0.003718715	0.01743238
0.009372041	0.008506492	14.56	0.01901297	0.009176584	0.003654444	0.023124973
0.004343141	0.022511878	15.56	-0.001546012	0.020144613	0.000440898	0.013637318
-0.00081638	0.014915736	16.58	0.009551166	0.004577088	0.002626109	0.024073738
-0.006955556	0.015271805	17.58	-0.008145913	0.014365759	0.000890795	0.015179062
0.009372041	0.008506492	18.58	0.000790236	0.01012007	0.013295079	0.013755913
-0.001861346	0.018120358	19.58	-0.013752908	0.015073374	0.001469233	0.003200898
0.001665415	0.009693389	20.58	-0.013694502	0.013186401	-0.001615771	0.009012086
-0.013486595	0.010049458	21.58	-0.016439593	0.023918558	-0.014984119	0.004624046
-0.002122588	0.016696082	22.58	-0.003415011	0.007997226	-0.009713904	0.015179062
0.002906312	0.028090294	23.58	-0.030340269	0.023564751	-0.008299945	0.028817565
0.00081638	0.030226709	24.58	-0.038984386	0.032292	-0.003286814	0.038186625
-0.00257976	0.063578519	25.58	-0.034545515	0.072743977	-0.033365597	0.079220731
-0.001077621	0.102983503	26.58	-0.027536771	0.140910865	-0.009842446	0.107920887
-0.009437351	0.189152235	27.58	-0.015154657	0.260733631	-0.005407754	0.185719656
0.009698593	0.312470847	28.56	0.006806074	0.423602962	-0.004250878	0.293760325
0.014466251	0.508664943	29.61	-0.009489255	0.67964157	0.014387685	0.492763884
0.023675016	0.696550758	30.56	0.039338328	0.931198617	0.038810628	0.732682955
0.024066878	0.934286253	31.63	0.00902551	1.255286177	0.026663427	1.023479574
0.042549718	1.1633574	32.63	0.048157664	1.526184695	0.057513462	1.289489695
0.031381641	1.425899045	33.63	0.045354166	1.854989687	0.051279184	1.59356903
0.051627861	1.625653832	34.63	0.060306154	2.085200355	0.077116087	1.826135168
0.071743461	1.850926907	35.63	0.063985744	2.27543079	0.077437442	2.045774376
0.103810861	1.991218148	36.63	0.106563864	2.464717738	0.120948844	2.227462966
0.100675963	2.178391826	37.65	0.101015275	2.665208587	0.160861076	2.418994996
0.112758384	2.299692712	38.65	0.116434512	2.815222916	0.163239099	2.58075951
0.13568233	2.463365827	39.65	0.129575907	2.931035865	0.163753267	2.720346631

rived linear regress
value
0.0062
0.0223
0.77591708

periment derived linear regress		
parameter	value	SD
y0	-0.022	0.0292
a	0.0388	0.0222
R	0.7108714	

xperiment derived linear regressio	
parameter	value
y0	0.004
a	0.0329
R	0.81417076

AVERAGE	value	SD2
y0	-0.00958	
a	0.03416	0.007808841
