# Supplementary material for: Quantify single nucleotide polymorphism (SNP) ratio in pooled DNA based on normalized fluorescence real-time PCR
Source: BMC Genomics. 2006 Jun 9;7:143. doi: 10.1186/1471-2164-7-143 (PMC1552069; doi:10.1186/1471-2164-7-143)
Supplement: Additional file 3 — Contained the raw and analytical datas used during the procession. contain the ratios of normalized fluorescence for all samples. [file 1471-2164-7-143-S3.pdf]

Well/ Cycle	Raw fluorescence data				Well/ Cycle	C2 Normalized Fluorescence	C3	C4	C5
	C2 sample	C3 sample	C4 sample	C5 sample					
0.56	374.20	410.83	478.36	418.37	0.56	-0.0321	-0.04	-0.02	-0.02
1.56	373.70	420.19	487.86	425.06	1.56	-0.0334	-0.02	0.00	0.00
2.56	373.22	417.79	479.27	414.66	2.56	-0.0346	-0.03	-0.02	-0.03
3.56	381.71	426.28	485.53	424.35	3.56	-0.0126	-0.01	-0.01	0.00
4.56	378.90	423.25	489.46	417.81	4.56	-0.0199	-0.01	0.00	-0.02
5.56	380.32	421.94	487.72	423.27	5.56	-0.0162	-0.02	0.00	-0.01
6.56	388.67	433.44	496.52	426.48	6.56	0.0054	0.01	0.01	0.00
7.56	382.62	426.25	492.81	422.73	7.56	-0.0103	-0.01	0.01	-0.01
8.56	394.06	435.85	498.88	427.40	8.56	0.0193	0.02	0.02	0.00
9.56	390.59	428.75	492.71	427.76	9.56	0.0103	0.00	0.01	0.00
10.56	399.17	435.72	501.27	433.28	10.56	0.0325	0.02	0.02	0.02
11.56	390.59	433.93	491.38	430.09	11.56	0.0103	0.01	0.00	0.01
12.56	401.18	439.89	503.02	438.09	12.56	0.0377	0.03	0.03	0.03
13.56	393.02	443.39	507.21	435.42	13.56	0.0166	0.03	0.03	0.02
14.56	397.51	439.53	503.02	438.54	14.56	0.0282	0.03	0.03	0.03
15.56	401.68	440.47	502.05	440.70	15.56	0.0390	0.03	0.02	0.03
16.58	398.24	442.16	503.37	441.80	16.58	0.0301	0.03	0.03	0.04
17.58	401.74	450.24	504.95	442.29	17.58	0.0392	0.05	0.03	0.04
18.58	403.49	446.00	505.26	446.97	18.58	0.0437	0.04	0.03	0.05
19.58	405.15	445.70	508.58	449.80	19.58	0.0480	0.04	0.04	0.06
20.58	401.65	447.03	507.08	450.39	20.58	0.0389	0.04	0.03	0.06
21.58	399.22	442.83	498.96	437.79	21.58	0.0326	0.03	0.02	0.03
22.58	407.95	446.63	505.30	444.61	22.58	0.0552	0.04	0.03	0.04
23.58	409.78	455.37	522.06	453.85	23.58	0.0600	0.06	0.07	0.06
24.58	413.48	467.48	520.53	463.80	24.58	0.0695	0.09	0.06	0.09
25.58	433.78	483.59	547.63	479.34	25.58	0.1220	0.13	0.12	0.12
26.58	445.53	500.15	577.38	501.53	26.58	0.1524	0.17	0.18	0.18
27.58	482.03	554.79	622.28	551.18	27.58	0.2468	0.29	0.27	0.29
28.56	504.81	580.48	668.01	573.04	28.56	0.3058	0.35	0.36	0.34
29.61	545.66	639.22	739.57	633.48	29.61	0.4114	0.49	0.51	0.49
30.56	589.90	705.26	813.76	691.44	30.56	0.5259	0.65	0.66	0.62
31.63	626.42	753.29	881.08	741.11	31.63	0.6203	0.76	0.80	0.74
32.63	664.30	824.76	956.58	804.91	32.63	0.7183	0.92	0.95	0.89
33.63	695.29	864.82	1008.12	850.14	33.63	0.7985	1.02	1.06	0.99
34.63	735.38	923.73	1089.35	912.73	34.63	0.9022	1.16	1.22	1.14
35.63	761.64	968.13	1131.88	947.18	35.63	0.9701	1.26	1.31	1.22
36.63	808.92	1036.68	1222.01	1020.44	36.63	1.0924	1.42	1.49	1.39
37.65	837.30	1082.54	1291.45	1060.43	37.65	1.1658	1.53	1.64	1.49
38.65	879.41	1132.54	1355.97	1132.85	38.65	1.2747	1.64	1.77	1.66
39.65	899.87	1166.53	1389.23	1165.53	39.65	1.3277	1.72	1.83	1.73
Parameter									
a	576.40	818.60	1004.00	844.80					
b	3.38	3.18	3.13	3.35					
x0	33.07	33.12	33.32	33.58					
y0	386.60	428.60	490.10	426.19					
R	1.00	1.00	1.00	1.00					
**$CP_{(SDM)}$**	28.62	28.93	29.20	29.17					

C7			C8			C9		
Well/ Cycle	AM Normalize Fluorescence	VIC Normalized Fluorescence	Well/ Cycle	FAM Normalized Fluorescence	VIC Normalized Fluorescence	Well/ Cycle	FAM Normalized Fluorescence	VIC Normalized Fluorescence
0.56	-0.0211554	-0.039595434	0.56	-0.0274170	-0.017364091	0.56	0.001971578	-0.009169531
1.56	-0.01426213	-0.016533608	1.56	-0.0180162	-0.035245161	1.56	-0.002441815	-0.019701152
2.56	-0.02943658	-0.027685894	2.56	-0.0193323	-0.013174384	2.56	-0.001548628	-0.019155068
3.56	-0.01652905	-0.003522608	3.56	-0.0070407	-0.024920526	3.56	-0.00315111	-0.017204767
4.56	-0.01250412	-0.013711116	4.56	-0.0063592	-0.018037437	4.56	0.003258818	-0.007063207
5.56	-0.00514821	0.002604265	5.56	-0.0135508	-0.006066845	5.56	0.001157202	-0.003708691
6.56	0.003826922	-0.015982878	6.56	0.0031591	0.010841615	6.56	0.0088281	3.58855E-05
7.56	-0.00313574	-0.005519005	7.56	-0.0001781	-0.000156366	7.56	0.000316556	0.011191602
8.56	0.022424877	0.015546424	8.56	0.0142051	0.023335919	8.56	0.014949056	-0.003786703
9.56	0.005746861	0.003912249	9.56	0.0101157	-0.007263904	9.56	0.010614473	-0.008311399
10.56	0.023535203	-0.00524364	10.56	0.0186705	0.020418088	10.56	0.021700496	0.024141595
11.56	0.01870066	0.013481186	11.56	0.0176599	0.029321215	11.56	0.007094267	0.015950334
12.56	0.02143021	0.019952265	12.56	0.0360854	0.011889042	12.56	0.013609275	0.020475031
13.56	0.02751387	0.010107964	13.56	0.0219842	0.031640517	13.56	0.01423976	0.027184063
14.56	0.038779049	0.028626266	14.56	0.0291758	0.014732057	14.56	0.024327516	0.023829547
15.56	0.028392878	0.014444964	15.56	0.0248280	0.016901727	15.56	0.006463782	0.025857859
16.58	0.020759389	0.034890821	16.58	0.0292934	0.023560368	16.58	0.017418454	0.028510267
17.58	0.030335948	0.049347488	17.58	0.0367435	0.026927097	17.58	0.027427399	0.017120515
18.58	0.038085095	0.043977869	18.58	0.0311970	0.041965152	18.58	0.026324051	0.020943103
19.58	0.038524599	0.041224218	19.58	0.0372840	0.028498237	19.58	0.016735429	0.023049427
20.58	0.033181157	0.060362091	20.58	0.0223603	0.024009265	20.58	0.0222259	0.022269307
21.58	0.024298552	0.046938044	21.58	0.0312675	0.047651183	21.58	0.005150272	0.032566891
22.58	0.032302149	0.070206393	22.58	0.0327952	0.051017912	22.58	0.019414989	0.040056044
23.58	0.070585253	0.073786139	23.58	0.0510562	0.072714608	23.58	0.039958283	0.066502113
24.58	0.063113686	0.101598012	24.58	0.0644053	0.087603031	24.58	0.050834144	0.071962954
25.58	0.128807954	0.112887981	25.58	0.1105162	0.101069946	25.58	0.075974723	0.093728303
26.58	0.166906004	0.135812124	26.58	0.1427140	0.120522157	26.58	0.131509919	0.124543045
27.58	0.267529266	0.183450283	27.58	0.2548890	0.171247538	27.58	0.230128238	0.142719842
28.56	0.339191535	0.263168475	28.56	0.3338087	0.244043696	28.56	0.307021104	0.215271006
29.61	0.477334552	0.334350348	29.61	0.4596384	0.347065597	29.61	0.420613435	0.289226386
30.56	0.63389047	0.442155778	30.56	0.5997573	0.41365201	30.56	0.540799586	0.391032052
31.63	0.750844773	0.534127716	31.63	0.7082190	0.521387332	31.63	0.64808707	0.471462429
32.63	0.872147851	0.640143272	32.63	0.8564226	0.610942317	32.63	0.762309886	0.545963893
33.63	0.986742712	0.733147829	33.63	0.9484564	0.740523968	33.63	0.838703618	0.646677391
34.63	1.13254235	0.838612656	34.63	1.0782579	0.844144398	34.63	0.965325967	0.727185779
35.63	1.213249146	0.988893149	35.63	1.1365428	0.934148281	35.63	1.034442855	0.835232406
36.63	1.362310366	1.110122627	36.63	1.3044411	1.06709666	36.63	1.162904118	0.934931747
37.65	1.476974622	1.199340914	37.65	1.3747120	1.166153303	37.65	1.24998982	1.029014225
38.65	1.598162041	1.328418296	38.65	1.5043020	1.307780361	38.65	1.356121416	1.159840356
39.65	1.666285146	1.450473869	39.65	1.6008717	1.416189028	39.65	1.420194426	1.245497537

nt derived linear re		hent derived linear regr		hent derived linear regr	
aramet	value	paramete	value	paramete	value
y0	-0.0163	y0	-0.0186	y0	-0.0215
a	1.4236	a	1.4288	a	1.4461

AVERAGE

paramet	value
y0	-0.0141
a	1.3951

Well/ Cycle	Raw fluorescence data D2	D3	D4	D5	Well/ Cycle	D2 Normalized Fluorescence	D3 Normalized Fluorescence	D4 Normalized Fluorescence	D5 Normalized Fluorescence
0.56	329.07	377.95	325.12	391.09	0.56	-0.0168810	-0.0170484	-0.0219658	-0.0272758
1.56	331.3	381.23	326.07	396.23	1.56	-0.0102187	-0.0085180	-0.0191079	-0.0144915
2.56	326.4	376.62	324.65	388.15	2.56	-0.0248578	-0.0205074	-0.0233796	-0.0345882
3.56	334.42	382.3	330.17	397.32	3.56	-0.0008975	-0.0057352	-0.0067742	-0.0117804
4.56	329.88	376.87	327.75	396.23	4.56	-0.0144610	-0.0198572	-0.0140541	-0.0144915
5.56	332.01	380.64	325.23	397	5.56	-0.0080975	-0.0100524	-0.0216349	-0.0125763
6.56	337.51	386.83	334.08	403.6	6.56	0.0083341	0.0060462	0.0049879	0.0038393
7.56	336.35	382.11	330.91	400.16	7.56	0.0048685	-0.0062293	-0.0045481	-0.0047168
8.56	337.49	390.17	335.19	406.62	8.56	0.0082744	0.0147327	0.0083271	0.0113506
9.56	337.01	386.08	332.41	403.81	9.56	0.0068403	0.0040957	-0.0000358	0.0043616
10.56	339.83	388.39	337.91	411.47	10.56	0.0152653	0.0101034	0.0165094	0.0234136
11.56	333.97	386.91	334.46	402.46	11.56	-0.0022419	0.0062543	0.0061311	0.0010038
12.56	343.66	393.05	342	412.63	12.56	0.0267077	0.0222228	0.0288131	0.0262988
13.56	341.84	395.27	344.34	409.69	13.56	0.0212703	0.0279965	0.0358523	0.0189864
14.56	343.51	395.45	342.04	413.29	14.56	0.0262595	0.0284646	0.0289334	0.0279404
15.56	344.38	393.13	343.31	416.03	15.56	0.0288587	0.0224309	0.0327539	0.0347553
16.58	342.37	395.76	344.34	417.57	16.58	0.0228537	0.0292709	0.0358523	0.0385856
17.58	345.79	393.44	351.64	416.09	17.58	0.0330712	0.0232371	0.0578124	0.0349046
18.58	343.91	397.35	351.84	415.7	18.58	0.0274546	0.0334061	0.0584140	0.0339345
19.58	347.2	398.65	352.03	417.5	19.58	0.0372837	0.0367870	0.0589856	0.0384115
20.58	343.91	399.76	363.95	415.01	20.58	0.0274546	0.0396738	0.0948436	0.0322184
21.58	341.53	389.63	343.78	414.47	21.58	0.0203441	0.0133283	0.0341677	0.0308753
22.58	344.94	394.77	353.79	421.96	22.58	0.0305318	0.0266961	0.0642801	0.0495045
23.58	353.48	407.85	367.13	424.38	23.58	0.0560456	0.0607139	0.1044098	0.0555236
24.58	355.38	408.63	370.16	426.23	24.58	0.0617220	0.0627425	0.1135247	0.0601249
25.58	367.68	423.38	385.39	442.91	25.58	0.0984691	0.1011034	0.1593400	0.1016116
26.58	380.74	434.44	397.8	456.23	26.58	0.1374867	0.1298677	0.1966721	0.1347413
27.58	409.2	472.96	429.07	494.66	27.58	0.2225129	0.2300484	0.2907393	0.2303249
28.56	429.79	489.31	439.04	509.64	28.56	0.2840269	0.2725706	0.3207313	0.2675834
29.61	465.57	537.53	474.53	553.25	29.61	0.3909221	0.3979785	0.4274932	0.3760507
30.56	504.15	587.39	516.49	602.73	30.56	0.5061825	0.5276516	0.5537183	0.4991180
31.63	535.18	622.55	543.56	644.47	31.63	0.5988867	0.6190938	0.6351510	0.6029343
32.63	576.31	677.25	584.73	697.26	32.63	0.7217654	0.7613546	0.7589996	0.7342343
33.63	595.56	707.53	611.17	733.51	33.63	0.7792761	0.8401052	0.8385371	0.8243958
34.63	639.21	759.2	653.63	784.97	34.63	0.9096834	0.9744857	0.9662664	0.9523878
35.63	669.3	790.6	676.59	816.64	35.63	0.9995794	1.0561490	1.0353352	1.0311578
36.63	712.48	848.33	728.64	869.03	36.63	1.1285825	1.2062901	1.1919133	1.1614629
37.65	742.33	889.76	759.41	922.55	37.65	1.2177615	1.3140389	1.2844764	1.2945786
38.65	779.47	932.7	807.69	967.09	38.65	1.3287197	1.4257149	1.4297136	1.4053590
39.65	797.24	953.7	831.12	996.64	39.65	1.3818088	1.4803306	1.5001963	1.4788562
Parameter					Parameter Value				
a	525.59	646.18	633.02	691.05	a	1.570200	1.680600	1.904300	1.718800
b	3.27	3.19	3.97	3.32	b	3.267400	3.186500	3.969500	3.324800
x0	33.45	33.52	34.55	33.90	x0	33.448100	33.519700	34.545300	33.899100
y0	334.72	384.51	332.42	402.06	y0	0.000000	0.000000	0.000000	0.000000
R	1.00	1.00	1.00	1.00	R	0.998805	0.998843	0.998805	0.998792
**$CP_{(SDM)}$**	29.14	29.32	29.32	29.52					

Well/ Cycle	Raw fluorescence data D2	D3	D4	D5
0.56	108.16	119.08	131.5	175.96
1.56	108.51	118.72	131.4	173.03
2.56	109.24	118.45	130.98	174.09
3.56	110.11	119.08	131.71	175.8
4.56	111.88	120.36	131.56	176.38
5.56	110.34	119.94	134.34	174.6
6.56	111.62	120.83	133.31	178.05
7.56	111.36	120.17	134.07	179.93
8.56	111.06	121.78	134.01	179.8
9.56	111.81	122.12	134.67	177.03
10.56	111.84	120.92	136.08	181.23
11.56	112.55	124.03	135.54	179.91
12.56	113.04	121.97	135.96	181.98
13.56	113.65	124.51	136.84	182.36
14.56	113.64	123.35	136.97	183.07
15.56	113.2	122.64	137.51	184.67
16.58	113.59	123.7	138.4	181.07
17.58	113.76	123.52	138.91	183.66
18.58	113.87	125.53	138.77	187.31
19.58	114.8	125.37	138.97	188.96
20.58	113.75	124.25	140.07	185.32
21.58	115.51	126.18	142.92	185.72
22.58	117.13	126.77	142.83	190.86
23.58	118.3	127.73	142.66	194.6
24.58	120.63	132.65	144.61	195.37
25.58	122.27	133.58	149.44	205.36
26.58	127.48	139.39	158.81	215.2
27.58	133.32	146.58	166.13	229.16
28.56	144.79	158.3	179.69	251.09
29.61	157.19	172.98	197.48	279.48
30.56	170.56	187.05	213.37	312.15
31.63	182.88	205.29	236.89	346.23
32.63	196.53	220.17	252.6	382.72
33.63	212	236.65	275.23	421.43
34.63	225.87	256.41	291.02	449.09
35.63	241.22	273.2	314.94	495.57
36.63	252.01	289.78	330.17	533.2
37.65	265.22	306.62	350.92	557.16
38.65	280.75	324.72	369.72	598.54
39.65	294.5	342.05	389.42	642.62
Parameter				
a	210.56	260.23	295.17	543.15
b	3.30	3.32	3.32	3.28
x0	33.98	34.36	34.05	34.47
y0	110.96	120.70	133.86	177.73
R	1.00	1.00	1.00	1.00
$CP_{(SDM)}$	29.64	29.98	29.68	30.15

Well/ Cycle	D2 Normalized Fluorescence	D3 Normalized Fluorescence	D4 Normalized Fluorescence	D5 Normalized Fluorescence
0.56	-0.0252431	-0.0134054	-0.0176641	-0.0099372
1.56	-0.0220888	-0.0163880	-0.0184111	-0.0264232
2.56	-0.0155100	-0.0186250	-0.0215486	-0.0204590
3.56	-0.0076694	-0.0134054	-0.0160954	-0.0108375
4.56	0.0082822	-0.0028004	-0.0172159	-0.0075740
5.56	-0.0055966	-0.0062801	0.0035513	-0.0175894
6.56	0.0059390	0.0010936	-0.0041430	0.0018225
7.56	0.0035959	-0.0043746	0.0015344	0.0124005
8.56	0.0008922	0.0089645	0.0010862	0.0116691
9.56	0.0076513	0.0117815	0.0060165	-0.0039167
10.56	0.0079217	0.0018393	0.0165496	0.0197152
11.56	0.0143203	0.0276061	0.0125156	0.0122880
12.56	0.0187363	0.0105387	0.0156531	0.0239351
13.56	0.0242337	0.0315830	0.0222269	0.0260733
14.56	0.0241436	0.0219722	0.0231981	0.0300682
15.56	0.0201783	0.0160897	0.0272320	0.0390708
16.58	0.0236930	0.0248720	0.0338805	0.0188149
17.58	0.0252251	0.0233807	0.0376903	0.0333879
18.58	0.0262164	0.0400338	0.0366445	0.0539251
19.58	0.0345977	0.0387082	0.0381385	0.0632091
20.58	0.0251350	0.0294288	0.0463558	0.0427281
21.58	0.0409964	0.0454191	0.0676460	0.0449788
22.58	0.0555961	0.0503074	0.0669736	0.0738997
23.58	0.0661404	0.0582611	0.0657037	0.0949433
24.58	0.0871387	0.0990240	0.0802707	0.0992758
25.58	0.1019187	0.1067292	0.1163519	0.1554859
26.58	0.1488721	0.1548659	0.1863480	0.2108520
27.58	0.2015032	0.2144360	0.2410301	0.2893998
28.56	0.3048729	0.3115379	0.3423265	0.4127919
29.61	0.4166239	0.4331638	0.4752220	0.5725321
30.56	0.5371166	0.5497357	0.5939240	0.7563543
31.63	0.6481466	0.7008567	0.7696239	0.9481100
32.63	0.7711628	0.8241396	0.8869813	1.1534260
33.63	0.9105812	0.9606787	1.0560327	1.3712330
34.63	1.0355801	1.1243931	1.1739877	1.5268658
35.63	1.1739170	1.2635006	1.3526758	1.7883918
36.63	1.2711583	1.4008683	1.4664474	2.0001221
37.65	1.3902092	1.5403901	1.6214548	2.1349363
38.65	1.5301683	1.6903511	1.7618952	2.3677665
39.65	1.6540857	1.8339326	1.9090589	2.6157886

D2			D3			D4	
Well/ Cycle	FAM Normalized Fluorescence	VIC Normalized Fluorescence	Well/ Cycle	FAM Normalize Fluorescence	VIC Normalized Fluorescence	Well/ Cycle	FAM Normalized Fluorescence
0.56	-0.0168810	-0.025243103	0.56	-0.017048404	-0.013405359	0.56	-0.0219658
1.56	-0.0102187	-0.022088842	1.56	-0.00851796	-0.01638801	1.56	-0.0191079
2.56	-0.0248578	-0.015509954	2.56	-0.020507395	-0.018624998	2.56	-0.0233796
3.56	-0.0008975	-0.007669361	3.56	-0.005735163	-0.013405359	3.56	-0.0067742
4.56	-0.0144610	0.008282189	4.56	-0.019857209	-0.002800378	4.56	-0.0140541
5.56	-0.0080975	-0.005596561	5.56	-0.0100524	-0.006280137	5.56	-0.0216349
6.56	0.0083341	0.005939024	6.56	0.006046212	0.001093639	6.56	0.0049879
7.56	0.0048685	0.003595858	7.56	-0.006229305	-0.004374555	7.56	-0.0045481
8.56	0.0082744	0.000892205	8.56	0.014732701	0.008964523	8.56	0.0083271
9.56	0.0068403	0.007651337	9.56	0.004095653	0.011781471	9.56	-0.0000358
10.56	0.0152653	0.007921702	10.6	0.010103374	0.001839301	10.56	0.0165094
11.56	-0.0022419	0.014320347	11.6	0.006254272	0.027606091	11.56	0.0061311
12.56	0.0267077	0.018736313	12.6	0.022222846	0.0105387	12.56	0.0288131
13.56	0.0212703	0.02423374	13.6	0.0279965	0.031582959	13.56	0.0358523
14.56	0.0262595	0.024143618	14.6	0.028464635	0.021972195	14.56	0.0289334
15.56	0.0288587	0.020178261	15.6	0.022430906	0.016089745	15.56	0.0327539
16.58	0.0228537	0.023693009	16.6	0.029270866	0.024871995	16.58	0.0358523
17.58	0.0330712	0.025225079	17.6	0.023237137	0.023380669	17.58	0.0578124
18.58	0.0274546	0.026216418	18.6	0.03340605	0.040033803	18.58	0.0584140
19.58	0.0372837	0.034597742	19.6	0.036787019	0.038708181	19.58	0.0589856
20.58	0.0274546	0.025134957	20.6	0.039673846	0.029428822	20.58	0.0948436
21.58	0.0203441	0.040996386	21.6	0.013328298	0.045419145	21.58	0.0341677
22.58	0.0305318	0.05559611	22.6	0.026696128	0.050307379	22.58	0.0642801
23.58	0.0560456	0.066140356	23.6	0.060713873	0.058261115	23.58	0.1044098
24.58	0.0617220	0.087138724	24.6	0.062742454	0.09902401	24.58	0.1135247
25.58	0.0984691	0.101918692	25.6	0.101103444	0.106729192	25.58	0.1593400
26.58	0.1374867	0.148872126	26.6	0.129867684	0.154865864	26.58	0.1966721
27.58	0.2225129	0.201503231	27.6	0.230048384	0.21443603	27.58	0.2907393
28.56	0.2840269	0.304872883	28.6	0.272570566	0.311537888	28.56	0.3207313
29.61	0.3909221	0.416623859	29.6	0.397978493	0.433163764	29.61	0.4274932
30.56	0.5061825	0.537116645	30.6	0.527651642	0.549735704	30.56	0.5537183
31.63	0.5988867	0.648146646	31.6	0.619093838	0.700856684	31.63	0.6351510
32.63	0.7217654	0.771162841	32.6	0.761354593	0.824139588	32.63	0.7589996
33.63	0.7792761	0.910581195	33.6	0.840105153	0.960678719	33.63	0.8385371
34.63	0.9096834	1.035580069	34.6	0.974485651	1.124393113	34.63	0.9662664
35.63	0.9995794	1.173916962	35.6	1.056149046	1.263500638	35.63	1.0353352
36.63	1.1285825	1.271158335	36.6	1.206290058	1.400868283	36.63	1.1919133
37.65	1.2177615	1.390209173	37.7	1.314038926	1.540390064	37.65	1.2844764
38.65	1.3287197	1.530168257	38.7	1.425714919	1.690351124	38.65	1.4297136
39.65	1.3818088	1.65408567	39.7	1.480330565	1.833932625	39.65	1.5001963

eriment derived linear regres	
aramet	value
y0	0.0115
a	0.9163
R	0.9986361

nt derived linear re	
paramet	value
y0	0.006
a	0.9079
R	0.99685065

eriment derived linear regress	
parametei	value
y0	0.0735
a	0.7627
R	0.99502288

D5

VIC Normalized Fluorescence	Well/ Cycle	FAM Normalized Fluorescence	VIC Normalized Fluorescence
-0.017664117	0.56	-0.027275775	-0.009937201
-0.018411141	1.56	-0.014491499	-0.026423243
-0.021548639	2.56	-0.034588182	-0.02045901
-0.016095368	3.56	-0.011780437	-0.010837463
-0.017215903	4.56	-0.014491499	-0.007574014
0.00355135	5.56	-0.012576345	-0.017589426
-0.004142992	6.56	0.003839262	0.001822467
0.001534386	7.56	-0.004716751	0.012400542
0.001086172	8.56	0.011350646	0.01166908
0.006016527	9.56	0.004361577	-0.003916701
0.016549558	10.56	0.023413631	0.019715168
0.012515631	11.56	0.001003839	0.012288009
0.01565313	12.56	0.026298798	0.023935145
0.022226937	13.56	0.018986391	0.026073267
0.023198067	14.56	0.027940359	0.030068178
0.027231994	15.56	0.034755323	0.039070795
0.033880503	16.58	0.038585631	0.018814907
0.037690323	17.58	0.034904556	0.033387893
0.03664449	18.58	0.033934543	0.053925113
0.038138537	19.58	0.038411526	0.063209062
0.046355795	20.58	0.032218365	0.042728108
0.067645965	21.58	0.03087527	0.044978762
0.066973644	22.58	0.049504497	0.073899669
0.065703704	23.58	0.055523553	0.094943286
0.080270662	24.58	0.060124898	0.099275796
0.116351896	25.58	0.101611615	0.155485885
0.186347996	26.58	0.134741295	0.21085198
0.241030116	27.58	0.2303249	0.289399812
0.3423265	28.56	0.267583354	0.412791931
0.475221978	29.61	0.376050723	0.572532115
0.59392401	30.56	0.499118034	0.7563543
0.769623933	31.63	0.602934315	0.948110041
0.886981323	32.63	0.734234301	1.153425974
1.056032738	33.63	0.824395781	1.371233038
1.173987746	34.63	0.952387774	1.526865778
1.352675763	35.63	1.031157818	1.788391801
1.46644744	36.63	1.161462919	2.000122098
1.621454813	37.65	1.294578572	2.134936287
1.761895228	38.65	1.405359049	2.367766468
1.909058855	39.65	1.4788562	2.615788565

ent derived	linear regr
paramete	value
y0	0.0172
a	0.6244
**R**	**0.99776804**

AVERAGE

ent derived	linear regr
paramete	value
y0	0.02705
a	0.802825

Well/ Cycle	Raw fluorescence data)6 sampl	)7 sampl(	D8 sample	D9 sample	Well/ Cycle	D6 Normalized Fluorescence	D7 Normalized Fluorescence	D8 Normalized Fluorescence	D9 Normalized Fluorescence
0.56	379.47	458.66	445.11	412.24	0.56	-0.0199395	-0.0080848	-0.0090932	-0.0226210
1.56	380.66	459.63	442.73	407.55	1.56	-0.0168661	-0.0059870	-0.0143915	-0.0337405
2.56	373.61	454.97	442.68	410.22	2.56	-0.0350742	-0.0160649	-0.0145028	-0.0274102
3.56	382.89	458.37	448.58	415.22	3.56	-0.0111067	-0.0087120	-0.0013682	-0.0155557
4.56	381.01	454.56	445.07	416.53	4.56	-0.0159622	-0.0169516	-0.0091822	-0.0124498
5.56	382.48	458	448.13	415.7	5.56	-0.0121656	-0.0095121	-0.0023700	-0.0144177
6.56	388.31	467.73	451.87	424.26	6.56	0.0028916	0.0115303	0.0059560	0.0058772
7.56	382.58	455.97	447.53	424.46	7.56	-0.0119073	-0.0139023	-0.0037057	0.0063514
8.56	390.55	467.24	451.76	423.9	8.56	0.0086769	0.0104706	0.0057111	0.0050237
9.56	390.1	468.88	453.8	423.81	9.56	0.0075146	0.0140173	0.0102526	0.0048103
10.56	392.82	465.61	456.63	431.38	10.56	0.0145396	0.0069455	0.0165527	0.0227580
11.56	389.34	465.63	453.62	430.1	11.56	0.0055518	0.0069888	0.0098519	0.0197233
12.56	397.53	468.69	459.51	435.81	12.56	0.0267042	0.0136064	0.0229642	0.0332611
13.56	397.85	472.86	460.97	433.39	13.56	0.0275306	0.0226246	0.0262145	0.0275235
14.56	399.33	474.77	458.08	441.8	14.56	0.0313531	0.0267553	0.0197807	0.0474628
15.56	397.45	476.16	456.97	433.39	15.56	0.0264976	0.0297613	0.0173096	0.0275235
16.58	401.23	470.11	460.58	430.95	16.58	0.0362602	0.0166774	0.0253463	0.0217385
17.58	400.12	480.44	463.96	434.13	17.58	0.0333934	0.0390174	0.0328708	0.0292780
18.58	402.09	478.18	460.1	435.66	18.58	0.0384813	0.0341299	0.0242777	0.0329055
19.58	402.95	476.04	457.92	435.01	19.58	0.0407025	0.0295018	0.0194245	0.0313644
20.58	405.24	479.01	459.57	434.93	20.58	0.0466169	0.0359249	0.0230978	0.0311747
21.58	397.12	475.62	462.51	431.4	21.58	0.0256453	0.0285935	0.0296428	0.0228054
22.58	403.36	480.35	462.38	439.18	22.58	0.0417614	0.0388228	0.0293534	0.0412510
23.58	410.26	487.69	474.23	445.61	23.58	0.0595821	0.0546966	0.0557340	0.0564959
24.58	415.94	489.68	472.41	450.07	24.58	0.0742518	0.0590002	0.0516823	0.0670701
25.58	427.75	513.2	491.99	466.56	25.58	0.1047536	0.1098654	0.0952714	0.1061662
26.58	441.7	526.36	502.56	479.65	26.58	0.1407824	0.1383257	0.1188024	0.1372013
27.58	472.34	565.4	541.01	517.57	27.58	0.2199166	0.2227551	0.2044001	0.2271057
28.56	488.57	586.66	568.58	555.61	28.56	0.2618340	0.2687328	0.2657766	0.3172947
29.61	537.75	643.48	626.24	589.72	29.61	0.3888516	0.3916138	0.3941396	0.3981660
30.56	586.92	696.21	674.22	636.29	30.56	0.5158434	0.5056497	0.5009530	0.5085787
31.63	625.62	751.74	715.19	679.56	31.63	0.6157942	0.6257409	0.5921607	0.6111675
32.63	670.61	810.42	774.39	726.42	32.63	0.7319903	0.7526445	0.7239522	0.7222678
33.63	696.9	849.15	802.19	759.05	33.63	0.7998897	0.8364034	0.7858407	0.7996302
34.63	754.67	905.14	857.31	806.17	34.63	0.9490927	0.9574895	0.9085492	0.9113469
35.63	785.2	951.23	892.46	834.09	35.63	1.0279428	1.0571654	0.9868004	0.9775424
36.63	836.57	1008.95	946.31	894.71	36.63	1.1606166	1.1819928	1.1066816	1.1212662
37.65	875.97	1059.78	1002.43	924.16	37.65	1.2623753	1.2919197	1.2316163	1.1910892
38.65	926.32	1126.5	1042.08	970.28	38.65	1.3924147	1.4362109	1.3198854	1.3004350
39.65	949.57	1157.13	1077.13	992.52	39.65	1.4524627	1.5024524	1.3979140	1.3531638
Parameter					Parameter Value				
a	651.86	800.363	704.7422	639.4115	a	1.683600	1.730900	1.568900	1.516000
b	3.341	3.2985	3.2178	3.273	b	3.341000	3.298500	3.217800	3.273000
x0	33.7798	33.8228	33.4847	33.1656	x0	33.779800	33.822800	33.484700	33.165600
y0	387.19	462.398	449.1946	421.7811	y0	0.000000	0.000000	0.000000	0.000000
R	0.99867	0.99888	0.998606	0.998496	R	0.998672	0.998884	0.998606	0.998496
**$CP_{(SDM)}$**	29.38	29.48	29.25	28.86					

Well/ Cycle	Raw fluorescence data D6	D7	D8	D9
0.56	146.17	157.28	176.61	157.66
1.56	145.6	156.77	175.77	159.17
2.56	143.7	154.58	177.86	161.04
3.56	146.21	155.46	174.39	162.17
4.56	146.74	159.22	178.76	162.39
5.56	146.74	158	178.69	163.4
6.56	149.11	158.48	176.46	162.52
7.56	147.85	157.83	174.38	165.12
8.56	148.06	159.64	174.37	165.84
9.56	149.16	158.19	172.5	163.72
10.56	152.69	161.82	173.41	166.09
11.56	151.7	162.37	174.78	168.28
12.56	151.77	162.05	174.05	163.33
13.56	150.2	161.14	173.03	163.2
14.56	153.15	165.25	173.9	164.09
15.56	155.09	163.8	173.69	166.64
16.58	150.1	166.07	175.04	161.01
17.58	154.25	166.13	177.32	164.94
18.58	157.03	166.11	172.83	166.63
19.58	155.71	167.18	176.88	165.91
20.58	160.7	166.3	178.97	165.03
21.58	159.41	170.28	177.04	168.6
22.58	162.83	170.91	177.75	169.31
23.58	162.7	172.64	183.11	171.32
24.58	170.29	177.04	185.26	176.96
25.58	174.25	186.61	198.78	182.16
26.58	181.64	194.9	211.26	198.04
27.58	195.23	212.82	228.23	212.78
28.56	219.16	238.59	259.45	240
29.61	244.88	260.66	295.52	274.34
30.56	273.79	298.15	337.96	307.04
31.63	303.25	328.11	371.46	341.67
32.63	337.1	365.31	407.29	368.5
33.63	363.44	400.52	452.99	409.54
34.63	397.47	430.02	495.68	440.65
35.63	431.62	465.67	545.4	477.62
36.63	458.89	497.87	580.14	505.79
37.65	477.23	524.26	614.39	535.84
38.65	508.76	552.57	649.12	566.82
39.65	537.9	587.81	686.66	603.48
Parameter				
a	435.78	477.08	566.27	483.02
b	3.15	3.13	2.96	3.05
x0	33.68	33.65	33.65	33.52
y0	148.76	158.97	172.58	161.12
R	1.00	1.00	1.00	1.00
$CP_{(SDM)}$	29.53	29.53	29.76	29.50

Well/ Cycle	D6 Normalized Fluorescence	D7 Normalized Fluorescence	D8 Normalized Fluorescence	D9 Normalized Fluorescence
0.56	-0.0174218	-0.0106154	0.0233230	-0.0214941
1.56	-0.0212535	-0.0138236	0.0184559	-0.0121224
2.56	-0.0340256	-0.0276000	0.0305658	-0.0005164
3.56	-0.0171529	-0.0220643	0.0104598	0.0064969
4.56	-0.0135902	0.0015884	0.0357807	0.0078623
5.56	-0.0135902	-0.0060861	0.0353751	0.0141308
6.56	0.0023413	-0.0030667	0.0224539	0.0086691
7.56	-0.0061286	-0.0071556	0.0104018	0.0248059
8.56	-0.0047169	0.0042304	0.0103439	0.0292745
9.56	0.0026774	-0.0048909	-0.0004914	0.0161169
10.56	0.0264067	0.0179439	0.0047814	0.0308261
11.56	0.0197517	0.0214037	0.0127195	0.0444182
12.56	0.0202223	0.0193908	0.0084897	0.0136964
13.56	0.0096685	0.0136663	0.0025796	0.0128895
14.56	0.0294989	0.0395207	0.0076206	0.0184132
15.56	0.0425398	0.0303993	0.0064038	0.0342396
16.58	0.0089963	0.0446789	0.0142261	-0.0007026
17.58	0.0368932	0.0450564	0.0274369	0.0236887
18.58	0.0555808	0.0449306	0.0014208	0.0341776
19.58	0.0467076	0.0516615	0.0248875	0.0297089
20.58	0.0802512	0.0461258	0.0369975	0.0242473
21.58	0.0715796	0.0711623	0.0258146	0.0464042
22.58	0.0945694	0.0751254	0.0299285	0.0508108
23.58	0.0936955	0.0860081	0.0609857	0.0632857
24.58	0.1447167	0.1136868	0.0734433	0.0982900
25.58	0.1713364	0.1738877	0.1517816	0.1305634
26.58	0.2210132	0.2260368	0.2240939	0.2291216
27.58	0.3123674	0.3387642	0.3224224	0.3206044
28.56	0.4732287	0.5008728	0.5033189	0.4895434
29.61	0.6461226	0.6397062	0.7123177	0.7026722
30.56	0.8404603	0.8755406	0.9582258	0.9056225
31.63	1.0384951	1.0640068	1.1523332	1.1205512
32.63	1.2660403	1.2980169	1.3599413	1.2870698
33.63	1.4431020	1.5195087	1.6247387	1.5417817
34.63	1.6718571	1.7050812	1.8720953	1.7348638
35.63	1.9014188	1.9293409	2.1601856	1.9643155
36.63	2.0847322	2.1318980	2.3614780	2.1391507
37.65	2.2080166	2.2979068	2.5599311	2.3256539
38.65	2.4199663	2.4759935	2.7611655	2.5179291
39.65	2.6158500	2.6976741	2.9786818	2.7454569
Parameter Value				
a	2.929400	3.001100	3.281100	2.997800
b	3.153000	3.128800	2.959300	3.052000
x0	33.681000	33.654700	33.653000	33.524400
y0	0.000000	0.000000	0.000000	0.000000
R	0.999323	0.999327	0.999203	0.998905

D6			D7			D8	
Well/ Cycle	FAM Normalized Fluorescence	VIC Normalized Fluorescence	Well/ Cycle	FAM Normalized Fluorescence	VIC Normalized Fluorescence	Well/ Cycle	FAM Normalized Fluorescence
0.56	-0.0199395	-0.017421823	0.56	-0.008084803	-0.010615377	0.56	-0.0090932
1.56	-0.0168661	-0.021253454	1.56	-0.005987045	-0.01382358	1.56	-0.0143915
2.56	-0.0350742	-0.034025559	2.56	-0.016064934	-0.027599981	2.56	-0.0145028
3.56	-0.0111067	-0.017152937	3.56	-0.008711968	-0.022064258	3.56	-0.0013682
4.56	-0.0159622	-0.013590192	4.56	-0.016951616	0.001588375	4.56	-0.0091822
5.56	-0.0121656	-0.013590192	5.56	-0.009512144	-0.00608615	5.56	-0.0023700
6.56	0.0028916	0.002341328	6.56	0.011530317	-0.003066665	6.56	0.0059560
7.56	-0.0119073	-0.006128594	7.56	-0.013902297	-0.007155551	7.56	-0.0037057
8.56	0.0086769	-0.00471694	8.56	0.010470624	0.004230424	8.56	0.0057111
9.56	0.0075146	0.002677436	9.56	0.01401735	-0.004890937	9.56	0.0102526
10.56	0.0145396	0.026406662	10.56	0.006945526	0.017943919	10.56	0.0165527
11.56	0.0055518	0.019751724	11.56	0.006988779	0.021403746	11.56	0.0098519
12.56	0.0267042	0.020222275	12.56	0.013606448	0.019390756	12.56	0.0229642
13.56	0.0275306	0.009668483	13.56	0.022624646	0.013666315	13.56	0.0262145
14.56	0.0313531	0.029498856	14.56	0.026755283	0.039520657	14.56	0.0197807
15.56	0.0264976	0.042539847	15.56	0.029761349	0.030399295	15.56	0.0173096
16.58	0.0362602	0.008996267	16.58	0.016677393	0.044678944	16.58	0.0253463
17.58	0.0333934	0.036893233	17.58	0.039017436	0.045056379	17.58	0.0328708
18.58	0.0384813	0.055580838	18.58	0.034129876	0.044930568	18.58	0.0242777
19.58	0.0407025	0.046707587	19.58	0.029501832	0.051661503	19.58	0.0194245
20.58	0.0466169	0.080251167	20.58	0.035924865	0.04612578	20.58	0.0230978
21.58	0.0256453	0.07157958	21.58	0.028593525	0.071162345	21.58	0.0296428
22.58	0.0417614	0.094569368	22.58	0.038822799	0.075125419	22.58	0.0293534
23.58	0.0595821	0.093695487	23.58	0.054696556	0.086008146	23.58	0.0557340
24.58	0.0742518	0.144716684	24.58	0.059000204	0.11368676	24.58	0.0516823
25.58	0.1047536	0.171336439	25.58	0.109865432	0.173887744	25.58	0.0952714
26.58	0.1407824	0.221013204	26.58	0.138325738	0.226036769	26.58	0.1188024
27.58	0.2199166	0.312367363	27.58	0.222755096	0.338764213	27.58	0.2044001
28.56	0.2618340	0.47322866	28.56	0.268732764	0.50087282	28.56	0.2657766
29.61	0.3888516	0.646122624	29.61	0.391613812	0.639706229	29.61	0.3941396
30.56	0.5158434	0.84046028	30.56	0.505649674	0.875540598	30.56	0.5009530
31.63	0.6157942	1.038495123	31.63	0.62574092	1.064006794	31.63	0.5921607
32.63	0.7319903	1.266040251	32.63	0.752644473	1.29801689	32.63	0.7239522
33.63	0.7998897	1.443101954	33.63	0.836403413	1.519508705	33.63	0.7858407
34.63	0.9490927	1.671857071	34.63	0.957489472	1.705081227	34.63	0.9085492
35.63	1.0279428	1.901418846	35.63	1.057165423	1.929340903	35.63	0.9868004
36.63	1.1606166	2.084732159	36.63	1.181992844	2.131898029	36.63	1.1066816
37.65	1.2623753	2.20801658	37.65	1.291919695	2.297906805	37.65	1.2316163
38.65	1.3924147	2.419966295	38.65	1.436210852	2.475993521	38.65	1.3198854
39.65	1.4524627	2.615850047	39.65	1.502452431	2.697674053	39.65	1.3979140

ment derived	linear regr		nent derived	linear regi		iment derived	linear regre
paramete	value		paramete	value		parameter	value
y0	0.016		y0	0.0109		y0	0.0183
a	0.5739		a	0.5722		a	0.5106

D9

VIC Normalized Fluorescence	Well/ Cycle	**FAM** Normalized Fluorescence	VIC Normalized Fluorescence
0.023323027	0.56	-0.022620976	-0.021494111
0.018455855	1.56	-0.033740488	-0.012122401
0.030565844	2.56	-0.02741019	-0.000516375
0.010459786	3.56	-0.015555699	0.006496892
0.035780671	4.56	-0.012449823	0.007862307
0.035375074	5.56	-0.014417668	0.014130802
0.022453889	6.56	0.005877219	0.008669143
0.010401843	7.56	0.006351399	0.024805863
0.010343901	8.56	0.005023696	0.029274493
-0.000491353	9.56	0.004810315	0.01611686
0.004781418	10.56	0.022758014	0.030826101
0.012719544	11.56	0.019723264	0.044418184
0.00848974	12.56	0.033261092	0.013696352
0.002579601	13.56	0.027523519	0.012889516
0.007620602	14.56	0.047462772	0.018413239
0.006403808	15.56	0.027523519	0.034239638
0.01422605	16.58	0.021738527	-0.000702568
0.027436947	17.58	0.029277983	0.023688705
0.001420751	18.58	0.032905457	0.034177573
0.024887476	19.58	0.031364374	0.029708943
0.036997464	20.58	0.031174702	0.024247284
0.025814556	21.58	0.022805432	0.046404242
0.029928476	22.58	0.041251019	0.050810808
0.060985672	23.58	0.056495893	0.063285734
0.073443316	24.58	0.067070099	0.098290004
0.151781617	25.58	0.106166208	0.130563445
0.224093895	26.58	0.137201264	0.229121567
0.322422369	27.58	0.227105719	0.320604357
0.503318948	28.56	0.317294682	0.489543405
0.712317655	29.61	0.398166015	0.702672241
0.958225753	30.56	0.508578739	0.90562253
1.15233323	31.63	0.611167499	1.12055123
1.359941316	32.63	0.722267783	1.28706977
1.624738679	33.63	0.799630187	1.541781693
1.872095341	34.63	0.911346905	1.734863756
2.160185602	35.63	0.977542379	1.964315505
2.361477952	36.63	1.121266221	2.139150662
2.559931118	37.65	1.191089169	2.32565391
2.761165526	38.65	1.300434989	2.517929137
2.978681784	39.65	1.353163762	2.745456893

**eriment derived paramete**	linear regress **value**
y0	0.0406
a	0.5213

AVERAGE

**eriment derived paramete**	linear regress **value**
y0	0.02145
a	0.5445
**R**	

Well/ Cycle	Raw fluorescence data E2	E3	E4	E5	Well/ Cycle	E2 Normalized Fluorescence	E3 Normalized Fluorescence	E4 Normalized Fluorescence	E5 Normalized Fluorescence
0.56	343.45	356.36	348.07	386.88	0.56	-0.0300100	-0.0119037	-0.0306628	-0.0133801
1.56	345.55	361.16	356.94	389.4	1.56	-0.0240790	0.0014055	-0.0059608	-0.0069536
2.56	347.17	354.32	350.74	382.91	2.56	-0.0195037	-0.0175601	-0.0232271	-0.0235044
3.56	352.38	359.62	353.46	389.01	3.56	-0.0047894	-0.0028645	-0.0156522	-0.0079482
4.56	347.77	355.21	354.14	389.13	4.56	-0.0178092	-0.0150923	-0.0137585	-0.0076422
5.56	350.98	353.04	356.22	387.79	5.56	-0.0087433	-0.0211092	-0.0079659	-0.0110594
6.56	356.35	364.54	361.57	394	6.56	0.0064229	0.0107774	0.0069333	0.0047773
7.56	352.81	360.94	358.72	392.81	7.56	-0.0035749	0.0007955	-0.0010037	0.0017425
8.56	357.54	365.53	362.69	400.92	8.56	0.0097838	0.0135224	0.0100523	0.0224246
9.56	357.11	360.41	357.72	391.22	9.56	0.0085694	-0.0006741	-0.0037886	-0.0023123
10.56	358.51	366.76	368.4	396.27	10.56	0.0125233	0.0169329	0.0259541	0.0105662
11.56	356.02	360.1	361.43	394.59	11.56	0.0054909	-0.0015336	0.0065434	0.0062819
12.56	359.6	366.65	365.99	400.8	12.56	0.0156017	0.0166279	0.0192425	0.0221186
13.56	361.95	364.73	367.9	400.37	13.56	0.0222387	0.0113042	0.0245616	0.0210220
14.56	364.2	369.12	366.59	400.19	14.56	0.0285933	0.0234766	0.0209134	0.0205630
15.56	363.07	368.96	369.68	398.82	15.56	0.0254019	0.0230329	0.0295187	0.0170692
16.58	365.26	365.88	362.73	399.97	16.58	0.0315870	0.0144929	0.0101637	0.0200020
17.58	367.93	369.69	368.01	399.22	17.58	0.0391278	0.0250570	0.0248680	0.0180893
18.58	362.04	369.13	369	400.44	18.58	0.0224929	0.0235043	0.0276250	0.0212005
19.58	367.77	371.52	369.62	401.39	19.58	0.0386759	0.0301312	0.0293516	0.0236232
20.58	366.52	373.91	371.09	402.05	20.58	0.0351456	0.0367580	0.0334454	0.0253064
21.58	363.33	363.29	365.72	397.32	21.58	0.0261362	0.0073115	0.0184906	0.0132439
22.58	367.01	370.95	369.68	400.72	22.58	0.0365295	0.0285507	0.0295187	0.0219146
23.58	376.16	377.6	376.99	405.54	23.58	0.0623714	0.0469895	0.0498763	0.0342065
24.58	375.56	377.27	374.29	410.37	24.58	0.0606768	0.0460745	0.0423571	0.0465240
25.58	386.53	391.76	387.83	419.52	25.58	0.0916589	0.0862516	0.0800645	0.0698583
26.58	395.19	397.78	393.47	429.18	26.58	0.1161169	0.1029435	0.0957713	0.0944932
27.58	419.1	427.04	415.87	458.26	27.58	0.1836449	0.1840741	0.1581529	0.1686529
28.56	428.3	433.39	427.67	463.18	28.56	0.2096280	0.2016811	0.1910146	0.1811999
29.61	450.05	466.42	466.28	504.75	29.61	0.2710555	0.2932649	0.2985393	0.2872115
30.56	478.38	497.73	493.74	542.93	30.56	0.3510666	0.3800796	0.3750124	0.3845780
31.63	498.26	528.94	520.32	571.75	31.63	0.4072128	0.4666171	0.4490348	0.4580746
32.63	535.07	563.75	556.1	616.86	32.63	0.5111736	0.5631364	0.5486782	0.5731140
33.63	548.91	588.59	580.52	642.43	33.63	0.5502613	0.6320115	0.6166853	0.6383225
34.63	588.22	627.64	613.63	679.89	34.63	0.6612827	0.7402873	0.7088931	0.7338529
35.63	609.67	651.15	638.38	708.92	35.63	0.7218629	0.8054746	0.7778191	0.8078851
36.63	650.05	691.8	679.25	753.71	36.63	0.8359063	0.9181868	0.8916376	0.9221083
37.65	675.27	726.57	706.27	786.44	37.65	0.9071340	1.0145952	0.9668854	1.0055763
38.65	710.1	766.6	746.73	824.8	38.65	1.0055028	1.1255883	1.0795621	1.1034018
39.65	730.24	780.75	762.85	851.45	39.65	1.0623833	1.1648227	1.1244546	1.1713645
Parameter					Paramete	Value			
a	484.03	501.13	475.7	525.99	a	1.367	1.3895	1.3248	1.3414
b	3.8025	3.3699	3.3483	3.2047	b	3.8025	3.3699	3.3483	3.2047
x0	34.935	34.183	34.081	33.93	x0	34.9352	34.1826	34.0809	33.9304
y0	354.08	360.65	359.08	392.13	y0	0	0	0	0
R	0.9988	0.9989	0.9985	0.9987	R	0.99880422	0.99886415	0.99853295	0.9987139
**$CP_{(SDM)}$**	29.93	29.74	29.67	29.71					

Well/ Cycle	Raw fluorescence data E2	E3	E4	E5	Well/ Cycle	E2 Normalized Fluorescence	E3 Normalized Fluorescence	E4 Normalized Fluorescence	E5 Normalized Fluorescence
0.56	108.23	126.72	141	129.32	0.56	-0.0320359	-0.0051938	-0.0071755	-0.0071630
1.56	110.89	126.28	140	125.94	1.56	-0.0082460	-0.0086480	-0.0174464	-0.0331125
2.56	109.04	126.56	139	126.93	2.56	-0.0247916	-0.0064499	-0.0234963	-0.0255119
3.56	111.17	126.04	140	126.39	3.56	-0.0057418	-0.0105321	-0.0168836	-0.0296577
4.56	110.59	125.59	139	127.5	4.56	-0.0109291	-0.0140648	-0.0201899	-0.0211358
5.56	110.8	129.04	140	130.02	5.56	-0.0090509	0.0130191	-0.0171650	-0.0017888
6.56	111.14	127.47	143	130.46	6.56	-0.0060101	0.0006940	0.0088639	0.0015892
7.56	111.9	128.46	144	128.96	7.56	0.0007870	0.0084659	0.0094970	-0.0099268
8.56	112.4	127.75	144	131.49	8.56	0.0052588	0.0028921	0.0113261	0.0094969
9.56	113.66	128.39	145	131.24	9.56	0.0165277	0.0079164	0.0202603	0.0075776
10.56	113.76	127.53	145	133.49	10.56	0.0174221	0.0011650	0.0170946	0.0248516
11.56	114.28	129.38	147	134.07	11.56	0.0220728	0.0156883	0.0329230	0.0293045
12.56	115.22	130.07	144	134.9	12.56	0.0304797	0.0211051	0.0156173	0.0356767
13.56	113.94	130.64	144	132.29	13.56	0.0190319	0.0255798	0.0118185	0.0156388
14.56	115.09	131.01	146	134	14.56	0.0293171	0.0284845	0.0255364	0.0287671
15.56	115.59	131.51	145	134.66	15.56	0.0337889	0.0324097	0.0227928	0.0338342
16.58	116.13	131.11	148	135.15	16.58	0.0386184	0.0292695	0.0417165	0.0375961
17.58	115.64	130.13	147	134.88	17.58	0.0342360	0.0215761	0.0365811	0.0355232
18.58	116.72	131.92	147	134.01	18.58	0.0438951	0.0356284	0.0370735	0.0288439
19.58	116.47	132.06	149	137.31	19.58	0.0416592	0.0367274	0.0481182	0.0541792
20.58	117.43	131.24	148	135.95	20.58	0.0502451	0.0302901	0.0441787	0.0437380
21.58	117.53	134.31	154	140.02	21.58	0.0511394	0.0543909	0.0845586	0.0749848
22.58	120.75	136.23	153	142.3	22.58	0.0799378	0.0694637	0.0780162	0.0924892
23.58	121.61	139.14	156	143.8	23.58	0.0876292	0.0923085	0.0984875	0.1040053
24.58	126.29	142.63	160	148.74	24.58	0.1294852	0.1197065	0.1245867	0.1419315
25.58	129.88	146.69	166	153.6	25.58	0.1615927	0.1515792	0.1705241	0.1792435
26.58	140.16	155.39	178	164.65	26.58	0.2535327	0.2198779	0.2493141	0.2640784
27.58	149.5	170.66	195	178.76	27.58	0.3370658	0.3397539	0.3727049	0.3724060
28.56	162.11	190.45	219	201.56	28.56	0.4498444	0.4951139	0.5407668	0.5474500
29.61	182.39	216.43	251	225.52	29.61	0.6312203	0.6990680	0.7670770	0.7313997
30.56	202.26	241.72	284	251.71	30.56	0.8089293	0.8976053	0.9998593	0.9324699
31.63	219.42	268.04	320	278.27	31.63	0.9624012	1.1042286	1.2518466	1.1363807
32.63	240.31	289.84	350	305.17	32.63	1.1492326	1.2753679	1.4630320	1.3429019
33.63	259.81	320.41	385	340.2	33.63	1.3236325	1.5153554	1.7065072	1.6118400
34.63	276.89	345.31	413	366.13	34.63	1.4763889	1.7108311	1.9085473	1.8109141
35.63	299.34	372.26	449	396.2	35.63	1.6771724	1.9224001	2.1584945	2.0417726
36.63	313.66	388.52	476	416.61	36.63	1.8052445	2.0500480	2.3490679	2.1984676
37.65	331.88	407.83	497	437.78	37.65	1.9681966	2.2016398	2.4979247	2.3609974
38.65	346.39	429.61	523	463.37	38.65	2.0979680	2.3726221	2.6812522	2.5574612
39.65	365.51	454.19	554	483.1	39.65	2.2689693	2.5655856	2.8970102	2.7089357
Parameter					Paramete Value				
a	281.43	349.2124	441	388.4996	a	2.517	2.7415	3.1007	2.9827
b	3.2306	2.9812	2.96	3.0748	b	3.2306	2.9812	2.9601	3.0748
x0	33.332	33.0373	33.1	33.2256	x0	33.3316	33.0373	33.0586	33.2256
y0	111.81	127.3816	142	130.253	y0	0	0	0	0
R	0.9993	0.999223	1	0.999508	R	0.99933057	0.99922339	0.99924734	0.99950843
**$CP_{(SDM)}$**	29.08	29.11	####	29.18					

E2			E3			E4	
Well/ Cycle	AM Normalize Fluorescence	/IC Normalize Fluorescence	Well/ Cycle	FAM Normalized Fluorescence	VIC Normalized Fluorescence	Well/ Cycle	FAM Normalized Fluorescence
0.56	-0.0300100	-0.03203592	0.56	-0.011903683	-0.005193843	0.56	-0.0306628
1.56	-0.0240790	-0.00824598	1.56	0.001405506	-0.008648031	1.56	-0.0059608
2.56	-0.0195037	-0.02479161	2.56	-0.017560088	-0.006449911	2.56	-0.0232271
3.56	-0.0047894	-0.00574178	3.56	-0.002864525	-0.010532133	3.56	-0.0156522
4.56	-0.0178092	-0.01092906	4.56	-0.015092342	-0.014064826	4.56	-0.0137585
5.56	-0.0087433	-0.00905091	5.56	-0.021109204	0.013019149	5.56	-0.0079659
6.56	0.0064229	-0.00601009	6.56	0.010777392	0.000693978	6.56	0.0069333
7.56	-0.0035749	0.000787035	7.56	0.000795501	0.008465901	7.56	-0.0010037
8.56	0.0097838	0.005258827	8.56	0.013522413	0.002892097	8.56	0.0100523
9.56	0.0085694	0.016527743	9.56	-0.000674055	0.007916371	9.56	-0.0037886
10.56	0.0125233	0.017422101	10.56	0.016932892	0.001165003	10.56	0.0259541
11.56	0.0054909	0.022072765	11.56	-0.001533607	0.015688294	11.56	0.0065434
12.56	0.0156017	0.030479734	12.56	0.01662789	0.021105089	12.56	0.0192425
13.56	0.0222387	0.019031946	13.56	0.011304214	0.025579833	13.56	0.0245616
14.56	0.0285933	0.029317068	14.56	0.023476576	0.028484491	14.56	0.0209134
15.56	0.0254019	0.03378886	15.56	0.023032937	0.032409704	15.56	0.0295187
16.58	0.0315870	0.038618395	16.58	0.014492874	0.029269533	16.58	0.0101637
17.58	0.0391278	0.034236039	17.58	0.025057042	0.021576115	17.58	0.0248680
18.58	0.0224929	0.04389511	18.58	0.023504304	0.03562838	18.58	0.0276250
19.58	0.0386759	0.041659214	19.58	0.03013117	0.036727439	19.58	0.0293516
20.58	0.0351456	0.050245054	20.58	0.036758037	0.030290089	20.58	0.0334454
21.58	0.0261362	0.051139413	21.58	0.007311458	0.054390901	21.58	0.0184906
22.58	0.0365295	0.079937753	22.58	0.028550704	0.069463722	22.58	0.0295187
23.58	0.0623714	0.087629235	23.58	0.046989475	0.092308465	23.58	0.0498763
24.58	0.0606768	0.129485207	24.58	0.046074469	0.119706457	24.58	0.0423571
25.58	0.0916589	0.161592673	25.58	0.086251581	0.151579192	25.58	0.0800645
26.58	0.1161169	0.253532716	26.58	0.102943521	0.21987791	26.58	0.0957713
27.58	0.1836449	0.337065789	27.58	0.184074114	0.339753936	27.58	0.1581529
28.56	0.2096280	0.449844382	28.56	0.201681061	0.495113894	28.56	0.1910146
29.61	0.2710555	0.631220263	29.61	0.293264913	0.699067997	29.61	0.2985393
30.56	0.3510666	0.808929274	30.56	0.380079639	0.897605306	30.56	0.3750124
31.63	0.4072128	0.962401173	31.63	0.46661709	1.104228554	31.63	0.4490348
32.63	0.5111736	1.149232641	32.63	0.563136432	1.275367871	32.63	0.5486782
33.63	0.5502613	1.323632526	33.63	0.632011481	1.515355436	33.63	0.6166853
34.63	0.6612827	1.476388939	34.63	0.740287273	1.710831078	34.63	0.7088931
35.63	0.7218629	1.677172397	35.63	0.805474568	1.922400095	35.63	0.7778191
36.63	0.8359063	1.805244518	36.63	0.918186756	2.050048045	36.63	0.8916376
37.65	0.9071340	1.968196616	37.65	1.014595189	2.201639797	37.65	0.9668854
38.65	1.0055028	2.097968018	38.65	1.125588273	2.372622106	38.65	1.0795621
39.65	1.0623833	2.268969341	39.65	1.164822651	2.56558561	39.65	1.1244546

iment derived	linear regre
**parameter**	**value**
y0	0.0234
a	0.4111
**R**	**0.99617478**

riment derived	linear regres
**parameter**	**value**
y0	0.0171
a	0.4132
**R**	**0.99567293**

periment derived	linear regress
**parameter**	**value**
y0	0.0132
a	0.3595
**R**	**0.99790205**

E5

VIC Normalized Fluorescence	Well/ Cycle	AM Normalize Fluorescence	VIC Normalized Fluorescence
-0.007175519	0.56	-0.01338011	-0.007162983
-0.017446359	1.56	-0.00695362	-0.033112481
-0.023496307	2.56	-0.02350439	-0.025511888
-0.016883574	3.56	-0.0079482	-0.029657666
-0.02018994	4.56	-0.00764217	-0.02113579
-0.017164967	5.56	-0.01105944	-0.001788826
0.008863876	6.56	0.004777282	0.001589215
0.00949701	7.56	0.001742549	-0.009926835
0.011326064	8.56	0.02242464	0.009496902
0.020260288	9.56	-0.00231226	0.007577561
0.017094618	10.56	0.010566228	0.024851635
0.032922969	11.56	0.006281898	0.029304507
0.015617306	12.56	0.022118616	0.035676721
0.011818502	13.56	0.021022032	0.015638795
0.025536405	14.56	0.020562997	0.028767092
0.022792824	15.56	0.017069228	0.033834154
0.041716497	16.58	0.020001953	0.037596063
0.036581076	17.58	0.018089306	0.035523174
0.037073514	18.58	0.021200546	0.028843865
0.048118185	19.58	0.023623232	0.054179174
0.044178684	20.58	0.025306361	0.043737956
0.084558565	21.58	0.013243934	0.074984837
0.07801618	22.58	0.021914601	0.092489232
0.098487513	23.58	0.034206546	0.104005282
0.124586704	24.58	0.046523993	0.141931472
0.170524094	25.58	0.069858288	0.179243472
0.249314105	26.58	0.094493183	0.264078371
0.372704889	27.58	0.168652887	0.37240601
0.540766796	28.56	0.181199852	0.547449963
0.767077031	29.61	0.287211506	0.731399661
0.999859304	30.56	0.384577995	0.932469886
1.251846641	31.63	0.458074648	1.136380736
1.463032008	32.63	0.573113996	1.342901891
1.706507211	33.63	0.638322512	1.611840034
1.908547309	34.63	0.733852859	1.810914144
2.158494548	35.63	0.807885053	2.04177255
2.349067886	36.63	0.922108339	2.198467598
2.497924727	37.65	1.005576259	2.360997443
2.681252198	38.65	1.103401783	2.557461248
2.8970102	39.65	1.171364511	2.708935687

nent derived	linear regr
**parameter**	**value**
y0	-0.0161
a	0.426
**R**	**0.99444647**

AVERAGE

nent derived	linear regr
**parameter**	**value**
y0	0.0094
a	0.40245

Well/ Cycle	Raw fluorescence data 6 sampl	7 sample	8 sampl	9 sampl	Well/ Cycle	E6 Normalized Fluorescence	E7 Normalized Fluorescence	E8 Normalized Fluorescence	E9 Normalized Fluorescence
0.56	394.85	409.2	375.4	376.17	0.56	-0.0023326	-0.0016851	-0.0201685	-0.0017276
1.56	392.24	409.56	379.97	376.95	1.56	-0.0089273	-0.0008068	-0.0082403	0.0003423
2.56	384.84	405.72	376.93	373.42	2.56	-0.0276249	-0.0101752	-0.0161751	-0.0090255
3.56	394.26	410.96	375.71	375.77	3.56	-0.0038234	0.0026087	-0.0193594	-0.0027891
4.56	391.53	406.61	376.42	374.49	4.56	-0.0107213	-0.0080038	-0.0175062	-0.0061860
5.56	393.53	408.56	383.32	375.1	5.56	-0.0056679	-0.0032465	0.0005035	-0.0045672
6.56	399.01	409.25	384.46	380.86	6.56	0.0081784	-0.0015631	0.0034790	0.0107186
7.56	392.81	409.73	383.29	376.29	7.56	-0.0074871	-0.0003921	0.0004252	-0.0014092
8.56	402.4	414.28	385.49	382.76	8.56	0.0167439	0.0107085	0.0061674	0.0157608
9.56	396.83	410.76	385.78	378.23	9.56	0.0026702	0.0021208	0.0069243	0.0037392
10.56	404.65	413.16	387.79	380.73	10.56	0.0224290	0.0079760	0.0121706	0.0103736
11.56	396.56	412.01	386.87	380.58	11.56	0.0019880	0.0051704	0.0097693	0.0099756
12.56	405.12	417.17	391.43	382.46	12.56	0.0236166	0.0177591	0.0216714	0.0149647
13.56	404.09	413.35	391.71	380.12	13.56	0.0210141	0.0084396	0.0224022	0.0087548
14.56	401.74	416.61	390.74	382.65	14.56	0.0150763	0.0163929	0.0198704	0.0154689
15.56	403.55	416.3	390.43	381.14	15.56	0.0196496	0.0156366	0.0190613	0.0114617
16.58	403.58	418.62	387.89	378.68	16.58	0.0197254	0.0212967	0.0124316	0.0049334
17.58	403.59	416.36	388.31	382.52	17.58	0.0197507	0.0157830	0.0135279	0.0151239
18.58	396.28	418.73	393.02	379.86	18.58	0.0012805	0.0215650	0.0258215	0.0080648
19.58	401.66	418.52	394.09	382.94	19.58	0.0148742	0.0210527	0.0286143	0.0162385
20.58	406.42	417.95	391.55	383.4	20.58	0.0269013	0.0196621	0.0219846	0.0174592
21.58	397.96	409.54	389.9	374.81	21.58	0.0055254	-0.0008556	0.0176779	-0.0053368
22.58	405.3	414.74	393.1	382.91	22.58	0.0240714	0.0118307	0.0260303	0.0161589
23.58	409.23	421.6	397.46	383.75	23.58	0.0340013	0.0285669	0.0374103	0.0183880
24.58	409.21	421.19	402.1	385.86	24.58	0.0339508	0.0275666	0.0495212	0.0239875
25.58	420.41	433.45	408	396.37	25.58	0.0622498	0.0574770	0.0649208	0.0518787
26.58	423.56	436.86	417.45	400.42	26.58	0.0702089	0.0657963	0.0895862	0.0626266
27.58	455.26	463.84	435.09	419.53	27.58	0.1503053	0.1316187	0.1356284	0.1133403
28.56	459.3	477.01	445.11	427.68	28.56	0.1605131	0.1637493	0.1617816	0.1349686
29.61	491.88	509.61	475.86	456.13	29.61	0.2428330	0.2432827	0.2420421	0.2104686
30.56	525.51	534.4	501.95	480.82	30.56	0.3278059	0.3037622	0.3101396	0.2759905
31.63	551.07	563.38	521.44	504.44	31.63	0.3923884	0.3744640	0.3610105	0.3386727
32.63	589.34	606.78	558.85	532.65	32.63	0.4890852	0.4803459	0.4586543	0.4135359
33.63	612.12	624.35	570.47	545.85	33.63	0.5466434	0.5232109	0.4889837	0.4485658
34.63	646.47	666.93	601.42	576.64	34.63	0.6334355	0.6270923	0.5697663	0.5302756
35.63	667.2	688.23	626.06	591.62	35.63	0.6858140	0.6790574	0.6340791	0.5700293
36.63	715.74	728.44	664.4	620.42	36.63	0.8084600	0.7771567	0.7341504	0.6464581
37.65	744.83	766.9	686.26	651.1	37.65	0.8819617	0.8709866	0.7912071	0.7278761
38.65	778.65	804.62	714.31	678.68	38.65	0.9674147	0.9630111	0.8644204	0.8010674
39.65	805.21	831.22	738.24	694.3	39.65	1.0345238	1.0279065	0.9268801	0.8425194
Parameter					Parameter	Value			
a	480.36	498.845	424.22	364.78	a	1.2137	1.217	1.1073	0.968
b	3.2837	3.2775	3.4758	3.2111	b	3.2837	3.2775	3.4758	3.2111
x0	34.2861	34.4796	34.292	33.99	x0	34.2861	34.4796	34.2918	33.9898
y0	395.773	409.891	383.13	376.82	y0	0	0	0	0
R	0.9984	0.9986	0.9986	0.9982	R	0.99840242	0.99859654	0.99861586	0.9981963
**$CP_{(SDM)}$**	29.96	30.16	29.71	29.76					

Well/ Cycle	Raw fluorescence data E6	E7	E8	E9
0.56	146.72	145	150.84	127.36
1.56	146.1	143.69	151.51	126.85
2.56	146.3	147.19	151.21	129.01
3.56	147.51	148.94	151.4	129.07
4.56	149.05	147.01	154.94	128.46
5.56	147.95	147.55	152.08	129.85
6.56	148.78	148.6	151.71	130.32
7.56	150.76	148.83	157.34	130.79
8.56	149.1	148.55	158.34	131.18
9.56	149.82	150.13	155.59	132.1
10.56	149.03	149.97	155.51	131.63
11.56	148.61	150.46	156.82	132.53
12.56	149.92	150.14	157.82	130.77
13.56	149.05	150.7	156.52	132.75
14.56	150.73	149.85	160.85	131.82
15.56	154.48	151.55	157.65	133.31
16.58	152.11	150.31	155.82	130.75
17.58	153.98	150.1	155.7	131.81
18.58	151.82	149.22	158.07	133.33
19.58	154.45	151.48	161.27	132.8
20.58	154.82	151.3	160.65	135.39
21.58	156.53	155.54	163.25	135.06
22.58	157.62	155.52	164.91	136.96
23.58	156.91	159.3	166.98	138.83
24.58	165.44	163.24	172.48	142.91
25.58	170.87	171.28	182.96	147.4
26.58	189.03	187.3	198.93	162.14
27.58	205.36	206.39	219.38	173.98
28.56	235.2	232.9	254.18	200.98
29.61	270.01	272.33	286.47	224.42
30.56	304.84	310.53	323.42	248.97
31.63	344.9	350.98	364.44	273.85
32.63	382.4	380.36	398.7	295.14
33.63	420.5	422.86	437.16	325.68
34.63	450.23	452.5	471.02	350.55
35.63	485.23	488.55	506.31	374.64
36.63	513.29	516.95	536.1	396.85
37.65	537.16	545.49	559.92	413.03
38.65	567.19	576.75	588.88	430.92
39.65	592.72	594.68	614.28	455.16
Parameter				
a	468.09	475.9454	488.3224	346.2049
b	2.8256	2.8348	2.9079	2.9742
x0	32.739	32.7456	32.6515	32.8062
y0	147.79	146.7892	153.3446	129.1417
R	0.9993	0.999216	0.999324	0.999138
**CP$_{(SDM)}$**	29.02	29.01	28.82	28.89

Well/ Cycle	E6 Normalized Fluorescence	E7 Normalized Fluorescence	E8 Normalized Fluorescence	E9 Normalized Fluorescence
0.56	-0.0072575	-0.0121889	-0.0163331	-0.0137965
1.56	-0.0114525	-0.0211133	-0.0119639	-0.0177456
2.56	-0.0100993	0.0027304	-0.0139203	-0.0010198
3.56	-0.0019121	0.0146523	-0.0126812	-0.0005552
4.56	0.0085079	0.0015042	0.0104040	-0.0052787
5.56	0.0010650	0.0051829	-0.0082468	0.0054847
6.56	0.0066810	0.0123361	-0.0106597	0.0091241
7.56	0.0200781	0.0139029	0.0260550	0.0127635
8.56	0.0088462	0.0119954	0.0325763	0.0157834
9.56	0.0137179	0.0227592	0.0146428	0.0229074
10.56	0.0083725	0.0216692	0.0141211	0.0192680
11.56	0.0055307	0.0250073	0.0226640	0.0262371
12.56	0.0143945	0.0228273	0.0291852	0.0126086
13.56	0.0085079	0.0266423	0.0207076	0.0279406
14.56	0.0198751	0.0208517	0.0489447	0.0207392
15.56	0.0452485	0.0324329	0.0280766	0.0322769
16.58	0.0292126	0.0239854	0.0161427	0.0124538
17.58	0.0418654	0.0225548	0.0153602	0.0206618
18.58	0.0272503	0.0165598	0.0308156	0.0324318
19.58	0.0450456	0.0319560	0.0516836	0.0283278
20.58	0.0475491	0.0307298	0.0476404	0.0483833
21.58	0.0591193	0.0596147	0.0645957	0.0458280
22.58	0.0664945	0.0594785	0.0754210	0.0605405
23.58	0.0616905	0.0852297	0.0889200	0.0750207
24.58	0.1194065	0.1120709	0.1247869	0.1066139
25.58	0.1561472	0.1668433	0.1931297	0.1413819
26.58	0.2790221	0.2759794	0.2972742	0.2555201
27.58	0.3895148	0.4060299	0.4306340	0.3472023
28.56	0.5914193	0.5866290	0.6575739	0.5562750
29.61	0.8269521	0.8552455	0.8681453	0.7377811
30.56	1.0626202	1.1154826	1.1091059	0.9278823
31.63	1.3336757	1.3910478	1.3766080	1.1205389
32.63	1.5874097	1.5911988	1.6000263	1.2853966
33.63	1.8452033	1.8807296	1.8508340	1.5218810
34.63	2.0463636	2.0826519	2.0716439	1.7144602
35.63	2.2831820	2.3282421	2.3017791	1.9009994
36.63	2.4730426	2.5217169	2.4960475	2.0729811
37.65	2.6345527	2.7161453	2.6513839	2.1982698
38.65	2.8377429	2.9291038	2.8402396	2.3367998
39.65	3.0104850	3.0512517	3.0058796	2.5245006

E6			E7			E8	
Well/ Cycle	FAM Normalized Fluorescence	VIC Normalized Fluorescence	Well/ Cycle	AM Normalize Fluorescence	VIC Normalized Fluorescence	Well/ Cycle	FAM Normalized Fluorescence
0.56	-0.0023326	-0.007257468	0.56	-0.001685083	-0.012188908	0.56	-0.0201685
1.56	-0.0089273	-0.011452536	1.56	-0.0008068	-0.02111327	1.56	-0.0082403
2.56	-0.0276249	-0.010099288	2.56	-0.010175152	0.002730446	2.56	-0.0161751
3.56	-0.0038234	-0.001912139	3.56	0.002608744	0.014652304	3.56	-0.0193594
4.56	-0.0107213	0.008507868	4.56	-0.008003841	0.001504198	4.56	-0.0175062
5.56	-0.0056679	0.001065006	5.56	-0.003246475	0.005182943	5.56	0.0005035
6.56	0.0081784	0.006680984	6.56	-0.0015631	0.012336057	6.56	0.0034790
7.56	-0.0074871	0.020078137	7.56	-0.000392056	0.01390293	7.56	0.0004252
8.56	0.0167439	0.00884618	8.56	0.010708464	0.011995433	8.56	0.0061674
9.56	0.0026702	0.013717872	9.56	0.002120809	0.022759168	9.56	0.0069243
10.56	0.0224290	0.008372544	10.56	0.007976029	0.021669169	10.56	0.0121706
11.56	0.0019880	0.005530723	11.56	0.005170403	0.025007289	11.56	0.0097693
12.56	0.0236166	0.014394496	12.56	0.017759125	0.022827292	12.56	0.0216714
13.56	0.0210141	0.008507868	13.56	0.008439567	0.026642287	13.56	0.0224022
14.56	0.0150763	0.019875149	14.56	0.016392907	0.02085167	14.56	0.0198704
15.56	0.0196496	0.045248544	15.56	0.015636608	0.032432904	15.56	0.0190613
16.58	0.0197254	0.029212559	16.58	0.021296653	0.023985416	16.58	0.0124316
17.58	0.0197507	0.041865425	17.58	0.015782988	0.022554793	17.58	0.0135279
18.58	0.0012805	0.027250349	18.58	0.021565017	0.016559801	18.58	0.0258215
19.58	0.0148742	0.045045557	19.58	0.021052686	0.031956029	19.58	0.0286143
20.58	0.0269013	0.047549065	20.58	0.019662071	0.030729781	20.58	0.0219846
21.58	0.0055254	0.059119333	21.58	-0.000855594	0.05961474	21.58	0.0176779
22.58	0.0240714	0.066494534	22.58	0.011830715	0.05947849	22.58	0.0260303
23.58	0.0340013	0.061690504	23.58	0.028566884	0.085229704	23.58	0.0374103
24.58	0.0339508	0.11940652	24.58	0.027566617	0.112070915	24.58	0.0495212
25.58	0.0622498	0.156147195	25.58	0.05747703	0.166843337	25.58	0.0649208
26.58	0.0702089	0.279022089	26.58	0.065796321	0.275979432	26.58	0.0895862
27.58	0.1503053	0.389514766	27.58	0.131618746	0.406029871	27.58	0.1356284
28.56	0.1605131	0.591419327	28.56	0.163749263	0.586628989	28.56	0.1617816
29.61	0.2428330	0.826952094	29.61	0.24328266	0.855245481	29.61	0.2420421
30.56	0.3278059	1.062620185	30.56	0.303762198	1.11548261	30.56	0.3101396
31.63	0.3923884	1.333675705	31.63	0.374463973	1.391047843	31.63	0.3610105
32.63	0.4890852	1.587409654	32.63	0.480345858	1.591198808	32.63	0.4586543
33.63	0.5466434	1.845203346	33.63	0.523210944	1.880729645	33.63	0.4889837
34.63	0.6334355	2.04636362	34.63	0.627092296	2.082651857	34.63	0.5697663
35.63	0.6858140	2.283181973	35.63	0.679057368	2.328242132	35.63	0.6340791
36.63	0.8084600	2.473042629	36.63	0.777156691	2.521716857	36.63	0.7341504
37.65	0.8819617	2.634552745	37.65	0.870986583	2.716145329	37.65	0.7912071
38.65	0.9674147	2.837742891	38.65	0.963011115	2.929103776	38.65	0.8644204
39.65	1.0345238	3.010484963	39.65	1.027906464	3.051251727	39.65	0.9268801

nent derived	linear regr
aramet	value
y0	-0.0009
a	0.303
**R**	**0.9928678**

nent derived	linear regr
paramete	value
y0	-0.0049
a	0.2889
**R**	**0.99289059**

riment derived	linear regres
paramet	value
y0	0.0039
a	0.2731
**R**	**0.99350397**

E9

VIC Normalized Fluorescence	Well/ Cycle	**FAM** Normalized Fluorescence	VIC Normalized Fluorescence
-0.016333148	0.56	-0.001727611	-0.013796473
-0.011963904	1.56	0.000342338	-0.017745624
-0.013920282	2.56	-0.009025505	-0.00101981
-0.012681242	3.56	-0.002789123	-0.000555204
0.010404018	4.56	-0.006185961	-0.005278698
-0.008246785	5.56	-0.004567155	0.005484673
-0.010659652	6.56	0.010718617	0.009124086
0.026055042	7.56	-0.001409157	0.012763499
0.032576302	8.56	0.015760799	0.015783438
0.014642837	9.56	0.003739176	0.022907396
0.014121136	10.56	0.010373626	0.019267982
0.022663987	11.56	0.009975559	0.026237071
0.029185247	12.56	0.014964665	0.012608631
0.020707609	13.56	0.00875482	0.027940626
0.048944665	14.56	0.015468883	0.020739234
0.028076633	15.56	0.011461675	0.032276948
0.016142727	16.58	0.004933377	0.012453762
0.015360176	17.58	0.015123892	0.0206618
0.030815562	18.58	0.008064837	0.032431817
0.051683594	19.58	0.016238479	0.028327798
0.047640413	20.58	0.017459218	0.048383288
0.064595688	21.58	-0.005336751	0.045827955
0.07542098	22.58	0.016158866	0.060540476
0.088919988	23.58	0.018388041	0.075020694
0.124786918	24.58	0.023987517	0.106613898
0.193129722	25.58	0.051878743	0.141381908
0.297274244	26.58	0.062626552	0.255520099
0.43063401	27.58	0.113340286	0.347202337
0.657573857	28.56	0.134968593	0.556275006
0.868145341	29.61	0.210468631	0.737781058
1.109105896	30.56	0.275990457	0.927882318
1.37660798	31.63	0.338672739	1.120538912
1.600026346	32.63	0.41353587	1.285396584
1.850834004	33.63	0.448565765	1.521881004
2.071643866	34.63	0.530275648	1.714460163
2.30177913	35.63	0.570029271	1.900999445
2.496047464	36.63	0.646458133	2.072981074
2.651383877	37.65	0.7278761	2.198269808
2.840239565	38.65	0.80106735	2.33679981
3.005879568	39.65	0.842519392	2.524500607

**periment derived**	linear regressi
**paramete**	**value**
y0	-0.0235
a	0.3277
**R**	**0.99252131**

AVERAGE

**periment derived**	linear regressi
**paramete**	**value**
y0	-0.00635
a	0.298175
**R**	

Well/ Cycle	Raw fluorescence data F2	F3	F4	F5	Well/ Cycle	F2 Normalized Fluorescence	F3 Normalized Fluorescence	F4 Normalized Fluorescence	F5 Normalized Fluorescence
0.56	340.41	337.67	344.77	362.54	0.56	-0.0230094	-0.0103853	-0.0335254	-0.0456598
1.56	343.44	338.53	353.31	368.33	1.56	-0.0143132	-0.0078649	-0.0095857	-0.0304184
2.56	338.41	338.34	349.59	369.29	2.56	-0.0287495	-0.0084217	-0.0200138	-0.0278913
3.56	346.15	340.59	355.94	373.92	3.56	-0.0065354	-0.0018276	-0.0022132	-0.0157034
4.56	345.96	339.96	352.16	372.31	4.56	-0.0070807	-0.0036739	-0.0128094	-0.0199415
5.56	344.24	335.64	351.75	375.63	5.56	-0.0120171	-0.0163346	-0.0139588	-0.0112021
6.56	350.48	348.05	360.54	379.8	6.56	0.0058919	0.0200355	0.0106818	-0.0002251
7.56	347.62	344	355.41	379.19	7.56	-0.0023164	0.0081661	-0.0036989	-0.0018308
8.56	351.32	346.75	358.93	385.57	8.56	0.0083027	0.0162256	0.0061685	0.0149637
9.56	346.6	337.08	356.35	382.59	9.56	-0.0052439	-0.0121144	-0.0010638	0.0071193
10.56	352.07	340.47	360.02	388.31	10.56	0.0104553	-0.0021793	0.0092241	0.0221764
11.56	351.39	339.84	359.09	383.77	11.56	0.0085036	-0.0040256	0.0066171	0.0102254
12.56	354.62	346.04	362.8	390.09	12.56	0.0177739	0.0141448	0.0170171	0.0268620
13.56	355.12	345.39	360.93	388.58	13.56	0.0192089	0.0122398	0.0117750	0.0228872
14.56	354.51	345.81	366.99	396.21	14.56	0.0174582	0.0134707	0.0287627	0.0429722
15.56	356.87	345.34	363.9	390.31	15.56	0.0242315	0.0120933	0.0201007	0.0274412
16.58	357.94	349.46	367.33	392.69	16.58	0.0273024	0.0241679	0.0297158	0.0337062
17.58	358.89	344.35	362.55	394.6	17.58	0.0300290	0.0091919	0.0163163	0.0387340
18.58	359.84	343.54	366.39	396.88	18.58	0.0327555	0.0068180	0.0270807	0.0447358
19.58	356.34	348.49	367.92	397.48	19.58	0.0227103	0.0213251	0.0313697	0.0463153
20.58	357.76	348.99	364.81	397.71	20.58	0.0267858	0.0227904	0.0226516	0.0469207
21.58	352.02	343.48	363.61	391.73	21.58	0.0103118	0.0066422	0.0192877	0.0311791
22.58	356.82	345.45	361.99	396.95	22.58	0.0240880	0.0124157	0.0147465	0.0449201
23.58	365.29	352.36	371.49	403.28	23.58	0.0483972	0.0326669	0.0413773	0.0615830
24.58	360.71	345.28	368.93	402.62	24.58	0.0352524	0.0119175	0.0342010	0.0598457
25.58	371.74	354.23	377.96	411.8	25.58	0.0669090	0.0381474	0.0595143	0.0840108
26.58	376.73	362.48	383.11	416.5	26.58	0.0812305	0.0623258	0.0739510	0.0963830
27.58	398.16	375.18	398.65	435.86	27.58	0.1427355	0.0995459	0.1175134	0.1473457
28.56	401.46	376.53	408.99	433.2	28.56	0.1522066	0.1035023	0.1464990	0.1403436
29.61	424.16	403.08	426.31	458.06	29.61	0.2173565	0.1813128	0.1950512	0.2057844
30.56	447.57	418.32	448.27	470.17	30.56	0.2845442	0.2259769	0.2566104	0.2376624
31.63	464.76	437.5	466.42	484.87	31.63	0.3338802	0.2821880	0.3074893	0.2763583
32.63	481.92	460.01	491.55	516.18	32.63	0.3831301	0.3481585	0.3779348	0.3587778
33.63	500.01	475.63	506.79	528.89	33.63	0.4350491	0.3939362	0.4206563	0.3922353
34.63	528.72	499.49	534.08	556.26	34.63	0.5174480	0.4638631	0.4971568	0.4642833
35.63	545.64	513.53	551.25	567.35	35.63	0.5660091	0.5050104	0.5452885	0.4934763
36.63	573.95	544.14	584.62	600.25	36.63	0.6472599	0.5947196	0.6388328	0.5800814
37.65	596.66	562.83	610.34	618.26	37.65	0.7124386	0.6494946	0.7109322	0.6274904
38.65	616.88	590.26	638.88	646.23	38.65	0.7704708	0.7298842	0.7909368	0.7011178
39.65	634.4	607.24	654.42	665.2	39.65	0.8207539	0.7796477	0.8344992	0.7510539

Parameter	F2	F3	F4	F5	Parameter	Value F2	F3	F4	F5
a	352.93	322.45	387.05	438.62	a	1.0129	0.95	1.09	1.154600
b	3.64	3.34	3.67	4.60	b	3.6415	3.34	3.67	4.595400
x0	34.50	34.77	35.24	36.72	x0	34.4997	34.77	35.24	36.724100
y0	348.43	341.21	356.73	379.89	y0	0.0000	0.00	0.00	0.000000
R	1.00	1.00	1.00	1.00	R	0.998273	0.998195	0.998352	0.996759

	F2	F3	F4	F5
**$CP_{(SDM)}$**	29.70	30.37	30.41	30.67

Well/ Cycle	Raw fluorescence data F2	F3	F4	F5	Well/ Fluorescence	F2 Normalized	F3 Normalized predicted	F4 Normalized predicted	E5 Normalized predicted	E5 Fluorescence
0.6	113.11	116.15	120.16	117.41	0.56	-0.0200495	-0.0026208	-0.0301622	-0.0249691	0.0001191
1.6	114.02	117.12	123.22	118.22	1.56	-0.0121656	0.0057086	-0.0054642	-0.0182425	0.0001627
2.6	115.77	116.34	124.58	118.82	2.56	0.0029959	-0.0009892	0.0055126	-0.0132598	0.0002224
3.6	115.18	115.37	122.76	118.64	3.56	-0.0021157	-0.0093186	-0.0091770	-0.0147546	0.0003040
4.6	114.86	116.83	124.54	120.17	4.56	-0.0048881	0.0032184	0.0051898	-0.0020487	0.0004157
5.6	114.13	117.85	125.38	120.72	5.56	-0.0112126	0.0119771	0.0119696	0.0025188	0.0005683
6.6	116.03	117.54	124.77	121.3	6.56	0.0052485	0.0093152	0.0070462	0.0073354	0.0007771
7.6	116.53	117.87	126.05	120.95	7.56	0.0095803	0.0121489	0.0173773	0.0044288	0.0010625
8.6	116.16	116.29	124.74	121.5	8.56	0.0063747	-0.0014186	0.0068040	0.0089963	0.0014528
9.6	118.75	118.32	125.69	121.6	9.56	0.0288137	0.0160130	0.0144717	0.0098267	0.0019863
11	116.49	119.03	125.82	121.5	10.56	0.0092338	0.0221098	0.0155210	0.0089963	0.0027158
12	117.2	118.2	127.59	124.01	11.56	0.0153850	0.0149826	0.0298070	0.0298405	0.0037127
13	118.36	118.77	126.43	123.1	12.56	0.0254349	0.0198772	0.0204444	0.0222835	0.0050751
14	117.51	118.84	126.38	123.64	13.56	0.0180707	0.0204783	0.0200408	0.0267679	0.0069362
15	119.28	118.68	126.37	123.76	14.56	0.0334055	0.0191043	0.0199601	0.0277644	0.0094775
16	118.55	119.09	127.82	123.4	15.56	0.0270810	0.0226250	0.0316634	0.0247748	0.0129457
17	118.17	118.61	128.62	125.44	16.58	0.0237888	0.0185033	0.0381204	0.0417160	0.0177853
18	118.32	118.89	128.28	124.19	17.58	0.0250883	0.0209076	0.0353762	0.0313354	0.0242674
19	121.7	119.28	129.19	126.66	18.58	0.0543716	0.0242565	0.0427210	0.0518475	0.0330841
20	121.09	119.11	127.12	126.73	19.58	0.0490868	0.0227967	0.0260135	0.0524288	0.0450521
21	121.88	121.74	129.19	127.24	20.58	0.0559311	0.0453805	0.0427210	0.0566641	0.0612542
22	123.11	122.16	129.85	127.79	21.58	0.0665874	0.0489871	0.0480480	0.0612315	0.0831080
23	126.41	122.31	132.63	133.25	22.58	0.0951776	0.0502751	0.0704860	0.1065741	0.1124397
24	127.31	126.75	133.33	135.27	23.58	0.1029749	0.0884014	0.0761358	0.1233492	0.1515485
25	132.87	129.6	140.24	140.96	24.58	0.1511451	0.1128743	0.1319080	0.1706018	0.2032356
26	138.43	136.12	144.22	146.91	25.58	0.1993152	0.1688615	0.1640314	0.2200135	0.2707560
27	152.32	147.04	159.43	159.79	26.58	0.3196539	0.2626315	0.2867947	0.3269754	0.3576319
28	167.14	160.5	176.23	173.96	27.58	0.4480499	0.3782124	0.4223912	0.4446501	0.4672548
29	195.29	184.53	202.12	194.53	28.56	0.6919329	0.5845578	0.6313551	0.6154736	0.5992723
30	224.84	213.3	235.68	217.13	29.61	0.9479451	0.8316056	0.9022252	0.8031552	0.7687264
31	253.19	239.62	265.8	240.3	30.56	1.1935608	1.0576153	1.1453304	0.9955704	0.9454392
32	281.23	268.75	296.84	265.78	31.63	1.4364908	1.3077544	1.3958611	1.2071689	1.1656390
33	306.77	290.84	324.55	286.44	32.63	1.6577615	1.4974411	1.6195146	1.3787398	1.3832680
34	334.39	321.78	357.16	314.08	33.63	1.8970528	1.7631226	1.8827171	1.6082761	1.6019590
35	357.81	339.91	383.89	334.13	34.63	2.0999565	1.9188048	2.0984608	1.7747812	1.8113475
36	383.7	365.53	408.74	358.76	35.63	2.3242596	2.1388036	2.2990306	1.9793210	2.0027535
37	401.72	386.03	434.17	379.05	36.63	2.4803793	2.3148370	2.5042818	2.1478192	2.1704527
38	419.25	398.93	451.8	394.67	37.65	2.6322539	2.4256092	2.6465774	2.2775354	2.3145686
39	439.42	419.85	475.32	413.98	38.65	2.8070006	2.6052491	2.8364125	2.4378952	2.4298436
40	457.8	436.29	496.01	430.72	39.65	2.9662393	2.7464192	3.0034061	2.5769125	2.5216728
Parameter										
a	356.20	331.22	387.61	338.44						
b	2.87	2.76	2.82	3.19						
x0	32.23	32.33	32.46	32.73						
y0	115.42	116.46	123.90	120.42						
R	1.00	1.00	1.00	1.00						
$CP_{(SDM)}$	28.45	28.68	28.75	28.52						

F2			F3			F4	
Well/ Cycle	**FAM** Normalized Fluorescence	VIC Normalized Fluorescence	Well/ Cycle	**FAM** Normalized Fluorescence	VIC Normalized Fluorescence	Well/ Cycle	**FAM** Normalized Fluorescence
0.56	-0.0230094	-0.020049522	0.56	-0.010385284	-0.00262075	0.56	-0.0335254
1.56	-0.0143132	-0.01216556	1.56	-0.007864868	0.005708633	1.56	-0.0095857
2.56	-0.0287495	0.002995906	2.56	-0.008421704	-0.000989222	2.56	-0.0200138
3.56	-0.0065354	-0.002115674	3.56	-0.001827594	-0.009318605	3.56	-0.0022132
4.56	-0.0070807	-0.004888056	4.56	-0.003673945	0.003218405	4.56	-0.0128094
5.56	-0.0120171	-0.011212553	5.56	-0.016334636	0.011977138	5.56	-0.0139588
6.56	0.0058919	0.005248466	6.56	0.020035544	0.00931517	6.56	0.0106818
7.56	-0.0023164	0.009580313	7.56	0.008166146	0.012148878	7.56	-0.0036989
8.56	0.0083027	0.006374746	8.56	0.016225614	-0.001418571	8.56	0.0061685
9.56	-0.0052439	0.028813715	9.56	-0.012114406	0.016013025	9.56	-0.0010638
10.56	0.0104553	0.009233766	10.56	-0.00217928	0.02210979	10.56	0.0092241
11.56	0.0085036	0.015384989	11.56	-0.004025631	0.014982586	11.56	0.0066171
12.56	0.0177739	0.025434874	12.56	0.014144805	0.019877172	12.56	0.0170171
13.56	0.0192089	0.018070734	13.56	0.01223984	0.020478261	13.56	0.0117750
14.56	0.0174582	0.033405473	14.56	0.013470741	0.019104342	14.56	0.0287627
15.56	0.0242315	0.027080976	15.56	0.012093305	0.022625009	15.56	0.0201007
16.58	0.0273024	0.023788772	16.58	0.024167853	0.018503253	16.58	0.0297158
17.58	0.0300290	0.025088326	17.58	0.009191896	0.020907611	17.58	0.0163163
18.58	0.0327555	0.054371614	18.58	0.006818017	0.024256538	18.58	0.0270807
19.58	0.0227103	0.04908676	19.58	0.021325059	0.022796749	19.58	0.0313697
20.58	0.0267858	0.055931079	20.58	0.022790416	0.045380541	20.58	0.0226516
21.58	0.0103118	0.066587423	21.58	0.006642174	0.048987078	21.58	0.0192877
22.58	0.0240880	0.095177614	22.58	0.012415683	0.050275127	22.58	0.0147465
23.58	0.0483972	0.102974939	23.58	0.032666928	0.088401377	23.58	0.0413773
24.58	0.0352524	0.15114508	24.58	0.011917462	0.112874307	24.58	0.0342010
25.58	0.0669090	0.199315222	25.58	0.038147366	0.168861502	25.58	0.0595143
26.58	0.0812305	0.319653937	26.58	0.062325769	0.262631467	26.58	0.0739510
27.58	0.1427355	0.448049889	27.58	0.099545856	0.378212394	27.58	0.1175134
28.56	0.1522066	0.691932888	28.56	0.103502322	0.584557839	28.56	0.1464990
29.61	0.2173565	0.947945058	29.61	0.18131282	0.83160563	29.61	0.1950512
30.56	0.2845442	1.193560796	30.56	0.225976925	1.057615289	30.56	0.2566104
31.63	0.3338802	1.436490788	31.63	0.282188049	1.307754398	31.63	0.3074893
32.63	0.3831301	1.657761544	32.63	0.348158456	1.497441076	32.63	0.3779348
33.63	0.4350491	1.897052784	33.63	0.393936232	1.763122643	33.63	0.4206563
34.63	0.5174480	2.099956508	34.63	0.463863105	1.918804828	34.63	0.4971568
35.63	0.5660091	2.324259557	35.63	0.505010351	2.138803591	35.63	0.5452885
36.63	0.6472599	2.480379331	36.63	0.594719554	2.31483695	36.63	0.6388328
37.65	0.7124386	2.632253895	37.65	0.649494627	2.425609161	37.65	0.7109322
38.65	0.7704708	2.807000612	38.65	0.729884155	2.605249057	38.65	0.7909368
39.65	0.8207539	2.966239315	39.65	0.779647705	2.746419224	39.65	0.8344992

**eriment derived**	linear regres
**parameter**	**value**
y0	0.0209
a	0.2168
**R**	**0.99390489**

**riment derived**	linear regre
**paramet**	**value**
y0	-0.0016
a	0.2223
**R**	**0.99218473**

**riment derived**	linear regres
**paramet**	**value**
y0	0.0169
a	0.2131
**R**	**0.99598589**

F5

VIC Normalized Fluorescence	Well/ Cycle	FAM Normalized Fluorescence	VIC Normalized Fluorescence
-0.030162151	0.56	-0.045659811	-0.024969128
-0.005464216	1.56	-0.030418376	-0.018242486
0.005512644	2.56	-0.027891299	-0.013259789
-0.009176978	3.56	-0.015703416	-0.014754598
0.005189795	4.56	-0.019941535	-0.002048719
0.01196962	5.56	-0.01120206	0.002518754
0.007046175	6.56	-0.000225068	0.007335361
0.017377338	7.56	-0.001830815	0.004428788
0.006804039	8.56	0.014963719	0.00899626
0.014471698	9.56	0.00711925	0.00982671
0.015520957	10.56	0.022176419	0.00899626
0.029807017	11.56	0.01022545	0.029840545
0.020444401	12.56	0.026862041	0.022283454
0.02004084	13.56	0.022887159	0.026767882
0.019960128	14.56	0.042972159	0.027764421
0.031663398	15.56	0.027441163	0.024774803
0.038120374	16.58	0.033706209	0.041715975
0.035376159	17.58	0.03873404	0.031335355
0.04272097	18.58	0.044735848	0.05184746
0.026013544	19.58	0.046315271	0.052428774
0.04272097	20.58	0.046920717	0.056664067
0.048047975	21.58	0.031179132	0.06123154
0.070485968	22.58	0.044920114	0.106574088
0.076135822	23.58	0.06158303	0.12334917
0.131907956	24.58	0.059845664	0.170601752
0.164031413	25.58	0.08401084	0.220013503
0.286794676	26.58	0.096382989	0.326975411
0.42239118	27.58	0.147345713	0.444650119
0.631355077	28.56	0.140343604	0.615473601
0.902225235	29.61	0.205784375	0.80315521
1.145330395	30.56	0.237662401	0.995570382
1.395861078	31.63	0.276358271	1.207168939
1.619514597	32.63	0.358777842	1.378739826
1.882717096	33.63	0.392235292	1.608276095
2.098460818	34.63	0.464283317	1.774781239
2.299030646	35.63	0.493476324	1.979320975
2.504281782	36.63	0.580081367	2.147819198
2.646577399	37.65	0.627490389	2.277535425
2.836412504	38.65	0.701117837	2.437895242
3.003406055	39.65	0.751053936	2.576912505

periment derived	linear regressi
paramete	value
y0	0.0279
a	0.2209
R	0.98227916

AVERAGE

periment derived	linear regressi
paramete	value
y0	0.016025
a	0.218275
R	

Well/ Cycle	Raw fluorescence data F6	F7	F8	F9	Well/ Cycle	F6 Normalized Fluorescence	F7 Normalized Fluorescence	F8 Normalized Fluorescence	F9 Normalized Fluorescence
0.56	356.66	346.39	357.14	336.73	0.56	-0.0252496	-0.0109831	-0.0164952	-0.0196352
1.56	363.8	345.74	353.88	336.57	1.56	-0.0057360	-0.0128390	-0.0254727	-0.0201011
2.56	355.09	344.03	354.18	336.3	2.56	-0.0295404	-0.0177214	-0.0246466	-0.0208872
3.56	362.48	342.51	358.88	338.54	3.56	-0.0093436	-0.0220614	-0.0117035	-0.0143656
4.56	360.1	344.01	358.96	340.95	4.56	-0.0158481	-0.0177785	-0.0114832	-0.0073490
5.56	362.32	343.74	362.72	339.02	5.56	-0.0097808	-0.0185495	-0.0011288	-0.0129681
6.56	363.86	350.31	362.25	342.72	6.56	-0.0055720	0.0002093	-0.0024231	-0.0021958
7.56	361.54	345.21	360.14	345.19	7.56	-0.0119126	-0.0143523	-0.0082337	0.0049954
8.56	368.84	352.09	363.77	346.57	8.56	0.0080383	0.0052916	0.0017627	0.0090132
9.56	367.98	351.45	367.21	346.75	9.56	0.0056879	0.0034642	0.0112359	0.0095373
10.56	370.95	351.78	367.39	347.68	10.56	0.0138049	0.0044064	0.0117316	0.0122449
11.56	365.31	353.47	367.01	345.39	11.56	-0.0016092	0.0092318	0.0106852	0.0055777
12.56	372.26	357.93	371.96	349.68	12.56	0.0173851	0.0219660	0.0243166	0.0180677
13.56	373.93	361.57	367.56	350.96	13.56	0.0219492	0.0323590	0.0121998	0.0217944
14.56	375.18	363.11	370.91	352.87	14.56	0.0253655	0.0367560	0.0214251	0.0273552
15.56	372.08	356.79	369.34	345.58	15.56	0.0168932	0.0187111	0.0171016	0.0061309
16.58	374.41	356.42	366.76	353.18	16.58	0.0232611	0.0176546	0.0099967	0.0282577
17.58	371.28	361.4	369.57	351.19	17.58	0.0147068	0.0318736	0.0177350	0.0224640
18.58	376.79	359.26	372	352.23	18.58	0.0297656	0.0257634	0.0244268	0.0254919
19.58	372.88	359.73	371.59	351.1	19.58	0.0190796	0.0271054	0.0232977	0.0222020
20.58	377.37	360.11	375.67	354.22	20.58	0.0313507	0.0281904	0.0345334	0.0312856
21.58	374.03	356.54	369.87	353.61	21.58	0.0222225	0.0179973	0.0185611	0.0295096
22.58	374.09	354.42	372.9	354.04	22.58	0.0223865	0.0119442	0.0269052	0.0307616
23.58	378.45	360.39	373.07	355.35	23.58	0.0343024	0.0289898	0.0273734	0.0345755
24.58	377.5	362.67	372.8	356.5	24.58	0.0317060	0.0354997	0.0266299	0.0379237
25.58	383.52	366.6	385.65	364.51	25.58	0.0481587	0.0467207	0.0620167	0.0612442
26.58	385.06	370.59	383.06	366.23	26.58	0.0523675	0.0581130	0.0548842	0.0662518
27.58	400.98	380.44	398.29	376.44	27.58	0.0958768	0.0862368	0.0968251	0.0959775
28.56	404.58	388.8	401.33	387.63	28.56	0.1057156	0.1101064	0.1051968	0.1285564
29.61	419.24	403.3	416.36	397.19	29.61	0.1457813	0.1515070	0.1465869	0.1563896
30.56	433.84	418.46	428.9	409.89	30.56	0.1856830	0.1947920	0.1811200	0.1933647
31.63	450.17	429.31	445.61	422.61	31.63	0.2303129	0.2257710	0.2271366	0.2303981
32.63	467.46	442.59	460.32	430.69	32.63	0.2775664	0.2636882	0.2676455	0.2539224
33.63	479.29	455.86	469.69	441.79	33.63	0.3098977	0.3015769	0.2934490	0.2862393
34.63	498.35	471.39	482.14	458.79	34.63	0.3619886	0.3459183	0.3277342	0.3357335
35.63	508.23	480.33	498.47	457.32	35.63	0.3889906	0.3714439	0.3727044	0.3314537
36.63	538.34	495.92	510.17	473.68	36.63	0.4712811	0.4159567	0.4049242	0.3790847
37.65	552.89	516.45	522.12	490.62	37.65	0.5110462	0.4745742	0.4378326	0.4284042
38.65	577.01	532.28	544.32	500.84	38.65	0.5769661	0.5197722	0.4989677	0.4581590
39.65	584.71	548.65	550.88	509.54	39.65	0.5980102	0.5665120	0.5170329	0.4834884
arameter									
a	295.43	261.77	233.44	208.30					
b	3.86	4.04	3.84	4.16					
x0	35.45	35.34	34.33	34.12					
y0	365.90	350.24	363.13	343.47					
R	1.00	1.00	1.00	1.00					
**$CP_{(SDM)}$**	30.36	30.02	29.27	28.64					

Well/ Cycle	Raw fluorescence data F6	F7	F8	F9
0.56	124.81	121.1	122.03	114.29
1.56	124.4	122.09	121.01	112.55
2.56	124.97	121.74	123.01	111.73
3.56	124.88	123.04	122.13	113.16
4.56	127.08	123.71	123.45	113.45
5.56	127.91	124.91	126.31	113.71
6.56	129.3	124.55	124.78	114.86
7.56	126.08	124.32	124.76	115.42
8.56	126.81	126.85	124.23	115.87
9.56	128.15	126.73	128.32	115.05
10.56	129.34	127.01	124.65	115.11
11.56	128.02	128.41	128.3	116.85
12.56	129.98	125.59	126.03	116.42
13.56	129.41	126.27	126.62	117.04
14.56	129.03	128.45	125.98	119.34
15.56	132.47	127.69	124.38	118.27
16.58	129.51	127.57	125.19	116.86
17.58	129.46	128.01	126.27	117.35
18.58	131.7	129.5	128.59	117.68
19.58	133.39	128.56	126.79	118.2
20.58	134.15	132.2	130.17	120.86
21.58	133.22	134.26	130.5	122.3
22.58	136.46	136.15	133.57	122.11
23.58	140.58	138.62	135.43	126.86
24.58	144.81	142.12	142.55	131.06
25.58	154.37	148.92	147.28	136.07
26.58	172.44	160.94	163.64	151.21
27.58	192.82	183.03	184.91	169.11
28.56	222.51	210.49	210.76	192.88
29.61	258.4	240.77	242.41	220.45
30.56	293.97	273.29	273.56	245.33
31.63	330.71	303.54	303.5	269.59
32.63	364.45	333.23	328.84	290.18
33.63	391.88	361.54	359.82	311.39
34.63	421.63	383.84	382.32	328.7
35.63	452.82	407.72	408.75	351.98
36.63	476.2	431.83	426.69	366.94
37.65	495.66	448.78	445.82	379.4
38.65	514.67	466.42	462.98	399.2
39.65	537.98	483.1	484.66	417.12
Parameter				
a	423.87	370.75	370.98	310.38
b	2.79	2.79	2.83	2.93
x0	32.04	32.00	32.00	31.87
y0	126.34	124.36	123.35	113.90
R	1.00	1.00	1.00	1.00
$CP_{(SDM)}$	28.37	28.33	28.27	28.01

Well/ Cycle	F6 Normalized	F7 Normalized Fluorescence	F8 Normalized Fluorescence	F9 Normalized Fluorescence
0.56	-0.0121352	-0.0262220	-0.0106636	0.0034302
1.56	-0.0153803	-0.0182614	-0.0189330	-0.0118464
2.56	-0.0108688	-0.0210757	-0.0027184	-0.0190458
3.56	-0.0115812	-0.0106223	-0.0098528	-0.0064908
4.56	0.0058317	-0.0052348	0.0008488	-0.0039447
5.56	0.0124011	0.0044146	0.0240358	-0.0016620
6.56	0.0234029	0.0015198	0.0116316	0.0084346
7.56	-0.0020832	-0.0003297	0.0114694	0.0133513
8.56	0.0036947	0.0200143	0.0071725	0.0173021
9.56	0.0143007	0.0190494	0.0403315	0.0101028
10.56	0.0237195	0.0213009	0.0105776	0.0106296
11.56	0.0132718	0.0325584	0.0401693	0.0259062
12.56	0.0287851	0.0098825	0.0217657	0.0221310
13.56	0.0242736	0.0153505	0.0265490	0.0275744
14.56	0.0212659	0.0328801	0.0213604	0.0477676
15.56	0.0484933	0.0267688	0.0083886	0.0383734
16.58	0.0250651	0.0258039	0.0149556	0.0259940
17.58	0.0246693	0.0293420	0.0237115	0.0302961
18.58	0.0423988	0.0413232	0.0425205	0.0331934
19.58	0.0557751	0.0337646	0.0279273	0.0377588
20.58	0.0617904	0.0630342	0.0553300	0.0611128
21.58	0.0544295	0.0795989	0.0580055	0.0737555
22.58	0.0800740	0.0947966	0.0828949	0.0720874
23.58	0.1126835	0.1146581	0.0979745	0.1137909
24.58	0.1461638	0.1428020	0.1556987	0.1506655
25.58	0.2218307	0.1974815	0.1940463	0.1946518
26.58	0.3648538	0.2941356	0.3266821	0.3275762
27.58	0.5261605	0.4717637	0.4991248	0.4847326
28.56	0.7611553	0.6925724	0.7086991	0.6934257
29.61	1.0452229	0.9360571	0.9652958	0.9354816
30.56	1.3267576	1.1975539	1.2178389	1.1539202
31.63	1.6175528	1.4407974	1.4605721	1.3669153
32.63	1.8846032	1.6795378	1.6660116	1.5476891
33.63	2.1017103	1.9071815	1.9171764	1.7339062
34.63	2.3371800	2.0864982	2.0995911	1.8858825
35.63	2.5840473	2.2785198	2.3138677	2.0902736
36.63	2.7690988	2.4723909	2.4593130	2.2216177
37.65	2.9231237	2.6086876	2.6144061	2.3310126
38.65	3.0735869	2.7505327	2.7535277	2.5048503
39.65	3.2580843	2.8846584	2.9292944	2.6621823

F6			F7			F8	
Well/ Cycle	FAM Normalizec Fluorescence	VIC Normalizec Fluorescence	Well Cycl	FAM Normalized Fluorescence	VIC Normalized Fluorescence	Well/ Cycle	FAM Normalized Fluorescence
0.56	-0.0252496	-0.0121352	0.6	-0.010983144	-0.026222047	0.56	-0.0164952
1.56	-0.0057360	-0.015380329	1.6	-0.012839031	-0.018261352	1.56	-0.0254727
2.56	-0.0295404	-0.010868808	2.6	-0.017721444	-0.021075739	2.56	-0.0246466
3.56	-0.0093436	-0.011581154	3.6	-0.022061366	-0.010622301	3.56	-0.0117035
4.56	-0.0158481	0.005831735	4.6	-0.017778548	-0.00523476	4.56	-0.0114832
5.56	-0.0097808	0.012401142	5.6	-0.018549455	0.004414567	5.56	-0.0011288
6.56	-0.0055720	0.023402922	6.6	0.000209287	0.001519769	6.56	-0.0024231
7.56	-0.0119126	-0.002083215	7.6	-0.014352294	-0.000329685	7.56	-0.0082337
8.56	0.0080383	0.003694698	8.6	0.005291564	0.020014313	8.56	0.0017627
9.56	0.0056879	0.01430073	9.6	0.003464229	0.01904938	9.56	0.0112359
10.56	0.0138049	0.02371952	11	0.004406449	0.02130089	10.56	0.0117316
11.56	-0.0016092	0.013271787	12	0.009231757	0.032558439	11.56	0.0106852
12.56	0.0173851	0.028785087	13	0.021966002	0.009882519	12.56	0.0243166
13.56	0.0219492	0.024273566	14	0.032358973	0.015350472	13.56	0.0121998
14.56	0.0253655	0.021265885	15	0.036756	0.032880083	14.56	0.0214251
15.56	0.0168932	0.04849331	16	0.01871106	0.026768842	15.56	0.0171016
16.58	0.0232611	0.025065061	17	0.017654632	0.02580391	16.58	0.0099967
17.58	0.0147068	0.024669313	18	0.031873587	0.029341996	17.58	0.0177350
18.58	0.0297656	0.042398799	19	0.025763434	0.041323244	18.58	0.0244268
19.58	0.0190796	0.055775063	20	0.027105383	0.033764605	19.58	0.0232977
20.58	0.0313507	0.061790425	21	0.028190364	0.063034231	20.58	0.0345334
21.58	0.0222225	0.054429522	22	0.017997257	0.07959891	21.58	0.0185611
22.58	0.0223865	0.080073957	23	0.011944208	0.0947966	22.58	0.0269052
23.58	0.0343024	0.112683548	24	0.028989823	0.114658132	23.58	0.0273734
24.58	0.0317060	0.146163782	25	0.035499706	0.142802004	24.58	0.0266299
25.58	0.0481587	0.221830696	26	0.046720689	0.197481526	25.58	0.0620167
26.58	0.0523675	0.364853827	27	0.058112985	0.294135621	26.58	0.0548842
27.58	0.0958768	0.52616049	28	0.086236822	0.471763656	27.58	0.0968251
28.56	0.1057156	0.761155329	29	0.110106394	0.69257243	28.56	0.1051968
29.61	0.1457813	1.045222853	30	0.151506967	0.936057124	29.61	0.1465869
30.56	0.1856830	1.326757594	31	0.194791979	1.197553896	30.56	0.1811200
31.63	0.2303129	1.617552824	32	0.225771029	1.440797356	31.63	0.2271366
32.63	0.2775664	1.884603208	33	0.263688243	1.679537797	32.63	0.2676455
33.63	0.3098977	2.101710262	34	0.301576905	1.907181512	33.63	0.2934490
34.63	0.3619886	2.337179999	35	0.345918346	2.086498179	34.63	0.3277342
35.63	0.3889906	2.584047262	36	0.371443941	2.278519793	35.63	0.3727044
36.63	0.4712811	2.769098772	37	0.415956694	2.472390862	36.63	0.4049242
37.65	0.5110462	2.923123682	38	0.474574195	2.608687611	37.65	0.4378326
38.65	0.5769661	3.073586865	39	0.5197722	2.750532723	38.65	0.4989677
39.65	0.5980102	3.258084329	40	0.566512019	2.884658374	39.65	0.5170329

ient derived	linear reg	iment derived	linear regre	nent derived	linear regr
aramet	value	parame	value	paramet	value
y0	0.0055	y0	0.0131	y0	0.0064
a	0.140200000	a	0.148700	a	0.1511
R	0.99375831	R	0.99908558	R	0.99267996

	F9		
VIC Normalized Fluorescence	Well/ Cycle	FAM Normalized Fluorescence	VIC Normalized Fluorescence
-0.01066356	0.56	-0.019635245	0.003430223
-0.018933028	1.56	-0.020101073	-0.011846429
-0.002718385	2.56	-0.020887158	-0.019045771
-0.009852828	3.56	-0.014365562	-0.006490821
0.000848837	4.56	-0.007349024	-0.003944713
0.024035776	5.56	-0.012968077	-0.001661994
0.011631574	6.56	-0.002195798	0.008434644
0.011469428	7.56	0.004995426	0.013351267
0.007172547	8.56	0.009013195	0.017302126
0.040331492	9.56	0.009537252	0.010102784
0.010577622	10.56	0.012244879	0.010629565
0.040169346	11.56	0.005577712	0.025906217
0.021765726	12.56	0.018067733	0.022130953
0.026549046	13.56	0.021794359	0.027574357
0.02136036	14.56	0.027355184	0.047767633
0.008388646	15.56	0.006130883	0.03837337
0.014955576	16.58	0.028257726	0.025994014
0.023711483	17.58	0.022463987	0.03029606
0.042520469	18.58	0.025491871	0.033193356
0.02792729	19.58	0.022201959	0.037758792
0.055330037	20.58	0.03128561	0.061112755
0.058005453	21.58	0.02950964	0.073755502
0.08289493	22.58	0.030761554	0.072087361
0.097974548	23.58	0.034575523	0.113790866
0.155698677	24.58	0.037923664	0.150665544
0.194046307	25.58	0.061244192	0.194651767
0.326682087	26.58	0.066251847	0.327576201
0.499124815	27.58	0.095977514	0.484732566
0.708699075	28.56	0.128556381	0.693425684
0.9652958	29.61	0.156389621	0.935481605
1.217838864	30.56	0.193364742	1.153920173
1.460572069	31.63	0.230398091	1.366915337
1.666011595	32.63	0.25392242	1.547689055
1.917176415	33.63	0.286239258	1.733906179
2.099591148	34.63	0.335733514	1.88588253
2.313867654	35.63	0.331453716	2.090273601
2.459313002	36.63	0.379084659	2.221617692
2.614406062	37.65	0.428404229	2.33101257
2.753527698	38.65	0.458159012	2.504850337
2.929294428	39.65	0.483488425	2.662182296

nent derived	linear regr
paramete	value
y0	0.0192
a	0.1522
**R**	**0.99876291**

average	nent derived	linear regr
	paramete	value
	y0	0.01105
	a	0.14805

Well/ Cycle	Raw fluorescence data G2	G3	G4	G5	Well/ Cycle	G2 Normalized Fluorescence	G3 Normalized Fluorescence	G4 Normalized Fluorescence	G5 Normalized Fluorescence
0.56	320.64	328.26	309.07	334.16	0.56	-0.0433383	-0.0208384	-0.0306293	-0.0172438
1.56	325.86	331.9	315.46	334.51	1.56	-0.0277639	-0.0099807	-0.0105876	-0.0162145
2.56	327.44	326.67	311.66	330.66	2.56	-0.0230498	-0.0255812	-0.0225059	-0.0275372
3.56	330.69	333.43	317.08	336.9	3.56	-0.0133531	-0.0054169	-0.0055066	-0.0091855
4.56	330.92	329.74	315.56	334.04	4.56	-0.0126669	-0.0164238	-0.0102739	-0.0175967
5.56	329.63	331	317.75	337.59	5.56	-0.0165157	-0.0126653	-0.0034052	-0.0071563
6.56	338.28	333.61	320.24	340.29	6.56	0.0092924	-0.0048800	0.0044045	0.0007844
7.56	337.19	334.93	322.19	343.06	7.56	0.0060403	-0.0009426	0.0105205	0.0089309
8.56	342.12	337.55	321.58	344.9	8.56	0.0207495	0.0068726	0.0086073	0.0143423
9.56	336.75	333.53	321.19	342.56	9.56	0.0047275	-0.0051186	0.0073841	0.0074604
10.56	338.05	337.92	324.35	348.58	10.56	0.0086062	0.0079762	0.0172951	0.0251650
11.56	337.89	336.6	321.47	345.15	11.56	0.0081288	0.0040388	0.0082622	0.0150775
12.56	341.68	340.95	324.26	350.38	12.56	0.0194367	0.0170144	0.0170128	0.0304588
13.56	343.6	342.75	325.41	351.37	13.56	0.0251652	0.0223836	0.0206197	0.0333704
14.56	345.39	340.44	326.47	349.81	14.56	0.0305058	0.0154931	0.0239443	0.0287824
15.56	345.39	341.94	327.6	348.86	15.56	0.0305058	0.0199674	0.0274885	0.0259885
16.58	345.92	336.56	330.13	349.1	16.58	0.0320871	0.0039195	0.0354236	0.0266943
17.58	346.35	339.86	327.84	352.99	17.58	0.0333701	0.0137630	0.0282412	0.0381347
18.58	343.8	341.82	330.04	355.56	18.58	0.0257619	0.0196095	0.0351413	0.0456930
19.58	349.21	346.94	332.2	354.01	19.58	0.0419032	0.0348818	0.0419159	0.0411345
20.58	345.73	344.54	329.7	355.49	20.58	0.0315202	0.0277229	0.0340749	0.0454872
21.58	337.13	341.14	325.15	354.52	21.58	0.0058613	0.0175811	0.0198042	0.0426344
22.58	339.13	341.81	328.19	353.53	22.58	0.0118285	0.0195797	0.0293389	0.0397229
23.58	349.68	343	328.1	354.46	23.58	0.0433055	0.0231293	0.0290567	0.0424580
24.58	343.88	341.66	327.72	354.12	24.58	0.0260006	0.0191322	0.0278648	0.0414580
25.58	352.88	344.92	332.28	354.1	25.58	0.0528530	0.0288564	0.0421669	0.0413992
26.58	349.66	346.77	334.21	358.3	26.58	0.0432458	0.0343748	0.0482201	0.0537513
27.58	356.92	348.73	336.72	366.92	27.58	0.0649067	0.0402212	0.0560925	0.0791025
28.56	363.4	354.94	337.21	364.24	28.56	0.0842405	0.0587449	0.0576294	0.0712207
29.61	370.96	358.58	345.88	371.32	29.61	0.1067965	0.0696026	0.0848221	0.0920428
30.56	374.61	370.72	352.63	379.95	30.56	0.1176866	0.1058148	0.1059928	0.1174234
31.63	378.42	371.83	352.33	382.88	31.63	0.1290542	0.1091258	0.1050519	0.1260405
32.63	392.92	385.62	364.81	393.38	32.63	0.1723164	0.1502598	0.1441943	0.1569207
33.63	398.96	391.6	369.03	398.23	33.63	0.1903373	0.1680975	0.1574300	0.1711844
34.63	416.61	406.71	376.27	409.36	34.63	0.2429979	0.2131688	0.1801376	0.2039175
35.63	415.66	411.66	380.87	418.96	35.63	0.2401634	0.2279341	0.1945651	0.2321509
36.63	429.44	428.66	393.56	432.85	36.63	0.2812775	0.2786431	0.2343662	0.2730010
37.65	444.84	438.63	400.95	442.51	37.65	0.3272249	0.3083825	0.2575442	0.3014108
38.65	457.04	454.24	407.4	455.74	38.65	0.3636248	0.3549453	0.2777741	0.3403199
39.65	462.19	468.73	417.16	459.63	39.65	0.3789904	0.3981673	0.3083855	0.3517603
Parameter					Parameter Value				
a	260.50	260.12	386.17	958.66	a	0.7772	0.78	1.21	2.819400
b	5.31	4.52	6.72	7.30	b	5.3081	4.52	6.72	7.296100
x0	39.57	39.41	46.66	53.41	x0	39.5719	39.41	46.66	53.405300
y0	335.17	335.25	318.84	340.02	y0	0.0000	0.00	0.00	0.000000
R	0.99	0.99	0.99	0.99	R	0.988974	0.994596	0.989732	0.990279
**$CP_{(SDM)}$**	32.58	33.45	37.81	43.80					

Well/	Raw fluorescence data				Well/	G2 Normalized	G3 Normalized	G4 Normalized	G5 Normalized
Cycle	G2	G3	G4	G5	Fluorescence		Fluorescence	Fluorescence	Fluorescence
0.56	110.55	115.86	118.52	116.78	0.56	-0.0405387	-0.0108232	-0.0395361	-0.0321308
1.56	113.25	114.65	119.92	117.36	1.56	-0.0171054	-0.0211538	-0.0281907	-0.0273238
2.56	112.68	114.56	122.67	117.83	2.56	-0.0220524	-0.0219222	-0.0059052	-0.0234284
3.56	114.12	115.68	122.04	117.65	3.56	-0.0095547	-0.0123600	-0.0110107	-0.0249203
4.56	114.91	114.33	122.78	119.03	4.56	-0.0026983	-0.0238859	-0.0050138	-0.0134829
5.56	114.06	116.9	123.32	120.79	5.56	-0.0100754	-0.0019440	-0.0006378	0.0011040
6.56	114.33	117.92	124.66	120.91	6.56	-0.0077321	0.0067644	0.0102213	0.0020985
7.56	116.65	120.02	124.18	121.29	7.56	0.0124031	0.0246936	0.0063315	0.0052479
8.56	118.39	120.77	124.93	122.66	8.56	0.0275046	0.0310968	0.0124094	0.0166025
9.56	117.73	120.47	123.96	121.89	9.56	0.0217764	0.0285355	0.0045487	0.0102207
10.56	118.94	120.16	123.61	122.18	10.56	0.0322780	0.0258888	0.0017123	0.0126242
11.56	119.17	121.54	126.15	123.55	11.56	0.0342742	0.0376708	0.0222960	0.0239788
12.56	119.41	119.51	125.31	123.44	12.56	0.0363571	0.0203393	0.0154888	0.0230671
13.56	119.56	121.1	126.68	124.01	13.56	0.0376590	0.0339143	0.0265910	0.0277912
14.56	118.72	119.92	127.46	126.35	14.56	0.0303686	0.0238398	0.0329120	0.0471851
15.56	119.32	119.34	127.59	126.03	15.56	0.0355760	0.0188879	0.0339655	0.0445329
16.58	118.84	120.39	127.55	126.66	16.58	0.0314101	0.0278525	0.0336414	0.0497543
17.58	120.35	120.64	129.24	125.91	17.58	0.0445154	0.0299869	0.0473368	0.0435384
18.58	119.31	121.32	127.85	125.93	18.58	0.0354892	0.0357926	0.0360725	0.0437041
19.58	121.94	122.63	128.25	127.54	19.58	0.0583149	0.0469769	0.0393140	0.0570478
20.58	124.43	124.08	128.72	126.87	20.58	0.0799256	0.0593566	0.0431228	0.0514948
21.58	123.65	124.6	130.36	130.23	21.58	0.0731560	0.0637962	0.0564131	0.0793424
22.58	128.14	127.99	130.68	132.78	22.58	0.1121246	0.0927390	0.0590063	0.1004767
23.58	131.32	129.64	135.98	134.84	23.58	0.1397238	0.1068261	0.1019565	0.1175499
24.58	138.12	139.81	139.81	143.11	24.58	0.1987409	0.1936544	0.1329941	0.1860915
25.58	149.86	147.81	149.13	150.4	25.58	0.3006321	0.2619560	0.2085216	0.2465108
26.58	171.92	172.48	164.36	170.48	26.58	0.4920904	0.4725808	0.3319427	0.4129332
27.58	200.15	191.78	180.89	189.67	27.58	0.7370980	0.6373582	0.4658987	0.5719794
28.56	237.1	231.86	210.09	220.45	28.56	1.0577864	0.9795488	0.7025301	0.8270831
29.61	278.69	271.38	243.59	259.84	29.61	1.4187452	1.3169583	0.9740078	1.1535463
30.56	319.6	310.57	275.04	295.92	30.56	1.7738023	1.6515504	1.2288728	1.4525762
31.63	354.59	357.1	307.02	327.47	31.63	2.0774799	2.0488091	1.4880327	1.7140617
32.63	385.95	387.51	335.7	358.1	32.63	2.3496527	2.3084403	1.7204501	1.9679222
33.63	415.5	419.85	363.38	397.5	33.63	2.6061166	2.5845492	1.9447636	2.2944683
34.63	440.02	445.8	386.05	422.2	34.63	2.8189252	2.8061022	2.1284770	2.4991811
35.63	467.09	478.85	412.06	449.76	35.63	3.0538652	3.0882729	2.3392572	2.7275976
36.63	489.25	491.86	426.52	470.1	36.63	3.2461914	3.1993482	2.4564384	2.8961749
37.65	499.4	513.25	444.29	487.51	37.65	3.3342831	3.3819694	2.6004431	3.0404685
38.65	516.02	528.61	456.62	505.98	38.65	3.4785278	3.5131083	2.7003631	3.1935473
39.65	533.99	543.96	469.92	523.34	39.65	3.6344891	3.6441619	2.8081438	3.3374265
Parameter									
a	418.51	429.73	352.9406	413.1889					
b	2.59	2.54	2.607	2.725					
x0	31.01	31.27	31.5584	31.7149					
y0	115.22	117.13	123.3987	120.66					
R	1.00	0.999380	0.999430	0.999409					
$CP_{(SDM)}$	27.60	27.92	28.12	28.13					

G3				G4			
Well/ Cycle	**FAM** Normalized Fluorescence	VIC Normalized Fluorescence	**FSM predicted Fluorescence**	Well/ Cycle	**FAM** Normalized Fluorescence	VIC Normalized Fluorescence	Well/ Cycle
0.56	-0.020838429	-0.01082323	2.08144E-05	0.56	-0.0306293	-0.039536073	0.56
1.56	-0.009980731	-0.021153835	3.06849E-05	1.56	-0.0105876	-0.028190735	1.56
2.56	-0.025581215	-0.021922227	4.53216E-05	2.56	-0.0225059	-0.005905249	2.56
3.56	-0.005416918	-0.012360014	6.7026E-05	3.56	-0.0055066	-0.011010651	3.56
4.56	-0.01642376	-0.023885895	9.92107E-05	4.56	-0.0102739	-0.005013829	4.56
5.56	-0.012665326	-0.001944032	0.000146936	5.56	-0.0034052	-0.00063777	5.56
6.56	-0.004879999	0.006764412	0.000217705	6.56	0.0044045	0.010221339	6.56
7.56	-0.000942591	0.024693561	0.000322642	7.56	0.0105205	0.006331509	7.56
8.56	0.006872565	0.031096829	0.000478242	8.56	0.0086073	0.012409369	8.56
9.56	-0.005118629	0.028535521	0.000708954	9.56	0.0073841	0.00454867	9.56
10.56	0.007976232	0.025888838	0.00105102	10.56	0.0172951	0.001712336	10.56
11.56	0.004038825	0.03767085	0.001558149	11.56	0.0082622	0.022296021	11.56
12.56	0.017014372	0.020339339	0.002309907	12.56	0.0170128	0.015488818	12.56
13.56	0.022383563	0.033914266	0.003424111	13.56	0.0206197	0.026591042	13.56
14.56	0.015493101	0.023839792	0.005075105	14.56	0.0239443	0.032912016	14.56
15.56	0.019967427	0.018887932	0.007520607	15.56	0.0274885	0.033965512	15.56
16.58	0.00391951	0.027852506	0.011228872	16.58	0.0354236	0.033641359	16.58
17.58	0.013763028	0.029986929	0.016626404	17.58	0.0282412	0.047336803	17.58
18.58	0.019609481	0.035792558	0.024601077	18.58	0.0351413	0.036072503	18.58
19.58	0.034881848	0.046976932	0.036362717	19.58	0.0419159	0.039314028	19.58
20.58	0.027722926	0.059356583	0.053664813	20.58	0.0340749	0.043122821	20.58
21.58	0.01758112	0.063796181	0.079020578	21.58	0.0198042	0.056413074	21.58
22.58	0.019579652	0.092738951	0.115972306	22.58	0.0293389	0.059006294	22.58
23.58	0.023129284	0.106826139	0.169388208	23.58	0.0290567	0.101956504	23.58
24.58	0.019132219	0.193654447	0.245705671	24.58	0.0278648	0.132994108	24.58
25.58	0.028856422	0.261955968	0.35294001	25.58	0.0421669	0.208521646	25.58
26.58	0.034374758	0.472580781	0.500137397	26.58	0.0482201	0.331942719	26.58
27.58	0.040221211	0.6373582	0.695842481	27.58	0.0560925	0.465898749	27.58
28.56	0.058744922	0.979548817	0.939763667	28.56	0.0576294	0.702530091	28.56
29.61	0.06960262	1.316958328	1.256392491	29.61	0.0848221	0.97400783	29.61
30.56	0.105814834	1.651550402	1.58097261	30.56	0.1059928	1.228872751	30.56
31.63	0.109125836	2.04880912	1.965788881	31.63	0.1050519	1.488032694	31.63
32.63	0.150259809	2.308440275	2.315858486	32.63	0.1441943	1.720450053	32.63
33.63	0.168097457	2.584549172	2.631928566	33.63	0.1574300	1.9447636	33.63
34.63	0.213168837	2.806102229	2.898718312	34.63	0.1801376	2.128477042	34.63
35.63	0.227934114	3.088272885	3.111406587	35.63	0.1945651	2.339257221	35.63
36.63	0.278643146	3.199348233	3.273372924	36.63	0.2343662	2.456438358	36.63
37.65	0.308382501	3.381969423	3.394470095	37.65	0.2575442	2.600443116	37.65
38.65	0.354945324	3.513108342	3.479209696	38.65	0.2777741	2.700363132	38.65
39.65	0.398167316	3.644161885	3.538784173	39.65	0.3083855	2.808143846	39.65

**ment derived**	linear regre
**paramete**	**value**
y0	-0.0088
a	0.0647
**R**	**0.9580485**

**ment derived**	linear regre
**paramete**	**value**
y0	0.0071
a	0.0756
**R**	0.95955011

**xperiment d**
**parameter**
y0
a
**R**

average

G5

FAM Normalized Fluorescence	VIC Normalized Fluorescence
-0.017243818	-0.032130804
-0.016214477	-0.027323781
-0.027537231	-0.023428435
-0.009185547	-0.02492027
-0.017596735	-0.01348287
-0.007156274	0.001103958
0.000784358	0.002098514
0.008930859	0.005247943
0.014342252	0.016602463
0.007460371	0.010220725
0.02516504	0.012624237
0.015077496	0.023978756
0.030458795	0.02306708
0.03337036	0.027791223
0.028782439	0.047185074
0.025988513	0.044532923
0.026694347	0.049754345
0.03813474	0.043538367
0.045693045	0.043704126
0.041134534	0.057047759
0.045487177	0.051494818
0.042634431	0.079342399
0.039722866	0.100476724
0.042457973	0.117549943
0.041458041	0.186091459
0.041399222	0.246510764
0.053751316	0.412933212
0.07910252	0.571979366
0.071220708	0.827083099
0.09204281	1.153546257
0.117423424	1.452576233
0.12604048	1.714061702
0.156920717	1.967922239
0.171184445	2.294468277
0.203917496	2.499181149
0.232150856	2.727597616
0.273000997	2.896174936
0.301410815	3.040468502
0.340319913	3.193547318
0.351760306	3.337426486

lerived linear regressio
value
0.0102
0.0721
**0.989111**

**Experiment derived** linear regression	
**parameter**	**value**
y0	0.0065
a	0.06815
**R**	

Well/ Cycle	Raw fluorescence data C6 sample	C7 sample	C8 sample	C9 sample
0.56	147.96	139.51	131.34	127.01
1.56	147.05	142.86	128.95	125.66
2.56	146.77	141.24	131.9	125.73
3.56	147.58	144.75	130.33	125.98
4.56	148.69	143.27	131.25	127.28
5.56	152.21	145.64	132.85	127.71
6.56	150.25	142.94	135.11	128.19
7.56	153.87	144.46	133.64	129.62
8.56	152.48	147.52	136.78	127.7
9.56	152.48	145.83	132.69	127.12
10.56	152.33	144.5	136.39	131.28
11.56	155.19	147.22	137.58	130.23
12.56	153.53	148.16	135.25	130.81
13.56	153.78	146.73	137.89	131.67
14.56	155.37	149.42	135.63	131.24
15.56	154.13	147.36	135.92	131.5
16.58	156.04	150.33	136.81	131.84
17.58	157.47	152.43	137.26	130.38
18.58	157.24	151.65	139.27	130.87
19.58	156.87	151.25	137.47	131.14
20.58	155.03	154.03	136.87	131.04
21.58	160.05	152.08	140.03	132.36
22.58	163.51	155.46	140.48	133.32
23.58	163.45	155.98	143.38	136.71
24.58	166.07	160.02	145.37	137.41
25.58	166.83	161.66	147.17	140.2
26.58	174.19	164.99	149.77	144.15
27.58	181.74	171.91	156.55	146.48
28.56	191.42	183.49	166.28	155.78
29.61	205.5	193.83	180.05	165.26
30.56	221.28	209.49	188.95	178.31
31.63	241.39	222.85	203.35	188.62
32.63	258.02	238.25	215.32	198.17
33.63	278.22	251.76	232.64	211.08
34.63	293.39	267.08	246.49	221.4
35.63	320.68	288.91	258.52	235.25
36.63	335.5	306.52	276.29	248.03
37.65	359.88	319.48	289.53	260.09
38.65	380.84	338.23	308.46	276.86
39.65	401.27	355.96	322.95	287.84
a	321.03	274.33	239.42	202.70
b	3.62	3.77	3.64	3.66
x0	35.32	35.33	35.10	35.10
y0	151.41	145.26	133.66	128.19
R	1.00	1.00	1.00	1.00
$CP_{(SDM)}$	30.56	30.36	30.31	30.29

Well/ Cycle	C6Normalized Fluorescence	C7Normalized Fluorescence	C8Normalized Fluorescence	C9 Normalized Fluorescence
0.56	-0.0227826	-0.0395954	-0.0173641	-0.0091695
1.56	-0.0287928	-0.0165336	-0.0352452	-0.0197012
2.56	-0.0306421	-0.0276859	-0.0131744	-0.0191551
3.56	-0.0252923	-0.0035226	-0.0249205	-0.0172048
4.56	-0.0179612	-0.0137111	-0.0180374	-0.0070632
5.56	0.0052870	0.0026043	-0.0060668	-0.0037087
6.56	-0.0076580	-0.0159829	0.0108416	0.0000359
7.56	0.0162506	-0.0055190	-0.0001564	0.0111916
8.56	0.0070702	0.0155464	0.0233359	-0.0037867
9.56	0.0070702	0.0039122	-0.0072639	-0.0083114
10.56	0.0060795	-0.0052436	0.0204181	0.0241416
11.56	0.0249687	0.0134812	0.0293212	0.0159503
12.56	0.0140051	0.0199523	0.0118890	0.0204750
13.56	0.0156562	0.0101080	0.0316405	0.0271841
14.56	0.0261575	0.0286263	0.0147321	0.0238295
15.56	0.0179678	0.0144450	0.0169017	0.0258579
16.58	0.0305826	0.0348908	0.0235604	0.0285103
17.58	0.0400272	0.0493475	0.0269271	0.0171205
18.58	0.0385082	0.0439779	0.0419652	0.0209431
19.58	0.0360644	0.0412242	0.0284982	0.0230494
20.58	0.0239120	0.0603621	0.0240093	0.0222693
21.58	0.0570671	0.0469380	0.0476512	0.0325669
22.58	0.0799190	0.0702064	0.0510179	0.0400560
23.58	0.0795228	0.0737861	0.0727146	0.0665021
24.58	0.0968268	0.1015980	0.0876030	0.0719630
25.58	0.1018463	0.1128880	0.1010699	0.0937283
26.58	0.1504562	0.1358121	0.1205222	0.1245430
27.58	0.2003210	0.1834503	0.1712475	0.1427198
28.56	0.2642536	0.2631685	0.2440437	0.2152710
29.61	0.3572464	0.3343503	0.3470656	0.2892264
30.56	0.4614671	0.4421558	0.4136520	0.3910321
31.63	0.5942857	0.5341277	0.5213873	0.4714624
32.63	0.7041203	0.6401433	0.6109423	0.5459639
33.63	0.8375333	0.7331478	0.7405240	0.6466774
34.63	0.9377252	0.8386127	0.8441444	0.7271858
35.63	1.1179649	0.9888931	0.9341483	0.8352324
36.63	1.2158451	1.1101226	1.0670967	0.9349317
37.65	1.3768654	1.1993409	1.1661533	1.0290142
38.65	1.5152979	1.3284183	1.3077804	1.1598404
39.65	1.6502300	1.4504739	1.4161890	1.2454975

Well/ Cycle	C2	C3	C4	C5
0.56	138.68	137.43	146.68	138.65
1.56	138.01	138.33	147.15	138.77
2.56	142.8	139.7	149.24	140.69
3.56	141.3	140.17	148.16	139.25
4.56	142.57	142.24	149.03	140.38
5.56	142.4	141.37	150.8	140.04
6.56	144.08	141.85	150.68	141.05
7.56	145.4	141.72	151.9	140.82
8.56	145.18	142.46	150.1	142.56
9.56	146.61	143.55	153.72	142.91
10.56	146.06	143.01	151.08	141.69
11.56	147.43	144.98	151.98	143.88
12.56	148.04	143.89	152.04	143.35
13.56	147.73	144.41	150.82	141.93
14.56	146.43	144.07	152.91	144.81
15.56	149.78	148.34	152.03	144.93
16.58	151.77	146.29	152.39	146.16
17.58	149.12	147.88	151.51	146.08
18.58	147.92	146.71	153	145.93
19.58	150.02	148.26	153.94	146.82
20.58	149.02	149.04	154.3	146.83
21.58	154.16	148.15	154.16	148.8
22.58	153.62	149.39	158.15	150.27
23.58	155.06	152.53	155.6	150.51
24.58	156.89	154.94	158.71	152.74
25.58	155.65	154.11	159.27	154.05
26.58	158.74	156.04	164.21	156.11
27.58	162.55	161.98	168.21	161.2
28.56	166.51	166.14	173.93	165.33
29.61	170.92	172.92	178.45	172.73
30.56	176.89	180.88	183.27	181.66
31.63	183.68	186.79	194.63	190.23
32.63	189.38	198.34	200.35	198.22
33.63	192.48	207.17	213	207.95
34.63	199.48	214.46	221.03	213.82
35.63	207.7	229.88	235.3	226.15
36.63	215.67	238.74	246.97	236.26
37.65	217.67	246.29	259.05	248.96
38.65	229.03	262.18	271.35	257.14
39.65	236.92	273.44	286.27	269
Parameter				
a	189.98	210.04	213.82	187.87
b	5.9579	4.5038	4.1506	4.3622
x0	39.832	37.4031	37.43	36.468
y0	143.33	142.187	150.18	141.47
R	0.9964	0.99846	0.9992	0.999
**$CP_{(SDM)}$**	31.99	31.47	31.96	30.72

Well/ Cycle	Fluorescence Data Normalized To Y0 C2	C3	C4	C5
0.56	-0.03	-0.03	-0.02	-0.02
1.56	-0.04	-0.03	-0.02	-0.02
2.56	0.00	-0.02	-0.01	-0.01
3.56	-0.01	-0.01	-0.01	-0.02
4.56	-0.01	0.00	-0.01	-0.01
5.56	-0.01	-0.01	0.00	-0.01
6.56	0.01	0.00	0.00	0.00
7.56	0.01	0.00	0.01	0.00
8.56	0.01	0.00	0.00	0.01
9.56	0.02	0.01	0.02	0.01
10.56	0.02	0.01	0.01	0.00
11.56	0.03	0.02	0.01	0.02
12.56	0.03	0.01	0.01	0.01
13.56	0.03	0.02	0.00	0.00
14.56	0.02	0.01	0.02	0.02
15.56	0.04	0.04	0.01	0.02
16.58	0.06	0.03	0.01	0.03
17.58	0.04	0.04	0.01	0.03
18.58	0.03	0.03	0.02	0.03
19.58	0.05	0.04	0.03	0.04
20.58	0.04	0.05	0.03	0.04
21.58	0.08	0.04	0.03	0.05
22.58	0.07	0.05	0.05	0.06
23.58	0.08	0.07	0.04	0.06
24.58	0.09	0.09	0.06	0.08
25.58	0.09	0.08	0.06	0.09
26.58	0.11	0.10	0.09	0.10
27.58	0.13	0.14	0.12	0.14
28.56	0.16	0.17	0.16	0.17
29.61	0.19	0.22	0.19	0.22
30.56	0.23	0.27	0.22	0.28
31.63	0.28	0.31	0.30	0.34
32.63	0.32	0.39	0.33	0.40
33.63	0.34	0.46	0.42	0.47
34.63	0.39	0.51	0.47	0.51
35.63	0.45	0.62	0.57	0.60
36.63	0.50	0.68	0.64	0.67
37.65	0.52	0.73	0.72	0.76
38.65	0.60	0.84	0.81	0.82
39.65	0.65	0.92	0.91	0.90

C2			C3			
Well/ Cycle	**FAM** Normalized Fluorescence	VIC Normalized Fluorescence	Well/ Cycle	**FAM** Normalized Fluorescence	VIC Normalized Fluorescence	Well/ Cycle
0.56	-0.032074496	-0.032451391	0.56	-0.041460569	-0.033453223	0.56
1.56	-0.033367822	-0.037125876	1.56	-0.019622025	-0.027123512	1.56
2.56	-0.034609415	-0.003706797	2.56	-0.025221652	-0.017488287	2.56
3.56	-0.012648733	-0.014172062	3.56	-0.005412972	-0.014182771	3.56
4.56	-0.019917227	-0.005311471	4.56	-0.012482501	0.000375563	4.56
5.56	-0.01624418	-0.006497534	5.56	-0.015538964	-0.005743157	5.56
6.56	0.005354371	0.005223562	6.56	0.01129258	-0.002367312	6.56
7.56	-0.010294878	0.014432995	7.56	-0.005482968	-0.003281603	7.56
8.56	0.01929643	0.01289809	8.56	0.016915539	0.001922825	8.56
9.56	0.010320745	0.022874976	9.56	0.000349977	0.009588808	9.56
10.56	0.032514227	0.019037712	10.56	0.016612226	0.005790982	10.56
11.56	0.010320745	0.028595987	11.56	0.012435838	0.019646014	11.56
12.56	0.037713399	0.032851861	12.56	0.026341577	0.011980032	12.56
13.56	0.016606311	0.03068904	13.56	0.034507699	0.015637198	13.56
14.56	0.028220383	0.021619144	14.56	0.025501633	0.013245974	14.56
15.56	0.039006725	0.044991568	15.56	0.02769482	0.043276933	15.56
16.58	0.030108639	0.058875486	16.58	0.031637891	0.02885926	16.58
17.58	0.039161924	0.040386852	17.58	0.050489967	0.040041748	17.58
18.58	0.043688567	0.03201464	18.58	0.040597294	0.031813124	18.58
19.58	0.047982411	0.046666011	19.58	0.03989734	0.042714292	19.58
20.58	0.038929126	0.039689168	20.58	0.043000467	0.048200041	20.58
21.58	0.032643559	0.075550142	21.58	0.03320112	0.041940661	21.58
22.58	0.055225039	0.071782646	22.58	0.042067196	0.050661595	22.58
23.58	0.059958614	0.0818293	23.58	0.062459169	0.072745252	23.58
24.58	0.069529229	0.094596923	24.58	0.090713952	0.089694809	24.58
25.58	0.122038282	0.085945638	25.58	0.128301447	0.08385741	25.58
26.58	0.152431454	0.107504083	26.58	0.166938871	0.097431122	26.58
27.58	0.246844283	0.134085856	27.58	0.294423705	0.139207211	27.58
28.56	0.305768236	0.161714155	28.56	0.354363042	0.168464539	28.56
29.61	0.411433006	0.192482033	29.61	0.491413906	0.216148357	29.61
30.56	0.525866529	0.234133787	30.56	0.645496967	0.272131129	30.56
31.63	0.620331092	0.281506552	31.63	0.757559496	0.313696227	31.63
32.63	0.718313502	0.321274558	32.63	0.924311713	0.394927511	32.63
33.63	0.798473875	0.342902771	33.63	1.017778815	0.457029003	33.63
34.63	0.902172788	0.391740674	34.63	1.155226318	0.508299657	34.63
35.63	0.970098293	0.449090324	35.63	1.258819412	0.616748695	35.63
36.63	1.092395241	0.504695764	36.63	1.418758749	0.679061177	36.63
37.65	1.165804449	0.518649451	37.65	1.525758283	0.732160415	37.65
38.65	1.274728401	0.597906389	38.65	1.642417172	0.843914968	38.65
39.65	1.327651319	0.652953681	39.65	1.721721885	0.923106678	39.65

**Experiment derived**	linear regression	**periment derived**	linear regress	**periment der**
**parameter**	**value**	**parameter**	**value**	**parameter**
y0	-0.1071	y0	-0.0788	y0
a	2.6077	a	2.6058	a
**R**	**0.99771781**	**R**	**0.9985567**	**R**

C4			C5	
**FAM** Normalized Fluorescence	VIC Normalized Fluorescence	Well/ Cycle	**FAM** Normalized Fluorescence	VIC Normalized Fluorescence
-0.023954295	-0.023315772	0.56	-0.018345399	-0.019910693
-0.004570496	-0.020186228	1.56	-0.002648123	-0.019062437
-0.022097531	-0.006269743	2.56	-0.027050465	-0.005490338
-0.009324628	-0.013461037	3.56	-0.004314053	-0.015669412
-0.001305856	-0.00766805	4.56	-0.019659371	-0.007681666
-0.004856152	0.004117682	5.56	-0.006848142	-0.010085059
0.013099367	0.003318649	6.56	0.000683735	-0.00294557
0.005529484	0.011442147	7.56	-0.008115187	-0.004571394
0.017914711	-0.000543342	8.56	0.002842404	0.007728321
0.005325444	0.023560809	9.56	0.0036871	0.010202401
0.022791267	0.005982091	10.56	0.016639112	0.001578463
0.002611712	0.011974836	11.56	0.009154163	0.017059138
0.026361967	0.012374352	12.56	0.027925196	0.013312674
0.034911243	0.004250854	13.56	0.021660364	0.003274976
0.026361967	0.018167339	14.56	0.028981066	0.023633124
0.024382779	0.012307766	15.56	0.034049245	0.02448138
0.027076107	0.014704864	16.58	0.036630262	0.033176005
0.030299939	0.008845291	17.58	0.037779988	0.032610501
0.030932463	0.018766613	18.58	0.048761041	0.031550181
0.03770659	0.025025702	19.58	0.055401294	0.037841414
0.034645991	0.0274228	20.58	0.056785658	0.037912102
0.018077943	0.026490595	21.58	0.027221282	0.051837641
0.031014079	0.053058431	22.58	0.043223587	0.062228779
0.065211181	0.036078987	23.58	0.064904129	0.063925291
0.06208937	0.05678725	24.58	0.088250601	0.079688718
0.117384207	0.060516069	25.58	0.124713331	0.088948848
0.178086105	0.093409579	26.58	0.176779482	0.103510579
0.269700061	0.120044	27.58	0.293277202	0.139490778
0.363007549	0.158131222	28.56	0.344569048	0.168684927
0.509018568	0.188228118	29.61	0.486384197	0.220994057
0.660395838	0.220322596	30.56	0.622380326	0.284118453
0.79775556	0.295964352	31.63	0.738924974	0.344698081
0.951805754	0.334051575	32.63	0.888623957	0.401177804
1.056967966	0.418282932	33.63	0.994750681	0.469957241
1.222709651	0.471751533	34.63	1.141610545	0.511451105
1.30948786	0.566769831	35.63	1.222443303	0.598609425
1.493389104	0.644475755	36.63	1.394339032	0.670075007
1.635074475	0.724911707	37.65	1.48817073	0.759848784
1.766721077	0.806812552	38.65	1.6580955	0.817671579
1.834584779	0.906158944	39.65	1.734775168	0.901507563

rived linear regress
**value**
-0.0929
3.1214
**0.9985567**

**periment derived**	linear regress:
**parameter**	**value**
y0	-0.0602
a	2.3734
**R**	**0.9978818**

AVERAGE	
parameter	value
y0	-0.08475
a	2.677075

Well/	Raw fluorescence data				Well/	C6Normalized	C7Normalized	C8Normalized	C9 Normalized
Cycle	6 sampl	C7 sample	C8 sample	C9 sample	Cycle	Fluorescence	Fluorescence	Fluorescence	Fluorescence
0.56	444.3	423.16	413.83	381.41	0.56	-0.0256453	-0.0211554	-0.0274170	0.0019716
1.56	451.65	426.14	417.83	379.73	1.56	-0.0095267	-0.0142621	-0.0180162	-0.0024418
2.56	443.32	419.58	417.27	380.07	2.56	-0.0277944	-0.0294366	-0.0193323	-0.0015486
3.56	451.49	425.16	422.5	379.46	3.56	-0.0098775	-0.0165290	-0.0070407	-0.0031511
4.56	448.2	426.9	422.79	381.9	4.56	-0.0170925	-0.0125041	-0.0063592	0.0032588
5.56	448.46	430.08	419.73	381.1	5.56	-0.0165224	-0.0051482	-0.0135508	0.0011572
6.56	458.6	433.96	426.84	384.02	6.56	0.0057148	0.0038269	0.0031591	0.0088281
7.56	453.72	430.95	425.42	380.78	7.56	-0.0049871	-0.0031357	-0.0001781	0.0003166
8.56	461.68	442	431.54	386.35	8.56	0.0124692	0.0224249	0.0142051	0.0149491
9.56	460.21	434.79	429.8	384.7	9.56	0.0092455	0.0057469	0.0101157	0.0106145
10.56	464.49	442.48	433.44	388.92	10.56	0.0186316	0.0235352	0.0186705	0.0217005
11.56	461.69	440.39	433.01	383.36	11.56	0.0124912	0.0187007	0.0176599	0.0070943
12.56	466.37	441.57	440.85	385.84	12.56	0.0227545	0.0214302	0.0360854	0.0136093
13.56	467.91	444.2	434.85	386.08	13.56	0.0261317	0.0275139	0.0219842	0.0142398
14.56	471.68	449.07	437.91	389.92	14.56	0.0343993	0.0387790	0.0291758	0.0243275
15.56	469.35	444.58	436.06	383.12	15.56	0.0292896	0.0283929	0.0248280	0.0064638
16.58	466.14	441.28	437.96	387.29	16.58	0.0222501	0.0207594	0.0292934	0.0174185
17.58	469.86	445.42	441.13	391.1	17.58	0.0304081	0.0303359	0.0367435	0.0274274
18.58	473.39	448.77	438.77	390.68	18.58	0.0381494	0.0380851	0.0311970	0.0263241
19.58	475.08	448.96	441.36	387.03	19.58	0.0418556	0.0385246	0.0372840	0.0167354
20.58	477.65	446.65	435.01	389.12	20.58	0.0474916	0.0331812	0.0223603	0.0222259
21.58	467.35	442.81	438.8	382.62	21.58	0.0249036	0.0242986	0.0312675	0.0051503
22.58	477.01	446.27	439.45	388.05	22.58	0.0460881	0.0323021	0.0327952	0.0194150
23.58	486.47	462.82	447.22	395.87	23.58	0.0668340	0.0705853	0.0510562	0.0399583
24.58	490.71	459.59	452.9	400.01	24.58	0.0761323	0.0631137	0.0644053	0.0508341
25.58	512.51	487.99	472.52	409.58	25.58	0.1239400	0.1288080	0.1105162	0.0759747
26.58	533.69	504.46	486.22	430.72	26.58	0.1703880	0.1669060	0.1427140	0.1315099
27.58	583.6	547.96	533.95	468.26	27.58	0.2798411	0.2675293	0.2548890	0.2301282
28.56	603.79	578.94	567.53	497.53	28.56	0.3241180	0.3391915	0.3338087	0.3070211
29.61	671.55	638.66	621.07	540.77	29.61	0.4727164	0.4773346	0.4596384	0.4206134
30.56	743.33	706.34	680.69	586.52	30.56	0.6301307	0.6338905	0.5997573	0.5407996
31.63	790.46	756.9	726.84	627.36	31.63	0.7334873	0.7508448	0.7082190	0.6480871
32.63	865.75	809.34	789.9	670.84	32.63	0.8985991	0.8721479	0.8564226	0.7623099
33.63	912.84	858.88	829.06	699.92	33.63	1.0018680	0.9867427	0.9484564	0.8387036
34.63	979.2	921.91	884.29	748.12	34.63	1.1473962	1.1325423	1.0782579	0.9653260
35.63	1018.5	956.8	909.09	774.43	35.63	1.2336473	1.2132491	1.1365428	1.0344429
36.63	1089.3	1021.24	980.53	823.33	36.63	1.3887370	1.3623104	1.3044411	1.1629041
37.65	1136.9	1070.81	1010.43	856.48	37.65	1.4931682	1.4769746	1.3747120	1.2499898
38.65	1203.5	1123.2	1065.57	896.88	38.65	1.6393763	1.5981620	1.5043020	1.3561214
39.65	1229.6	1152.65	1106.66	921.27	39.65	1.6965700	1.6662851	1.6008717	1.4201944
a	######	794.70	734.29	581.07					
b	3.16	3.14	3.11	3.03					
x0	33.20	33.08	32.97	32.85					
y0	######	432.31	425.50	380.66					
R	1.00	1.00	1.00	1.00					
**CP$_{(SDM)}$**	29.04	28.94	28.88	28.86					
