# Supplementary material for: Quantify single nucleotide polymorphism (SNP) ratio in pooled DNA based on normalized fluorescence real-time PCR
Source: BMC Genomics. 2006 Jun 9;7:143. doi: 10.1186/1471-2164-7-143 (PMC1552069; doi:10.1186/1471-2164-7-143)
Supplement: Additional file 4 — Contained the raw and analytical datas used during the procession. provide baseline-subtracted fluorescence ratios. [file 1471-2164-7-143-S4.pdf]

Well/	FAM baseline subtracted fluorescence																									
Cycle	B5	B6	B7	B8	B9	C3	C4	C5	C6	C7	C8	C9	D2	D3	D4	D5	D6	D7	D8	D9	E2	E3	E4	E5	E6	E7
0.58	-9.33	-5.94	-9.24	-9.73	-12.1	-4.38	-3.79	-1.9	-5.12	-1.17	-8.88	-9.7	-4.66	-7.91	-4.8	-5.19	-6.16	-13.3	-10.2	-7.4	-9.17	-6.64	-3.93	-6.33	-4.37	-7.32
1.7	-5.62	-4.85	-6.42	-9.48	-8.08	-4.03	-2.69	-2.57	-2.84	-3.19	-5.57	-5.39	-3.45	-5.78	-3.99	-4.65	-3.84	-6.64	-5.61	-5.45	-4.4	-4.4	-3.43	-2.72	-1.98	-2.8
2.7	-1.92	-1.87	-3.24	-5.74	-3.24	-1.52	0.8	-0.88	0.02	-1.99	-2.39	-2.13	-1.79	-2.68	-2.49	-2.66	-1.32	-2.3	-2.13	-3.04	-1.62	-2.49	-2.21	-0.93	-0.82	-0.52
3.7	0.72	0.69	-0.74	-1.84	0.56	0.82	3.72	0.9	2.05	-0.24	-0.1	0.01	-0.36	-0.04	-1.03	-0.65	0.62	0.38	0.19	-0.94	-0.04	-1.05	-0.97	-0.03	-0.21	0.58
4.7	1.93	1.99	0.68	0.86	2.53	2.1	4.96	1.9	2.78	1.01	1.01	1	0.55	1.52	0.05	0.77	1.63	1.63	1.32	0.48	0.63	-0.13	-0.02	0.32	0.06	0.92
5.7	1.87	1.99	1.1	2.04	2.76	2.18	4.45	1.94	2.3	1.44	1.09	1.06	0.92	1.96	0.64	1.42	1.78	1.81	1.5	1.17	0.69	0.31	0.54	0.35	0.18	0.8
6.7	1	1.08	0.85	1.95	1.75	1.37	2.71	1.22	1.05	1.14	0.51	0.56	0.85	1.54	0.79	1.41	1.31	1.31	1.07	1.25	0.45	0.4	0.74	0.23	0.22	0.47
7.7	-0.16	-0.19	0.32	1.11	0.17	0.14	0.42	0.12	-0.47	0.39	-0.33	-0.17	0.54	0.63	0.64	0.96	0.54	0.5	0.41	0.94	0.11	0.31	0.69	0.08	0.25	0.09
8.7	-1.17	-1.32	-0.17	0.06	-1.37	-1.05	-1.71	-0.98	-1.79	-0.44	-1.06	-0.81	0.16	-0.37	0.33	0.34	-0.24	-0.31	-0.2	0.45	-0.15	0.15	0.51	-0.05	0.3	-0.23
9.7	-1.71	-1.94	-0.37	-0.75	-2.43	-1.85	-3.19	-1.77	-2.59	-1.09	-1.43	-1.18	-0.14	-1.17	0	-0.24	-0.8	-0.93	-0.56	-0.04	-0.25	0.04	0.29	-0.11	0.35	-0.44
10.7	-1.66	-1.87	-0.2	-1.06	-2.75	-2.05	-3.72	-2.06	-2.7	-1.36	-1.33	-1.17	-0.29	-1.57	-0.24	-0.62	-1.02	-1.25	-0.59	-0.38	-0.16	0.02	0.13	-0.12	0.4	-0.53
11.7	-1.04	-1.14	0.3	-0.79	-2.3	-1.65	-3.27	-1.83	-2.15	-1.19	-0.79	-0.82	-0.26	-1.51	-0.35	-0.72	-0.89	-1.24	-0.3	-0.53	0.08	0.1	0.05	-0.07	0.43	-0.5
12.7	-0.04	0.06	1	-0.06	-1.24	-0.75	-2.02	-1.15	-1.07	-0.65	0.07	-0.2	-0.08	-1.04	-0.31	-0.56	-0.46	-0.96	0.19	-0.48	0.39	0.25	0.06	0.01	0.4	-0.39
13.7	1.09	1.41	1.67	0.89	0.14	0.39	-0.32	-0.2	0.25	0.11	1.04	0.51	0.17	-0.31	-0.15	-0.21	0.13	-0.51	0.72	-0.3	0.7	0.41	0.13	0.08	0.3	-0.24
14.7	2.05	2.58	2.11	1.76	1.5	1.49	1.41	0.77	1.53	0.89	1.89	1.15	0.4	0.5	0.07	0.23	0.72	-0.01	1.11	-0.06	0.89	0.53	0.2	0.14	0.11	-0.11
15.7	2.57	3.2	2.12	2.24	2.51	2.25	2.75	1.54	2.46	1.47	2.4	1.55	0.53	1.17	0.26	0.61	1.14	0.43	1.2	0.13	0.89	0.51	0.23	0.16	-0.19	-0.03
16.7	2.44	3.02	1.56	2.08	2.88	2.44	3.33	1.91	2.8	1.68	2.42	1.58	0.46	1.52	0.37	0.82	1.27	0.7	0.86	0.19	0.65	0.31	0.16	0.14	-0.6	-0.03
17.7	1.57	1.92	0.44	1.15	2.5	1.92	2.96	1.77	2.41	1.4	1.88	1.18	0.17	1.42	0.33	0.77	1.02	0.77	0.03	0.06	0.15	-0.09	-0.06	0.05	-1.12	-0.11
18.7	0.03	-0.05	-1.14	-0.5	1.4	0.72	1.62	1.13	1.3	0.63	0.82	0.37	-0.34	0.86	0.12	0.44	0.42	0.64	-1.24	-0.29	-0.55	-0.69	-0.42	-0.08	-1.72	-0.27
19.7	-1.94	-2.65	-2.9	-2.64	-0.18	-0.99	-0.46	0.14	-0.36	-0.51	-0.57	-0.73	-0.98	-0.1	-0.26	-0.14	-0.43	0.4	-2.81	-0.79	-1.38	-1.44	-0.91	-0.24	-2.39	-0.48
20.7	-3.97	-5.42	-4.46	-4.84	-1.8	-2.85	-2.84	-0.92	-2.26	-1.77	-1.98	-1.91	-1.62	-1.26	-0.74	-0.85	-1.34	0.2	-4.45	-1.34	-2.18	-2.23	-1.47	-0.41	-3.08	-0.69
21.7	-5.49	-7.73	-5.29	-6.51	-2.81	-4.36	-4.84	-1.63	-3.91	-2.79	-2.98	-2.86	-2.06	-2.29	-1.21	-1.49	-2	0.25	-5.82	-1.77	-2.77	-2.92	-2.01	-0.57	-3.74	-0.81
22.7	-5.86	-8.81	-4.77	-6.88	-2.46	-4.88	-5.61	-1.47	-4.73	-3.07	-3.06	-3.21	-2.04	-2.81	-1.5	-1.82	-2.05	0.8	-6.53	-1.84	-2.92	-3.33	-2.38	-0.67	-4.3	-0.77
23.7	-4.34	-7.79	-2.26	-5.12	0.13	-3.67	-4.19	0.18	-4.02	-2.08	-1.64	-2.54	-1.28	-2.32	-1.41	-1.53	-1.06	2.14	-6.12	-1.27	-2.39	-3.25	-2.44	-0.69	-4.7	-0.45
24.7	-0.23	-3.81	2.89	-0.4	5.82	-0.01	0.41	3.93	-1.11	0.77	1.85	-0.43	0.55	-0.32	-0.69	-0.29	1.4	4.56	-4.14	0.24	-0.93	-2.46	-2.01	-0.57	-4.85	0.25
25.7	7.16	3.95	11.25	8.07	15.43	6.81	9.14	10.36	4.67	6.02	7.95	3.51	3.73	3.7	0.92	2.22	5.76	8.34	-0.17	3.02	1.7	-0.74	-0.92	-0.29	-4.65	1.45
26.7	18.33	16.1	23.22	20.88	29.58	17.37	22.75	19.98	13.84	14.14	17.08	9.61	8.53	10.16	3.69	6.28	12.36	13.68	6.15	7.35	5.67	2.12	0.99	0.19	-4.01	3.24
27.58	31.6	30.81	37	36.09	46.17	30.1	39.23	31.39	25.05	23.96	27.87	16.98	14.28	18.16	7.29	11.31	20.27	19.82	13.88	12.65	10.41	5.71	3.47	0.81	-2.99	5.38
28.56	50.07	51.54	55.74	57.26	69.09	48.05	62.51	47.33	40.98	37.87	42.87	27.38	22.41	29.7	12.72	18.59	31.42	28.21	24.88	20.29	17.06	10.96	7.17	1.73	-1.29	8.45
29.56	72.76	77.21	78.35	83.23	97.11	70.36	91.46	67.05	60.9	55.26	61.31	40.33	32.62	44.34	19.98	27.83	45.32	38.43	38.68	30.07	25.33	17.74	12.02	2.94	1.15	12.36
30.56	98.7	106.7	103.9	112.8	129.1	96.15	124.9	89.82	84.01	75.51	82.47	55.36	44.62	61.63	29	38.75	61.51	50.12	54.81	41.78	34.97	25.88	17.92	4.46	4.38	17.05
31.58	127.5	139.6	131.9	145.5	164.5	125.1	162.4	115.5	110.1	98.44	106.1	72.36	58.43	81.48	39.97	51.28	79.92	63.21	73.21	55.53	45.99	35.46	24.92	6.35	8.55	22.63
32.58	156.8	172.9	160.1	178.5	200.4	154.9	200.7	141.9	136.9	122.2	130.3	89.98	73.05	102.4	52.23	64.45	99.14	76.72	92.49	70.4	57.59	45.84	32.57	8.53	13.5	28.78
33.56	185	204.9	187.1	209.9	235	183.8	237.9	167.8	163.1	145.6	154	107.4	87.86	123.3	65.35	77.64	118.3	90.14	111.9	85.85	69.34	56.63	40.6	11.03	19.13	35.34
34.58	212.4	235.7	213.3	240	268.5	212.3	274.2	193.7	189.1	169	177.4	125	103.2	144.6	79.66	91.08	137.8	103.8	131.9	102.3	81.56	68.18	49.28	14.05	25.74	42.56
35.65	237.3	263.5	237.1	266.9	299.2	238.7	307.6	218	213.4	191	199.5	141.9	118.2	165.3	94.53	104.2	156.6	117.4	151.9	119.1	93.86	80.08	58.42	17.74	33.21	50.28
36.65	256.7	284.3	255.7	287.2	323	259.4	333.9	237.6	233	208.6	217.6	155.9	130.8	182.6	107.5	115.2	172.3	129.3	169.6	133.9	104.7	90.72	66.88	21.77	40.42	57.42
37.56	271.2	299.5	269.9	302.4	341.1	275	354	252.7	248.6	222.1	232.2	167.4	140.6	196.4	117.8	124.3	184.8	139.9	185.2	146.3	113.8	99.78	74.5	26.07	46.79	63.62
38.65	287	315.3	285.6	319.6	361.1	291.6	376.4	269	266.3	236.2	249	180.6	150.1	211.3	126.9	134.7	198.3	153.2	204.6	159.4	124.4	109.8	83.76	32.17	53.42	70.33
39.56	302.8	331.4	301.5	338.9	381.7	307.4	399.5	283.9	284.1	248.8	265.9	193.1	156.8	224.6	131	144.7	210	166.9	223.9	169.4	133.6	117.8	92.28	38.37	57.43	75.18

VIC baseline subtracted fluorescence

Well/ Cycle	B5	B6	B7	B8	B9	C3	C4	C5	C6	C7	C8	C9	D2	D3	D4	D5	D6	D7	D8	D9	E2	E3	E4	E5	E6	E7
0.58	-4.38	-3.48	-3.8	-3.36	-1.07	-1.55	-0.32	0.74	-0.29	-1.7	-0.59	-0.16	-1.23	-0.8	-0.38	2.35	1.43	-0.79	-0.86	-0.57	-0.15	-0.27	3.5	6.28	0.85	3.96
1.7	-2.41	-0.92	-1.96	-3.41	-1.29	-3.18	-1.77	-1.62	-2.69	-2.04	-3.47	-3.04	-2.45	-2.83	-4.16	-5.77	-3.3	-2.64	-7.29	-4.8	-3.24	-5.02	-3.39	-6.04	-5.73	-5.27
2.7	-1.06	0.47	-0.9	-1.88	-0.44	-2.08	-1.18	-1.24	-1.83	-0.75	-2.4	-2.2	-1.43	-1.7	-2.98	-3.93	-2.04	-1.32	-5.08	-3.03	-1.89	-3.38	-2.24	-5.31	-3.6	-4.16
3.7	-0.17	1.11	-0.28	-0.24	0.43	-0.47	-0.18	-0.21	-0.28	0.59	-0.5	-0.55	-0.05	0	-0.75	0.09	0.41	0.38	-1.03	-0.14	0.2	-0.4	0.62	-1.22	0.17	-0.56
4.7	0.29	1.16	0.03	0.89	0.91	0.74	0.57	0.62	0.91	1.36	0.96	0.75	0.92	1.22	1	3.2	2.21	1.4	2.14	1.94	1.61	1.9	2.76	2.42	2.87	2.45
5.7	0.43	0.85	0.14	1.33	0.91	1.25	0.85	0.95	1.39	1.44	1.55	1.29	1.26	1.61	1.74	4.3	2.73	1.5	3.47	2.61	1.93	2.81	3.34	4.08	3.62	3.69
6.7	0.35	0.35	0.14	1.17	0.53	1.1	0.69	0.78	1.19	0.98	1.31	1.09	1.05	1.26	1.5	3.46	2.08	0.86	3.04	2	1.32	2.41	2.45	3.67	2.63	3.16
7.7	0.17	-0.16	0.11	0.61	-0.03	0.53	0.26	0.29	0.55	0.24	0.53	0.42	0.51	0.45	0.66	1.37	0.75	-0.13	1.47	0.63	0.19	1.15	0.67	1.81	0.64	1.45
8.7	-0.03	-0.56	0.08	-0.08	-0.6	-0.19	-0.25	-0.3	-0.22	-0.5	-0.42	-0.4	-0.12	-0.49	-0.37	-1.11	-0.73	-1.08	-0.47	-0.9	-0.98	-0.38	-1.29	-0.58	-1.51	-0.66
9.7	-0.17	-0.79	0.08	-0.7	-1.02	-0.81	-0.65	-0.81	-0.87	-1	-1.21	-1.09	-0.62	-1.26	-1.21	-3.19	-1.86	-1.68	-2.1	-2.08	-1.82	-1.67	-2.84	-2.68	-3.1	-2.44
10.7	-0.22	-0.83	0.12	-1.1	-1.18	-1.16	-0.84	-1.08	-1.2	-1.16	-1.63	-1.44	-0.86	-1.66	-1.6	-4.33	-2.34	-1.77	-2.94	-2.57	-2.09	-2.36	-3.56	-3.87	-3.68	-3.41
11.7	-0.18	-0.69	0.18	-1.19	-1.07	-1.16	-0.77	-1.06	-1.14	-0.95	-1.57	-1.38	-0.78	-1.6	-1.46	-4.29	-2.06	-1.33	-2.81	-2.27	-1.73	-2.31	-3.31	-3.88	-3.12	-3.34
12.7	-0.07	-0.42	0.24	-0.98	-0.71	-0.82	-0.47	-0.75	-0.71	-0.42	-1.06	-0.94	-0.43	-1.12	-0.85	-3.12	-1.12	-0.49	-1.77	-1.27	-0.87	-1.56	-2.19	-2.76	-1.58	-2.31
13.7	0.07	-0.09	0.3	-0.55	-0.19	-0.24	-0.01	-0.25	-0.04	0.28	-0.23	-0.23	0.09	-0.34	0.07	-1.15	0.22	0.54	-0.11	0.16	0.27	-0.32	-0.5	-0.83	0.54	-0.61
14.7	0.21	0.23	0.31	-0.01	0.39	0.43	0.49	0.33	0.71	0.98	0.72	0.56	0.65	0.56	1.06	1.15	1.64	1.49	1.73	1.67	1.41	1.09	1.34	1.43	2.74	1.34
15.7	0.3	0.47	0.28	0.52	0.9	1.03	0.91	0.86	1.35	1.51	1.56	1.22	1.09	1.37	1.85	3.27	2.77	2.11	3.29	2.88	2.24	2.33	2.89	3.46	4.44	3.04
16.7	0.32	0.59	0.21	0.89	1.24	1.42	1.13	1.21	1.72	1.73	2.1	1.59	1.31	1.91	2.24	4.7	3.3	2.24	4.15	3.45	2.53	3.08	3.76	4.73	5.21	4.05
17.7	0.25	0.55	0.11	1.02	1.35	1.48	1.09	1.31	1.69	1.55	2.2	1.54	1.22	2.04	2.08	5.1	3.04	1.77	4.04	3.2	2.16	3.14	3.69	4.9	4.74	4.08
18.7	0.1	0.37	0.01	0.85	1.2	1.19	0.77	1.13	1.24	0.97	1.81	1.04	0.84	1.74	1.39	4.36	1.95	0.8	2.88	2.09	1.16	2.44	2.63	3.84	3.01	3.05
19.7	-0.09	0.09	-0.03	0.39	0.84	0.6	0.23	0.73	0.44	0.12	1.01	0.16	0.26	1.06	0.3	2.63	0.22	-0.45	0.88	0.29	-0.28	1.11	0.76	1.73	0.31	1.12
20.7	-0.27	-0.2	0.04	-0.26	0.38	-0.15	-0.39	0.24	-0.53	-0.82	-0.01	-0.89	-0.34	0.18	-0.89	0.38	-1.79	-1.59	-1.51	-1.78	-1.77	-0.53	-1.48	-0.92	-2.78	-1.24
21.7	-0.34	-0.4	0.3	-0.96	-0.02	-0.81	-0.88	-0.13	-1.38	-1.53	-0.93	-1.82	-0.69	-0.6	-1.74	-1.65	-3.47	-2.09	-3.55	-3.52	-2.77	-1.97	-3.42	-3.29	-5.37	-3.32
22.7	-0.22	-0.36	0.84	-1.48	-0.13	-1.07	-0.95	-0.11	-1.7	-1.62	-1.31	-2.21	-0.45	-0.88	-1.68	-2.44	-4.07	-1.29	-4.29	-4.14	-2.63	-2.53	-4.17	-4.3	-6.32	-4.18
23.7	0.2	0.09	1.75	-1.54	0.3	-0.54	-0.27	0.66	-1.03	-0.64	-0.67	-1.6	0.77	-0.2	-0.09	-0.78	-2.71	1.51	-2.59	-2.73	-0.61	-1.43	-2.74	-2.66	-4.36	-2.71
24.7	1.06	1.1	3.13	-0.84	1.56	1.2	1.53	2.5	1.15	1.88	1.54	0.53	3.38	1.97	3.68	4.6	1.52	7.03	2.71	1.67	4.03	2.14	1.93	2.97	1.85	2.23
25.7	2.45	2.83	5.05	0.94	3.92	4.56	4.81	5.78	5.35	6.39	5.84	4.65	7.74	6.11	10.2	14.95	9.5	15.9	12.76	9.93	11.95	8.96	10.81	13.88	13.54	11.7
26.7	4.48	5.44	7.6	4.07	7.62	9.91	9.91	10.79	12.02	13.29	12.68	11.19	14.17	12.66	19.93	31.35	21.96	28.62	28.5	22.82	23.65	19.67	24.69	31.14	31.67	26.6
27.58	6.85	8.55	10.4	8.16	12.14	16.51	16.15	16.84	20.26	21.58	21.09	19.2	21.75	20.73	31.42	51.48	37.08	43.26	47.68	38.43	37.33	32.69	41.5	52.24	53.54	44.72
28.56	10.15	12.94	14.14	14.25	18.62	26.03	25.09	25.41	32.16	33.31	33.14	30.66	32.26	32.31	47.31	80.28	58.53	63.22	74.93	60.51	56.13	51.13	65.24	82.25	84.29	70.34
29.56	14.24	18.46	18.65	22.22	26.81	38.14	36.49	36.18	47.34	48	48.33	45.07	45.21	46.99	66.71	116.6	85.33	87.36	109	88.02	78.89	74.06	94.64	119.7	122.3	102.1
30.56	19.01	24.99	23.82	31.85	36.47	52.49	50.08	48.8	65.39	65.16	66.14	61.92	60.09	64.29	88.69	159	116.4	114.6	148.6	119.8	104.5	100.5	128.4	163	165.7	138.7
31.58	24.49	32.57	29.71	43.18	47.56	69.06	65.91	63.23	86.31	84.75	86.42	81.06	76.76	84.13	112.8	207.1	151.5	144.6	193.3	155.5	132.3	129.9	165.9	211.4	213.7	179.2
32.58	30.32	40.73	35.99	55.36	59.24	86.63	82.91	78.36	108.6	105.3	107.5	100.9	93.85	104.9	137	256.9	187.4	174.8	239.1	191.9	159.7	159.6	203.6	260.5	261.9	219.9
33.56	36.32	49.2	42.52	67.89	71.01	104.4	100.4	93.54	131.1	125.9	128.5	120.4	110.5	125.7	159.7	305.8	222.5	203.8	283.6	227.1	185.2	187.9	239.4	307.3	307.4	258.5
34.58	42.7	58.32	49.57	81.03	83.11	122.9	118.9	109.1	154.5	146.9	149.5	139.9	127	146.8	181.3	354	256.8	231.8	327.1	261.2	208.5	214.7	273.2	351.6	350	294.6
35.65	49.39	67.91	57.07	94.31	95.02	141.3	137.6	124.3	177.4	167.5	169.7	158.2	142.5	166.9	200.4	398.7	288.2	257.4	366.7	292.1	228.1	238	302.8	390.2	386.4	325.7
36.65	55.61	76.83	64.12	106	105.2	157.2	154	137.2	196.6	184.9	186.4	172.9	154.8	183.4	215	433.5	312.4	277.8	396.7	315.5	241.6	254.8	324.4	417.4	411.7	347.7
37.56	61.35	84.91	70.52	116	113.7	170.7	167.6	148	211.8	199.1	200.1	184.4	164.4	196.1	226.5	459.3	330	294	418.2	332.4	250.7	266.3	339.8	435.1	428.1	362.6
38.65	68.62	94.78	78.16	127.7	123.4	186.4	182.5	160.4	227.3	215	216.2	197	174.9	209.2	241.4	485.6	347.8	313.4	439.3	349.7	261.2	278	356.9	451.4	443.9	378.3
39.56	75.65	103.8	84.78	138.6	132.3	201.1	194.5	172.2	239.2	229.2	232.4	209.1	185.1	220.4	260.1	509.8	364.3	334.7	459	366.7	275.4	291.2	377.3	468.7	462.1	397.4

baseline subtracted fluorescence ratio

26.7	18.33	16.1	23.22	20.88	29.58	17.37	22.75	19.98	13.84	14.14	17.08	9.61	8.53	10.16	3.69	6.28	12.36	13.68	6.15	7.35	5.67	2.12	0.99	0.19	-4.01	3.24
27.58	31.6	30.81	37	36.09	46.17	30.1	39.23	31.39	25.05	23.96	27.87	16.98	14.28	18.16	7.29	11.31	20.27	19.82	13.88	12.65	10.41	5.71	3.47	0.81	-2.99	5.38
28.56	50.07	51.54	55.74	57.26	69.09	48.05	62.51	47.33	40.98	37.87	42.87	27.38	22.41	29.7	12.72	18.59	31.42	28.21	24.88	20.29	17.06	10.96	7.17	1.73	-1.29	8.45
29.56	72.76	77.21	78.35	83.23	97.11	70.36	91.46	67.05	60.9	55.26	61.31	40.33	32.62	44.34	19.98	27.83	45.32	38.43	38.68	30.07	25.33	17.74	12.02	2.94	1.15	12.36
30.56	98.7	106.7	103.9	112.8	129.1	96.15	124.9	89.82	84.01	75.51	82.47	55.36	44.62	61.63	29	38.75	61.51	50.12	54.81	41.78	34.97	25.88	17.92	4.46	4.38	17.05
31.58	127.5	139.6	131.9	145.5	164.5	125.1	162.4	115.5	110.1	98.44	106.1	72.36	58.43	81.48	39.97	51.28	79.92	63.21	73.21	55.53	45.99	35.46	24.92	6.35	8.55	22.63
32.58	156.8	172.9	160.1	178.5	200.4	154.9	200.7	141.9	136.9	122.2	130.3	89.98	73.05	102.4	52.23	64.45	99.14	76.72	92.49	70.4	57.59	45.84	32.57	8.53	13.5	28.78

26.7	4.48	5.44	7.6	4.07	7.62	9.91	9.91	10.79	12.02	13.29	12.68	11.19	14.17	12.66	19.93	31.35	21.96	28.62	28.5	22.82	23.65	19.67	24.69	31.14	31.67	26.6
27.58	6.85	8.55	10.4	8.16	12.14	16.51	16.15	16.84	20.26	21.58	21.09	19.2	21.75	20.73	31.42	51.48	37.08	43.26	47.68	38.43	37.33	32.69	41.5	52.24	53.54	44.72
28.56	10.15	12.94	14.14	14.25	18.62	26.03	25.09	25.41	32.16	33.31	33.14	30.66	32.26	32.31	47.31	80.28	58.53	63.22	74.93	60.51	56.13	51.13	65.24	82.25	84.29	70.34
29.56	14.24	18.46	18.65	22.22	26.81	38.14	36.49	36.18	47.34	48	48.33	45.07	45.21	46.99	66.71	116.6	85.33	87.36	109	88.02	78.89	74.06	94.64	119.7	122.3	102.1
30.56	19.01	24.99	23.82	31.85	36.47	52.49	50.08	48.8	65.39	65.16	66.14	61.92	60.09	64.29	88.69	159	116.4	114.6	148.6	119.8	104.5	100.5	128.4	163	165.7	138.7
31.58	24.49	32.57	29.71	43.18	47.56	69.06	65.91	63.23	86.31	84.75	86.42	81.06	76.76	84.13	112.8	207.1	151.5	144.6	193.3	155.5	132.3	129.9	165.9	211.4	213.7	179.2
32.58	30.32	40.73	35.99	55.36	59.24	86.63	82.91	78.36	108.6	105.3	107.5	100.9	93.85	104.9	137	256.9	187.4	174.8	239.1	191.9	159.7	159.6	203.6	260.5	261.9	219.9

ratio	5.378	4.462	4.849	3.07	3.309	2.443	1.793	1.804	1.276	1.176	1.193	0.895	0.809	0.999	0.413	0.258	0.524	0.43	0.409	0.372	0.37	0.302	0.169	0.035		0.128
