# Supplementary material for: Quantify single nucleotide polymorphism (SNP) ratio in pooled DNA based on normalized fluorescence real-time PCR
Source: BMC Genomics. 2006 Jun 9;7:143. doi: 10.1186/1471-2164-7-143 (PMC1552069; doi:10.1186/1471-2164-7-143)
Supplement: Additional file 5 — Contained the raw and analytical datas used during the procession. provide baseline-subtracted fluorescence ratios. [file 1471-2164-7-143-S5.pdf]

Well/	FAM baseline subtracted fluorescence																								
Cycle	A7	A8	A9	B2	B4	B5	B6	B8	B9	C3	C4	C5	C6	C7	C8	C9	D3	D4	D5	D6	D7	D8	D9	E2	E3	E4
0.58	-11.6	-5.61	-8.18	-3.9	-6.79	-5.2	-6.11	-4.03	-7.2	-5.62	-7.66	-5.33	-6.34	-5.16	-5.26	-5.44	-3.83	-2.53	-3.78	-2.85	-3.81	-9.61	-9.41	-10.5	-6.99	-4.48
1.7	-11.3	-7.01	-9.24	-3.45	-6.19	-5.87	-7.22	-6.43	-7	-5.2	-8.69	-6.29	-6.49	-4.49	-5.8	-5.13	-3.85	-2.94	-4.43	-3.39	-3.6	-5.44	-5.81	-4.8	-5.17	-3.3
2.7	-7.13	-4.23	-5.96	-1.81	-3.41	-3.66	-4.69	-4.25	-4.4	-3.15	-5.73	-4.27	-4.11	-2.55	-3.89	-3.09	-2.4	-2.2	-3	-2.08	-1.27	-1.9	-2.69	-1.7	-3.09	-1.78
3.7	-2.56	-0.88	-2.11	-0.23	-0.65	-1.06	-1.61	-1.18	-1.62	-1.02	-2.12	-1.72	-1.43	-0.64	-1.63	-0.95	-0.78	-1.18	-1.18	-0.52	0.98	0.54	-0.38	-0.02	-1.28	-0.44
4.7	0.82	1.55	0.73	0.79	1.17	0.87	0.74	1.25	0.38	0.51	0.71	0.31	0.6	0.67	0.09	0.58	0.42	-0.27	0.31	0.65	2.24	1.67	0.94	0.67	-0.01	0.46
5.7	2.54	2.59	2.06	1.13	1.84	1.79	1.91	2.47	1.34	1.26	2.26	1.42	1.65	1.23	0.96	1.27	1.02	0.35	1.17	1.18	2.36	1.67	1.37	0.72	0.68	0.86
6.7	2.75	2.37	2.04	0.94	1.59	1.79	2	2.51	1.41	1.31	2.58	1.64	1.79	1.16	1.06	1.23	1.08	0.65	1.39	1.12	1.55	0.92	1.13	0.46	0.89	0.85
7.7	1.95	1.32	1.13	0.44	0.79	1.17	1.34	1.72	0.9	0.91	2.04	1.22	1.31	0.68	0.64	0.7	0.76	0.71	1.13	0.65	0.24	-0.15	0.55	0.12	0.77	0.58
8.7	0.7	-0.04	-0.12	-0.11	-0.13	0.29	0.37	0.54	0.18	0.33	1.08	0.48	0.53	0.04	0	-0.03	0.27	0.61	0.6	0.02	-1.14	-1.15	-0.12	-0.15	0.5	0.17
9.7	-0.47	-1.25	-1.21	-0.53	-0.84	-0.52	-0.53	-0.59	-0.43	-0.2	0.12	-0.28	-0.23	-0.52	-0.59	-0.71	-0.2	0.43	0.01	-0.59	-2.22	-1.81	-0.66	-0.27	0.23	-0.22
10.7	-1.2	-1.99	-1.82	-0.68	-1.13	-1.02	-1.08	-1.37	-0.72	-0.52	-0.55	-0.82	-0.75	-0.86	-0.92	-1.16	-0.51	0.27	-0.48	-1	-2.75	-1.98	-0.93	-0.2	0.04	-0.5
11.7	-1.32	-2.1	-1.8	-0.53	-0.92	-1.11	-1.15	-1.61	-0.62	-0.56	-0.75	-1.01	-0.9	-0.91	-0.91	-1.26	-0.6	0.16	-0.75	-1.13	-2.66	-1.63	-0.89	0.02	-0.01	-0.63
12.7	-0.83	-1.61	-1.18	-0.13	-0.28	-0.8	-0.75	-1.32	-0.15	-0.33	-0.5	-0.83	-0.67	-0.67	-0.57	-1.01	-0.47	0.13	-0.79	-0.98	-2	-0.86	-0.57	0.33	0.07	-0.59
13.7	0.09	-0.67	-0.15	0.42	0.6	-0.19	0	-0.6	0.53	0.09	0.11	-0.36	-0.15	-0.22	0	-0.49	-0.16	0.16	-0.59	-0.6	-0.93	0.15	-0.07	0.65	0.24	-0.41
14.7	1.19	0.43	1.01	0.97	1.51	0.54	0.89	0.34	1.26	0.55	0.85	0.27	0.52	0.32	0.65	0.19	0.23	0.22	-0.25	-0.09	0.28	1.18	0.5	0.88	0.42	-0.15
15.7	2.13	1.42	1.96	1.37	2.2	1.18	1.68	1.25	1.81	0.91	1.49	0.88	1.14	0.81	1.21	0.88	0.58	0.27	0.15	0.43	1.36	2	0.98	0.95	0.53	0.12
16.7	2.63	1.98	2.42	1.49	2.44	1.53	2.14	1.87	2.02	1.04	1.79	1.3	1.56	1.1	1.49	1.42	0.78	0.27	0.5	0.83	2.05	2.4	1.25	0.81	0.49	0.33
17.7	2.45	1.91	2.17	1.24	2.1	1.44	2.12	2.01	1.75	0.84	1.56	1.38	1.61	1.1	1.37	1.7	0.75	0.16	0.72	1	2.15	2.24	1.21	0.43	0.24	0.43
18.7	1.49	1.1	1.11	0.58	1.14	0.85	1.54	1.58	0.99	0.28	0.72	1.05	1.24	0.76	0.79	1.65	0.44	-0.06	0.75	0.9	1.55	1.48	0.83	-0.18	-0.24	0.38
19.7	-0.19	-0.4	-0.67	-0.41	-0.32	-0.22	0.46	0.59	-0.17	-0.58	-0.69	0.31	0.46	0.08	-0.22	1.29	-0.14	-0.39	0.58	0.51	0.26	0.15	0.12	-0.95	-0.94	0.18
20.7	-2.35	-2.39	-2.92	-1.58	-2.05	-1.59	-0.92	-0.79	-1.51	-1.61	-2.5	-0.72	-0.62	-0.83	-1.52	0.69	-0.91	-0.79	0.28	-0.1	-1.55	-1.55	-0.82	-1.78	-1.78	-0.13
21.7	-4.56	-4.49	-5.2	-2.69	-3.66	-3.01	-2.27	-2.24	-2.69	-2.57	-4.35	-1.83	-1.76	-1.8	-2.89	0.04	-1.72	-1.2	-0.06	-0.79	-3.6	-3.36	-1.84	-2.53	-2.64	-0.48
22.7	-6.23	-6.17	-6.92	-3.42	-4.65	-4.09	-3.13	-3.33	-3.25	-3.14	-5.81	-2.72	-2.65	-2.57	-3.98	-0.42	-2.36	-1.5	-0.26	-1.38	-5.46	-4.88	-2.71	-3.03	-3.35	-0.75
23.7	-6.62	-6.8	-7.36	-3.38	-4.43	-4.37	-2.93	-3.5	-2.67	-2.95	-6.28	-2.96	-2.87	-2.82	-4.38	-0.35	-2.57	-1.58	-0.09	-1.6	-6.62	-5.66	-3.16	-3.07	-3.68	-0.79
24.7	-4.92	-5.67	-5.73	-2.14	-2.36	-3.31	-1.05	-2.14	-0.37	-1.55	-5.15	-2.12	-1.95	-2.2	-3.63	0.6	-2.05	-1.27	0.68	-1.17	-6.5	-5.21	-2.92	-2.45	-3.38	-0.44
25.7	-0.28	-2.04	-1.25	0.72	2.19	-0.41	3.14	1.38	4.22	1.47	-1.75	0.3	0.57	-0.32	-1.25	2.83	-0.46	-0.41	2.33	0.2	-4.5	-3.03	-1.68	-0.96	-2.2	0.48
26.7	8.06	4.71	6.77	5.62	9.75	4.85	10.2	7.65	11.6	6.51	4.51	4.77	5.17	3.17	3.22	6.69	2.48	1.17	5.12	2.8	-0.07	1.33	0.8	1.6	0.12	2.15
27.7	20.75	15.12	18.87	12.88	20.75	12.85	20.59	17.12	22.14	13.91	14.14	11.69	12.23	8.57	10.14	12.5	7.06	3.63	9.25	6.86	7.24	8.21	4.75	5.36	3.82	4.73
28.7	38.2	29.48	35.35	22.73	35.4	23.84	34.59	30.11	36.07	23.89	27.44	21.35	22.03	16.12	19.79	20.47	13.48	7.08	14.88	12.55	17.73	17.83	10.27	10.44	9.06	8.32
29.7	60.52	47.82	56.18	35.25	53.68	37.88	52.23	46.65	53.38	36.51	44.51	33.89	34.67	25.9	32.25	30.69	21.84	11.63	22.07	19.94	31.49	30.22	17.38	16.87	15.97	12.99
30.58	84.11	67.1	77.91	48.45	72.61	52.69	70.66	64.04	71.27	49.77	62.56	47.32	48.15	36.38	45.52	41.54	30.82	16.59	29.69	27.83	46.23	43.33	24.9	23.64	23.46	18.01
31.56	114	91.29	105	65.19	96.12	71.4	93.75	85.94	93.5	66.53	85.42	64.56	65.38	49.82	62.46	55.37	42.39	23.1	39.38	37.91	65.06	59.91	34.4	32.23	33.2	24.49
32.56	147.5	118.2	134.9	84.1	122.1	92.38	119.5	110.4	118.1	85.41	111.1	84.26	84.97	65.18	81.67	71.06	55.7	30.78	50.36	49.38	86.37	78.52	45.06	42	44.52	31.94
33.64	185.6	148.3	168.1	105.9	151.1	116.2	148.4	138	145.7	107	140.4	107.1	107.6	83	103.8	89.12	71.26	40.06	63.01	62.6	110.8	99.64	57.18	53.35	57.93	40.69
34.58	218.7	174	196.3	125.1	176.1	136.9	173.4	161.8	169.6	126.1	165.9	127.4	127.7	98.89	123.2	105.1	85.27	48.78	74.23	74.36	132.2	118.1	67.84	63.59	70.18	48.61
35.58	252.2	199.5	224	145	201.3	158	198.6	185.7	193.7	145.8	191.9	148.4	148.6	115.4	143.3	121.6	100	58.48	85.9	86.61	154.3	137	78.92	74.47	83.26	57.01
36.56	281.7	221.6	247.8	163.2	223.8	176.7	220.8	206.7	215.3	163.6	215.3	167.4	167.6	130.5	161.4	136.6	113.7	68.18	96.64	97.98	174.4	154.1	89.36	84.75	95.6	64.93
37.65	309.3	242.1	269.9	180.9	246.1	194.7	241.9	226.7	236.6	181.1	238	185.7	186.6	145.3	178.8	151.3	127.4	78.93	107.5	109.7	194.7	171.4	100.7	95.38	108.1	73.07
38.65	329.6	258	287.1	194.6	265.2	209	258.4	242.6	254.4	194.7	256.2	199.5	201.7	156.7	192.3	162.8	138.3	88.69	116.6	119.9	212.2	186.3	111.7	104.2	118	79.86
39.65	346.4	273.5	304.5	206.1	285.5	222.5	274	257.9	272.5	206.6	273.7	210.4	215.5	166.2	204	172.9	147.6	98.44	125.5	130.5	230.6	202.1	124.9	112.3	126	86.19

E5	E6	E7	E8	E9	F2	F3	F4	F5	F6	F7	F8	F9	G2	G3
-7.79	-5.9	-2.25	-5.09	-2.15	-7.87	-6.59	-6.82	-3.82	-5.57	-4.96	-9.57	-6.73	-10.7	-8.65
-5	-3.93	-3.75	-4.75	-1.46	-4.43	-3.6	-3.67	-3.37	-4.2	-3.45	-4.28	-4.58	-4.22	-4.84
-2.8	-2.54	-3.25	-3.33	-0.77	-2.2	-1.47	-1.76	-2.14	-2.42	-2.3	-1.73	-2.91	-1.26	-2.79
-1.16	-1.48	-2.13	-1.74	-0.31	-0.72	-0.03	-0.54	-0.86	-0.81	-1.36	-0.44	-1.57	-0.01	-1.53
-0.11	-0.69	-1	-0.42	-0.14	0.11	0.74	0.17	0.12	0.34	-0.6	0.12	-0.57	0.25	-0.74
0.42	-0.11	-0.13	0.48	-0.21	0.46	0.97	0.51	0.69	0.95	-0.03	0.31	0.12	0.01	-0.23
0.56	0.31	0.41	0.94	-0.4	0.47	0.8	0.62	0.87	1.09	0.37	0.31	0.52	-0.38	0.14
0.46	0.61	0.65	1.06	-0.6	0.31	0.41	0.59	0.74	0.9	0.63	0.26	0.71	-0.7	0.41
0.28	0.83	0.69	0.96	-0.69	0.08	-0.05	0.5	0.43	0.53	0.76	0.21	0.73	-0.87	0.61
0.1	0.97	0.62	0.76	-0.63	-0.12	-0.47	0.41	0.06	0.13	0.79	0.18	0.65	-0.84	0.76
0	1.07	0.52	0.55	-0.41	-0.24	-0.75	0.33	-0.25	-0.21	0.73	0.16	0.5	-0.63	0.84
0.01	1.1	0.46	0.39	-0.05	-0.25	-0.87	0.28	-0.46	-0.42	0.62	0.15	0.32	-0.31	0.84
0.11	1.07	0.43	0.31	0.38	-0.16	-0.84	0.26	-0.53	-0.49	0.46	0.13	0.14	0.06	0.76
0.28	0.97	0.43	0.29	0.79	-0.01	-0.67	0.24	-0.46	-0.44	0.29	0.11	-0.02	0.41	0.6
0.46	0.79	0.4	0.29	1.11	0.17	-0.44	0.2	-0.3	-0.32	0.1	0.08	-0.14	0.67	0.35
0.59	0.51	0.31	0.27	1.23	0.33	-0.19	0.13	-0.08	-0.18	-0.09	0.04	-0.24	0.79	0.03
0.62	0.13	0.1	0.17	1.1	0.43	0	0.01	0.13	-0.1	-0.26	0.01	-0.31	0.76	-0.34
0.52	-0.32	-0.27	-0.04	0.7	0.42	0.08	-0.17	0.28	-0.13	-0.42	-0.01	-0.36	0.56	-0.75
0.28	-0.84	-0.8	-0.38	0.04	0.31	0.02	-0.39	0.32	-0.29	-0.57	-0.01	-0.39	0.22	-1.17
-0.08	-1.38	-1.46	-0.84	-0.81	0.09	-0.19	-0.64	0.24	-0.58	-0.69	0.02	-0.41	-0.21	-1.57
-0.5	-1.88	-2.18	-1.36	-1.75	-0.22	-0.52	-0.87	0.04	-0.98	-0.78	0.1	-0.42	-0.68	-1.94
-0.89	-2.26	-2.86	-1.86	-2.63	-0.54	-0.93	-1.03	-0.24	-1.4	-0.85	0.23	-0.42	-1.11	-2.24
-1.14	-2.44	-3.35	-2.22	-3.25	-0.79	-1.33	-1.05	-0.53	-1.74	-0.87	0.4	-0.4	-1.41	-2.45
-1.08	-2.31	-3.47	-2.29	-3.39	-0.88	-1.61	-0.84	-0.75	-1.87	-0.85	0.63	-0.35	-1.5	-2.55
-0.57	-1.73	-3.01	-1.9	-2.84	-0.68	-1.64	-0.3	-0.77	-1.63	-0.75	0.93	-0.24	-1.31	-2.5
0.56	-0.57	-1.77	-0.9	-1.39	-0.05	-1.28	0.66	-0.48	-0.87	-0.57	1.29	-0.05	-0.76	-2.28
2.46	1.3	0.44	0.89	1.16	1.15	-0.39	2.14	0.27	0.57	-0.25	1.74	0.25	0.18	-1.86
5.26	4.02	3.79	3.59	4.94	3.04	1.15	4.25	1.58	2.79	0.23	2.31	0.7	1.53	-1.21
9.05	7.72	8.4	7.29	10.02	5.75	3.46	7.06	3.56	5.89	0.92	3.03	1.32	3.28	-0.29
13.85	12.5	14.31	12.03	16.41	9.34	6.59	10.63	6.26	9.9	1.87	3.94	2.15	5.41	0.92
18.93	17.68	20.6	17.06	23.07	13.29	10.06	14.45	9.25	14.17	2.97	4.94	3.08	7.53	2.28
25.38	24.51	28.66	23.49	31.41	18.52	14.65	19.42	13.22	19.62	4.49	6.33	4.34	10.09	4.11
32.74	32.63	37.88	30.83	40.74	24.71	20.07	25.24	17.87	25.79	6.43	8.07	5.88	12.81	6.36
41.3	42.59	48.69	39.41	51.39	32.23	26.59	32.26	23.41	32.93	8.97	10.36	7.83	15.76	9.22
49.03	52.08	58.51	47.2	60.84	39.29	32.66	38.85	28.48	39.29	11.54	12.67	9.74	18.24	12.04
57.26	62.72	69.07	55.61	70.77	47.04	39.3	46.13	33.91	45.99	14.6	15.37	11.97	20.72	15.3
65.13	73.28	79.28	63.85	80.24	54.55	45.77	53.26	39.07	52.33	17.8	18.07	14.29	23.03	18.61
73.46	84.48	90.33	73.03	90.5	62.37	52.74	60.76	44.43	59.11	21.39	20.71	16.93	25.56	22.12
80.78	93.49	100.4	81.85	100.2	68.59	58.85	66.8	48.97	65.39	24.42	22.22	19.32	28.16	24.79
88.12	100.3	110.9	91.8	111	73.44	64.76	71.51	53.32	72.39	26.74	21.96	21.57	31.52	26.35

Well/	VIC baseline subtracted fluorescence																											
Cycle	A7	A8	A9	B2	B4	B5	B6	B8	B9	C3	C4	C5	C6	C7	C8	C9	D3	D4	D5	D6	D7	D8	D9	E2	E3	E4	E5	E6
0.58	-5.13	-4.74	-4.6	-1.17	-0.63	-2.15	-1.17	-1.47	-1.83	-0.14	-1.15	-0.84	-2.24	1.31	0.48	-0.93	0.72	1.38	2.67	-0.82	0.96	7.54	1.94	-2.5	3.1	0.23	2.38	-1.1
1.7	-2.9	-2.82	-3.6	-1.73	-1.72	-1.95	-1.65	-2.29	-3.35	-0.91	-1.77	-1.99	-3.11	-1.86	-2.19	-2.33	-2.15	-3.13	-3.6	-3.49	-3.28	-1.36	-3.51	-2.99	0.08	-2.47	-2.18	-6.74
2.7	-1.56	-1.41	-2.47	-1.35	-1.18	-1.17	-1.07	-1.79	-2.57	-0.57	-1.17	-1.33	-1.84	-1.78	-1.82	-1.64	-1.72	-2.65	-2.43	-1.6	-2.17	-1.66	-2.85	-1.49	0	-1.53	-1.6	-4.61
3.7	-0.69	-0.45	-1.44	-0.72	-0.27	-0.34	-0.31	-0.93	-1.24	-0.02	-0.36	-0.31	-0.24	-0.76	-0.61	-0.47	-0.45	-0.84	0.46	1.02	0.15	0.37	-0.61	0.18	0.67	0.15	0.19	-0.75
4.7	-0.18	0.07	-0.63	-0.15	0.46	0.26	0.27	-0.13	-0.09	0.38	0.25	0.48	0.93	0.26	0.51	0.49	0.67	0.8	2.78	2.81	1.98	2.26	1.35	1.28	1.24	1.41	1.65	2.36
5.7	0.09	0.23	-0.09	0.24	0.82	0.57	0.55	0.4	0.59	0.53	0.51	0.83	1.42	0.87	1.15	0.97	1.23	1.65	3.63	3.26	2.7	3.02	2.3	1.61	1.36	1.83	2.19	3.76
6.7	0.19	0.14	0.22	0.41	0.8	0.61	0.54	0.64	0.76	0.43	0.45	0.75	1.27	0.97	1.23	0.95	1.21	1.64	3.01	2.5	2.32	2.53	2.16	1.28	1.02	1.47	1.8	3.44
7.7	0.2	-0.07	0.35	0.41	0.51	0.45	0.32	0.63	0.56	0.18	0.18	0.38	0.7	0.69	0.9	0.57	0.75	0.98	1.38	0.97	1.18	1.16	1.23	0.53	0.36	0.6	0.78	1.9
8.7	0.16	-0.3	0.38	0.3	0.12	0.2	0.02	0.48	0.16	-0.11	-0.16	-0.12	-0.04	0.19	0.33	0.01	0.1	0.02	-0.62	-0.79	-0.23	-0.52	-0.04	-0.33	-0.39	-0.43	-0.45	-0.18
9.7	0.11	-0.47	0.36	0.15	-0.25	-0.07	-0.26	0.26	-0.25	-0.35	-0.45	-0.57	-0.71	-0.32	-0.25	-0.54	-0.5	-0.9	-2.41	-2.3	-1.49	-1.96	-1.22	-1.05	-1.04	-1.31	-1.51	-2.13
10.7	0.07	-0.53	0.33	0.01	-0.49	-0.26	-0.43	0.06	-0.53	-0.46	-0.61	-0.84	-1.14	-0.68	-0.68	-0.94	-0.9	-1.53	-3.53	-3.21	-2.28	-2.78	-1.99	-1.45	-1.42	-1.82	-2.11	-3.45
11.7	0.05	-0.47	0.34	-0.08	-0.55	-0.36	-0.46	-0.07	-0.6	-0.42	-0.59	-0.88	-1.24	-0.81	-0.87	-1.09	-0.99	-1.71	-3.76	-3.34	-2.43	-2.76	-2.17	-1.47	-1.46	-1.84	-2.14	-3.86
12.7	0.05	-0.32	0.37	-0.1	-0.41	-0.35	-0.34	-0.1	-0.45	-0.24	-0.41	-0.68	-0.99	-0.68	-0.77	-0.99	-0.76	-1.41	-3.09	-2.73	-1.94	-1.94	-1.75	-1.11	-1.16	-1.41	-1.59	-3.32
13.7	0.07	-0.1	0.42	-0.06	-0.13	-0.24	-0.11	-0.04	-0.12	0.02	-0.11	-0.29	-0.48	-0.33	-0.44	-0.65	-0.29	-0.73	-1.69	-1.52	-0.96	-0.51	-0.86	-0.48	-0.59	-0.63	-0.62	-1.97
14.7	0.08	0.14	0.48	0.02	0.22	-0.06	0.18	0.08	0.31	0.32	0.24	0.2	0.19	0.15	0.04	-0.18	0.3	0.17	0.12	0	0.26	1.22	0.3	0.28	0.13	0.3	0.55	-0.14
15.7	0.09	0.34	0.5	0.11	0.55	0.12	0.45	0.21	0.74	0.57	0.56	0.7	0.87	0.64	0.56	0.34	0.86	1.09	1.95	1.51	1.43	2.84	1.43	0.99	0.86	1.17	1.66	1.79
16.7	0.07	0.46	0.47	0.19	0.77	0.28	0.64	0.31	1.06	0.7	0.76	1.07	1.4	1.03	0.98	0.78	1.25	1.8	3.41	2.7	2.26	3.98	2.26	1.49	1.44	1.78	2.43	3.41
17.7	0.04	0.46	0.37	0.22	0.82	0.37	0.7	0.34	1.18	0.68	0.78	1.25	1.67	1.2	1.21	1.06	1.35	2.12	4.2	3.29	2.53	4.35	2.59	1.67	1.77	1.97	2.67	4.39
18.7	-0.01	0.33	0.19	0.18	0.65	0.38	0.59	0.27	1.06	0.47	0.61	1.17	1.62	1.11	1.17	1.09	1.09	1.96	4.12	3.16	2.1	3.8	2.3	1.46	1.77	1.68	2.27	4.52
19.7	-0.06	0.09	-0.06	0.09	0.27	0.31	0.32	0.12	0.71	0.12	0.25	0.84	1.25	0.75	0.87	0.88	0.5	1.29	3.19	2.31	1.01	2.36	1.41	0.89	1.44	0.93	1.25	3.77
20.7	-0.07	-0.22	-0.33	-0.04	-0.25	0.21	-0.05	-0.1	0.18	-0.31	-0.22	0.3	0.65	0.2	0.35	0.45	-0.33	0.22	1.61	0.92	-0.58	0.31	0.11	0.09	0.86	-0.12	-0.24	2.35
21.7	0	-0.53	-0.56	-0.17	-0.81	0.14	-0.44	-0.3	-0.4	-0.69	-0.68	-0.31	-0.01	-0.41	-0.23	-0.08	-1.21	-1.03	-0.17	-0.65	-2.32	-1.87	-1.26	-0.74	0.19	-1.2	-1.86	0.64
22.7	0.2	-0.75	-0.69	-0.22	-1.23	0.21	-0.7	-0.38	-0.84	-0.87	-0.96	-0.82	-0.46	-0.86	-0.67	-0.55	-1.86	-2.13	-1.53	-1.86	-3.69	-3.48	-2.19	-1.3	-0.34	-1.93	-3.18	-0.72
23.7	0.64	-0.77	-0.59	-0.14	-1.31	0.55	-0.65	-0.22	-0.89	-0.62	-0.84	-0.98	-0.38	-0.87	-0.69	-0.71	-1.96	-2.66	-1.64	-2.05	-4.05	-3.62	-2.03	-1.22	-0.4	-1.83	-3.62	-0.94
24.7	1.38	-0.44	-0.16	0.19	-0.8	1.3	-0.1	0.35	-0.29	0.27	-0.08	-0.51	0.62	-0.12	0.05	-0.31	-1.11	-2.12	0.42	-0.44	-2.67	-1.29	-0.06	-0.11	0.34	-0.4	-2.56	0.88
25.7	2.56	0.35	0.74	0.86	0.54	2.63	1.2	1.5	1.26	2.07	1.6	0.88	2.94	1.74	1.89	0.96	1.09	-0.02	5.65	3.75	1.22	4.55	4.48	2.43	2.26	2.88	0.66	5.69
26.7	4.29	1.73	2.24	1.96	2.98	4.71	3.45	3.41	4.04	5	4.45	3.5	6.96	5.05	5.2	3.4	5.02	4.12	14.98	11.31	8.35	14.9	12.32	6.77	5.74	8.5	6.63	14.41
27.7	6.69	3.82	4.46	3.56	6.73	7.68	6.89	6.23	8.3	9.3	8.71	7.63	13.06	10.11	10.3	7.28	11.01	10.71	29.22	22.9	19.36	30.61	24.08	13.21	11.09	16.82	15.87	27.78
28.7	9.88	6.7	7.52	5.74	11.95	11.67	11.68	10.12	14.21	15.13	14.56	13.48	21.49	17.15	17.44	12.84	19.28	20.01	48.94	38.99	34.69	52.3	40.2	21.93	18.55	28.08	28.68	46.34
29.7	14	10.4	11.5	8.5	18.75	16.76	17.95	15.17	21.86	22.61	22.12	21.19	32.41	26.31	26.78	20.22	29.91	32.1	74.39	59.78	54.55	80.22	60.87	32.94	28.24	42.31	45.16	70.27
30.58	18.47	14.35	15.82	11.42	26.06	22.2	24.76	20.62	30.06	30.6	30.25	29.58	44.11	36.15	36.88	28.29	41.21	45	101.5	81.98	75.78	109.9	82.81	44.46	38.63	57.18	62.62	95.69
31.56	24.39	19.44	21.49	15.14	35.51	29.21	33.67	27.74	40.61	40.91	40.79	40.53	59.21	48.86	50	38.84	55.6	61.42	136.2	110.4	103	147.9	110.8	58.91	52.01	75.81	84.74	128
32.56	31.52	25.32	28.2	19.36	46.41	37.32	44.13	36.09	52.71	52.85	53.05	53.28	76.58	63.48	65.18	51.16	71.91	79.92	175.7	142.8	134	190.9	142.5	75.02	67.32	96.53	109.6	164.5
33.64	40.36	32.28	36.37	24.23	59.25	46.95	56.7	46.13	66.87	67	67.65	68.43	96.97	80.6	83.07	65.79	90.64	100.9	221.3	180.3	169.9	240.6	179.1	93.18	85.11	119.8	137.7	206
34.58	48.89	38.68	44.11	28.58	70.91	55.76	68.38	55.49	79.67	80	81.13	82.29	115.4	96.02	99.28	79.13	107.2	119.2	261.7	213.6	201.8	284.5	211.4	108.9	101	139.9	162.2	242.1
35.58	58.57	45.65	52.76	33.12	83.34	65.22	81.15	65.74	93.26	94.01	95.79	97.14	134.9	112.2	116.4	93.3	124.1	137.5	303.5	248.2	234.9	329.7	244.7	124.8	117.4	160.1	186.9	278.7
36.56	68.25	52.5	61.39	37.34	95.09	74.14	93.5	75.74	106.1	107.4	110	111.2	153.2	127.1	132.2	106.5	139.3	153.6	341.5	279.8	265	370.3	274.7	139	132.3	178	208.6	310.8
37.65	78.59	60.02	70.82	41.6	107.2	83.11	106.5	86.35	119.5	121.2	125	125.4	171.5	141.8	147.6	119.4	153.9	168.6	378.6	310.8	294.4	409	303.3	152.9	146.6	195.2	229	340.8
38.65	86.82	66.9	79.01	45.14	117.2	89.96	117.1	95.27	131.1	132.5	137.8	136.7	186.1	153	159.1	129	165	180	407.7	335.1	317.1	437.8	324.6	164.3	157	209	244.7	362.9
39.65	92.74	74	86.44	48.51	126.3	95.04	126.1	103.2	142.4	141.9	149.5	146.1	198.5	161.8	167.5	136	174.4	190.4	433.1	356.4	336.6	460.6	341.5	175.8	164.9	222.8	258.8	381.7

E7	E8	E9	F2	F3	F4	F5	F6	F7	F8	F9	G2	G3
-0.2	1.86	-0.55	0.18	0.56	0.1	1.25	-0.16	0.76	0.12	1.04	-0.15	-0.3
-5.9	-3.75	-3.48	-3.14	-2.6	-3.52	-2.46	-3.8	-4.12	-3.34	-2.36	-2.98	-3.52
-3.97	-2.75	-2.1	-2.12	-1.73	-2.26	-1.67	-2.46	-2.96	-2.11	-1.73	-1.63	-2.34
-0.36	-0.19	0.1	-0.15	0.02	0.02	0.15	-0.05	-0.48	0.1	-0.15	0.44	-0.24
2.5	1.89	1.72	1.4	1.39	1.76	1.59	1.82	1.47	1.78	1.13	1.87	1.41
3.69	2.72	2.27	2.04	1.91	2.4	2.12	2.57	2.21	2.4	1.66	2.23	2.09
3.22	2.29	1.82	1.78	1.61	1.98	1.74	2.22	1.81	2	1.45	1.63	1.84
1.61	1.01	0.72	0.89	0.75	0.87	0.76	1.12	0.67	0.91	0.73	0.48	0.96
-0.46	-0.59	-0.58	-0.22	-0.29	-0.49	-0.46	-0.28	-0.68	-0.42	-0.15	-0.78	-0.15
-2.33	-1.99	-1.69	-1.21	-1.2	-1.67	-1.53	-1.53	-1.79	-1.59	-0.91	-1.76	-1.11
-3.52	-2.81	-2.34	-1.81	-1.73	-2.36	-2.19	-2.33	-2.33	-2.3	-1.34	-2.21	-1.67
-3.78	-2.87	-2.38	-1.88	-1.76	-2.41	-2.3	-2.51	-2.19	-2.4	-1.33	-2.04	-1.7
-3.1	-2.19	-1.84	-1.44	-1.31	-1.85	-1.86	-2.06	-1.41	-1.91	-0.92	-1.31	-1.22
-1.65	-0.93	-0.86	-0.59	-0.5	-0.82	-0.98	-1.11	-0.2	-0.95	-0.22	-0.21	-0.37
0.22	0.62	0.32	0.47	0.49	0.44	0.14	0.13	1.14	0.24	0.59	0.99	0.64
2.09	2.11	1.45	1.5	1.42	1.66	1.25	1.36	2.3	1.4	1.3	2.01	1.57
3.55	3.2	2.26	2.27	2.09	2.54	2.12	2.33	2.96	2.26	1.72	2.59	2.17
4.25	3.62	2.57	2.6	2.32	2.89	2.56	2.81	2.95	2.63	1.71	2.57	2.27
4.01	3.25	2.28	2.39	2.02	2.6	2.45	2.69	2.2	2.39	1.21	1.9	1.79
2.81	2.08	1.43	1.66	1.24	1.69	1.79	1.95	0.82	1.57	0.29	0.66	0.77
0.87	0.35	0.21	0.55	0.1	0.34	0.71	0.77	-0.89	0.32	-0.9	-0.88	-0.63
-1.35	-1.55	-1.04	-0.66	-1.1	-1.09	-0.53	-0.55	-2.45	-1.05	-2.05	-2.31	-2.08
-3.2	-3.02	-1.85	-1.58	-1.98	-2.14	-1.54	-1.57	-3.23	-2.08	-2.79	-3.08	-3.15
-3.82	-3.34	-1.64	-1.72	-2.04	-2.2	-1.84	-1.71	-2.51	-2.23	-2.64	-2.58	-3.32
-2.24	-1.68	0.22	-0.52	-0.76	-0.6	-0.86	-0.35	0.48	-0.88	-1.09	-0.13	-1.98
2.56	2.82	4.41	2.59	2.45	3.35	1.97	3.18	6.47	2.6	2.36	4.92	1.44
11.54	10.95	11.52	8.14	8.08	10.26	7.19	9.5	16.08	8.79	8.15	13.12	7.49
25.51	23.38	22.03	16.57	16.57	20.65	15.26	19.12	29.7	18.14	16.62	24.82	16.57
45.04	40.55	36.24	28.14	28.19	34.82	26.47	32.37	47.44	30.93	27.92	40.17	28.92
70.32	62.59	54.22	42.93	42.99	52.84	40.93	49.34	69.06	47.22	42.03	59.01	44.54
97.29	85.93	73.04	58.51	58.58	71.76	56.28	67.27	90.89	64.31	56.6	78.16	60.84
131.7	115.5	96.68	78.17	78.24	95.51	75.76	89.92	117.2	85.75	74.61	101.4	81.17
170.5	148.9	123	100.1	100.2	121.9	97.65	115.3	145.1	109.6	94.31	126.4	103.6
214.9	186.8	152.8	124.9	125	151.6	122.5	144	174.7	136.2	116	153.1	128.5
253.7	219.9	178.5	146.3	146.5	176.9	144	168.7	198.3	158.9	134.3	174.8	149.6
292.9	253.5	204.3	167.6	167.9	201.9	165.5	193.3	219.9	181.3	152.1	195.1	170.2
327.4	283.2	227.1	186	186.5	223.3	184.1	214.6	236.7	200.4	167.2	211.4	187.7
359.5	311.6	248.6	202.8	203.5	242.3	200.9	233.8	251	217.5	181.1	225.3	203.3
382.7	333.3	265.3	215	215.7	255.6	212.5	247.3	262.4	229.9	192.1	236	214.8
401.4	352.6	280.8	225.3	225.7	266.3	221.6	258.2	277.4	240.7	203.7	248.3	225.6

Well/	baseline subtracted fluorescence ratio																								
Cycle	A7	A8	A9	B2	B4	B5	B6	B8	B9	C3	C4	C5	C6	C7	C8	C9	D3	D4	D5	D6	D7	D8	D9	E2	E3	E4
26.7	8.06	4.71	6.77	5.62	9.75	4.85	10.2	7.65	11.6	6.51	4.51	4.77	5.17	3.17	3.22	6.69	2.48	1.17	5.12	2.8	-0.07	1.33	0.8	1.6	0.12	2.15
27.7	20.75	15.12	18.87	12.88	20.75	12.85	20.59	17.12	22.14	13.91	14.14	11.69	12.23	8.57	10.14	12.5	7.06	3.63	9.25	6.86	7.24	8.21	4.75	5.36	3.82	4.73
28.7	38.2	29.48	35.35	22.73	35.4	23.84	34.59	30.11	36.07	23.89	27.44	21.35	22.03	16.12	19.79	20.47	13.48	7.08	14.88	12.55	17.73	17.83	10.27	10.44	9.06	8.32
29.7	60.52	47.82	56.18	35.25	53.68	37.88	52.23	46.65	53.38	36.51	44.51	33.89	34.67	25.9	32.25	30.69	21.84	11.63	22.07	19.94	31.49	30.22	17.38	16.87	15.97	12.99
30.58	84.11	67.1	77.91	48.45	72.61	52.69	70.66	64.04	71.27	49.77	62.56	47.32	48.15	36.38	45.52	41.54	30.82	16.59	29.69	27.83	46.23	43.33	24.9	23.64	23.46	18.01
31.56	114	91.29	105	65.19	96.12	71.4	93.75	85.94	93.5	66.53	85.42	64.56	65.38	49.82	62.46	55.37	42.39	23.1	39.38	37.91	65.06	59.91	34.4	32.23	33.2	24.49
32.56	147.5	118.2	134.9	84.1	122.1	92.38	119.5	110.4	118.1	85.41	111.1	84.26	84.97	65.18	81.67	71.06	55.7	30.78	50.36	49.38	86.37	78.52	45.06	42	44.52	31.94
33.64	185.6	148.3	168.1	105.9	151.1	116.2	148.4	138	145.7	107	140.4	107.1	107.6	83	103.8	89.12	71.26	40.06	63.01	62.6	110.8	99.64	57.18	53.35	57.93	40.69
26.7	4.29	1.73	2.24	1.96	2.98	4.71	3.45	3.41	4.04	5	4.45	3.5	6.96	5.05	5.2	3.4	5.02	4.12	14.98	11.31	8.35	14.9	12.32	6.77	5.74	8.5
27.7	6.69	3.82	4.46	3.56	6.73	7.68	6.89	6.23	8.3	9.3	8.71	7.63	13.06	10.11	10.3	7.28	11.01	10.71	29.22	22.9	19.36	30.61	24.08	13.21	11.09	16.82
28.7	9.88	6.7	7.52	5.74	11.95	11.67	11.68	10.12	14.21	15.13	14.56	13.48	21.49	17.15	17.44	12.84	19.28	20.01	48.94	38.99	34.69	52.3	40.2	21.93	18.55	28.08
29.7	14	10.4	11.5	8.5	18.75	16.76	17.95	15.17	21.86	22.61	22.12	21.19	32.41	26.31	26.78	20.22	29.91	32.1	74.39	59.78	54.55	80.22	60.87	32.94	28.24	42.31
30.58	18.47	14.35	15.82	11.42	26.06	22.2	24.76	20.62	30.06	30.6	30.25	29.58	44.11	36.15	36.88	28.29	41.21	45	101.5	81.98	75.78	109.9	82.81	44.46	38.63	57.18
31.56	24.39	19.44	21.49	15.14	35.51	29.21	33.67	27.74	40.61	40.91	40.79	40.53	59.21	48.86	50	38.84	55.6	61.42	136.2	110.4	103	147.9	110.8	58.91	52.01	75.81
32.56	31.52	25.32	28.2	19.36	46.41	37.32	44.13	36.09	52.71	52.85	53.05	53.28	76.58	63.48	65.18	51.16	71.91	79.92	175.7	142.8	134	190.9	142.5	75.02	67.32	96.53
33.64	40.36	32.28	36.37	24.23	59.25	46.95	56.7	46.13	66.87	67	67.65	68.43	96.97	80.6	83.07	65.79	90.64	100.9	221.3	180.3	169.9	240.6	179.1	93.18	85.11	119.8
	4.489	4.825	4.953	4.512	2.585	2.694	2.688	3.153	2.108	1.162	2.147	1.597	1.138	1.056	1.287	1.309	0.806	0.402	0.28	0.354	0.689	0.436	0.339	0.599	0.731	0.349

E5	E6	E7	E8	E9	F2	F3	F4	F5	F6	F7	F8	F9	G2	G3
2.46	1.3	0.44	0.89	1.16	1.15	-0.39	2.14	0.27	0.57	-0.25	1.74	0.25	0.18	-1.86
5.26	4.02	3.79	3.59	4.94	3.04	1.15	4.25	1.58	2.79	0.23	2.31	0.7	1.53	-1.21
9.05	7.72	8.4	7.29	10.02	5.75	3.46	7.06	3.56	5.89	0.92	3.03	1.32	3.28	-0.29
13.85	12.5	14.31	12.03	16.41	9.34	6.59	10.63	6.26	9.9	1.87	3.94	2.15	5.41	0.92
18.93	17.68	20.6	17.06	23.07	13.29	10.06	14.45	9.25	14.17	2.97	4.94	3.08	7.53	2.28
25.38	24.51	28.66	23.49	31.41	18.52	14.65	19.42	13.22	19.62	4.49	6.33	4.34	10.09	4.11
32.74	32.63	37.88	30.83	40.74	24.71	20.07	25.24	17.87	25.79	6.43	8.07	5.88	12.81	6.36
41.3	42.59	48.69	39.41	51.39	32.23	26.59	32.26	23.41	32.93	8.97	10.36	7.83	15.76	9.22
6.63	14.41	11.54	10.95	11.52	8.14	8.08	10.26	7.19	9.5	16.08	8.79	8.15	13.12	7.49
15.87	27.78	25.51	23.38	22.03	16.57	16.57	20.65	15.26	19.12	29.7	18.14	16.62	24.82	16.57
28.68	46.34	45.04	40.55	36.24	28.14	28.19	34.82	26.47	32.37	47.44	30.93	27.92	40.17	28.92
45.16	70.27	70.32	62.59	54.22	42.93	42.99	52.84	40.93	49.34	69.06	47.22	42.03	59.01	44.54
62.62	95.69	97.29	85.93	73.04	58.51	58.58	71.76	56.28	67.27	90.89	64.31	56.6	78.16	60.84
84.74	128	131.7	115.5	96.68	78.17	78.24	95.51	75.76	89.92	117.2	85.75	74.61	101.4	81.17
109.6	164.5	170.5	148.9	123	100.1	100.2	121.9	97.65	115.3	145.1	109.6	94.31	126.4	103.6
137.7	206	214.9	186.8	152.8	124.9	125	151.6	122.5	144	174.7	136.2	116	153.1	128.5
0.295	0.215	0.237	0.219	0.355	0.259	0.226	0.207	0.198	0.239	0.067	0.072	0.077	0.11	0.099
