# Supplementary material for: Quantify single nucleotide polymorphism (SNP) ratio in pooled DNA based on normalized fluorescence real-time PCR
Source: BMC Genomics. 2006 Jun 9;7:143. doi: 10.1186/1471-2164-7-143 (PMC1552069; doi:10.1186/1471-2164-7-143)
Supplement: Additional file 6 — Contained the raw and analytical datas used during the procession. provide baseline-subtracted fluorescence ratios. [file 1471-2164-7-143-S6.pdf]

Well/	FAM baseline subtracted fluorescence																									
Cycle	C2	C3	C4	C5	C6	C7	C8	C9	D2	D3	D4	D5	D6	D7	D8	D9	E2	E3	E4	E5	E6	E7	E8	E9	F2	F3
0.56	-2.56	-7.01	-4.65	6.06	1.63	0.47	-3.17	1.73	-0.35	2.29	3.48	0.13	2.94	7.46	0.32	-2.92	-4.07	3.33	-2.27	0.96	2.3	3.62	0.37	1.59	-2.32	-0.85
1.56	-4.35	-4.11	-4.15	3.58	-1.81	-3.55	-4.28	-0.58	-0.92	0.61	2.8	-2.34	-0.73	1.93	-2.03	-4.54	-1.9	1.51	-2.21	-2.89	-3.24	1.43	-1.44	-1.04	-1.39	-1.68
2.56	-3.92	-1.28	-2.3	2.93	-1.73	-3.69	-2.77	-0.58	-0.72	0.27	2.34	-2.1	-1.39	0.59	-1.91	-4.47	-0.78	0.74	-1.43	-3.13	-4.6	0.73	-1.65	-1.58	-0.9	-1.02
3.56	-2.86	0.4	-0.78	2.42	-0.77	-2.58	-1.04	-0.08	-0.51	0.17	1.72	-1.17	-1.06	0.65	-1.21	-3.83	-0.4	0.33	-0.68	-2.08	-4.01	0.44	-1.25	-1.28	-0.76	-0.1
4.56	-1.88	0.78	-0.15	1.47	-0.12	-1.47	-0.08	0.17	-0.51	-0.08	0.86	-0.47	-0.72	0.76	-0.76	-3.09	-0.43	-0.01	-0.26	-0.93	-2.79	0.13	-0.72	-0.8	-0.85	0.54
5.56	-1.2	0.2	-0.3	0.12	-0.07	-0.8	0	0.02	-0.7	-0.53	-0.08	-0.27	-0.67	0.46	-0.73	-2.38	-0.61	-0.33	-0.14	-0.17	-1.56	-0.29	-0.27	-0.4	-1.01	0.72
6.56	-0.76	-0.81	-0.83	-1.31	-0.47	-0.51	-0.49	-0.38	-0.94	-0.99	-0.94	-0.45	-0.85	-0.18	-0.97	-1.65	-0.76	-0.58	-0.2	0.14	-0.57	-0.74	0.07	-0.14	-1.09	0.51
7.56	-0.39	-1.72	-1.3	-2.44	-0.95	-0.36	-1.07	-0.74	-1.05	-1.27	-1.53	-0.75	-1.08	-0.89	-1.19	-0.81	-0.74	-0.69	-0.27	0.16	0.16	-1.07	0.35	0.05	-1.02	0.1
8.56	0.05	-2.14	-1.37	-2.96	-1.19	-0.11	-1.34	-0.86	-0.91	-1.21	-1.75	-0.91	-1.12	-1.37	-1.16	0.19	-0.52	-0.59	-0.21	0.09	0.7	-1.15	0.63	0.24	-0.74	-0.33
9.56	0.63	-1.89	-0.87	-2.73	-0.95	0.4	-1.07	-0.59	-0.51	-0.76	-1.57	-0.78	-0.86	-1.4	-0.75	1.32	-0.13	-0.27	0.06	0.12	1.14	-0.92	0.94	0.49	-0.3	-0.63
10.56	1.34	-1	0.16	-1.81	-0.22	1.19	-0.23	0.05	0.13	0.03	-1.07	-0.28	-0.26	-0.93	0.06	2.47	0.36	0.2	0.51	0.34	1.54	-0.4	1.27	0.81	0.24	-0.7
11.56	2.06	0.33	1.51	-0.42	0.88	2.11	1.03	0.93	0.86	1	-0.36	0.49	0.58	-0.05	1.11	3.47	0.85	0.75	1.06	0.73	1.9	0.3	1.56	1.16	0.79	-0.54
12.56	2.61	1.78	2.83	1.13	2.09	2.96	2.4	1.82	1.5	1.92	0.37	1.35	1.48	1.04	2.19	4.11	1.2	1.23	1.58	1.21	2.17	1.03	1.74	1.44	1.21	-0.2
13.56	2.8	2.96	3.74	2.43	3.06	3.43	3.5	2.47	1.89	2.56	0.97	2.07	2.21	2.03	3.02	4.18	1.33	1.51	1.91	1.62	2.25	1.6	1.7	1.57	1.41	0.22
14.56	2.45	3.5	3.87	3.13	3.46	3.25	3.93	2.62	1.84	2.68	1.27	2.38	2.52	2.61	3.31	3.51	1.14	1.46	1.9	1.77	2.05	1.83	1.37	1.43	1.28	0.59
15.56	1.43	3.14	2.99	2.94	3.02	2.23	3.42	2.09	1.25	2.12	1.17	2.09	2.22	2.52	2.88	2.03	0.6	1.01	1.44	1.52	1.49	1.57	0.68	0.94	0.79	0.78
16.58	-0.29	1.72	0.95	1.7	1.57	0.26	1.75	0.78	0.08	0.79	0.65	1.05	1.19	1.59	1.61	-0.27	-0.28	0.11	0.45	0.72	0.51	0.73	-0.4	0.07	-0.08	0.7
17.58	-2.53	-0.62	-2.02	-0.47	-0.76	-2.45	-0.92	-1.14	-1.53	-1.15	-0.2	-0.68	-0.51	-0.14	-0.38	-3.09	-1.38	-1.11	-0.97	-0.57	-0.8	-0.63	-1.74	-1.1	-1.22	0.31
18.58	-5.02	-3.55	-5.51	-3.28	-3.68	-5.6	-4.31	-3.43	-3.37	-3.5	-1.17	-2.91	-2.69	-2.45	-2.82	-6.13	-2.53	-2.52	-2.68	-2.24	-2.33	-2.37	-3.24	-2.46	-2.52	-0.37
19.58	-7.35	-6.51	-8.85	-6.19	-6.64	-8.63	-7.83	-5.6	-5.08	-5.84	-1.96	-5.28	-4.98	-4.91	-5.25	-8.87	-3.52	-3.85	-4.42	-4.04	-3.83	-4.23	-4.65	-3.79	-3.77	-1.22
20.58	-8.93	-8.69	-11.1	-8.42	-8.85	-10.7	-10.7	-7.03	-6.18	-7.6	-2.14	-7.25	-6.85	-6.87	-7.02	-10.7	-4.03	-4.73	-5.81	-5.54	-4.98	-5.83	-5.66	-4.78	-4.7	-2.04
21.58	-9.06	-9.11	-11.1	-9.02	-9.3	-11	-11.8	-6.92	-6.12	-8.08	-1.22	-8.1	-7.63	-7.5	-7.3	-10.8	-3.72	-4.73	-6.39	-6.23	-5.32	-6.67	-5.9	-5.05	-5	-2.55
22.58	-7	-6.63	-7.58	-6.9	-6.84	-8.3	-10.2	-4.41	-4.24	-6.48	1.38	-7.02	-6.51	-5.83	-5.19	-8.37	-2.2	-3.33	-5.63	-5.46	-4.34	-6.2	-4.94	-4.16	-4.33	-2.4
23.58	-1.96	-0.08	0.85	-0.91	-0.25	-1.52	-4.39	1.42	0.13	-1.96	6.24	-3.13	-2.65	-0.79	0.3	-2.61	0.93	-0.01	-2.95	-2.55	-1.5	-3.8	-2.32	-1.66	-2.31	-1.21
24.58	6.81	11.69	15.52	10.06	11.67	10.42	6.56	11.43	7.61	6.31	13.93	4.47	4.79	8.67	10.11	7.27	6.07	5.76	2.22	3.21	3.77	1.12	2.41	2.9	1.39	1.43
25.58	19.92	29.68	37.59	26.99	30	28.48	23.71	26.33	18.78	19.08	24.95	16.6	16.59	23.51	25.07	21.93	13.57	14.5	10.39	12.48	12	9.14	9.65	9.94	7.09	5.92
26.58	37.79	54.64	67.95	50.61	55.56	53.38	47.73	46.64	34.06	36.94	39.69	33.91	33.36	44.49	45.79	41.82	23.73	26.59	21.97	25.79	23.62	20.69	19.74	19.76	15.04	12.6
27.58	60.62	86.97	107.1	81.33	88.84	85.48	78.94	72.58	53.7	60.27	58.38	56.84	55.49	72.1	72.64	67.15	36.77	42.35	37.25	43.5	38.93	36.1	32.89	32.56	25.4	21.7
28.56	87.65	125.7	154	118.3	129	123.8	116.3	103.3	77.16	88.5	80.56	84.87	82.45	105.7	104.8	97.11	52.44	61.43	55.89	65.24	57.62	55.03	48.8	48.02	37.93	33.11
29.61	121.2	174.5	213	164.8	179.6	171.9	162.9	141.4	106.6	124.4	108.4	120.8	116.9	148.4	145.3	134.4	72.38	85.82	79.76	93.16	81.59	79.41	69.06	67.62	53.88	48.02
30.56	155	224.2	273.4	212.4	231.5	220.8	210	179.7	136.8	161.4	136.8	158	152.4	192.6	186.7	172.1	93.14	111.2	104.6	122.2	106.6	104.9	90.06	87.79	70.43	63.78
31.63	195.3	284.2	346.6	269.8	294.5	279.6	266	225.2	173.3	206.8	171.5	203.9	196	246.6	236.7	217.2	119	142.8	135.2	158	137.5	136.4	116	112.4	90.87	83.52
32.63	234.2	342.9	418.8	326.2	356.4	337	319.7	269	209.4	252	206.1	249.7	239.4	300.2	285.8	261.3	145.5	174.7	166	193.9	168.7	168.2	142	137	111.5	103.6
33.63	272.6	401.4	491.6	382.8	418.5	394.1	372.2	311.9	245.9	298.1	241.7	296.5	283.5	354.7	335	305.2	173.4	208.1	197.7	230.7	201	201.2	169	162	133	124.6
34.63	308.9	457.7	562.5	437.7	478.5	448.9	421.2	352.3	281.6	343.6	277.3	342.9	326.9	408.3	382.8	347.6	202.2	241.8	229.4	267.1	233.5	234.5	196.1	186.8	154.7	145.8
35.63	342.4	510	629.3	489.4	534.5	499.6	465.6	389.1	315.5	386.9	312.2	387.4	368.3	459.4	427.8	387.1	231.3	275.1	260.3	302.1	265.3	267.3	222.7	210.8	176.1	166.8
36.63	372.7	557.3	691	537.5	585.4	545.6	505.5	422.1	347	427.1	346.2	429.3	407	507.6	469.7	423.4	260	307.1	289.8	335.2	296.1	299.3	248.5	233.8	196.9	187.4
37.65	401.8	601.6	749.3	584.8	633.1	589	544.3	453.6	377.1	465	380.4	470	444.5	554.8	510.5	458	288.8	338.3	318.6	367.5	326.8	331.7	274.1	256.8	217.6	208.3
38.65	431.6	644.2	804.7	632.9	678.5	631.1	586.1	486	405.7	500.1	414.7	509.7	481	602	551.7	491.7	316.1	367.5	346.5	399.4	357.3	364.6	299.1	280.2	237.5	229.6
39.65	468.7	693	865.7	691.1	729.9	680.5	642.7	527.5	437	536.6	453.1	554	522.4	657.1	600.8	530.4	343.3	396.8	376.6	435.6	391.1	402.1	326.4	307.6	258.4	254.2

F4	F5	F6	F7	F8	F9	G2	G3	G4	G5
-5.18	-6.11	-0.6	6.32	-1.12	-1.31	1.3	-7.77	0.44	-3.7
-1.04	-3.95	-0.1	3.65	-3.46	-2.47	-1.33	-4.05	-0.68	-2.28
0.58	-2.41	-0.28	1.56	-3.62	-2.51	-2.35	-2.15	-0.93	-1.18
0.73	-1.41	-0.73	0	-2.84	-2.06	-2.4	-1.27	-0.82	-0.45
0.17	-0.82	-1.17	-1.03	-1.81	-1.45	-1.92	-0.89	-0.62	-0.08
-0.56	-0.49	-1.42	-1.55	-0.89	-0.86	-1.2	-0.67	-0.44	0.04
-1.11	-0.27	-1.4	-1.58	-0.2	-0.33	-0.44	-0.43	-0.31	0
-1.33	-0.06	-1.1	-1.19	0.28	0.13	0.25	-0.1	-0.21	-0.08
-1.18	0.19	-0.57	-0.49	0.6	0.54	0.8	0.31	-0.12	-0.15
-0.71	0.5	0.11	0.4	0.82	0.89	1.19	0.74	-0.01	-0.14
-0.04	0.84	0.81	1.32	0.99	1.19	1.41	1.13	0.14	-0.06
0.66	1.14	1.44	2.11	1.11	1.41	1.45	1.4	0.32	0.08
1.24	1.32	1.87	2.63	1.18	1.49	1.34	1.46	0.48	0.23
1.56	1.31	2.02	2.74	1.16	1.42	1.08	1.26	0.59	0.34
1.5	1.03	1.83	2.37	0.99	1.14	0.68	0.78	0.6	0.34
1.03	0.46	1.28	1.48	0.62	0.65	0.15	0.01	0.46	0.18
0.11	-0.45	0.38	0.07	0.04	-0.05	-0.51	-1.04	0.13	-0.18
-1.13	-1.58	-0.76	-1.73	-0.72	-0.89	-1.25	-2.27	-0.41	-0.76
-2.55	-2.87	-2.05	-3.78	-1.61	-1.77	-2.08	-3.62	-1.14	-1.55
-3.96	-4.14	-3.33	-5.88	-2.52	-2.58	-2.94	-4.98	-2.02	-2.49
-5.09	-5.2	-4.4	-7.78	-3.27	-3.12	-3.81	-6.23	-2.98	-3.52
-5.61	-5.78	-5.05	-9.2	-3.65	-3.21	-4.62	-7.24	-3.91	-4.54
-5.21	-5.61	-5.02	-9.84	-3.42	-2.63	-5.29	-7.88	-4.68	-5.41
-3.53	-4.38	-4.08	-9.42	-2.3	-1.16	-5.74	-8.02	-5.14	-6.01
-0.23	-1.78	-1.97	-7.66	-0.03	1.4	-5.86	-7.52	-5.11	-6.17
4.99	2.46	1.52	-4.36	3.63	5.22	-5.52	-6.27	-4.43	-5.75
12.39	8.59	6.57	0.63	8.88	10.41	-4.61	-4.19	-2.93	-4.65
22.15	16.8	13.34	7.36	15.81	17.02	-2.99	-1.21	-0.46	-2.75
34.09	26.95	21.72	15.58	24.27	24.8	-0.59	2.63	2.98	-0.06
49.49	40.14	32.61	26.03	35.04	34.44	3	7.76	7.87	3.72
65.69	54.11	44.16	36.73	46.15	44.12	7.24	13.32	13.41	7.92
86.04	71.75	58.77	49.69	59.69	55.64	13.17	20.48	20.79	13.37
107	90	73.96	62.47	73.1	66.78	19.9	28.07	28.8	19.09
129.4	109.5	90.24	75.41	86.67	77.8	27.68	36.37	37.72	25.21
152.7	129.8	107.3	88.23	99.94	88.39	36.39	45.26	47.43	31.56
176.4	150.5	124.6	100.8	112.6	98.34	45.79	54.58	57.82	38.01
200.2	171.4	142	113.4	124.6	107.8	55.52	64.19	68.85	44.53
224.4	192.8	159.4	126.9	136.6	117.4	65.33	74.15	80.93	51.44
247.9	213.9	175.8	142.2	149	127.8	74.08	83.82	93.81	58.81
271.6	236	191.5	162	164.4	141.5	81.2	93.28	108.5	67.6

Well/	VIC baseline subtracted fluorescence																									
Cycle	C2	C3	C4	C5	C6	C7	C8	C9	D2	D3	D4	D5	D6	D7	D8	D9	E2	E3	E4	E5	E6	E7	E8	E9	F2	F3
0.56	-2.3	-1.56	-2.12	0.75	0.75	-1.4	0.53	0.79	-1.28	1.11	1.68	4.1	3.71	4.4	1.24	-3.53	-0.01	2.7	4.51	3.72	1.7	-1.83	0.5	-0.72	0.76	1.48
1.56	-2	-1.2	-2.13	0.23	-2.52	-1.18	-1.94	-0.71	-1.84	-0.95	-1.02	-1.97	-2.18	-0.84	-1.2	-5.7	-1	-1.14	-2.65	-1.73	-2.96	-3.96	-3.43	-2.86	-1.34	-1.73
2.56	-1.38	-0.54	-1.56	0.26	-2.61	-0.35	-2.07	-1.03	-1.17	-1	-1.06	-2.29	-2.47	-1.19	-0.17	-4.01	-0.65	-1.12	-3.14	-2.45	-2.18	-2.68	-2.88	-2.17	-0.66	-1.39
3.56	-0.74	0.04	-0.84	0.42	-1.46	0.39	-1.27	-0.84	-0.28	-0.38	-0.23	-0.62	-0.8	0.06	1.53	-1.41	-0.02	0.09	-1.23	-1.35	0.09	-0.68	-1.01	-0.73	0.6	-0.07
4.56	-0.23	0.37	-0.18	0.52	-0.17	0.75	-0.31	-0.51	0.34	0.21	0.52	1.05	0.88	1.21	2.57	0.64	0.38	1.12	0.78	-0.01	1.86	0.76	0.59	0.42	1.39	0.99
5.56	0.12	0.44	0.3	0.47	0.74	0.7	0.41	-0.22	0.57	0.51	0.85	1.88	1.74	1.6	2.56	1.63	0.42	1.47	1.9	0.86	2.42	1.26	1.32	0.94	1.44	1.34
6.56	0.3	0.3	0.58	0.31	1.12	0.34	0.79	-0.03	0.44	0.48	0.72	1.74	1.64	1.19	1.64	1.64	0.19	1.14	1.98	1.1	1.84	0.94	1.22	0.86	0.88	1.02
7.56	0.36	0.04	0.67	0.08	1.04	-0.17	0.86	0.08	0.11	0.23	0.29	0.91	0.86	0.26	0.23	1	-0.17	0.37	1.27	0.84	0.55	0.16	0.59	0.42	0.01	0.3
8.56	0.36	-0.24	0.63	-0.15	0.67	-0.67	0.71	0.14	-0.27	-0.1	-0.23	-0.21	-0.22	-0.79	-1.21	0.13	-0.49	-0.5	0.22	0.34	-0.92	-0.67	-0.19	-0.11	-0.82	-0.48
9.56	0.32	-0.46	0.5	-0.32	0.19	-1.02	0.48	0.2	-0.55	-0.37	-0.65	-1.23	-1.21	-1.62	-2.29	-0.61	-0.65	-1.17	-0.77	-0.16	-2.07	-1.22	-0.77	-0.51	-1.36	-1.04
10.56	0.28	-0.58	0.33	-0.4	-0.22	-1.15	0.26	0.27	-0.67	-0.5	-0.85	-1.85	-1.82	-1.98	-2.79	-0.99	-0.6	-1.47	-1.37	-0.46	-2.62	-1.32	-0.96	-0.63	-1.45	-1.21
11.56	0.26	-0.58	0.15	-0.39	-0.48	-1.04	0.11	0.36	-0.59	-0.45	-0.77	-1.95	-1.91	-1.81	-2.62	-0.92	-0.34	-1.33	-1.43	-0.49	-2.44	-0.94	-0.71	-0.45	-1.11	-0.96
12.56	0.25	-0.46	0	-0.29	-0.54	-0.73	0.05	0.45	-0.35	-0.24	-0.44	-1.53	-1.47	-1.16	-1.88	-0.47	0.06	-0.8	-0.99	-0.24	-1.63	-0.18	-0.1	-0.02	-0.42	-0.37
13.56	0.24	-0.27	-0.12	-0.13	-0.43	-0.29	0.06	0.5	-0.01	0.06	0.04	-0.75	-0.64	-0.2	-0.76	0.21	0.49	-0.04	-0.18	0.18	-0.41	0.74	0.66	0.51	0.41	0.4
14.56	0.22	-0.04	-0.21	0.04	-0.2	0.19	0.1	0.49	0.34	0.36	0.54	0.17	0.39	0.81	0.47	0.9	0.83	0.75	0.75	0.63	0.9	1.58	1.35	0.98	1.16	1.14
15.56	0.18	0.17	-0.26	0.2	0.06	0.62	0.12	0.38	0.6	0.56	0.93	0.98	1.35	1.62	1.54	1.36	0.99	1.35	1.54	0.95	1.97	2.09	1.73	1.24	1.63	1.65
16.58	0.09	0.33	-0.27	0.3	0.27	0.93	0.06	0.16	0.69	0.59	1.09	1.46	2.06	2.01	2.23	1.43	0.88	1.61	1.96	1.02	2.53	2.08	1.62	1.17	1.68	1.76
17.58	-0.02	0.4	-0.24	0.33	0.35	1.05	-0.1	-0.15	0.6	0.42	0.97	1.42	2.33	1.85	2.41	1.02	0.51	1.43	1.84	0.76	2.4	1.52	1	0.74	1.25	1.42
18.58	-0.15	0.38	-0.15	0.31	0.3	1	-0.34	-0.53	0.32	0.05	0.57	0.88	2.14	1.17	2.13	0.21	-0.04	0.84	1.21	0.23	1.62	0.5	-0.03	0.04	0.46	0.7
19.58	-0.27	0.32	0.02	0.27	0.16	0.84	-0.61	-0.88	-0.04	-0.41	0	-0.04	1.6	0.12	1.61	-0.78	-0.63	0.01	0.24	-0.43	0.42	-0.7	-1.18	-0.72	-0.45	-0.2
20.58	-0.3	0.25	0.31	0.28	0.05	0.69	-0.79	-1.1	-0.35	-0.81	-0.55	-1.05	0.98	-0.93	1.22	-1.57	-1.02	-0.76	-0.74	-0.92	-0.79	-1.62	-1.98	-1.21	-1.09	-0.91
21.58	-0.2	0.29	0.78	0.42	0.14	0.73	-0.74	-1.03	-0.4	-0.93	-0.79	-1.66	0.69	-1.51	1.55	-1.61	-0.89	-1.04	-1.19	-0.85	-1.36	-1.65	-1.81	-1.01	-0.95	-0.96
22.58	0.12	0.54	1.51	0.82	0.69	1.18	-0.24	-0.52	0.05	-0.49	-0.38	-1.29	1.3	-0.97	3.31	-0.24	0.11	-0.29	-0.46	0.28	-0.52	-0.04	0.1	0.41	0.61	0.24
23.58	0.76	1.14	2.6	1.61	2	2.32	0.94	0.63	1.3	0.82	1.08	0.79	3.43	1.42	7.31	3.26	2.38	2.09	2.23	3.04	2.63	4.02	4.56	3.62	4.23	3.31
24.58	1.8	2.25	4.14	2.93	4.4	4.42	3.04	2.61	3.64	3.34	4.02	5.33	7.79	6.4	14.41	9.65	6.31	6.71	7.65	8.01	9	11.34	12.4	9.18	10.57	8.9
25.58	3.32	4.03	6.25	4.94	8.24	7.77	6.31	5.61	7.34	7.4	8.84	13.08	15.06	14.71	25.43	19.62	12.26	14.13	16.55	15.76	19.42	22.69	24.36	17.59	20.19	17.57
26.58	5.41	6.6	9.05	7.75	13.83	12.62	10.97	9.77	12.63	13.26	15.88	24.69	25.82	26.94	41.06	33.73	20.51	24.82	29.57	26.76	34.59	38.64	41.01	29.25	33.51	29.75
27.58	8.09	10.11	12.64	11.48	21.44	19.18	17.19	15.22	19.67	21.14	25.37	40.66	40.5	43.54	61.82	52.34	31.22	39.05	47.09	41.33	54.92	59.57	62.69	44.37	50.73	45.69
28.56	11.33	14.52	17.02	16.08	31.04	27.39	24.89	21.84	28.31	30.91	37.13	60.75	58.88	64.17	87.34	74.99	44.11	56.47	68.73	59.18	79.89	84.91	88.79	62.53	71.3	64.89
29.61	15.45	20.35	22.75	22.09	43.74	38.18	34.9	30.35	39.41	43.6	52.38	87.13	82.86	90.91	120.3	103.9	60.4	78.7	96.55	82.08	111.8	117	121.7	85.36	96.95	89.04
30.56	19.73	26.64	28.93	28.46	57.38	49.67	45.47	39.25	50.96	56.95	68.32	115.1	108.1	118.9	154.7	133.6	77.08	101.6	125.4	105.9	144.7	150	155.2	108.6	122.8	113.5
31.63	25.02	34.68	36.96	36.53	74.75	64.23	58.73	50.34	65.16	73.58	88.02	150	139.3	153.3	197.2	169.7	97.29	129.3	160.4	135	184.4	189.7	195.5	136.4	153.3	142.7
32.63	30.39	43.13	45.59	44.9	92.82	79.26	72.32	61.67	79.32	90.44	107.8	185.4	170.7	187.6	240	205.4	117.2	156.3	194.9	164	222.9	228.5	234.5	163.4	182.4	170.7
33.63	36.02	52.25	55.21	53.84	112.1	95.21	86.59	73.57	93.76	107.9	127.9	222	202.6	222.4	283.9	241.1	137.1	183	229	193.3	260.8	267	272.8	190	210.3	197.7
34.63	41.8	61.86	65.73	63.16	132	111.6	101.2	85.76	108	125.5	147.8	258.7	233.8	256.3	327.2	275.7	156.3	208.2	261.4	221.7	296.1	303.3	308.8	215	235.6	222.4
35.63	47.66	71.82	77.03	72.69	152.1	128.1	115.7	98.03	121.6	142.6	166.7	294.5	263.2	288.2	368.6	308.1	174.3	231.1	290.8	248.2	327.5	336.2	341.1	237.5	257.6	244
36.63	53.65	82.02	88.98	82.3	172.1	144.4	130.1	110.3	134.4	159.1	184.6	328.8	290.2	317.5	407	338	190.7	251.4	316.7	272	354.5	365	369.1	257.4	275.9	262
37.65	60.1	92.79	101.7	92.27	192.3	160.8	144.8	122.8	147	175.5	202.2	363.3	315.7	345.6	443.4	367.3	206.4	270.4	340.7	293.7	378.7	390.7	394.2	275.8	292.2	277.7
38.65	67.11	103.9	114.6	102.4	212.3	177.1	159.8	135.6	159.9	191.8	220	398.6	340.5	373.5	477.8	397.4	221.9	289.6	364.4	313.7	402.7	414.6	418.3	294	308.7	293.3
39.65	75.55	116.2	127.8	113.4	233.9	194.6	176.9	149.9	175.4	210.2	241.2	440.5	369.4	406.8	514.9	435.1	240.2	314.5	394.6	335.7	434.3	442.8	448.1	317	331.9	314.5

F4	F5	F6	F7	F8	F9	G2	G3	G4	G5
-1.4	-0.18	0.69	-0.38	-0.72	1.92	0.7	-1.58	1.59	-1.87
-2.53	-1.31	-2.54	-3.43	-3.77	-1.11	-2.43	-2.98	-3.98	-2.72
-1.12	-0.78	-1.75	-2.6	-2.95	-1.27	-1.75	-1.81	-3.73	-1.19
0.59	0.08	0.01	-0.74	-1.06	-0.54	-0.13	-0.29	-1.62	0.53
1.6	0.67	1.3	0.73	0.51	0.1	1.05	0.61	0.32	1.49
1.68	0.79	1.64	1.33	1.26	0.33	1.37	0.7	1.33	1.51
1.03	0.54	1.14	1.11	1.22	0.17	0.93	0.22	1.39	0.82
0.05	0.08	0.18	0.41	0.68	-0.19	0.1	-0.45	0.82	-0.18
-0.89	-0.37	-0.81	-0.41	0	-0.51	-0.74	-0.95	0.08	-1.09
-1.48	-0.66	-1.51	-1.01	-0.54	-0.65	-1.26	-1.04	-0.48	-1.61
-1.56	-0.73	-1.7	-1.2	-0.75	-0.5	-1.31	-0.67	-0.62	-1.62
-1.14	-0.56	-1.35	-0.94	-0.58	-0.1	-0.88	0.1	-0.3	-1.12
-0.35	-0.23	-0.6	-0.31	-0.1	0.45	-0.1	1.05	0.36	-0.26
0.61	0.17	0.35	0.51	0.53	1	0.79	1.93	1.15	0.71
1.46	0.51	1.22	1.29	1.1	1.37	1.54	2.44	1.78	1.53
1.98	0.68	1.76	1.82	1.43	1.44	1.9	2.4	2	1.97
1.99	0.62	1.78	1.93	1.38	1.11	1.7	1.67	1.63	1.84
1.42	0.36	1.24	1.56	0.93	0.43	0.92	0.37	0.68	1.13
0.39	-0.01	0.26	0.81	0.21	-0.46	-0.3	-1.24	-0.68	-0.04
-0.86	-0.27	-0.86	-0.07	-0.52	-1.29	-1.62	-2.72	-2.08	-1.37
-1.89	-0.14	-1.64	-0.66	-0.84	-1.67	-2.55	-3.41	-2.92	-2.39
-2.15	0.77	-1.44	-0.41	-0.19	-1.1	-2.41	-2.53	-2.44	-2.49
-0.96	2.9	0.51	1.33	2.07	0.96	-0.42	0.81	0.24	-0.96
2.42	6.72	5.02	5.29	6.62	5.08	4.24	7.48	6.07	2.97
8.71	12.69	12.91	12.17	14.15	11.81	12.37	18.31	15.93	10.03
18.56	21.21	24.88	22.59	25.22	21.57	24.68	33.93	30.59	20.86
32.45	32.6	41.44	37.02	40.25	34.64	41.65	54.72	50.56	35.9
50.63	47	62.84	55.68	59.41	51.08	63.49	80.7	75.99	55.35
72.57	64.02	88.36	77.96	82.02	70.26	89.44	110.8	105.9	78.5
100.2	85.17	120.2	105.7	110	93.63	121.6	147.1	142.7	107.2
128.2	106.6	152.1	133.7	137.8	116.6	153.8	182.5	179	135.9
161.5	132.1	189.7	166.7	170.5	143.2	191.5	222.8	221.1	169.5
193.5	156.9	225.4	198	201.3	167.8	226.9	259.4	259.9	200.9
224.6	181.4	259.4	227.8	230.5	190.7	260.3	292.6	295.7	230.4
253.1	204.6	290	254.7	256.6	210.9	289.9	320.4	326.4	256.2
278.2	225.8	316	277.5	278.9	227.8	314.5	341.9	350.5	277.2
299.5	244.6	337.2	296	297.1	241.6	333.6	357.1	368.1	293.1
318.5	261.7	355.5	311.7	313.2	254.6	349.1	368.9	381.2	305.4
337.5	278	373.9	327.2	330.1	269.7	364.1	382.1	394.6	317
363.2	296.9	400.5	349.4	355.2	294.2	386.5	407.1	418.2	335.1

	baseline subtracted fluorescence ratio																											
**FAM**	25.58	19.92	29.68	37.59	26.99	30	28.48	23.71	26.33	18.78	19.08	24.95	16.6	16.59	23.51	25.07	21.93	13.57	14.5	10.39	12.48	12	9.14	9.65	9.94	7.09	5.92	4.99
	26.58	37.79	54.64	67.95	50.61	55.56	53.38	47.73	46.64	34.06	36.94	39.69	33.91	33.36	44.49	45.79	41.82	23.73	26.59	21.97	25.79	23.62	20.69	19.74	19.76	15.04	12.6	12.39
	27.58	60.62	86.97	107.1	81.33	88.84	85.48	78.94	72.58	53.7	60.27	58.38	56.84	55.49	72.1	72.64	67.15	36.77	42.35	37.25	43.5	38.93	36.1	32.89	32.56	25.4	21.7	22.15
	28.56	87.65	125.7	154	118.3	129	123.8	116.3	103.3	77.16	88.5	80.56	84.87	82.45	105.7	104.8	97.11	52.44	61.43	55.89	65.24	57.62	55.03	48.8	48.02	37.93	33.11	34.09
	29.61	121.2	174.5	213	164.8	179.6	171.9	162.9	141.4	106.6	124.4	108.4	120.8	116.9	148.4	145.3	134.4	72.38	85.82	79.76	93.16	81.59	79.41	69.06	67.62	53.88	48.02	49.49
	30.56	155	224.2	273.4	212.4	231.5	220.8	210	179.7	136.8	161.4	136.8	158	152.4	192.6	186.7	172.1	93.14	111.2	104.6	122.2	106.6	104.9	90.06	87.79	70.43	63.78	65.69
	31.63	195.3	284.2	346.6	269.8	294.5	279.6	266	225.2	173.3	206.8	171.5	203.9	196	246.6	236.7	217.2	119	142.8	135.2	158	137.5	136.4	116	112.4	90.87	83.52	86.04
	32.63	234.2	342.9	418.8	326.2	356.4	337	319.7	269	209.4	252	206.1	249.7	239.4	300.2	285.8	261.3	145.5	174.7	166	193.9	168.7	168.2	142	137	111.5	103.6	107
**VIC**	25.58	3.32	4.03	6.25	4.94	8.24	7.77	6.31	5.61	7.34	7.4	8.84	13.08	15.06	14.71	25.43	19.62	12.26	14.13	16.55	15.76	19.42	22.69	24.36	17.59	20.19	17.57	18.56
	26.58	5.41	6.6	9.05	7.75	13.83	12.62	10.97	9.77	12.63	13.26	15.88	24.69	25.82	26.94	41.06	33.73	20.51	24.82	29.57	26.76	34.59	38.64	41.01	29.25	33.51	29.75	32.45
	27.58	8.09	10.11	12.64	11.48	21.44	19.18	17.19	15.22	19.67	21.14	25.37	40.66	40.5	43.54	61.82	52.34	31.22	39.05	47.09	41.33	54.92	59.57	62.69	44.37	50.73	45.69	50.63
	28.56	11.33	14.52	17.02	16.08	31.04	27.39	24.89	21.84	28.31	30.91	37.13	60.75	58.88	64.17	87.34	74.99	44.11	56.47	68.73	59.18	79.89	84.91	88.79	62.53	71.3	64.89	72.57
	29.61	15.45	20.35	22.75	22.09	43.74	38.18	34.9	30.35	39.41	43.6	52.38	87.13	82.86	90.91	120.3	103.9	60.4	78.7	96.55	82.08	111.8	117	121.7	85.36	96.95	89.04	100.2
	30.56	19.73	26.64	28.93	28.46	57.38	49.67	45.47	39.25	50.96	56.95	68.32	115.1	108.1	118.9	154.7	133.6	77.08	101.6	125.4	105.9	144.7	150	155.2	108.6	122.8	113.5	128.2
	31.63	25.02	34.68	36.96	36.53	74.75	64.23	58.73	50.34	65.16	73.58	88.02	150	139.3	153.3	197.2	169.7	97.29	129.3	160.4	135	184.4	189.7	195.5	136.4	153.3	142.7	161.5
	32.63	30.39	43.13	45.59	44.9	92.82	79.26	72.32	61.67	79.32	90.44	107.8	185.4	170.7	187.6	240	205.4	117.2	156.3	194.9	164	222.9	228.5	234.5	163.4	182.4	170.7	193.5
linear regress		7.945	8.02	9.759	7.511	3.861	4.317	4.499	4.343	2.643	2.802	1.823	1.35	1.429	1.597	1.215	1.286	1.251	1.121	0.869	1.223	0.766	0.771	0.628	0.869	0.64	0.636	0.579

2.46	1.52	-4.36	3.63	5.22	-5.52	-6.27	-4.43	-5.75
8.59	6.57	0.63	8.88	10.41	-4.61	-4.19	-2.93	-4.65
16.8	13.34	7.36	15.81	17.02	-2.99	-1.21	-0.46	-2.75
26.95	21.72	15.58	24.27	24.8	-0.59	2.63	2.98	-0.06
40.14	32.61	26.03	35.04	34.44	3	7.76	7.87	3.72
54.11	44.16	36.73	46.15	44.12	7.24	13.32	13.41	7.92
71.75	58.77	49.69	59.69	55.64	13.17	20.48	20.79	13.37
90	73.96	62.47	73.1	66.78	19.9	28.07	28.8	19.09
21.21	24.88	22.59	25.22	21.57	24.68	33.93	30.59	20.86
32.6	41.44	37.02	40.25	34.64	41.65	54.72	50.56	35.9
47	62.84	55.68	59.41	51.08	63.49	80.7	75.99	55.35
64.02	88.36	77.96	82.02	70.26	89.44	110.8	105.9	78.5
85.17	120.2	105.7	110	93.63	121.6	147.1	142.7	107.2
106.6	152.1	133.7	137.8	116.6	153.8	182.5	179	135.9
132.1	189.7	166.7	170.5	143.2	191.5	222.8	221.1	169.5
156.9	225.4	198	201.3	167.8	226.9	259.4	259.9	200.9
0.643	0.359	0.381	0.394	0.42	0.16	0.183	0.167	0.164
