# Supplementary material for: Quantify single nucleotide polymorphism (SNP) ratio in pooled DNA based on normalized fluorescence real-time PCR
Source: BMC Genomics. 2006 Jun 9;7:143. doi: 10.1186/1471-2164-7-143 (PMC1552069; doi:10.1186/1471-2164-7-143)
Supplement: Additional file 7 — Contained the raw and analytical datas used during the procession. provide comparative ΔCt method for each allele frequency measurement. [file 1471-2164-7-143-S7.pdf]

FAM	Well	Ct	average Ct of each allele	VIC	Well	Ct	average Ct of each allele	ΔCt	$2^{-\Delta Ct}$	Av.($2^{-\Delta Ct}$)	SD.($2^{-\Delta Ct}$)	predefined ratio
	B5	29.19			C2	30.49		-1.30001	2.46	2.62	0.36	9
	B6	29.43			C3	31.04		-1.60122	3.03			
	B7	28.89			C4	30.13		-1.24097	2.36			
			29.17		C5		30.55					
	B8	28.91			B8	30.31		-1.40021	2.64	2.22	0.59	4
	B9	29.34			B9	30.19		-0.84846	1.80			
			29.13				30.25					
	C3	29.67			C3	30.43		-0.76143	1.70	1.63	0.19	2.333333333
	C4	29.67			C4	30.50		-0.83047	1.78			
	C5	29.61			C5	30.10		-0.4948	1.41			
			29.65				30.34					
	C6	29.73			C6	30.16		-0.42628	1.34	1.29	0.05	1.5
	C7	29.66			C7	30.00		-0.34045	1.27			
	C8	29.46			C8	29.80		-0.33776	1.26			
			29.62				29.99					
	C9	29.48			C9	29.50		-0.01561	1.01	0.85	0.19	1
	D2	30.13			D2	29.49		0.6342	0.64			
	D3	30.09			D3	29.95		0.140832	0.91			
			29.90				29.65					
	D4	30.63			D4	29.19		1.445151	0.37	0.66	0.25	0.666666667
	D5	29.99			D5	29.65		0.336315	0.79			
	D6	29.86			D6	29.55		0.309825	0.81			
			30.16				29.46					
	D7	29.83			D7	29.06		0.76997	0.59	0.54	0.04	0.428571429
	D8	30.51			D8	29.53		0.981767	0.51			
	D9	30.36			D9	29.44		0.916065	0.53			
			30.23				29.34					
	E2	30.24			E2	28.76		1.486783	0.36	0.34	0.05	0.25
	E3	30.64			E3	29.19		1.446548	0.37			
	E4	30.97			E4	29.15		1.823193	0.28			
			30.62				29.03					
	E5	37.35			E5	29.14		8.218469	0.00	0.15	0.14	0.111111111
	E6	31.86			E6	29.06		2.792484	0.14			
	E7	30.91			E7	29.13		1.779733	0.29			
			33.37				29.11					
