# Supplementary material for: Quantify single nucleotide polymorphism (SNP) ratio in pooled DNA based on normalized fluorescence real-time PCR
Source: BMC Genomics. 2006 Jun 9;7:143. doi: 10.1186/1471-2164-7-143 (PMC1552069; doi:10.1186/1471-2164-7-143)
Supplement: Additional file 8 — Contained the raw and analytical datas used during the procession. provide comparative ΔCt method for each allele frequency measurement. [file 1471-2164-7-143-S8.pdf]

FAM	Well	Ct	average Ct of each allele	VIC	Well	Ct	verage C f each alle	ΔCt	$2^{-\Delta Ct}$	Av.($2^{-\Delta Ct}$)	SD.($2^{-\Delta Ct}$)	predefined ratio
	A7	30.34			A7	31.53		-1.19	2.28			
	A8	30.01			A8	31.30		-1.29	2.45	2.26	0.86	9.00
	A9	29.87			A9	31.55		-1.68	3.21			
	B2	30.59	30.20		B2	30.76	31.29	-0.17	1.13			
	B4	30.43			B4	31.17		-0.74	1.67			
	B5	30.31			B5	30.69		-0.39	1.31	1.77	0.36	4.00
	B6	30.24			B6	31.29		-1.06	2.08			
	B8	30.20	30.29		B8	31.23	31.10	-1.03	2.04			
	B9	30.35			B9	31.05		-0.70	1.63			
	C3	30.66			C3	31.16		-0.50	1.41	1.53	0.15	2.33
	C4	30.63			C4	31.39		-0.76	1.70			
	C5	30.64	30.57		C5	31.11	31.18	-0.47	1.39			
	C6	30.47			C6	30.79		-0.31	1.24			
	C7	30.43			C7	30.64		-0.21	1.16	1.25	0.09	1.50
	C8	30.48			C8	30.77		-0.29	1.23			
	C9	30.42	30.45		C9	30.88	30.77	-0.46	1.38			
	D3	30.94			D3	30.60		0.35	0.79			
	D4	31.84			D4	30.59		1.25	0.42			
	D5	30.53			D5	30.72		-0.19	1.14	0.82	0.26	1.00
	D6	31.06			D6	30.86		0.21	0.87			
	D7	30.96	31.07		D7	30.79	30.71	0.17	0.89			
	D8	31.10			D8	30.76		0.34	0.79			
	D9	31.12			D9	30.67		0.45	0.73			
	E2	30.92			E2	30.29		0.64	0.64	0.70	0.09	0.67
	E3	31.14			E3	30.75		0.38	0.77			
	E4	31.07	31.07		E4	30.25	30.54	0.82	0.57			
	E5	31.30			E5	30.37		0.93	0.52			
	E6	31.89			E6	30.57		1.33	0.40			
	E7	31.71			E7	30.59		1.12	0.46	0.48	0.05	0.43
	E8	31.31			E8	30.40		0.92	0.53			
	E9	31.37	31.52		E9	30.36	30.46	1.02	0.49			
	F2	31.84			F2	30.27		1.57	0.34			
	F3	31.89			F3	30.35		1.54	0.34			
	F4	31.58			F4	30.22		1.36	0.39	0.39	0.05	0.25
	F5	31.58			F5	30.27		1.31	0.40			
	F6	31.44	31.66		F6	30.33	30.29	1.11	0.46			
	F7	32.86			F7	29.33		3.53	0.09			
	F8	33.55			F8	30.23		3.32	0.10			
	F9	32.89			F9	29.93		2.96	0.13	0.12	0.03	0.11
	G2	32.47			G2	29.53		2.95	0.13			
	G3	32.79	32.91		G3	30.06	29.82	2.73	0.15			
