# Supplementary material for: Quantify single nucleotide polymorphism (SNP) ratio in pooled DNA based on normalized fluorescence real-time PCR
Source: BMC Genomics. 2006 Jun 9;7:143. doi: 10.1186/1471-2164-7-143 (PMC1552069; doi:10.1186/1471-2164-7-143)
Supplement: Additional file 9 — Contained the raw and analytical datas used during the procession. provide comparative ΔCt method for each allele frequency measurement. [file 1471-2164-7-143-S9.pdf]

FAM	Well	Ct	average Ct of each allel	VIC	Well	Ct	average Ct of each allele	ΔCt	$2^{-\Delta Ct}$	Av.($2^{-\Delta Ct}$)	SD.($2^{-\Delta Ct}$)	edefined rat
	C2	28.62			C2	31.99		-3.37	10.34	6.46	3.06	9.00
	C3	28.93			C3	31.47		-2.54	5.82			
	C4	29.20			C4	31.96		-2.76	6.77			
	C5	29.17	28.98		C5	30.72	31.54	-1.55	2.93			
	C6	29.04			C6	30.56		-1.52	2.87	2.73	0.09	4.00
	C7	28.94			C7	30.36		-1.42	2.68			
	C8	28.88			C8	30.31		-1.43	2.69			
	C9	28.86	28.93		C9	30.29	30.38	-1.43	2.69			
	D2	29.14			D2	29.64		-0.50	1.41	1.46	0.14	2.33
	D3	29.32			D3	29.98		-0.66	1.58			
	D4	29.32			D4	29.68		-0.36	1.28			
	D5	29.52	29.33		D5	30.15	29.86	-0.63	1.55			
	D6	29.38			D6	29.53		-0.15	1.11	1.09	0.27	1.50
	D7	29.48			D7	29.53		-0.05	1.04			
	D8	29.25			D8	29.76		-0.51	1.42			
	D9	29.86	29.49		D9	29.50	29.58	0.36	0.78			
	E2	29.93			E2	29.08		0.85	0.55	0.65	0.07	1.00
	E3	29.74			E3	29.11		0.63	0.65			
	E4	29.67			E4	29.16		0.51	0.70			
	E5	29.71	29.76		E5	29.18	29.13	0.53	0.69			
	E6	29.96			E6	29.02		0.94	0.52	0.51	0.04	0.67
	E7	30.16			E7	29.01		1.15	0.45			
	E8	29.71			E8	28.82		0.89	0.54			
	E9	29.76	29.90		E9	28.89	28.94	0.87	0.55			
	F2	29.70			F2	28.45		1.25	0.42	0.32	0.08	0.43
	F3	30.37			F3	28.68		1.69	0.31			
	F4	30.41			F4	28.75		1.66	0.32			
	F5	30.67	30.29		F5	28.52	28.60	2.15	0.23			
	F6	30.36			F6	28.37		1.99	0.25	0.43	0.18	0.25
	F7	30.02			F7	28.33		1.69	0.31			
	F8	29.27			F8	28.27		1.00	0.50			
	F9	28.64	29.57		F9	28.01	28.25	0.63	0.65			
	G3	32.58			G3	27.60		4.98	0.03	0.01	0.02	0.11
	G4	33.45			G4	27.92		5.53	0.02			
	G5	37.81			G5	28.12		9.69	0.00			
	G6	40.00	35.96		G6	28.13	27.94	11.87	0.00			
