# Supplementary material for: Quantify single nucleotide polymorphism (SNP) ratio in pooled DNA based on normalized fluorescence real-time PCR
Source: BMC Genomics. 2006 Jun 9;7:143. doi: 10.1186/1471-2164-7-143 (PMC1552069; doi:10.1186/1471-2164-7-143)

**YMDD**	9.00	8.00	7.00	6.00	5.00	4.00	3.00	2.00	1.00
**YIDD**	1.00	2.00	3.00	4.00	5.00	6.00	7.00	8.00	9.00
**ratio**	9.00	4.00	2.33	1.50	1.00	0.67	0.43	0.25	0.11
**Run 1 K_f**	2.99	1.64	0.97	0.70	0.48	0.26	0.22	0.18	0.01
**041210**	2.75	1.44	1.07	0.68	0.44	0.21	0.20	0.15	0.04
	2.83		0.98	0.66	0.49	0.37	0.20	0.10	0.05
**AV.**	2.86	1.54	1.00	0.68	0.47	0.28	0.20	0.14	0.03
**S.D.**	0.12	0.14	0.05	0.02	0.03	0.08	0.01	0.04	0.02
**intra C.V.**	4.38%	9.18%	5.40%	2.99%	6.06%	28.91%	4.70%	29.02%	61.69%
**Run 2 K_f**	2.80	1.32	0.97	0.67	0.40	0.21	0.15	0.17	0.04
**041211**	3.01	1.57	0.90	0.62	0.26	0.21	0.13	0.12	0.03
	2.68	1.55	1.03	0.69	0.22	0.30	0.13	0.12	0.02
	2.83	1.94	0.90	0.66	0.27	0.37	0.15	0.12	0.04
					0.36	0.22	0.19	0.11	0.03
**AV.**	2.83	1.60	0.95	0.66	0.30	0.26	0.15	0.13	0.03
**S.D.**	0.14	0.26	0.06	0.03	0.08	0.07	0.02	0.02	0.01
**intra C.V.**	4.90%	16.15%	6.66%	4.44%	24.75%	27.05%	15.97%	18.71%	22.86%
**Run 3 Kf**	2.61	1.28	0.92	0.57	0.41	0.30	0.22	0.14	0.06
**041215**	2.61	1.42	0.91	0.57	0.41	0.29	0.22	0.15	0.06
	3.12	1.43	0.76	0.51	0.36	0.27	0.21	0.15	0.08
	2.37	1.45	0.62	0.52	0.43	0.33	0.22	0.15	0.07
**AV.**	2.68	1.40	0.80	0.54	0.40	0.30	0.22	0.15	0.07
**S.D.**	0.32	0.08	0.14	0.03	0.03	0.02	0.00	0.01	0.01
**intra C.V.**	0.12	0.05	0.17	0.06	0.07	0.08	0.02	0.04	0.10
**inter Av.**	2.78	1.50	0.91	0.62	0.38	0.28	0.19	0.14	0.05
**inter S.D.**	0.21	0.19	0.12	0.07	0.09	0.06	0.04	0.02	0.02
**inter C.V.**	7.69%	12.54%	13.64%	10.91%	23.71%	20.61%	20.19%	17.82%	44.25%

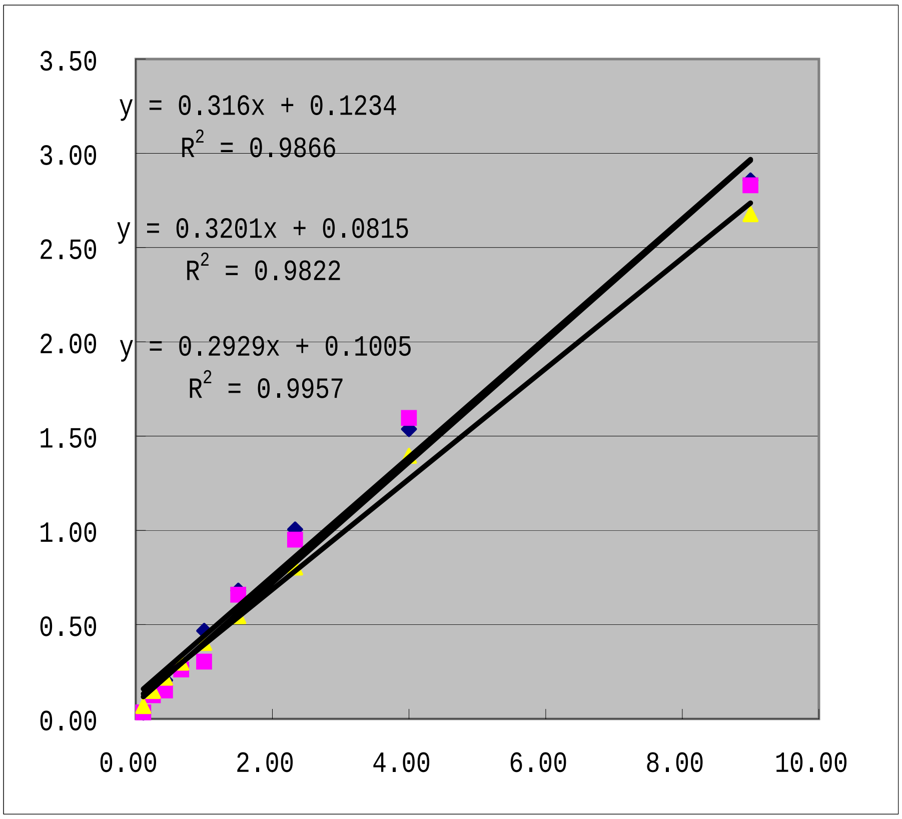

Supplement: Additional file 10 — Contained the raw and analytical datas used during the procession. provide detailed intra and inter CV values of three compared methods. [file 1471-2164-7-143-S10.pdf]
