# Supplementary material for: Quantify single nucleotide polymorphism (SNP) ratio in pooled DNA based on normalized fluorescence real-time PCR
Source: BMC Genomics. 2006 Jun 9;7:143. doi: 10.1186/1471-2164-7-143 (PMC1552069; doi:10.1186/1471-2164-7-143)

**YMDD**	9.00	8.00	7.00	6.00	5.00	4.00	3.00	2.00	1.00
**YIDD**	1.00	2.00	3.00	4.00	5.00	6.00	7.00	8.00	9.00
**ratio**	9.00	4.00	2.33	1.50	1.00	0.67	0.43	0.25	0.11
**Run 1**	5.38	3.07	2.44	1.28	0.90	0.41	0.43	0.37	0.03
**041210**	4.46	3.31	1.79	1.18	0.81	0.26	0.41	0.30	
	4.85		1.80	1.19	1.00	0.52	0.37	0.17	0.13
**AV.**	4.90	3.19	2.01	1.21	0.90	0.40	0.40	0.28	0.08
**S.D.**	0.46	0.17	0.37	0.05	0.10	0.13	0.03	0.10	0.07
**intra C.V.**	9.39%	5.30%	18.48%	4.41%	10.59%	33.55%	7.38%	36.42%	81.13%
**Run 2**	4.49	2.59	2.11	1.14	0.81	0.44	0.29	0.26	0.07
**041211**	4.82	2.69	1.16	1.06	0.40	0.34	0.22	0.23	0.07
	4.95	2.69	2.15	1.29	0.28	0.60	0.24	0.21	0.08
	4.51	3.15	1.60	1.31	0.35	0.73	0.22	0.20	0.11
					0.69	0.35	0.35	0.24	0.10
**AV.**	4.69	2.78	1.75	1.20	0.51	0.49	0.26	0.23	0.08
**S.D.**	0.23	0.25	0.47	0.12	0.23	0.17	0.06	0.02	0.02
**intra C.V.**	4.91%	9.11%	26.65%	10.11%	45.08%	34.65%	22.71%	10.93%	21.84%
**Run 3**	7.94	3.86	2.64	1.43	1.25	0.77	0.64	0.36	0.16
**041215**	8.02	4.32	2.80	1.60	1.12	0.77	0.64	0.38	0.18
	9.76	4.50	1.82	1.21	0.87	0.63	0.58	0.39	0.17
	7.51	4.34	1.35	1.29	1.22	0.87	0.64	0.42	0.16
**AV.**	8.31	4.25	2.15	1.38	1.12	0.76	0.62	0.39	0.17
**S.D.**	0.99	0.27	0.69	0.17	0.17	0.10	0.03	0.03	0.01
**intra C.V.**	11.95%	6.45%	31.87%	12.22%	15.59%	13.09%	4.89%	6.57%	5.98%
**inter Av.**	6.06	3.45	1.97	1.27	0.82	0.58	0.42	0.30	0.12
**inter S.D.**	1.88	0.74	0.52	0.15	0.34	0.20	0.17	0.09	0.05
**inter C.V.**	30.98%	21.53%	26.26%	11.55%	41.63%	34.48%	41.05%	30.18%	44.52%

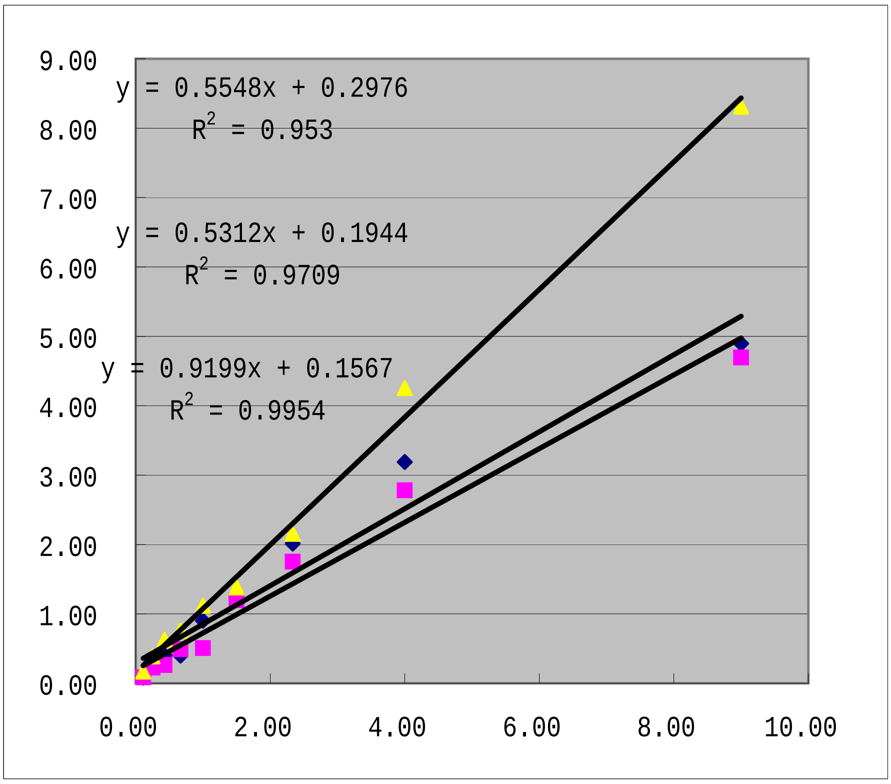

Supplement: Additional file 11 — Contained the raw and analytical datas used during the procession. provide detailed intra and inter CV values of three compared methods. [file 1471-2164-7-143-S11.pdf]
