# Supplementary material for: Quantify single nucleotide polymorphism (SNP) ratio in pooled DNA based on normalized fluorescence real-time PCR
Source: BMC Genomics. 2006 Jun 9;7:143. doi: 10.1186/1471-2164-7-143 (PMC1552069; doi:10.1186/1471-2164-7-143)

**YMDD**	9.00	8.00	7.00	6.00	5.00	4.00	3.00	2.00	1.00
**YIDD**	1.00	2.00	3.00	4.00	5.00	6.00	7.00	8.00	9.00
**ratio**	9.00	4.00	2.33	1.50	1.00	0.67	0.43	0.25	0.11
**Run 1 valu**	2.46	2.64	1.70	1.34	1.01	0.37	0.59	0.36	0.00
**041210**	3.03	1.80	1.78	1.27	0.64	0.79	0.51	0.37	0.14
	2.36		1.41	1.26	0.91	0.81	0.53	0.28	0.29
**AV.**	2.62	2.22	1.63	1.29	0.85	0.66	0.54	0.34	0.15
**S.D.**	0.36	0.59	0.19	0.05	0.19	0.25	0.04	0.05	0.14
**intra C.V.**	13.81%	26.72%	11.90%	3.52%	22.12%	38.09%	7.61%	13.72%	98.39%
**Run 2 valu**	2.28	1.67	1.63	1.24	0.79	0.79	0.52	0.34	0.09
**041211**	2.45	1.31	1.41	1.16	0.42	0.73	0.40	0.34	0.10
	3.21	2.08	1.70	1.23	1.14	0.64	0.46	0.39	0.13
	1.13	2.04	1.39	1.38	0.87	0.77	0.53	0.40	0.13
					0.89	0.57	0.49	0.46	0.15
**AV.**	2.26	1.77	1.53	1.25	0.82	0.70	0.48	0.39	0.12
**S.D.**	0.86	0.36	0.15	0.09	0.26	0.09	0.05	0.05	0.03
**intra C.V.**	37.98%	20.39%	10.05%	7.41%	31.72%	13.30%	11.17%	13.23%	21.62%
**Run 3 valu**	10.34	2.87	1.41	1.11	0.55	0.52	0.42	0.25	0.03
**041215**	5.82	2.68	1.58	1.04	0.65	0.45	0.31	0.31	0.02
	6.77	2.69	1.28	1.42	0.70	0.54	0.32	0.50	0.00
	2.93	2.69	1.55	0.78	0.69	0.55	0.23	0.65	0.00
**AV.**	6.46	2.73	1.46	1.09	0.65	0.51	0.32	0.43	0.01
**S.D.**	3.06	0.09	0.14	0.27	0.07	0.04	0.08	0.18	0.02
**intra C.V.**	47.29%	3.30%	9.32%	24.43%	10.38%	8.56%	25.12%	42.29%	113.30%
**inter Av.**	3.89	2.25	1.53	1.20	0.76	0.63	0.44	0.38	0.09
**inter S.D.**	2.69	0.54	0.16	0.18	0.21	0.16	0.11	0.11	0.09
**inter C.V.**	69.23%	23.96%	10.36%	15.06%	27.37%	24.53%	26.06%	28.82%	103.45%

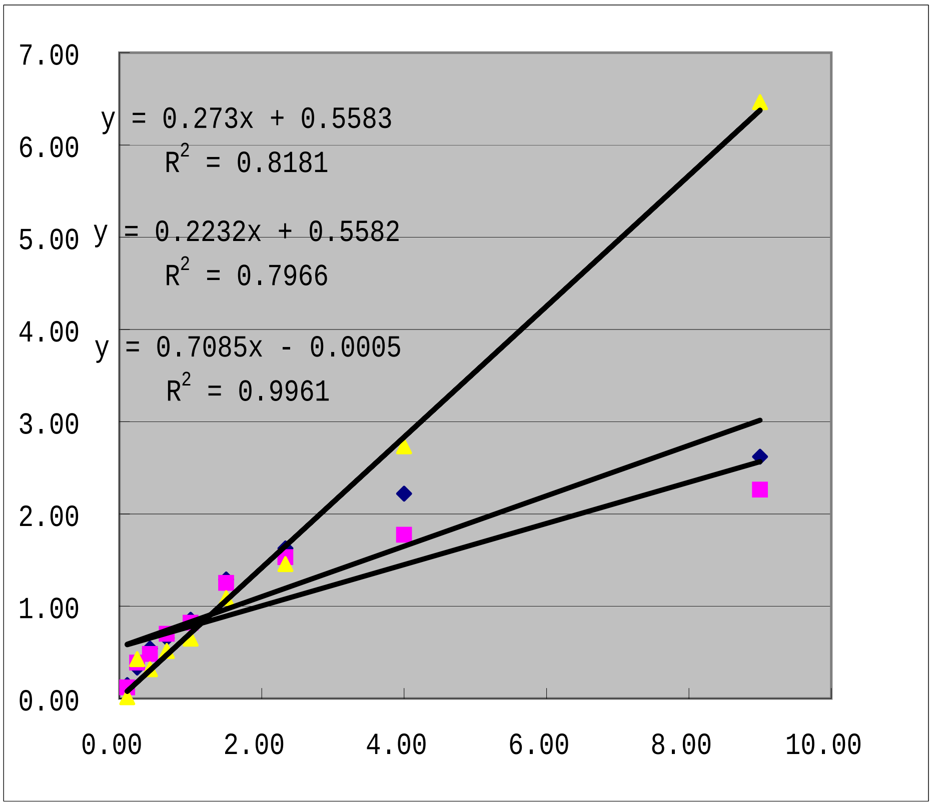

Supplement: Additional file 12 — Contained the raw and analytical datas used during the procession. provide detailed intra and inter CV values of three compared methods. [file 1471-2164-7-143-S12.pdf]
